# Study of the active ingredients and mechanism of *Sparganii rhizoma* in gastric cancer based on HPLC-Q-TOF–MS/MS and network pharmacology

**DOI:** 10.1038/s41598-021-81485-0

**Published:** 2021-01-21

**Authors:** Xiaona Lu, Yawei Zheng, Fang Wen, Wenjie Huang, Xiaoxue Chen, Shuai Ruan, Suping Gu, Yue Hu, Yuhao Teng, Peng Shu

**Affiliations:** 1 Oncology Department, Affiliated Hospital of Nanjing University of Chinese Medicine, Nanjing, China; 2 First School of Clinical Medicine, Nanjing University of Chinese Medicine, Nanjing, China

**Keywords:** Cancer, Computational biology and bioinformatics, Systems biology

## Abstract

*Sparganii rhizoma* (SL) has potential therapeutic effects on gastric cancer (GC), but its main active ingredients and possible anticancer mechanism are still unclear. In this study, we used HPLC-Q-TOF–MS/MS to comprehensively analyse the chemical components of the aqueous extract of SL. On this basis, a network pharmacology method incorporating target prediction, gene function annotation, and molecular docking was performed to analyse the identified compounds, thereby determining the main active ingredients and hub genes of SL in the treatment of GC. Finally, the mRNA and protein expression levels of the hub genes of GC patients were further analysed by the Oncomine, GEPIA, and HPA databases. A total of 41 compounds were identified from the aqueous extract of SL. Through network analysis, we identified seven main active ingredients and ten hub genes: acacetin, sanleng acid, ferulic acid, methyl 3,6-dihydroxy-2-[(2-hydroxyphenyl) ethynyl]benzoate, caffeic acid, adenine nucleoside, azelaic acid and PIK3R1, PIK3CA, SRC, MAPK1, AKT1, HSP90AA1, HRAS, STAT3, FYN, and RHOA. The results indicated that SL might play a role in GC treatment by controlling the PI3K-Akt and other signalling pathways to regulate biological processes such as proliferation, apoptosis, migration, and angiogenesis in tumour cells. In conclusion, this study used HPLC-Q-TOF–MS/MS combined with a network pharmacology approach to provide an essential reference for identifying the chemical components of SL and its mechanism of action in the treatment of GC.

## Introduction

Gastric cancer (GC) is one of the leading causes of cancer-related death worldwide, and its incidence rate is sixth among cancers^1^. At present, surgery, chemotherapy, and other traditional therapies are the main treatments. However, the incidence of local recurrence and distant metastasis after gastric cancer surgery is high. Chemotherapy is associated with toxicity and side effects; thus, it is challenging for these treatments to mediate a long-term antitumour effect. Therefore, it is necessary to explore new strategies for the treatment of this disease. In China, traditional Chinese medicine (TCM) is widely used in the treatment of GC and has shown advantages with its multipathway, multitarget, and multilink characteristics, small side effects, and significant efficacy. *Sparganii rhizoma* (SL) is the dried tuber of the Sparganiaceae plant *Sparganium stoloniferum* (Buch.-Ham. ex Graebn.) Buch.-Ham. ex Juz., which is a traditional Chinese medicine. It has a pungent, bitter, flat attributes and enters the liver and spleen meridians. Its effects include tonifying the blood and promoting qi, removing stagnant food, and alleviating pain. It is included in the *Pharmacopoeia of the People's Republic of China* (2015 Edition)^2^. Previous experiments by our research team suggested that the “*Sparganii rhizoma*-*Curcuma zedoary*-*Salvia chinensis*” herb pair with SL as one of the main components had growth-inhibitory effects on both regular and resistant GC cells, and the inhibitory effect increased with increasing concentration^3^. Modern pharmacological studies have also shown that SL has an apparent inhibitory effect on the proliferation of GC cells and can promote tumour cell apoptosis^4^. In addition, some studies have found that the combination of traditional Chinese medicine preparations mainly composed of SL and chemotherapy can prolong the progression-free survival (PFS) of patients with advanced gastric cancer, improve the quality of life of patients, and reduce the adverse reactions to chemotherapy^5^. Although previous studies have shown that SL has potential therapeutic effects on GC, its main active ingredients and possible anticancer mechanism are still unclear.

**Figure 1:**
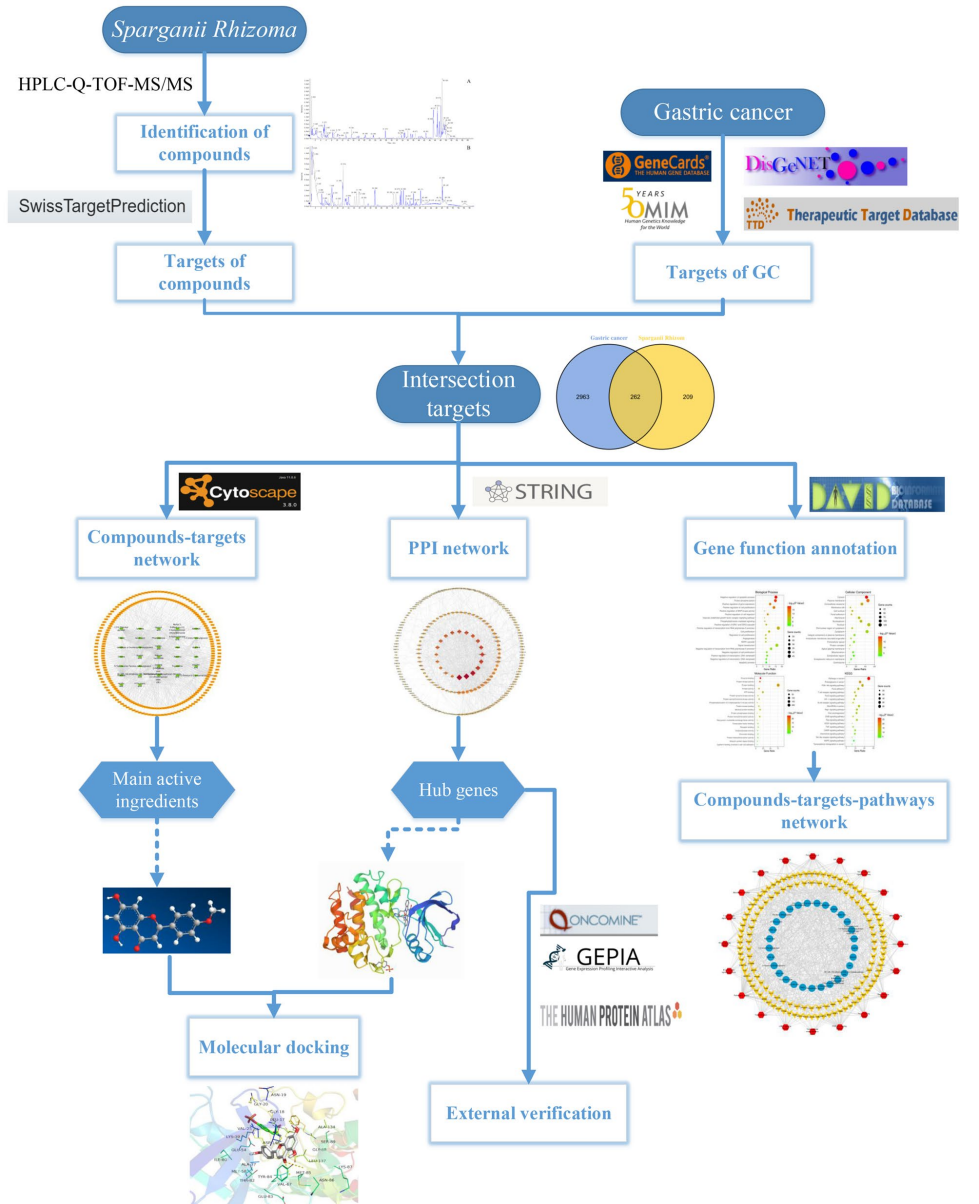
Scheme of analysis procedure.

High-performance liquid chromatography coupled with quadrupole time-of-flight mass spectrometry (HPLC-Q-TOF–MS), which is a common qualitative and quantitative analysis technology combining liquid chromatography and mass spectrometry, can be used to analyse the structure of trace components in crude substances without a reference substance^6^. Both positive and negative ionization modes have been used to confirm the related chemical compounds and their characteristic fragment ions according to the accurate molecular mass information of the excimer ion peaks and the fragment ions. Then, compounds are ultimately determined by comparisons with the relevant database. HPLC-Q-TOF–MS/MS is characterized by high resolution, high sensitivity, high selectivity, short response time, wide scanning range, high molecular mass accuracy, and an ability to obtain multistage mass spectrum fragment information for compounds. It can quickly analyse and identify the structures of complex substances such as TCM and is very convenient for basic research on TCM materials^7,8^. Network pharmacology is a method for predicting the pharmacological mechanism of drug treatments for diseases based on the theory of systems biology and the use of complex biological network models, starting from the integrity and systematic nature of interactions among drugs, chemical components, targets, and diseases^9,10^. Its holistic and systematic characteristics are consistent with the principles of the holistic view, syndrome differentiation and treatment of TCM, which have been widely used in the study of TCM^11,12^. For example, Yucheng Guo et al. used a network pharmacology research method to construct a multiscale mathematical model of inflammation-induced tumorigenesis, further identified the key biological molecular network and genetic interaction module from the dynamic evolution path of inflammation and cancer, and predicted the TCM ingredients that can inhibit inflammation-induced tumorigenesis. This method is of great value for the accurate prevention and treatment of cancer and the modernization of TCM^13,14^. Therefore, in this study, HPLC-Q-TOF–MS/MS was used to rapidly analyse and identify the chemical components in SL, and the mechanism of SL in the treatment of GC was explored by combining network pharmacology research methods. The specific flowchart is shown in Fig. 1.

## Results

### Identification of the chemical components of SL

We analysed SL aquatic extract samples based on the above conditions of liquid chromatography and mass spectrometry. We used positive and negative ion mode scanning in this paper to obtain as much information as possible. The exact mass-to-charge ratio (m/z) of the compound was obtained by TOF–MS, while the second-order fragment ion of this mass number was obtained by product ion secondary mass spectrometry. By using online databases, referring to the relevant literature and considering the fragmentation rule of compounds, we qualitatively analysed the structures of SL-related compounds. Forty-one compounds were ultimately identified: nine phenylpropanoids, eight organic acids, four flavonoids, four amino acids, two alkaloids, and fourteen other compounds. The secondary mass spectra of each compound are shown in the “Supplementary Figures”. Table 1 shows the retention time, mass spectrometry information, and related references of the identified compounds.

**Table 1:** The compounds identified of *Sparganii rhizoma.*

No.	Rt (min)	Quasi-molecular (p) [M + H]^+^ = [M + Na]^+^ (Error, ppm)	Quasi-molecular (n) [M − H]¯ = [M + Cl/COOH]^−^ (Error, ppm)	Molecular formula	MS/MS fragments (p)	MS/MS fragments (n)	Proposed compound	References
SL1	0.55	104.1072 (2.0)		C5H13NO	60, 58		Choline	^15^
SL2	0.57	175.1184 (− 3.2)		C6H14N4O2	130, 116, 70		Arginine	^15^
SL3	0.62	138.0545 (− 3.4)		C7H7NO2	94, 93, 92, 78, 65		Trigonelline	^15^
SL4	0.86	136.0617 (− 2.7)		C5H5N5	119, 94, 92, 77, 67, 65		Adenine	^15^
SL5	0.86	124.0394 (− 2.5)		C6H5NO2	106, 80, 78, 53, 52		Nicotinic acid	^15^
SL6	1.27	113.0346 (− 3.1)		C4H4N2O2	96, 95, 70,68,53		Uracil	^15^
SL7	1.35		117.0195 (1.4)	C4H6O4		117, 100, 73	Succinic acid	^16^
SL8	1.65	182.0812 (0.2)		C9H11NO3	165, 136, 123, 119, 95, 91, 77		Tyrosine	^15^
SL9	1.62	132.1015 (− 3.1)		C6H13NO2	86, 69, 57, 56		D-Tert-Leucine	^16^
SL10	2.13	268.1038 (− 1.2)		C10H13N5O4	136, 119		Adenine nucleoside	^15^
SL11	4.14		167.0352 (1.3)	C8H8O4		149, 137, 123, 121, 109, 108, 93, 81, 69	Vanillic acid	^16^
SL12	4.19	166.0853 (− 1.5)		C9H11NO2	120, 103, 91, 77, 51		Phenylalanine	^16^
SL13	6.07		137.0249 (3.5)	C7H6O3		109, 108, 93, 81, 65	4-Hydroxybenzoic acid	^16^
SL14	7.37	227.1028 (0.7)		C10H14N2O5	209, 181, 116, 84, 70		Carbidopa	^17^
SL15	9.76		179.0349 (5.7)	C9H8O4		135, 134, 117, 91, 71, 59	Caffeic acid	^18^
SL16	10.02	123.0439 (− 1.3)		C7H6O2	95, 77, 65, 51		Benzoic acid	^16^
SL17	10.44		253.0716 (− 0.6)	C12H14O6		179, 161, 135, 133	Hwanggeumchal B	^19^
SL18	12.88		445.1336 (− 3.5)	C19H26O12		383, 343, 301, 283, 139, 125, 124, 99	4-hydroxy-2-methoxyphenyl 1-O-[6-(hydrogen 3-hydroxy-3-methylpentanedioate)]-*β*-d-glucopyranoside	^20^
SL19	13.57		253.0716 (− 0.6)	C12H14O6		179, 161, 135, 133	1-Caffeoylglycerol	–
SL20	13.69		237.0764 (− 1.9)	C12H14O5		145, 119, 117, 59	2-Propenoic acid, 3-(4-hydroxyphenyl)-,2,3-dihydroxypropyl ester, (E)-	^21^
SL21	15.55		163.0404 (2.0)	C9H8O3		119, 117, 93	4-Coumaric acid	^16^
SL22	17.72		237.0768 (− 0.2)	C12H14O5		237, 163, 145, 119, 117	1-O-p-coumaroylglycerol	^21^
SL23	17.74		193.0505 (5.0)	C10H10O4		178, 134, 133	Ferulic acid	^22^
SL24	20.88		267.0873 (− 0.4)	C13H16O6		252, 193, 175, 160, 149, 134, 133, 105, 77	2-Propenoic acid, 3(4-hydroxy-3-methoxyphenyl)-,2,3-dihydroxypropyl ester, (2Z)-	^21^
SL25	21.11		267.0875 (0.3)	C13H16O6		252, 193, 175, 160, 149, 134, 133, 105, 77	1-O-Trans-Feruloylglycerol	^21, 23^
SL26	24.37		219.0661 (4.2)	C12H12O4		220, 202, 185, 175, 167, 147,	Decarboxy-citrinone	^24^
SL27	29.2		609.1450 (− 1.8)	C27H30O16		301, 300, 271, 151	Rutin	^16^
SL28	29.26	447.1279 (− 1.5)		C22H22O10	285, 270, 253		Tilianin	^25^
SL29	29.55		261.1337 (− 2.5)	C12H22O6		187, 169, 125, 123, 97	9-(2′,3′-Dihydroxypropyloxy)-9-oxononanoic Acid	^26^
SL30	30.18		187.0980 (2.2)	C9H16O4		187, 169, 143, 125, 123, 97, 57	Azelaic acid	^16^
SL31	32.53		623.1621 (0.5)	C28H32O16		315, 314, 300, 299, 271, 243	Narcissin	^16^
SL32	35.33	381.1180 (0.0)		C18H20O9	177, 145, 117, 89		β-d-Glucopyranosiduronic acid, 4-methyl-2-oxo-2H-1-benzopyran-7-yl, ethyl ester	–
SL33	35.66		283.0612 (0.0)	C16H12O5		268, 239, 224, 211	Acacetin	^27^
SL34	35.75		429.1168 (− 2.8)	C22H22O9		267, 253, 235, 193, 179, 161, 149, 135, 134, 133, 117	Feruloyl-caffeoylglycerol	^28^
SL35	37.29		413.1224 (− 4.3)	C22H22O8		193, 163, 134, 119, 117	*p*-Coumaroyl-feruloylglycerol	^28^
SL36	37.42		381.1131 (1.5)	C21H20O7		237, 219, 163, 145, 119, 117	1,3-O-Di-trans-p-coumaroylglycerol	^29^
SL37	37.43		443.1324 (− 5.3)	C23H24O9		193, 134	1,3-O-Diferuloyl glycerol	^16^
SL38	37.45		413.1226 (− 1.2)	C22H22O8		267, 249, 235, 219, 193, 177, 163, 145, 134, 119	1-O-Feruloyl-3-O-p-coumaroylglycerol	^16^
SL39	38.88		283.0605 (− 2.5)	C16H12O5		251, 239, 233, 207, 195, 179, 167, 151	Methyl 3, 6-dihydroxy-2-[(2-hydroxyphenyl) ethynyl]benzoate	^16^
SL40	40.359		327.21714 (1.6)	C18H31O5		327, 309, 291, 239, 229, 221, 211, 183, 171	9S,12R,13S-Trihydroxy-10E,15Zoctadecadienoic acid	^30^
SL41	41.74		329.2320 (− 4.1)	C18H34O5		329, 311, 229, 211, 209, 193, 183, 171	Sanleng acid	^22^

### Network pharmacology analysis

#### Prediction of potential targets of compounds and collection of targets for GC

**Figure 2:**
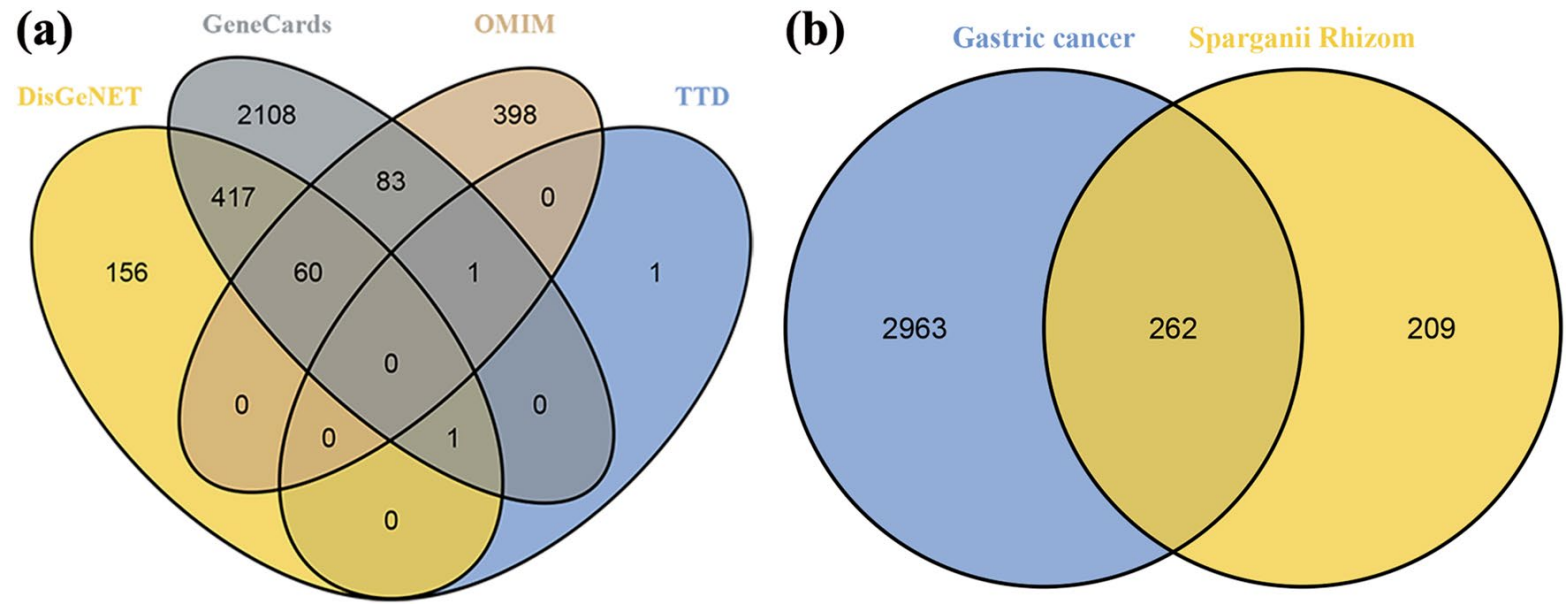
Target maps of *Sparganii rhizoma* and gastric cancer. (**a**) Gastric cancer targets in different disease databases. (**b**) Venn diagram of *Sparganii rhizoma* and gastric cancer targets.

SwissTargetPrediction predicted a total of 1157 potential targets of the 41 compounds identified by mass spectrometry, and we obtained 471 after removing duplicate targets (Supplementary Table S1). We retrieved data from the GeneCards, OMIM, DisGeNET, and TTD databases and identified 2670, 542, 634, and 3 GC-related targets after screening, respectively, which resulted in 3225 targets after merging and removal of duplicate targets (Supplementary Table S2) (Fig. 2a). Potential mapping of the targets of compounds resulted in a total of 262 common targets with those related to GC, which were ultimately identified as target genes of SL for the treatment of GC (Fig. 2b).

#### Compound-target network analysis

**Figure 3:**
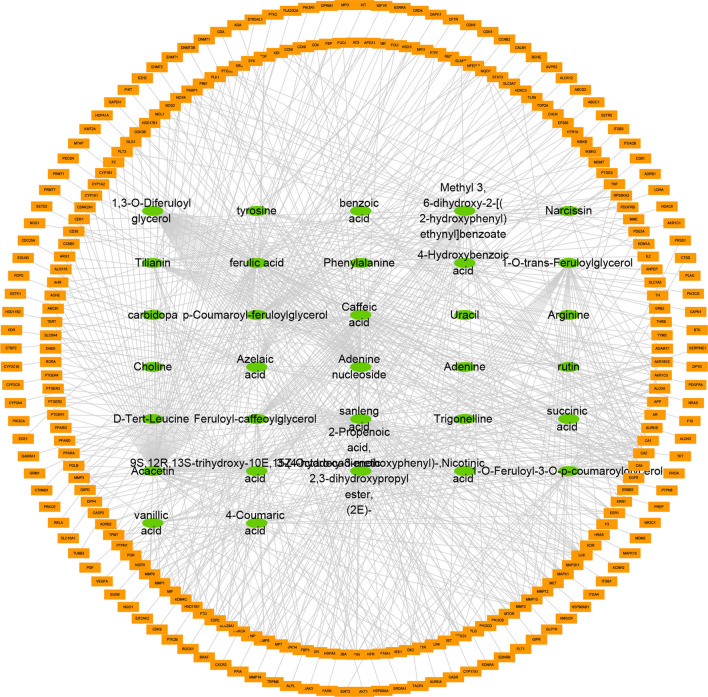
Compound-target network. Green elliptical nodes represent chemical components, and orange rectangular nodes represent targets.

We established a compound-target network with 262 GC target genes as anticancer targets (Fig. 3). There are 294 nodes and 685 edges in the network, among which the 32 green nodes represent the main components of SL, the 262 orange nodes represent the targets of GC, and the 685 edges represent the interactions between the components and the targets of GC. By observing the network, we found that the same active ingredient can act on multiple targets. The same target also corresponds to different chemical components, which fully reflect the multicomponent and multitarget characteristics of SL in GC treatment. According to the network topological parameters, the average values of the degree and betweenness centrality of compound nodes were 21.40625 and 0.076039366, respectively. We screened out compounds with a degree and betweenness centrality greater than the mean, such as acacetin, sanleng acid, ferulic acid, methyl 3.6-dihydroxy-2-[(2-hydroxyphenyl) ethynyl] benzoate, caffeic acid, adenine nucleoside, and azelaic acid, which may be the main active ingredients of SL in the treatment of GC.

#### PPI network analysis

**Figure 4:**
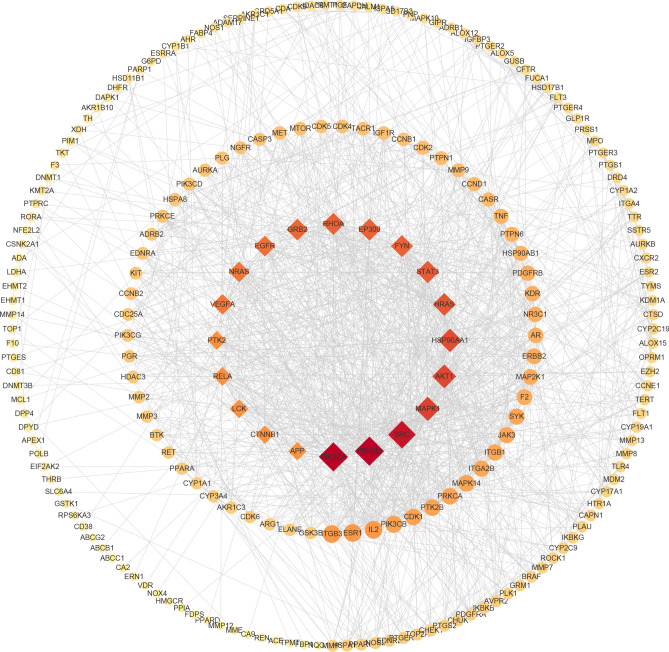
Protein–protein interaction (PPI) network.

The PPI network reveals the potential connection between targets. After removing the free genes, the PPI network contained 222 nodes and 1205 edges, with an average node degree of 10.9 (Fig. 4). The size and colour of the node reflects the importance of the degree. The larger the degree, the more important the node is in the network, suggesting that it may be a key target of SL in GC treatment. To make the figure clearer, we used diamonds to highlight the top 20 genes in all nodes. According to the degree value, the top 10 genes were regarded as hub genes, including PIK3R1 (degree = 56), PIK3CA (degree = 56), SRC (degree = 52), MAPK1 (degree = 43), AKT1 (degree = 42), HSP90AA1 (degree = 41), HRAS (degree = 39), STAT3 (degree = 38), FYN (degree = 37), and RHOA (degree = 37).

#### GO analysis and KEGG pathway analysis

**Figure 5:**
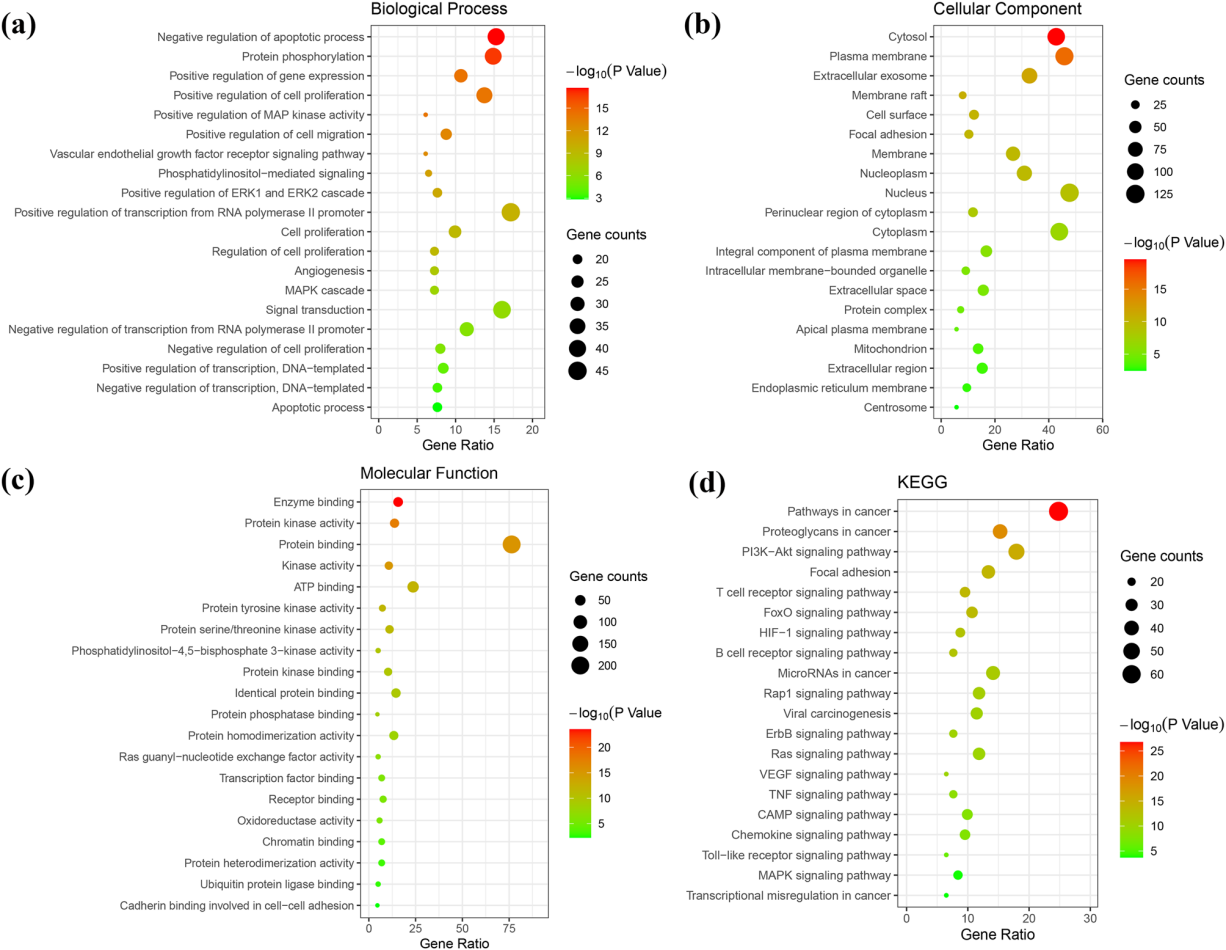
Bubble map of GO and KEGG pathway analyses. (**a**) Biological processes (BP) of GO terms. (**b**) Cellular components (CC) of GO terms. (**c**) Molecular functions (MF) of GO terms. (**d**) KEGG pathway analysis. Bubble size represents the number of enriched genes, and bubble colour difference represents the significance of target gene enrichment.

To elucidate the molecular mechanism underlying SL efficacy in the treatment of GC, we performed GO and KEGG pathway analyses on 262 anticancer targets (Supplementary Table S3). GO analysis identified 310 biological processes (BP), 50 cellular components (CC), and 93 molecular functions (MF). In BP, the targets mainly involve positive regulation of transcription from RNA, negative regulation of the apoptotic process, positive regulation of cell proliferation, and positive regulation of cell migration, angiogenesis, and the MAPK cascade. In CC, the targets mainly involve the nucleus, plasma membrane, cytoplasm, extracellular exosomes, integral components of the plasma membrane, and mitochondria. In MF, the targets mainly involve protein binding, ATP binding, enzyme binding, identical protein binding, protein kinase activity, and protein homodimerization activity. A total of 101 pathways were identified by KEGG pathway analysis, and the targets were closely related to pathways in cancer, PI3K-Akt signalling pathway, proteoglycans in cancer, microRNAs in cancer, focal adhesion, the Rap1 signalling pathway, the Ras signalling pathway, the cAMP signalling pathway, the HIF-1 signalling pathway, and the MAPK signalling pathway. This suggests that SL may play a role in the treatment of GC through the above pathways, among which the PI3K signalling pathway involves 47 potential targets, including most of the hub genes, and may be the key pathway. According to the number of enriched genes, the top 20 results in descending order of enrichment analysis were visualized, as shown in Fig. 5. The above results indicate that the biological processes involved in the anticancer targets of SL's main chemical components are diverse and distributed in different metabolic pathways, reflecting its multipathway characteristics.

#### Compound-target-pathway network analysis

**Figure 6:**
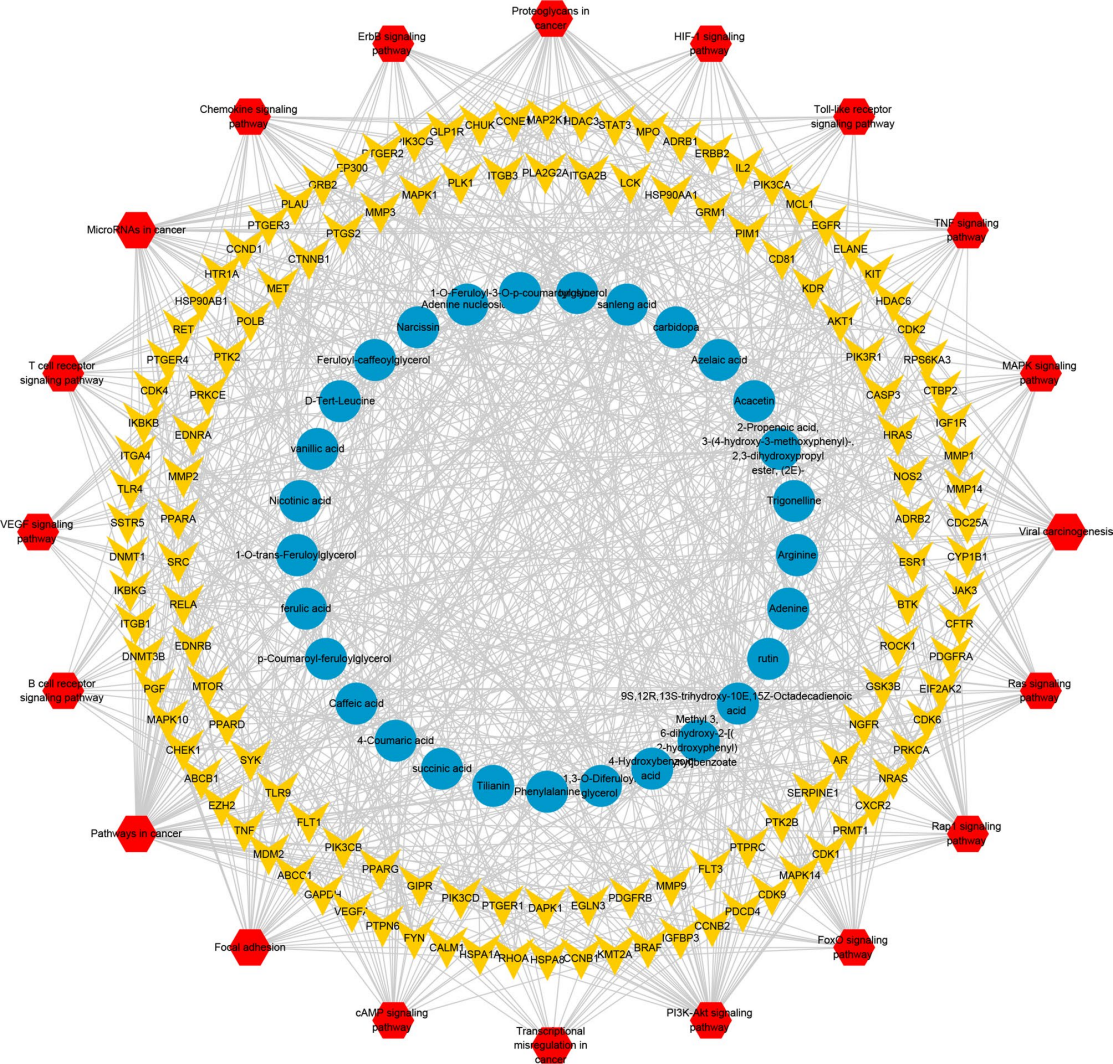
Compound-target-pathway network. Blue circular nodes represent chemical compounds, yellow V-shaped nodes represent targets, and red hexagonal nodes represent pathways.

A compound-target-pathway network was constructed with the targets included in the top 20 pathways and the chemical components corresponding to the targets obtained from KEGG pathway analysis (Fig. 6). The network contained 181 nodes with 29 representative components, 132 representative targets, 20 representative pathways, and 886 edges. From the diagram of the compound-target-pathway network, we can see intuitively that the targets of SL active components are distributed in different pathways, coordinate with each other, and play a common role in the treatment of GC, which comprehensively embodies the multicomponent, multitarget, and multipathway characteristics of traditional Chinese medicine.

#### Molecular docking analysis

**Figure 7:**
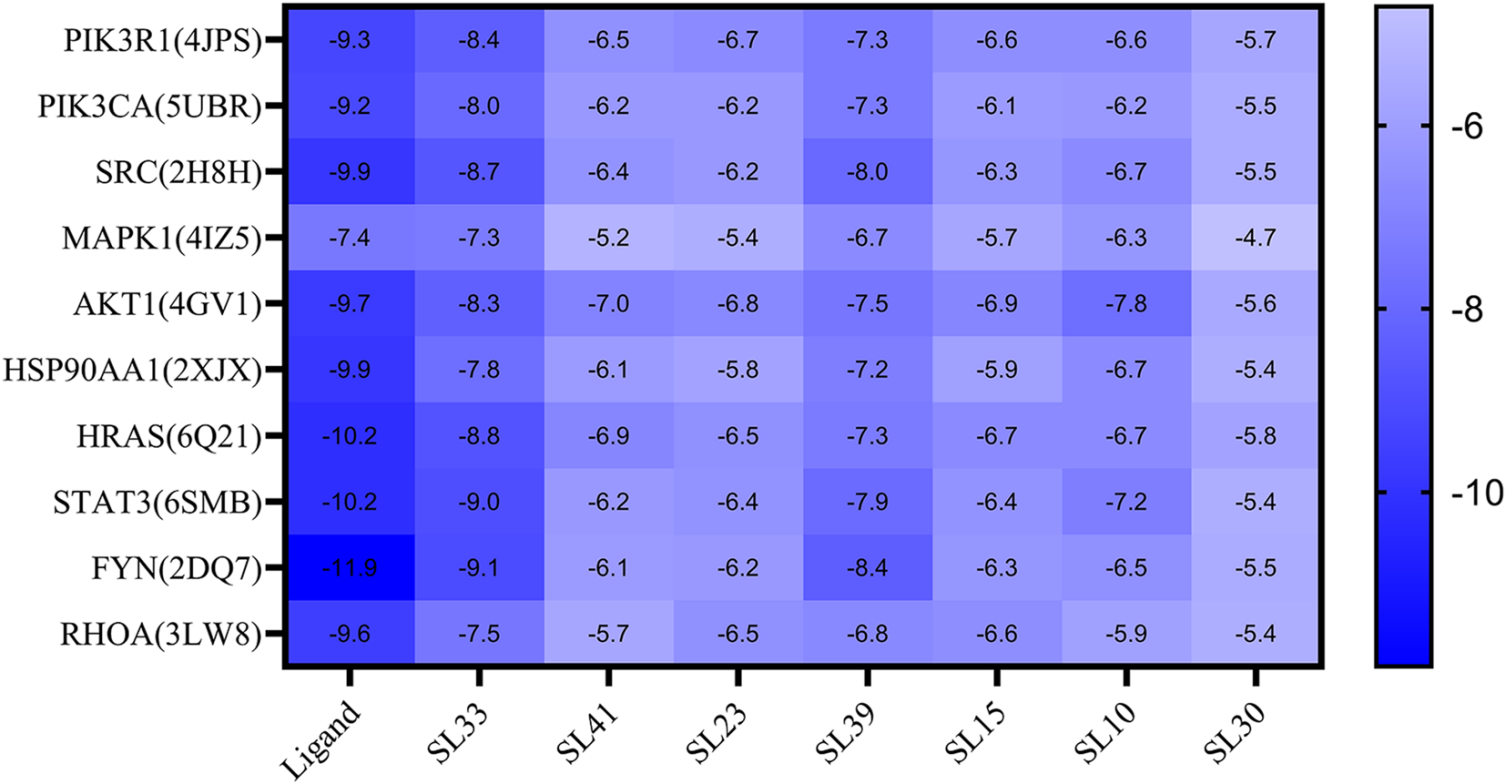
Heat map of molecular docking scores (kcal/mol^−1^). Ligand represents the original ligand of the protein. SL33, SL41, SL23, SL39, SL15, SL10, and SL30 are acacetin, sanleng acid, ferulic acid, methyl 3,6-dihydroxy-2-[(2-hydroxyphenyl) ethynyl]benzoate, caffeic acid, adenine nucleoside, and azelaic acid, respectively.

**Figure 8:**
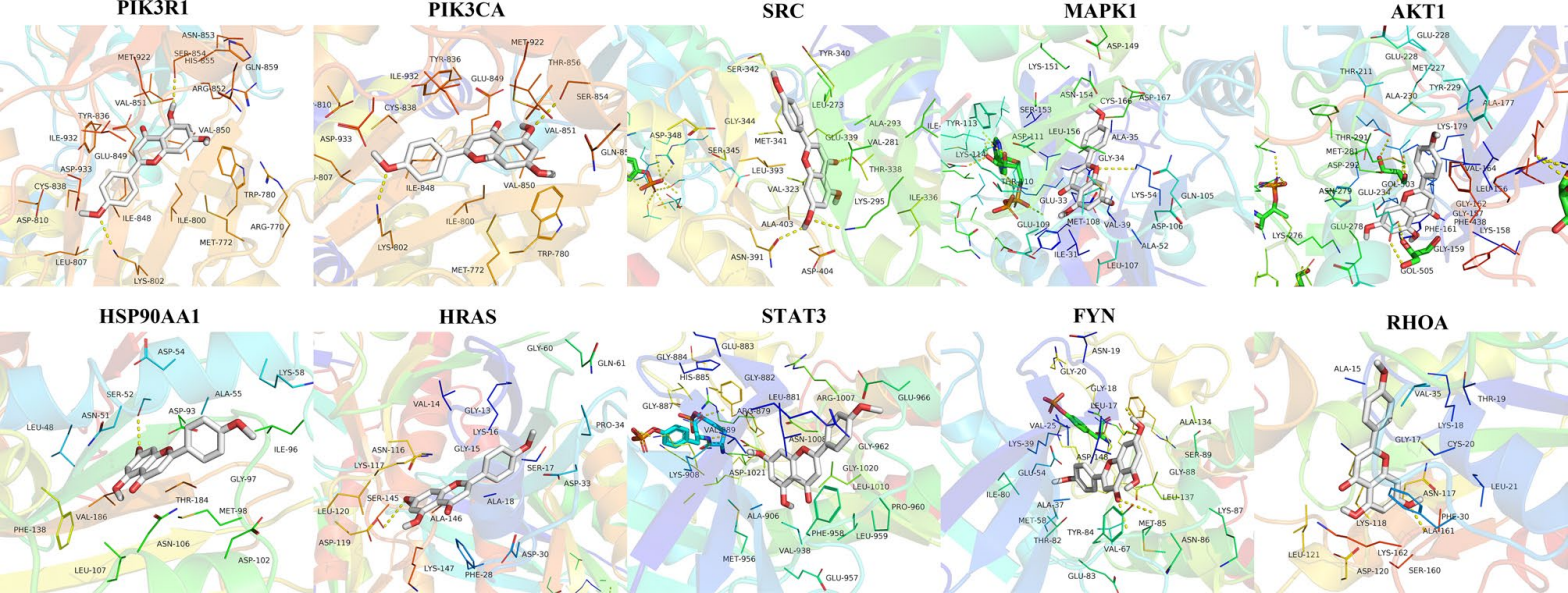
Schematic diagram of docking results. The docking results of acacetin with 10 core target proteins are shown.

We performed molecular docking analysis on seven major active ingredients with node degree and betweenness centrality greater than the average in the compound-target network and core targets with the top ten degrees in the PPI network. Moreover, the original ligands of potential protein targets were analysed. After docking with AutoDock Vina, the obtained data were analysed by a heat map, as shown in Fig. 7. It is generally believed that the lower the energy when the conformation of the ligand binding to the receptor is stable, the greater the possibility of action. In this study, almost all active ingredients and core target proteins' binding energies were less than − 5.0, which indicated that SL active ingredients had better binding activity with core targets, which stated that SL active ingredients had better binding activity with core targets. We selected the docking results of the compound (acacetin) that binds best to the target protein for display (Fig. 8).

### External validation of hub genes

#### mRNA expression levels of hub gens

**Figure 9:**
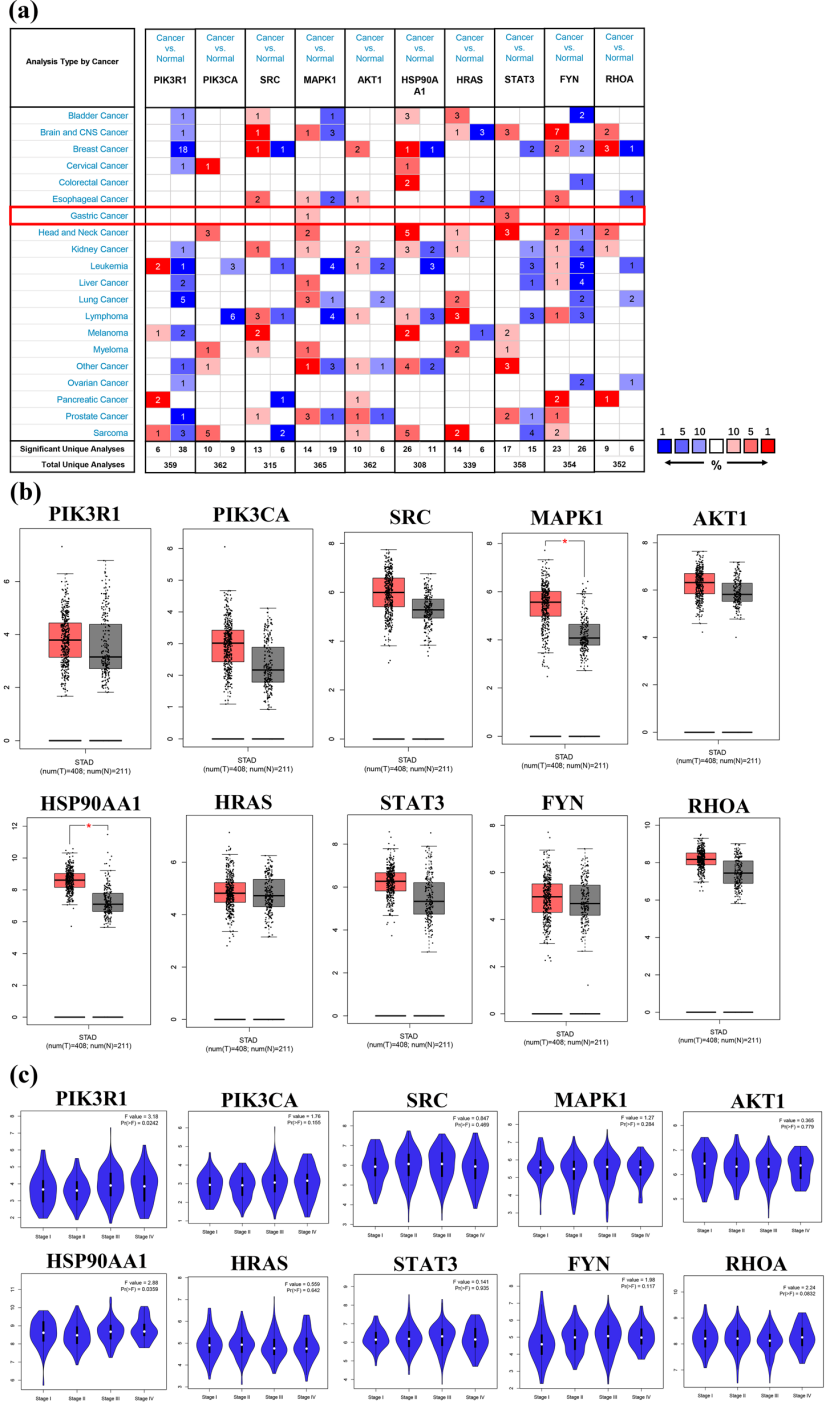
The mRNA expression levels of hub genes in different databases. (**a**) Oncomine analysis of hub gene mRNA expression levels in different cancers. Compared with normal tissues, the red box indicates the overexpression of the target gene in tumour tissues, while the blue box indicates the downregulation of the gene. The intensity of expression is expressed in shades of colour. (**b**) Boxplot of hub gene mRNA expression levels in the GEPIA database. Red represents GC tissue, and grey represents normal gastric tissue. (**c**) Stage plot of hub gene mRNA expression level and pathological stage in the GEPIA database.

We used the Oncomine database to analyse the differential expression of hub genes between GC tissues and normal tissues. The following thresholds were set: p-value: 0.01; fold change: 2; gene rank: Top 10%; data type: mRNA. The analysis results showed that the mRNA expression of MAPK1 and STAT3 was significantly upregulated in GC tissues, and there were no significant differences between GC and normal gastric tissues for other mRNA levels (Fig. 9a). Subsequently, further validation with the GEPIA database showed that the mRNA levels of MAPK1 and HSP90AA1 were significantly upregulated in GC specimens compared with normal gastric specimens (P < 0.01) (Fig. 9b). In addition, we analysed the relationship between hub gene mRNA levels and the pathological stage of GC. The results showed that the levels of PIK3R1 and HSP90AA1 changed significantly with pathological stage and increased significantly in stage III (Fig. 9c). These results suggested that the expression levels of these two genes might be correlated with GC progression.

#### Protein expression levels of hub gens

**Figure 10:**
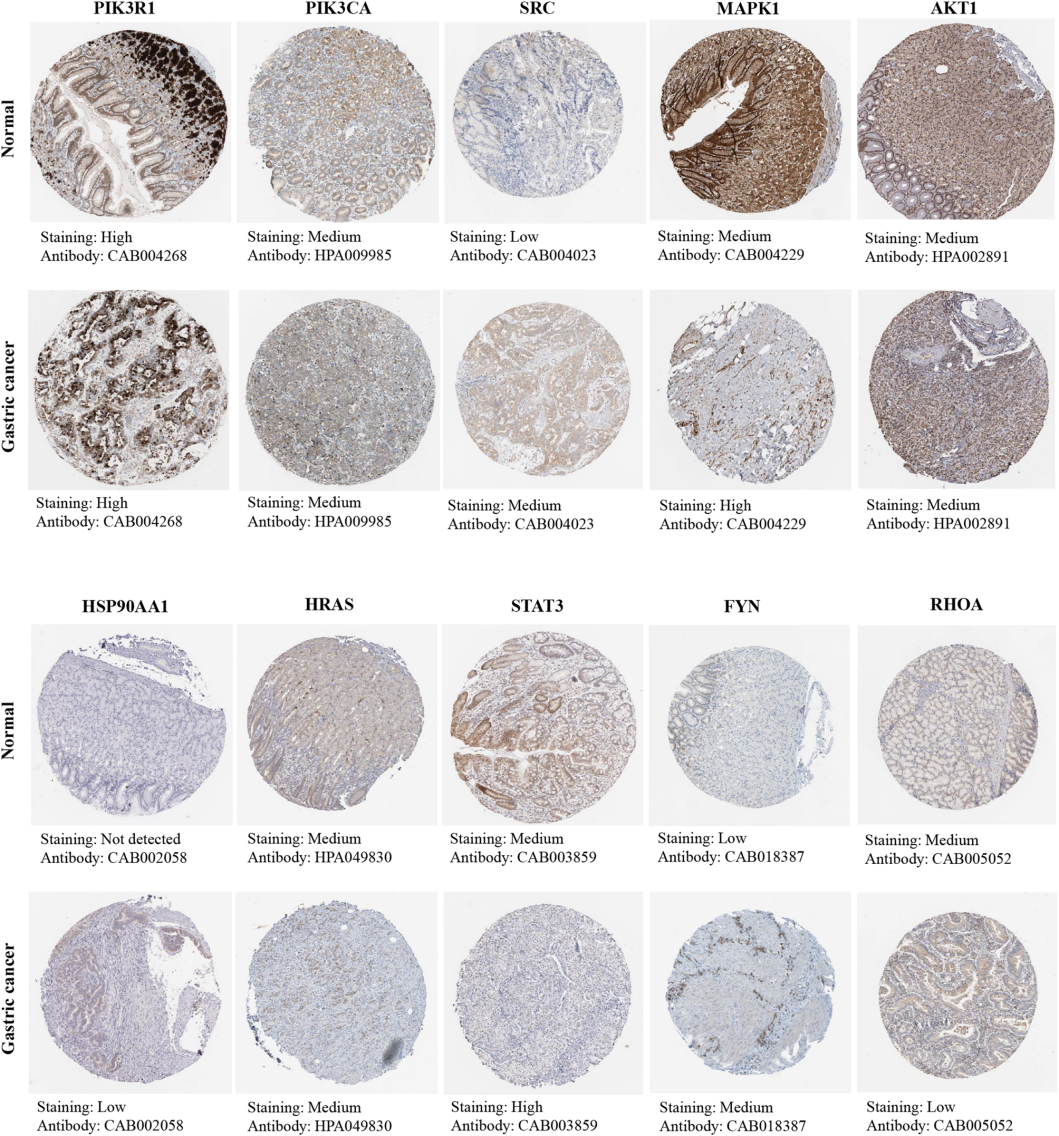
Immunohistochemical images of hub gene protein expression levels in the HPA database.

Additionally, we analysed the immunohistochemical staining images in the HPA database to observe the expression levels of hub gene proteins in GC. The results showed that except for HSP90AA1, the other nine hub genes were expressed to different degrees in normal gastric tissues. Compared with normal gastric tissues, the expression levels of SRC, MAPK1, HSP90AA1, STAT3, and FYN were increased in GC tissues, while the expression of RHOA was decreased in GC tissues (Fig. 10).

## Discussion

In this study, HPLC-Q-TOF–MS/MS technology was used to rapidly and comprehensively analyse the chemical components of SL, and 41 compounds were identified. Then, the identified compounds were studied in network pharmacology. Finally, we found seven main active ingredients in the drug, including acacetin, sanleng acid, ferulic acid, methyl 3.6-dihydroxy-2-[(2-hydroxyphenyl) ethynyl] benzoate, caffeic acid, adenine nucleoside, and azelaic acid; moreover, we identified PIK3R1, PIK3CA, SRC, MAPK1, AKT1, HSP90AA1, HRAS, STAT3, FYN, and RHOA as hub genes. Molecular docking showed that the active ingredients had good affinity for the hub gene proteins. These seven active ingredients may be the material basis for SL to exert therapeutic efficacy for GC. Modern pharmacological studies have shown that acacetin, as a natural flavonoid, can resist tumours in multiple links, pathways, and targets and is effective in most tumour cell lines. It can inhibit the proliferation of tumour cells, induce the autophagy and apoptosis of tumour cells, inhibit the invasion and migration of tumour cells and angiogenesis, regulate immunity, and reverse multidrug resistance^31^. Ferulic acid and caffeic acid are phenylpropanoids, and their antioxidant properties have been extensively demonstrated. Studies have shown that ferulic acid and caffeic acid can significantly inhibit COX-1 and COX-2 enzyme activities and inhibit tumour cell proliferation^32,33^. Ferulic acid can also induce the apoptosis of GC cells by upregulating the tumour suppressor transcription factor p53 and downregulating the mRNA and protein expression levels of the apoptosis inhibitory proteins Survivin and XIAP^34,35^. Caffeic acid can also cause apoptosis of SCM1 human GC cells^36^. Sanleng acid and azelaic acid are organic acid compounds. Sanleng acid is the earliest organic acid component identified by SL analysis, but no specific action mechanism has been reported yet. Azelaic acid can destroy mitochondrial respiration and inhibit cell synthesis; thus, it has good antiproliferation and cytotoxic effects on various cultured tumour cell lines and can be used as a potential anticancer drug^37^. Although methyl 3,6-dihydroxy-2-[(2-hydroxyphenyl) ethynyl] benzoate and adenine nucleoside are the main active ingredients in GC treatment screened by us, there is no clear report on the antitumour effect at present, which deserves further study to discover the potential mechanism of action.

An increasing number of studies have shown that TCM is a multitarget drug. Among the ten hub genes identified in this study, PIK3R1 and PIK3CA were identified as PI3K/protein kinase B (Akt) signalling pathway regulators. Studies have shown that abnormal upregulation of PIK3R1 and PIK3CA expression enhances the catalytic activity of PI3K and then activates the PI3K-Akt signalling pathway, causing GC cells to overproliferate and increasing the migration and invasion abilities of GC cells^38–40^. The proto-oncogene c-SRC, a member of the SRC family of kinases (SFKs), is one of the earliest nonreceptor-dependent tyrosine protein kinases found to be closely related to human diseases^41^. Current studies have shown that SRC can promote tumour cell proliferation and tumour angiogenesis, inhibit apoptosis, participate in cancer cell adhesion and invasion, and coregulate tumour growth through the interaction of growth factor receptors and growth factors^42,43^. Mitogen-activated protein kinase 1 (MAPK1) has been confirmed as an essential oncogene in the progression of GC, and its level is elevated in GC tissues and cells, which can promote the proliferation, migration, and invasion of GC cells^44–46^. Heat shock protein 90 (HSP90) is overexpressed in many malignant tumours, and members of the HSP90 gene family are essential for cell cycle regulation, survival, and apoptosis. Studies have shown that the expression of HSP90AA1 is associated with poor prognosis in GC^47,48^. STAT3, a key transcription factor in tumorigenesis, focuses on multiple signalling pathways, such as cell proliferation, carcinogenesis, and apoptosis, which can promote the growth, proliferation, angiogenesis, metastasis, and immune response of tumour cells^49,50^. Similar to SRC, FYN is an SFK that is overexpressed in GC and is positively correlated with metastasis and may promote gastric cancer metastasis by activating STAT3-mediated epithelial-mesenchymal transition^51^. In addition, our study also showed that SRC, MAPK1, STAT3, HSP90AA1, PIK3R1, and FYN were overexpressed in GC patients, which may be associated with the poor prognosis of GC patients. The AKT1 signalling pathway plays a vital role in regulating the biological functions of tumour cell growth, proliferation, apoptosis, and metabolism. Its positive expression rate in GC tissues is significantly higher than that in adjacent tissues, and it participates in the occurrence and development of GC^52–54^. HRAS belongs to the RAS gene family, which regulates RAF-MEK-ERK, PI3K/AKT, and other signalling pathways related to cell survival and proliferation by binding to GTP/GDP and the RAS protein to act as a molecular switch^55,56^. HRAS mutations are closely associated with the occurrence of various tumours. The expression of RHOA, a RAS homologous family, is related to certain tumorigenesis; however, its prognostic value in GC remains controversial. Some studies have found that the RHOA signaling pathway plays a vital role in the occurrence, invasion, metastasis, immune escape, and multidrug resistance mechanisms of gastric cancer^57,58^. Nevertheless, some studies have shown that the overall prevalence of RHOA-mutant GC is low, usually offering a lower T stage and no distant metastasis^59^. Our external validation also showed that RHOA was expressed at a low protein level in GC tissues; therefore, further study of this gene is necessary.

To better understand the molecular mechanism of SL in the treatment of GC, we performed GO and KEGG pathway analyses on the targets. GO analysis results showed that the target genes were mainly related to biological processes such as positive regulation of transcription from RNA, negative regulation of the apoptotic process, positive regulation of cell proliferation, positive regulation of cell migration, angiogenesis, and similar processes. In CC, the nucleus accounted for the largest proportion. In MF, protein binding, ATP binding, and enzyme binding were the main components. KEGG pathway analysis showed that the signalling pathway of SL in the treatment of GC was most related to the PI3K-Akt signalling pathway. Additionally, it involved the Ras signalling pathway, the MAPK signalling pathway, and other signalling pathways. Most of the hub genes, such as HRAS, AKT1, HSP90AA1, PIK3CA, PIK3R1, MAPK1, and RHOA, play roles in these signalling pathways, which is consistent with the results of modern pharmacological studies.

In conclusion, the analytical method based on HPLC-Q-TOF–MS/MS technology in this study can accurately identify the chemical components in SL efficiently, rapidly, and comprehensively. Simultaneously, the network pharmacology method is used to deeply excavate its potential active ingredients and the mechanism of drug treatment for GC to provide more scientific theoretical guidance for the improvement of quality control standards and clinical application of SL in the future. In our study, we found that SL is a multitarget anticancer drug. We predicted that the primary mechanism of action of SL in the treatment of GC is as follows: mediating PI3K-Akt, Ras, MAPK, and other signaling pathways to regulate the proliferation, apoptosis, migration, and angiogenesis of tumour cells, thus playing a role in the treatment of GC. However, the above results still need further experimental verification.

## Methods

### HPLC-Q-TOF–MS/MS analysis

#### Instruments and materials

*Instruments* Ultimate 3000 High-Performance Liquid Chromatograph (1000 mm × 1000 mm × 1000 mm), SIL-20A XR UFLC (Shimadzu, Japan); Triple TOF 5600 System-MS/MS High-Resolution Triple Quadrupole Time of Flight Mass Spectrometer (AB SCEIX, USA); Electronic Balance (Tianjin Tianma Hengji Instrument Co., Ltd.); SHZ-D (III) Circulating Water Vacuum Pump (Nanjing Wenke Instrument and Equipment Co., Ltd.); KQ-500B Ultrasound Cleaner (Kunshan Ultrasound Instrument Co., Ltd.); PST-JY-10 Puri Phil pure water machine.

*Materials* Methanol (TEDIA, batch No. 18095056), formic acid (Jiangsu Qiangsheng Functional Chemical Co., Ltd., batch No. 20160412), and acetonitrile (Merch, batch No. 1.00030.4000). SL medicinal materials were obtained from the TCM pharmacy of Jiangsu Province Hospital of Chinese Medicine and were purchased from Ma'anshan Jingquan Traditional Chinese Medicine Decoction Pieces Co., Ltd. Origin: Zhejiang, Batch No. 200601, Standard Basis: *Pharmacopoeia of the People's Republic of China* (2015 Edition). It was identified by associate professor Ruilian Yu, School of Pharmacy, the Nanjing University of Chinese Medicine as Sparganiaceae plant *Sparganium stoloniferum* (Buch.-Ham. ex Graebn.) Buch.-Ham. ex Juz. tubers. The specimens were deposited in the Central Laboratory of Jiangsu Province Hospital of Chinese Medicine.

#### Preparation of the test solution of SL

The proper amount of SL medicinal materials was crushed and sieved through 60 mesh, and 1 g of powder was precisely weighed. Then, the weighed 1 g powder was soaked ten times in double-distilled water for 30 min, refluxed and extracted twice, the first for 30 min and the second for 20 min, combined with two filtrates, evaporated by a rotary evaporator at 70 ℃, and then reconstituted with absolute ethanol to a 10-ml volumetric flask.

#### Chromatographic and mass spectrometry conditions

*Chromatographic conditions* Hedera C18 column (250 mm × 4.6 mm, 5 μm); mobile phase: 0.1% formic acid water (B)—0.1% formic acid methanol (C), gradient elution (0–7 min, 97–97% B; 7–15 min, 97–50% B; 15–20 min, 50–10% B; 20–25 min, 10–97% B; 25–37 min, 97–97% B); flow rate: 1 mL/min, column temperature 30 ℃, injection volume 5 μL. The detection wavelength of DAD was 260 nm. *Mass spectrometry conditions* Electron spray ionization (ESI), using positive and negative ion mode scanning; mass scanning range m/z 50–1500; ion source temperature 550 ℃; air curtain gas flow rate 40 L/min; atomization airflow speed 55 L/min; auxiliary airflow speed 55 L/min; spray voltage + 5500 V/− 4500 V; decluster voltage ± 100 V. Data acquisition software: Analyst TF 1.6 software (AB SCEIX, USA); data processing software: Peakview 1.2 software (AB SCEIX, USA).

#### Identification of compounds

According to the multistage mass spectrum fragment information and the precise relative molecular mass provided by high-resolution mass spectrometry, the molecular formula was fitted by Peakview 1.2 software with a mass deviation range (δ) ≤ 5 × 10^–6^, and the compounds were preliminarily predicted. Then, it was further confirmed by comparing the retention time and the mass spectrum fragment information provided by the SciFinder database and related references to achieve the purpose of the accurate identification of compounds.

### Network pharmacology research

#### Prediction of potential targets of compounds and collection of disease targets

SwissTargetPrediction (http://www.swisstargetprediction.ch/)^60^ is a network tool for ligand-based target prediction of any small biologically active molecule. We transformed the compounds identified by mass spectrometry into canonical SMILES through the PubChem (https://pubchem.ncbi.nlm.nih.gov/), Chemical Book (https://www.chemicalbook.com/ProductIndex.aspx), and ChemSpider (http://www.chemspider.com/) databases. We then imported SMILES into SwissTargetPrediction to predict all potential targets of compounds. Species were selected as “*Homo sapiens*” with probability > 0 as the screening condition.

Using “Gastric Cancer” as the keyword, the human gene database (GeneCards, https://www.genecards.org/)^61^, the Online Mendelian Inheritance in Man (OMIM, https://omim.org/)^62^, DisGeNET (Version 7.0) (https://www.disgenet.org/)^63^ and the Therapeutic Target Database (TTD, http://bid.nus.edu.sg/group/cjttd/)^64^ were used to collect relevant targets of GC. In this study, “score” ≥ mean value was used as the criterion for screening disease target genes.

Then, the predicted targets of the chemical components of SL were mapped with the targets of GC, and the intersection of the two was taken to obtain the target set of SL for the treatment of GC.

#### Construction of compound-target network

The chemical components of SL and its therapeutic targets in GC were introduced into Cytoscape (Version 3.8.0) (https://cytoscape.org/)^65^ to construct the compound-target network. The “network analysis” is used to analyse the topological parameters of the network, where the “degree” represents the number of nodes connected with this node in the network; the greater the degree of the node is, the more critical it is in the network. The “betweenness centrality” reflects the importance of a node in transmitting information through the network, and the greater the betweenness centrality of the node is, the more critical it is in the network. The core network was screened based on the network node topological parameters “degree” and “betweenness centrality” to obtain the main active ingredients of SL for the treatment of GC.

#### Construction of PPI network

The targets of SL for the treatment of GC were imported into the STRING Database (Version 11.0) (https://string-db.org/)^66^, and the correlation between target proteins was analysed. “Organism” was set as “*Homo sapiens*”. The PPI network was constructed with a “combined score” ≥ 0.9 as the screening condition. The visualization process was carried out with Cytoscape (Version 3.8.0), and targets with a high degree of connectivity were selected as hub genes.

#### Gene function annotation and construction of the compound-target-pathway network

The Database for Annotation, Visualization and Integrated Discovery (DAVID) (Version 6.8) (https://david.ncifcrf.gov/)^67,68^ provides systematic and comprehensive biological function annotation information for a large number of genes. It can identify the most significantly enriched biological annotations. We introduced the target set of SL for GC treatment into DAVID (Version 6.8) and defined the species as “*Homo sapiens*” for Gene Ontology (GO) and Kyoto Encyclopedia of Genes and Genomes (KEGG) pathway analyses. To more comprehensively annotate the biological functions of genes to better understand the molecular mechanism of SL in treating GC, GO will describe the nature of genes from three terms, including cell component (generally used to describe the location of gene action), molecular function (which can describe the activity at the molecular level) and biological process. P < 0.01 was used as a screening condition. Enrichment analysis bubble maps were plotted using the R language.

Based on the results of KEGG pathway analysis, pathways related to GC and the top 20 enriched genes were identified. Then, Cytoscape (version 3.8.0) was used to further construct the compound-target-pathway network.

#### Molecular docking between active ingredients and hub genes

To further validate the reliability of the target prediction results, molecular docking was performed on the selected active ingredients and hub genes. Active ingredients were loaded in the SDF format file of their 3D structure through the PubChem database and were then imported into Chem3D for optimization and saved in mol2 format; hub genes were kept in the Research Collaboratory for Structural Bioinformatics Protein Data Bank (RCSB PDB, https://www.rcsb.org/)^69,70^, where the best protein crystal structure was selected (human protein, with ligands, relatively complete structure, smaller resolution value), and its PDB format file was downloaded. Before docking, the original crystal ligand and water molecule in the protein–ligand complex were removed using PyMol^71^. The protein and ingredients were then hydrogenated, charged, and subjected to other operations using AutoDockTools and converted into PDBQT format files. Auto Dock Vina^72^ was used to perform molecular docking between the processed ingredients and protein, and the docking results were visualized using PyMol software.

### External validation of hub genes

#### Analysis of mRNA expression level

Oncomine 4.5 (https://www.Oncomine.org)^73^ is a cancer gene expression profile database and integrated data-mining platform designed to facilitate the discovery of genome-wide expression analysis. Through the Oncomine database, we compared the differential expression of hub genes in GC tissues and normal gastric tissues.

Gene Expression Profiling Interactive Analysis (GEPIA, http://gepia.cancer-pku.cn/index.html)^74^ is a newly developed interactive web server for analysing the RNA sequencing expression data of 9736 tumours and 8587 normal samples from the TCGA and GTEx projects using a standard processing pipeline. The GEPIA database can further verify the differential expression of hub genes between GC and normal gastric tissues, and it can also analyse them according to pathological stages.

#### Analysis of protein expression level

The Human Protein Atlas (Version 19.3) (HPA, https://www.proteinatlas.org/)^75^ database is mainly an extensive proteome database based on immunohistochemical analysis. The protein expression levels of hub genes in GC tissues and normal gastric tissues were compared according to the staining intensity and percentage of stained cells in the tissues, and representative immunohistochemical staining pictures were obtained.

## Supplementary Information

Supplementary Figures.

Supplementary Tables.

## Data Availability

All data generated or analysed during this study are included in this published article and its “Supplementary Information” files.
