# Supplementary material for: Study of the active ingredients and mechanism of Sparganii rhizoma in gastric cancer based on HPLC-Q-TOF–MS/MS and network pharmacology
Source: Sci Rep. 2021 Jan 21;11:1905. doi: 10.1038/s41598-021-81485-0 (PMC7820434; doi:10.1038/s41598-021-81485-0)
Supplement: Supplementary file 1 — Supplementary Figures. [file 41598_2021_81485_MOESM1_ESM.docx]

Study of the Active Ingredients and Mechanism of *Sparganii rhizoma* in Gastric Cancer Based on HPLC-Q-TOF-MS/MS and Network Pharmacology

Xiaona Lu^1,2†^, Yawei Zheng^2†^, Fang Wen^2^, Wenjie Huang^2^, Xiaoxue Chen^2^, Shuai Ruan^2^, Suping Gu, Yue Hu^1,2^, Yuhao Teng^1,2^ and Peng Shu^1,2*^

^1^ Oncology Department, Affiliated Hospital of Nanjing University of Chinese Medicine, Nanjing, China.

^2^ First School of Clinical Medicine, Nanjing University of Chinese Medicine, Nanjing, China.

**†**these authors contributed equally to this work

*****corresponding.shupengsp@njucm.edu.cn

**Supplementary Figures.** Secondary mass spectra of each compound.


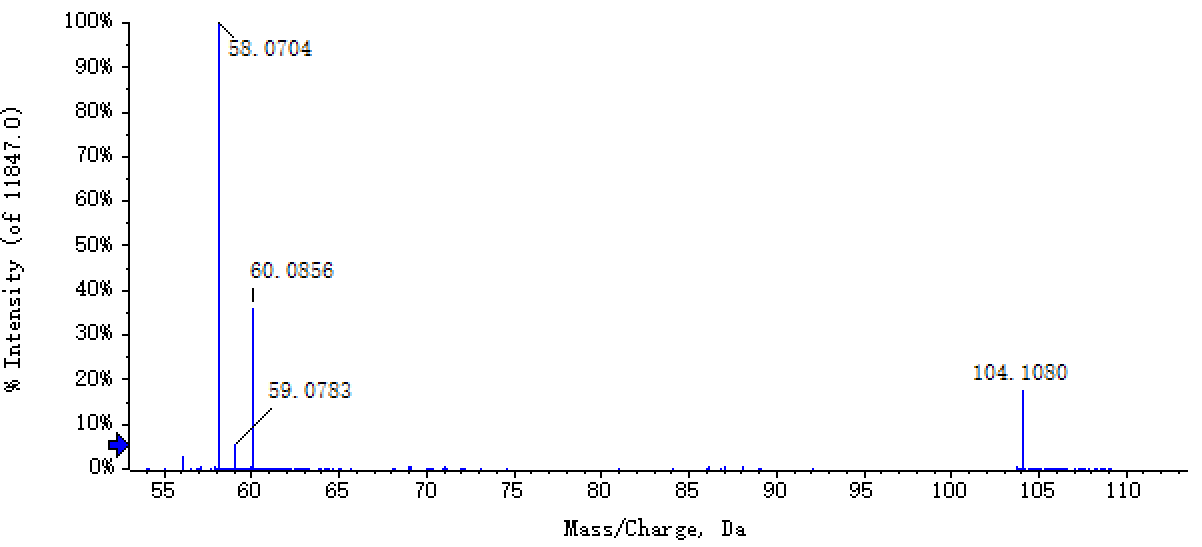


SL1: Choline


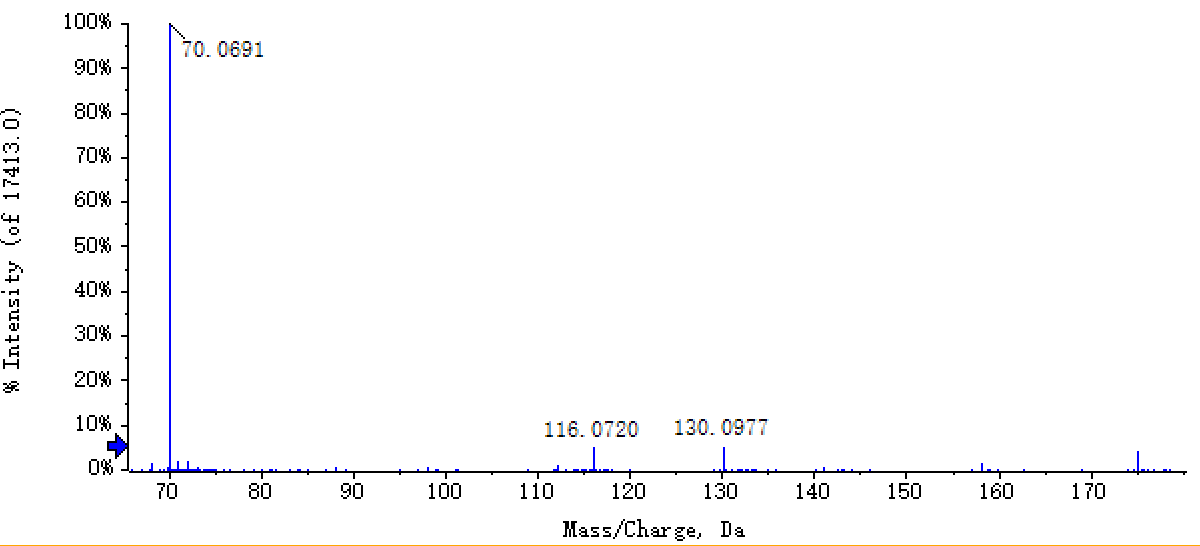


SL2: Arginine


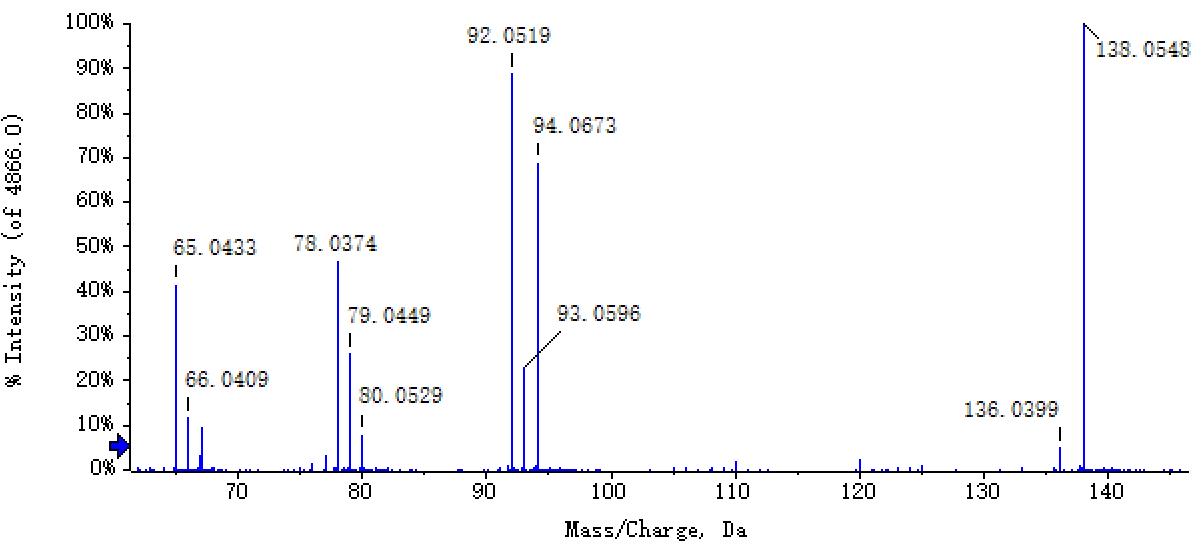


SL3: Trigonelline


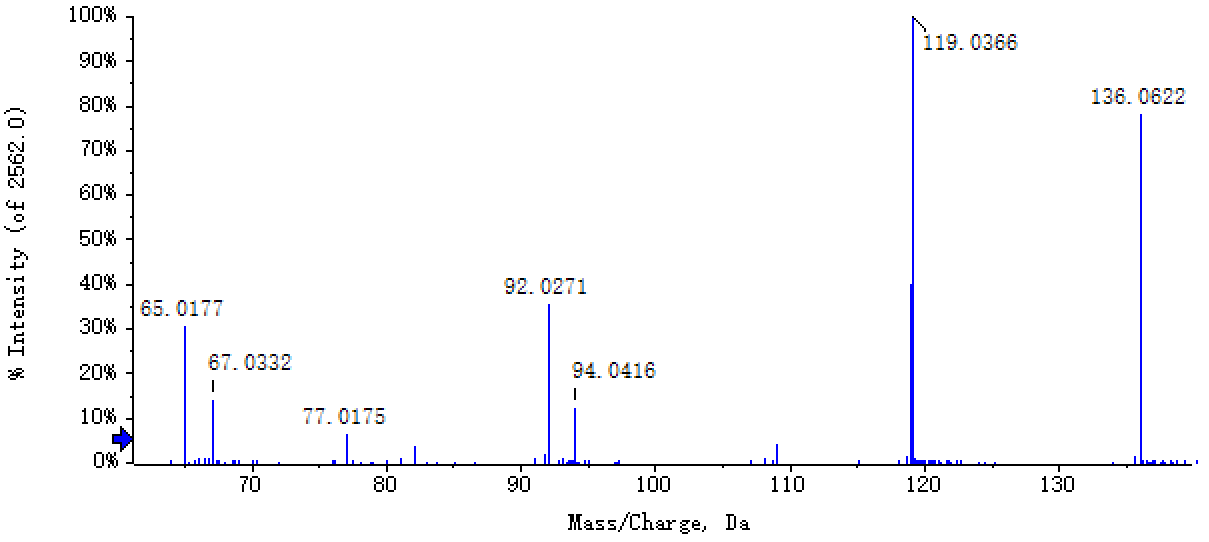


SL4: Adenine


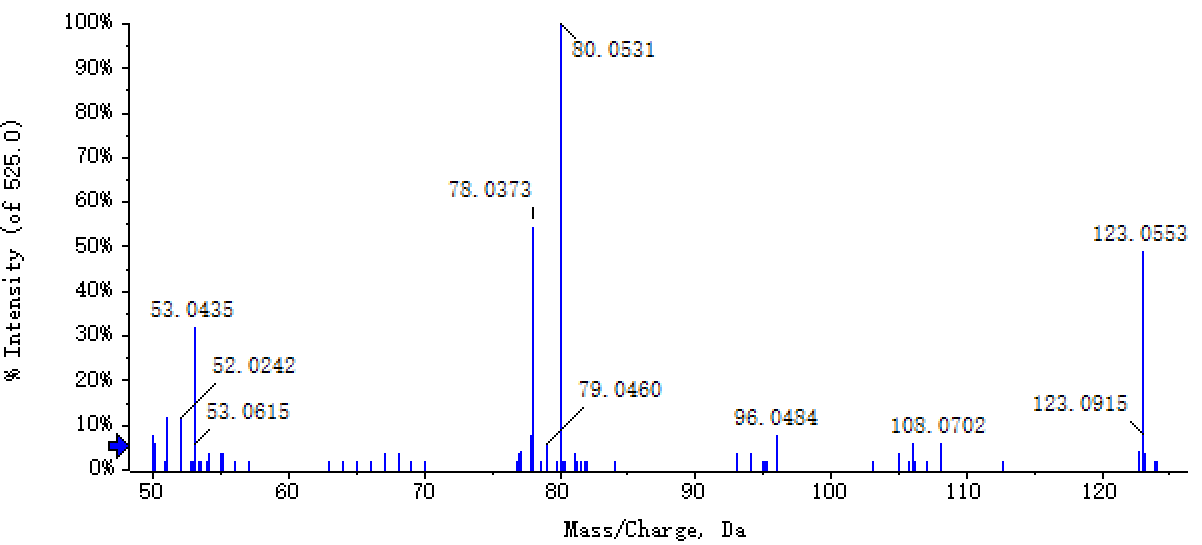


SL5: Nicotinic acid


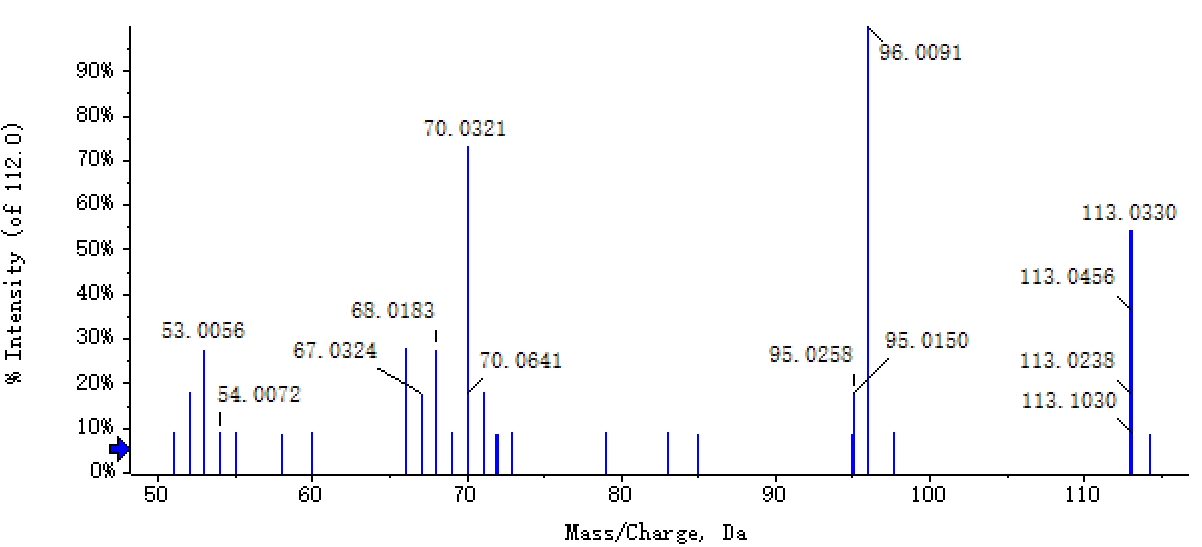


SL6: Uracil


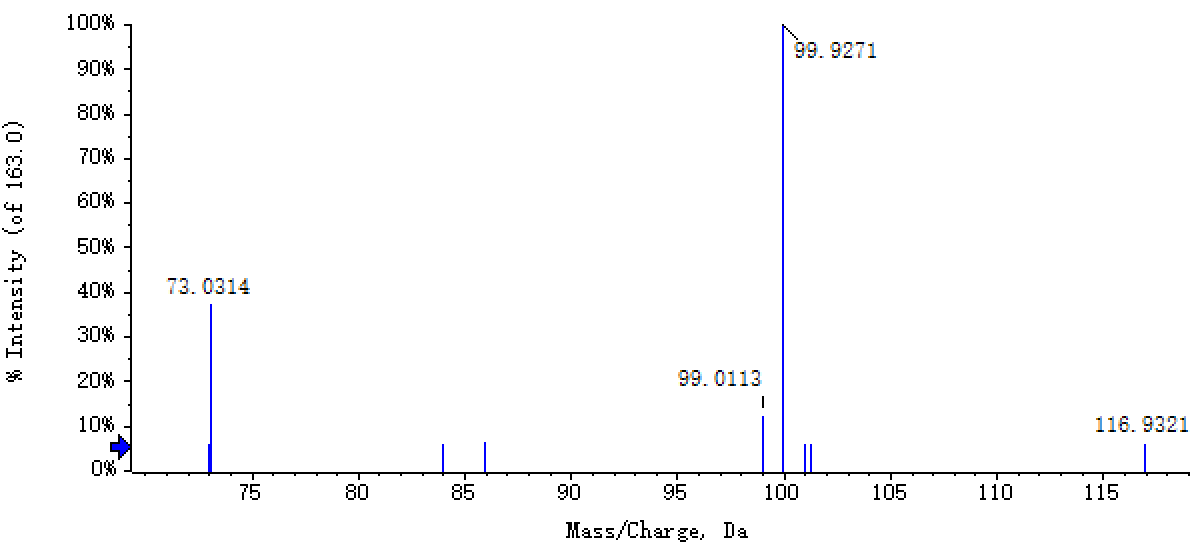


SL7: succinic acid


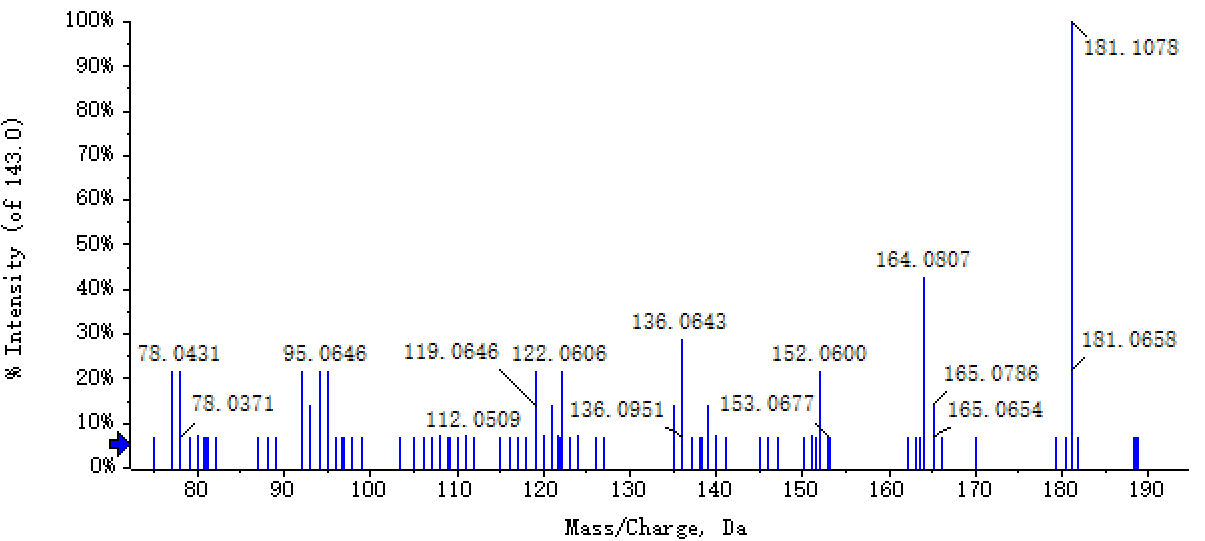


SL8: tyrosine


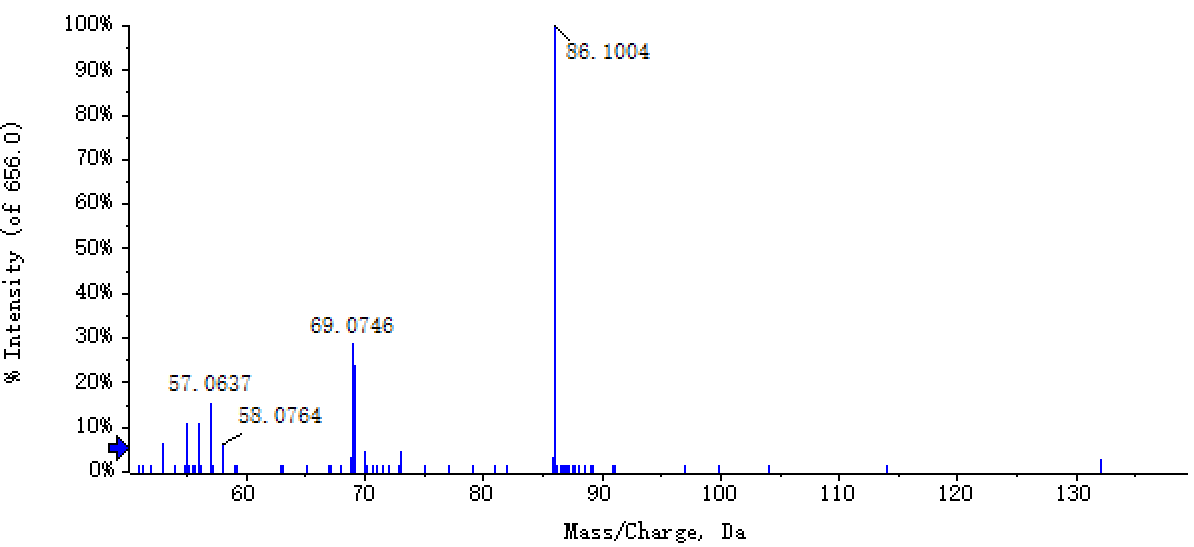


SL9: D-Tert-Leucine


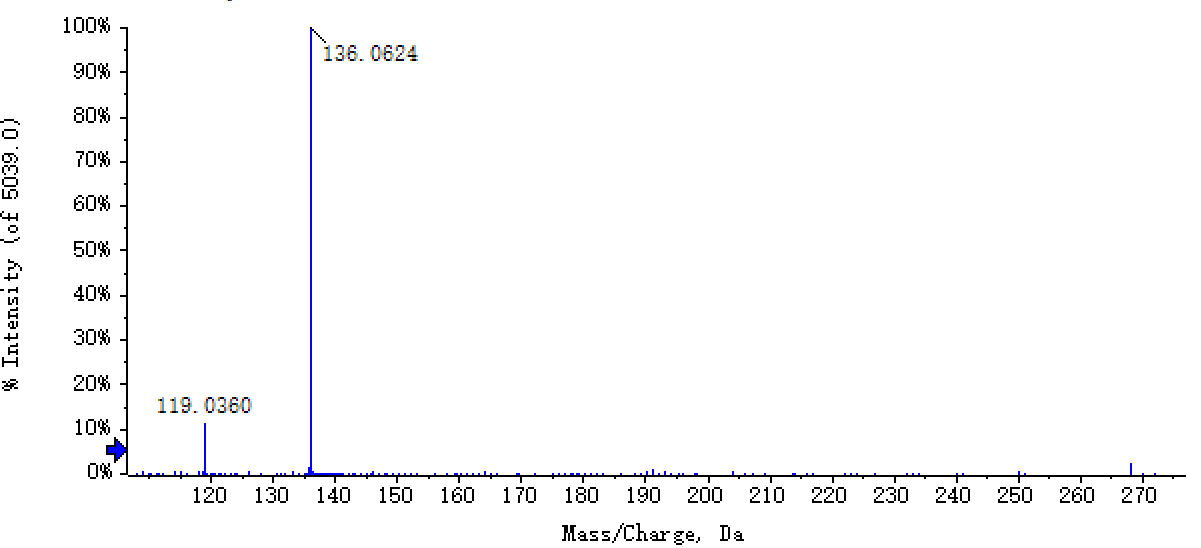


SL10: Adenine nucleoside


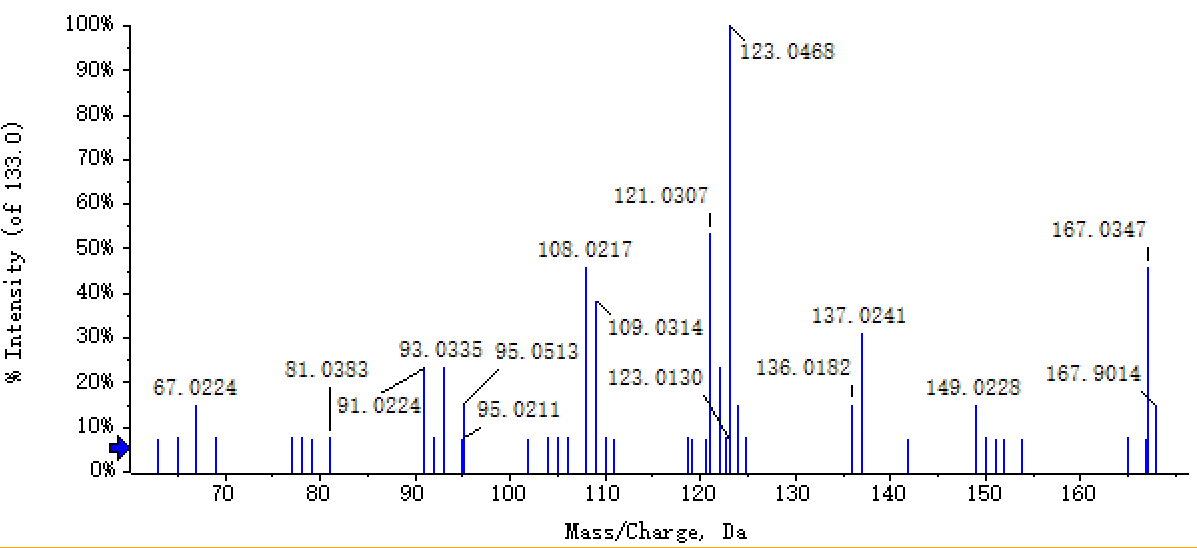


SL11: vanillic acid


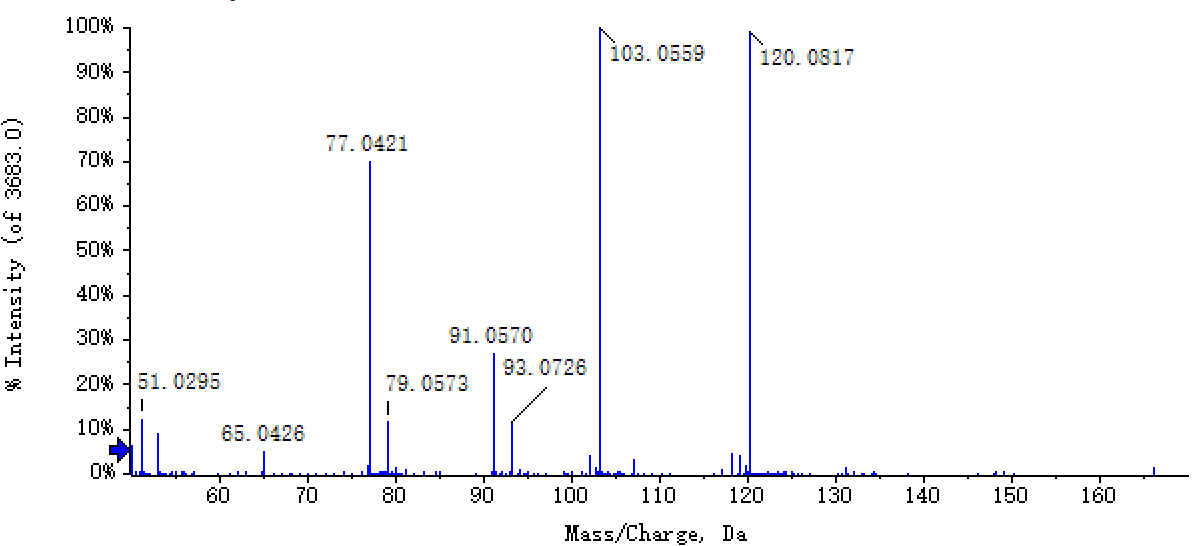


SL12: Phenylalanine


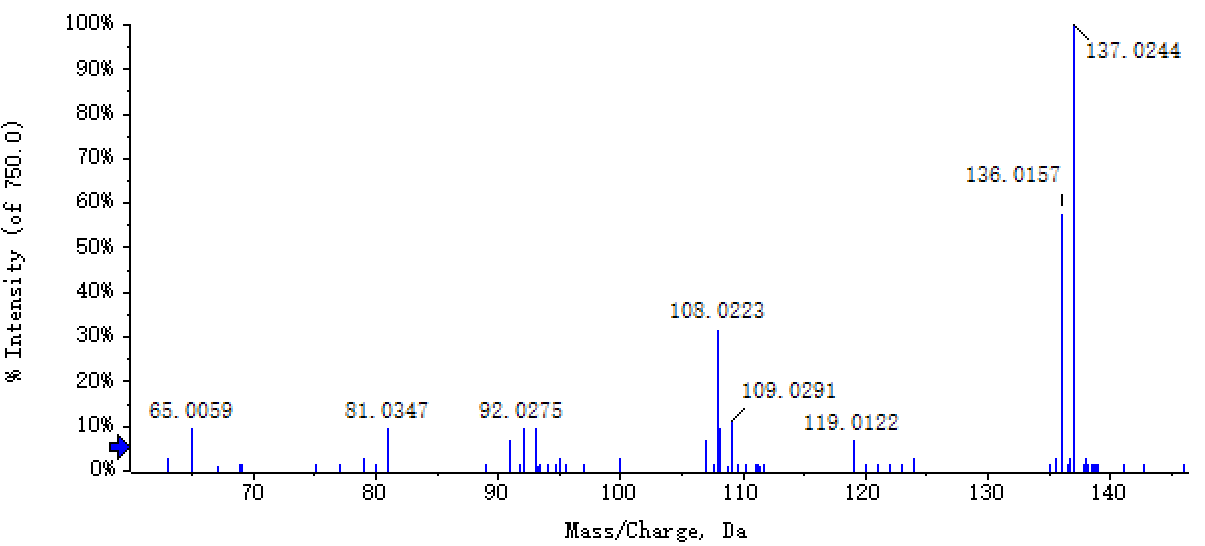


SL13: 4-Hydroxybenzoic acid


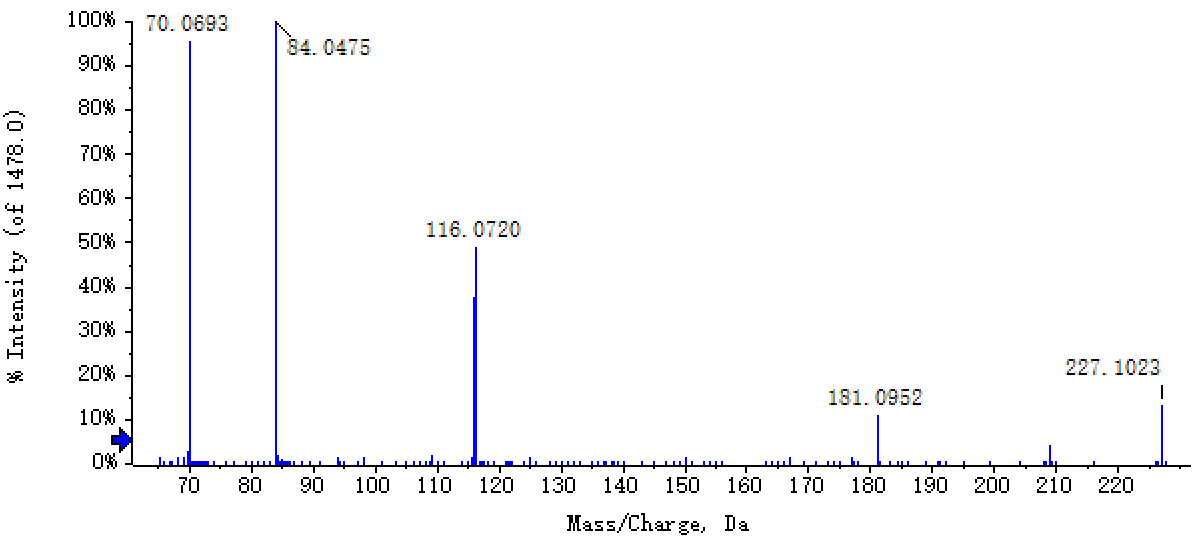


SL14: carbidopa


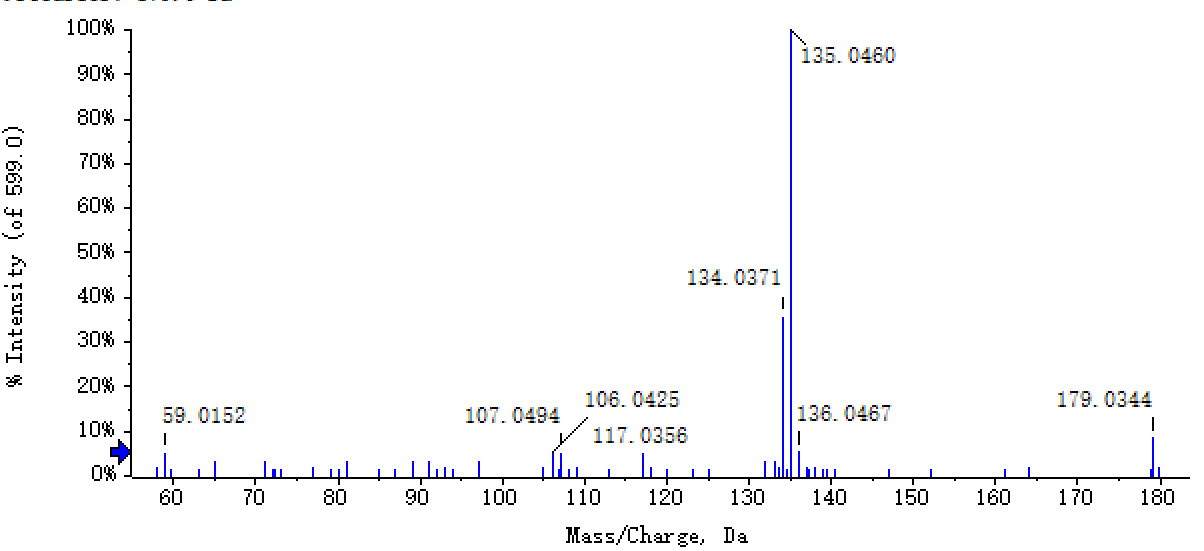


SL15: Caffeic acid


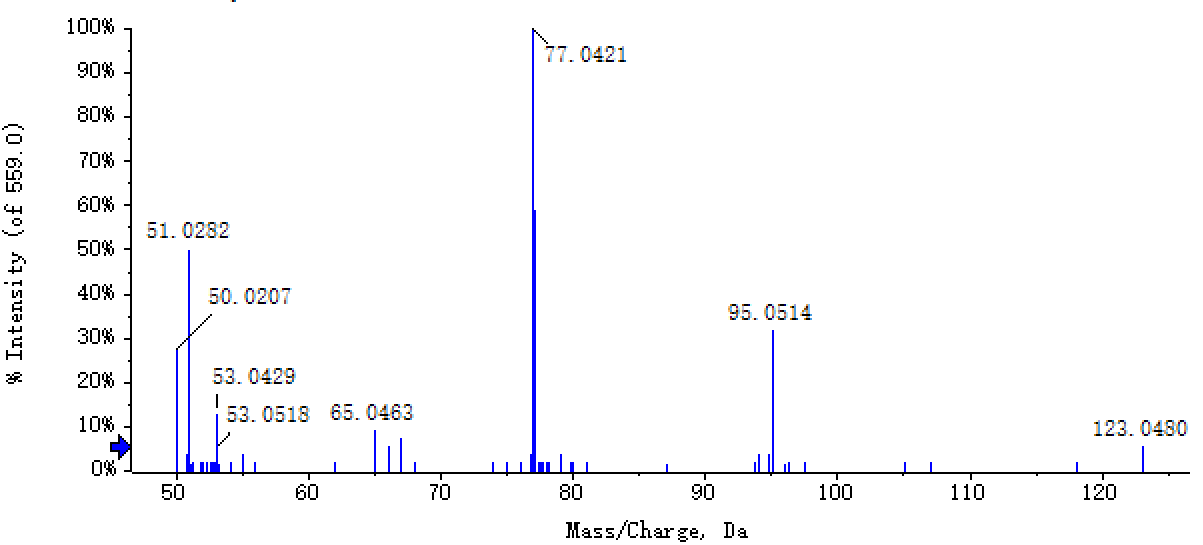


SL16: benzoic acid


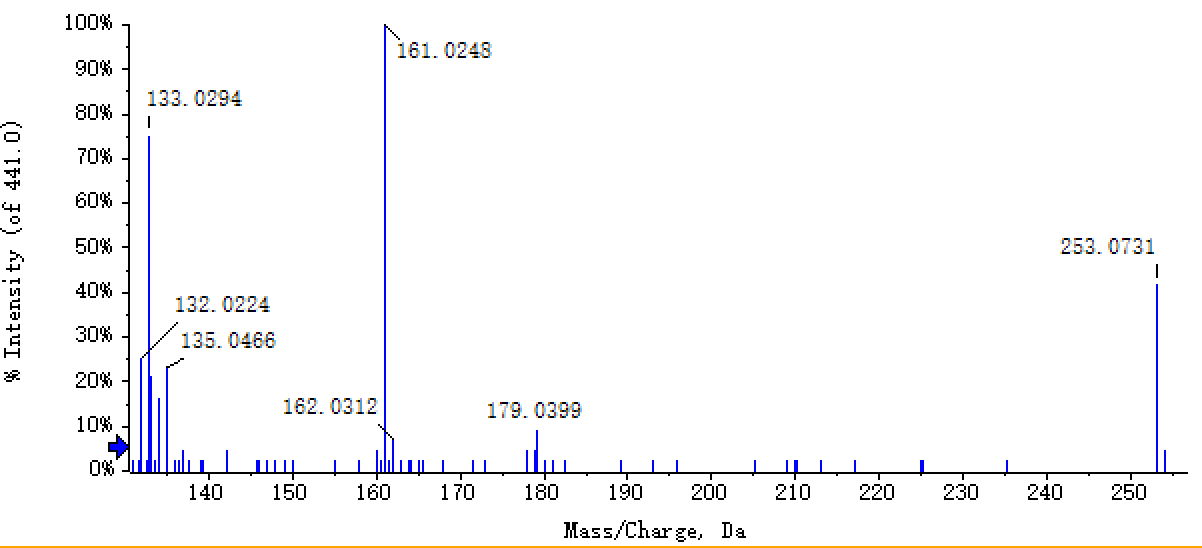


SL17: hwanggeumchal B


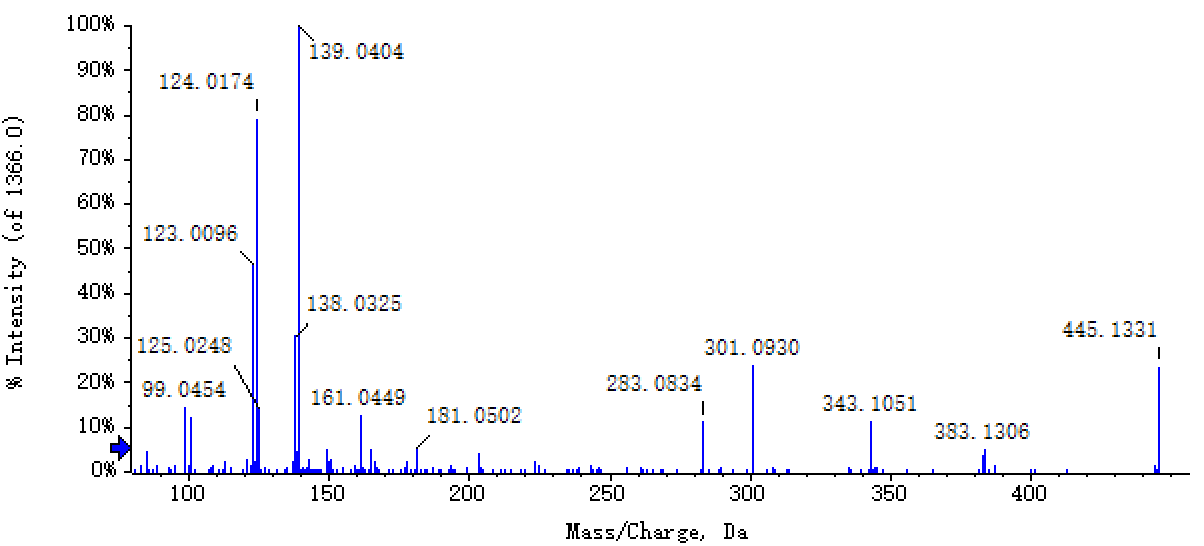


SL18: 4-hydroxy-2-methoxyphenyl 1-O-[6-(hydrogen 3-hydroxy-3- methylpentanedioate)]-β-D-glucopyranoside


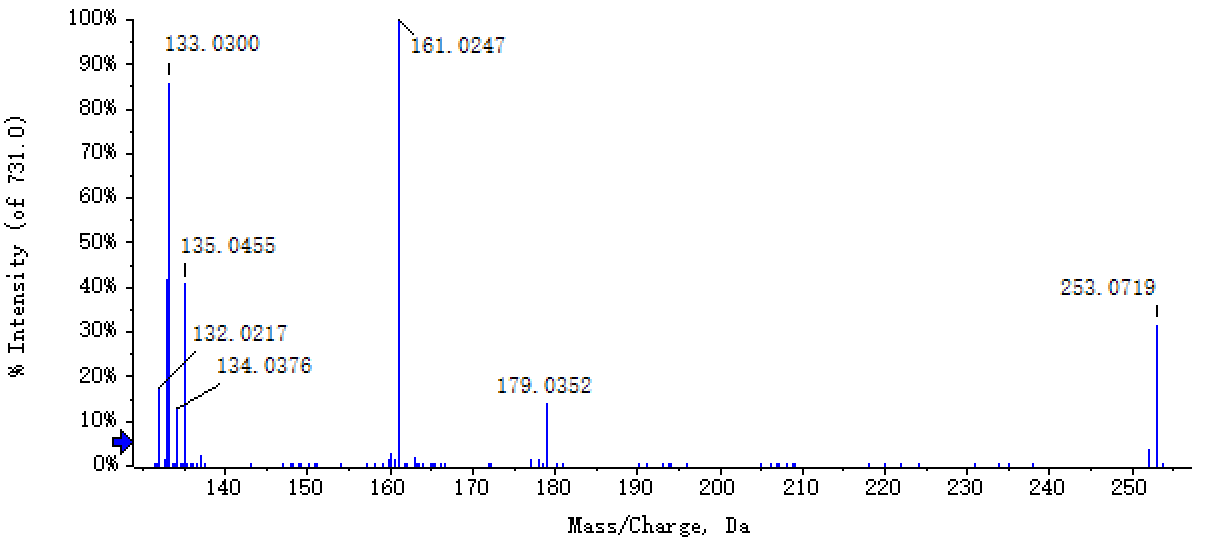


SL19: 1-Caffeoylglycerol


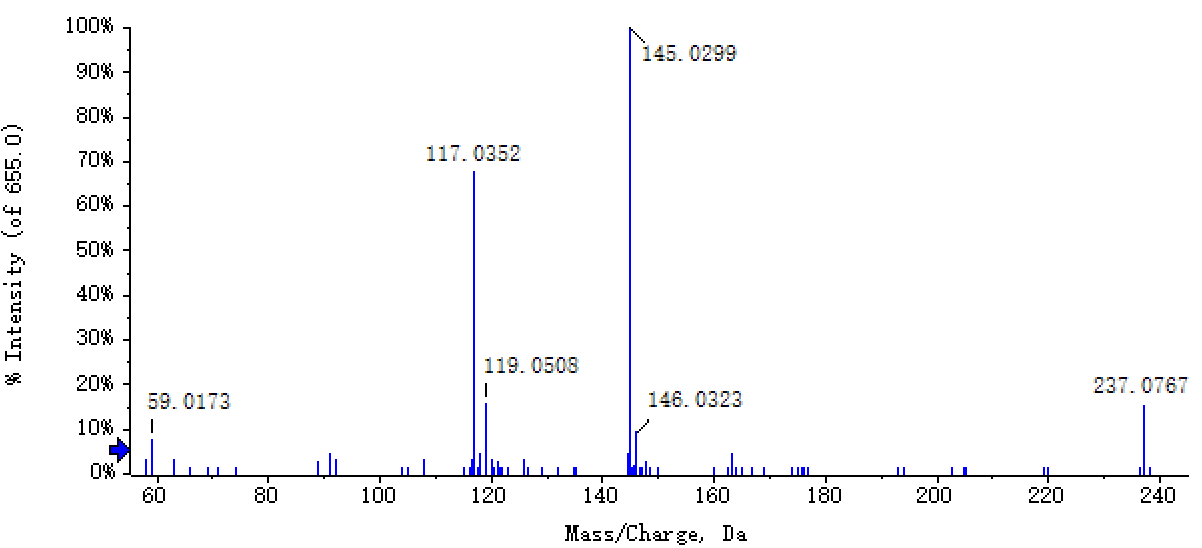


SL20: 2-Propenoic acid, 3-(4-hydroxyphenyl)-, 2,3-dihydroxypropyl ester, (E)-


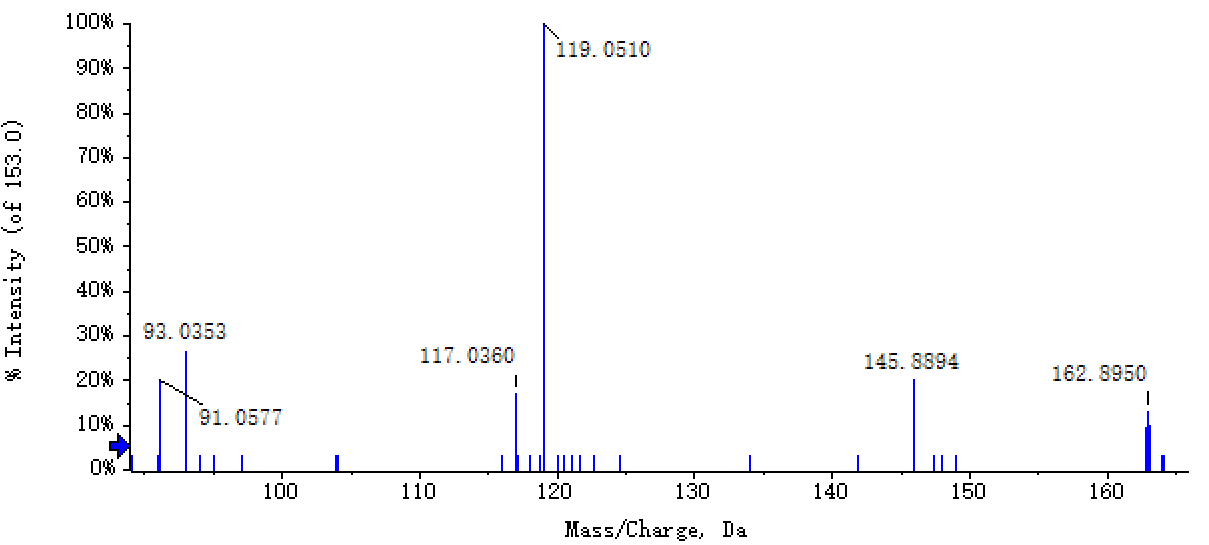


SL21: 4-Coumaric acid


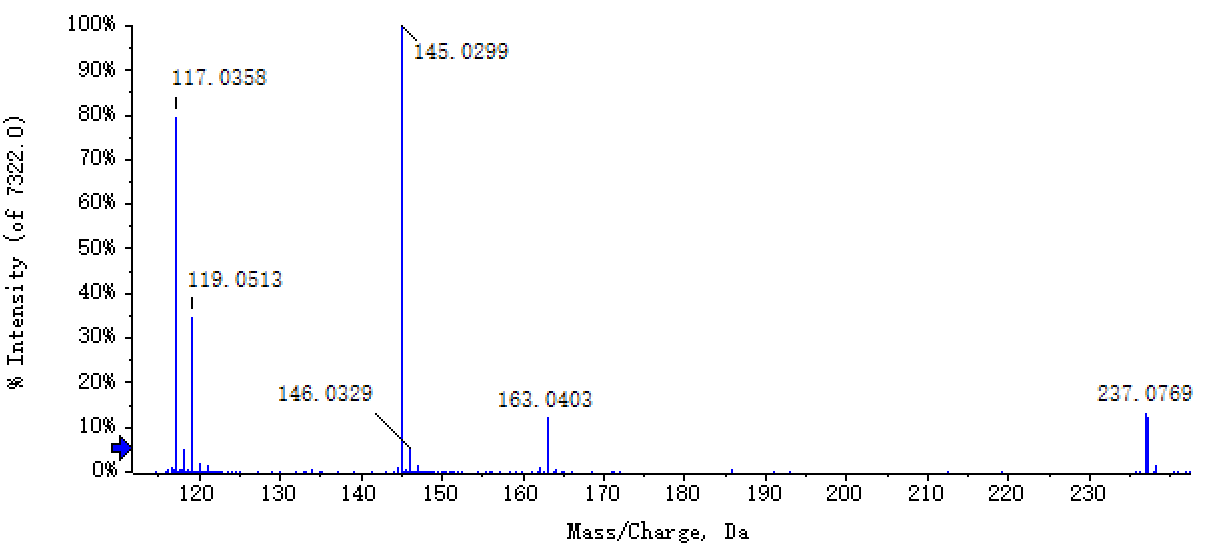


SL22: 1-O-p-coumaroylglycerol


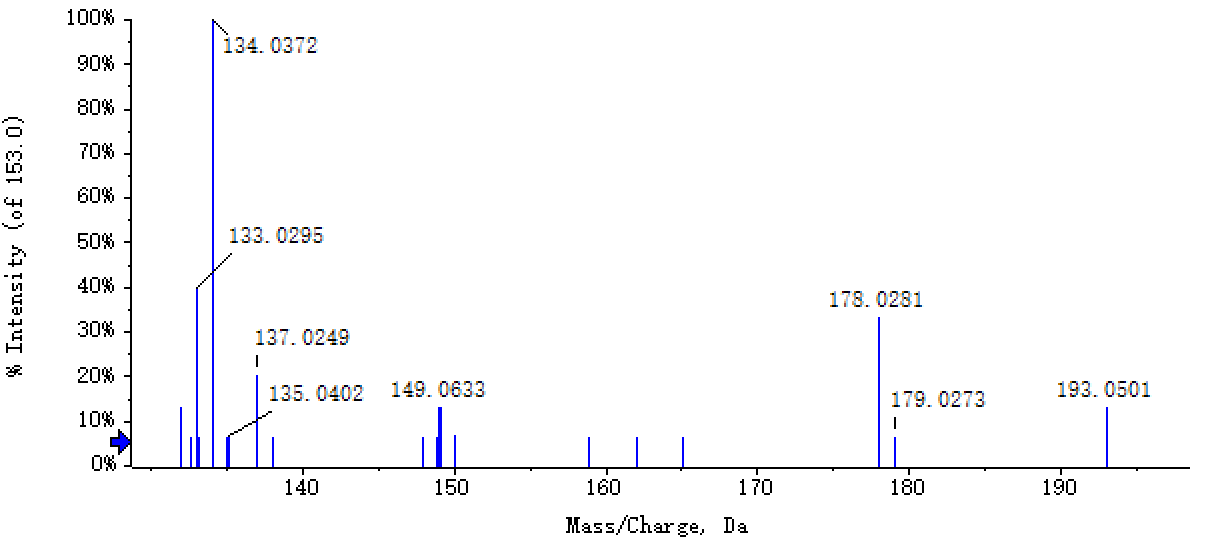


SL23: ferulic acid


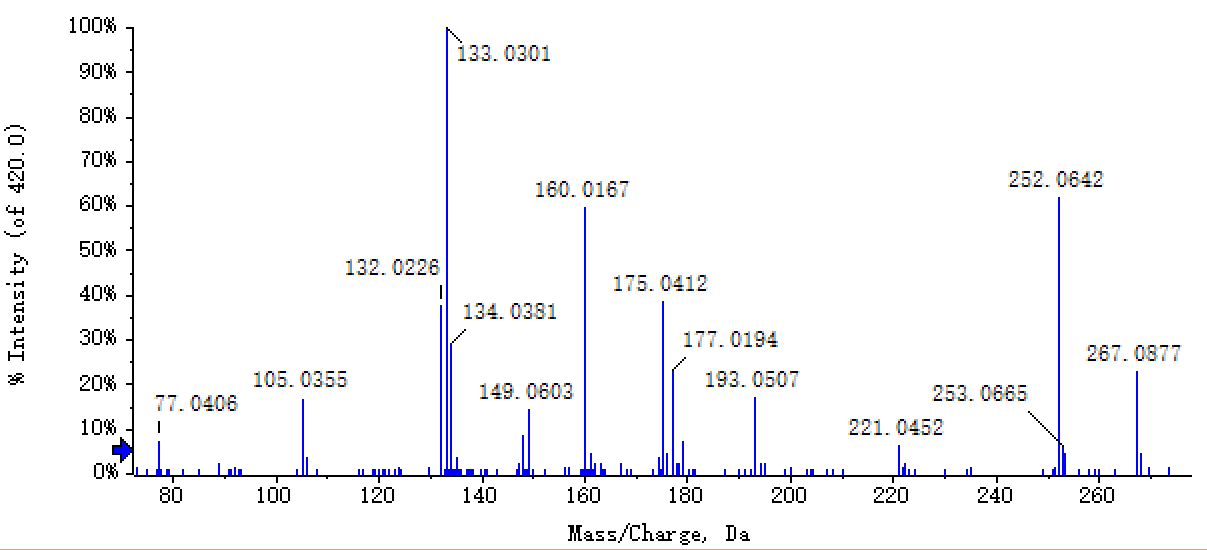


SL24: 2-Propenoic acid, 3-(4-hydroxy-3-methoxyphenyl)-, 2,3-dihydroxypropyl ester, (2Z)-


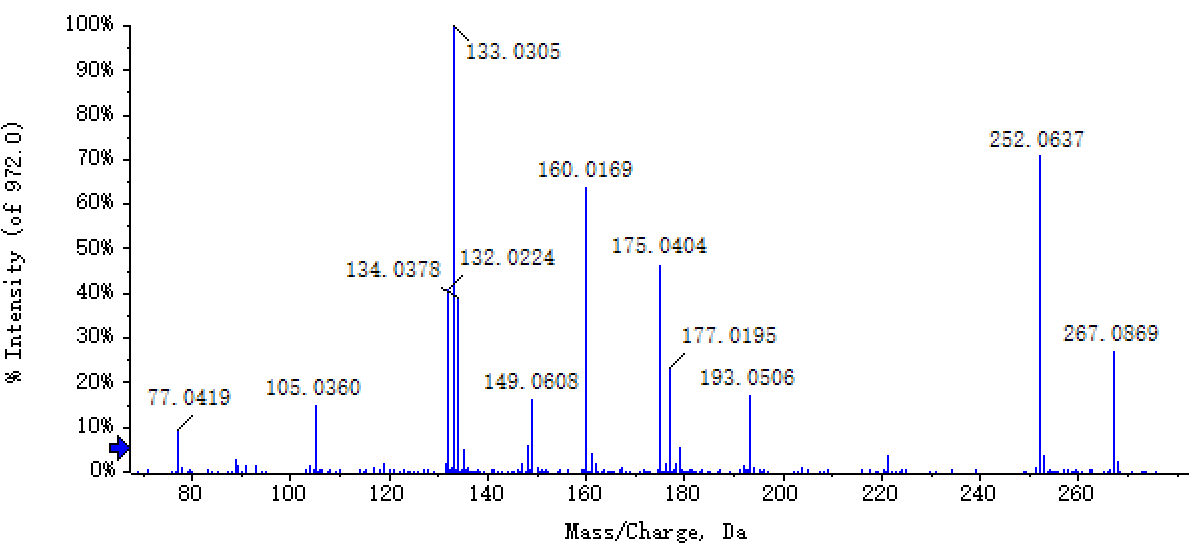


SL25: 1-O-trans-Feruloylglycerol


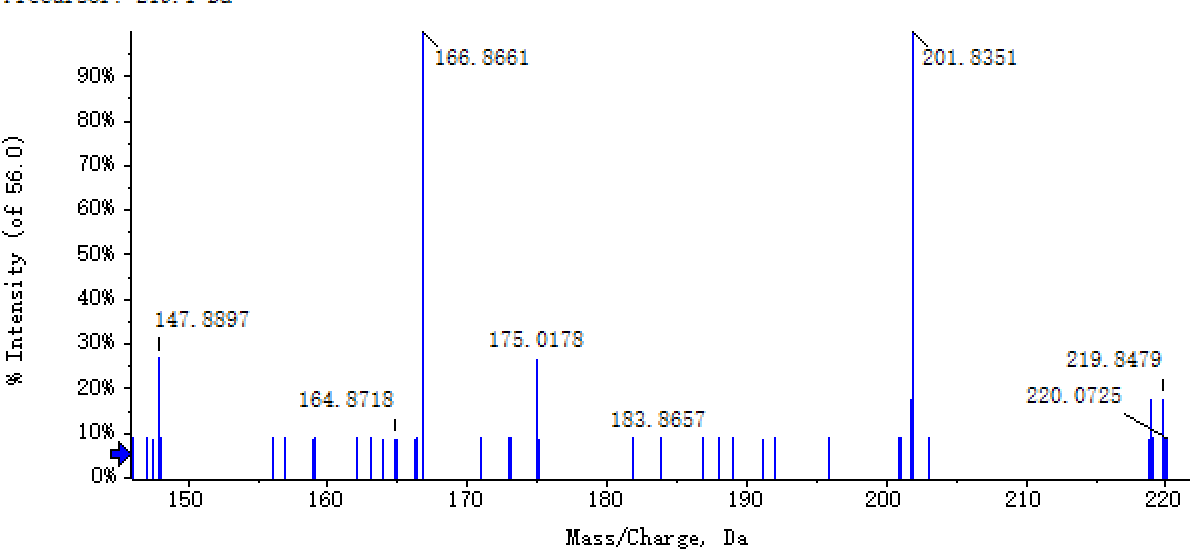


SL26: Decarboxy-citrinone


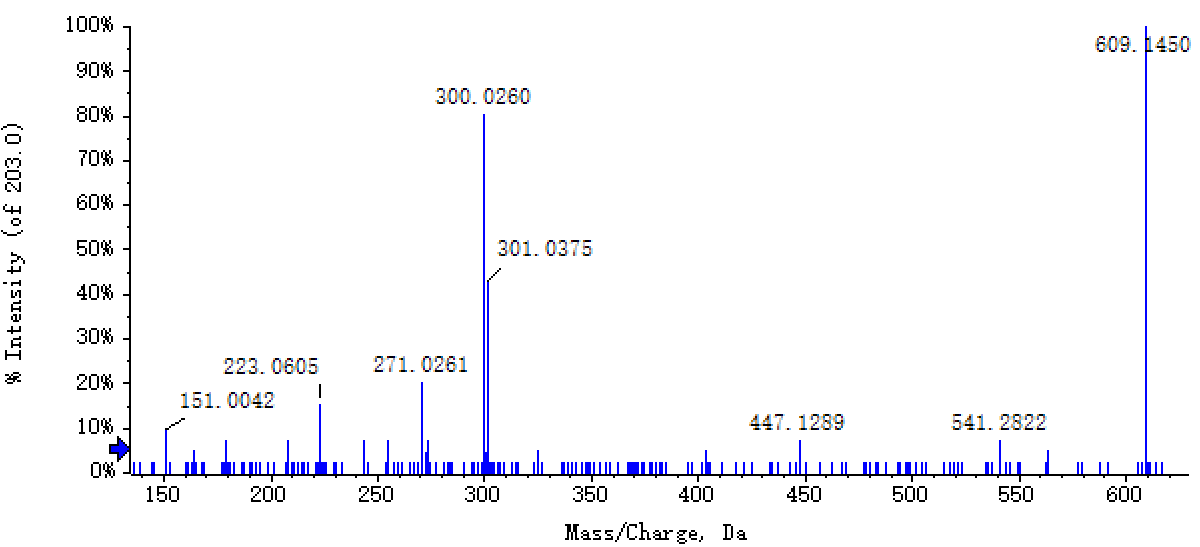


SL27: rutin


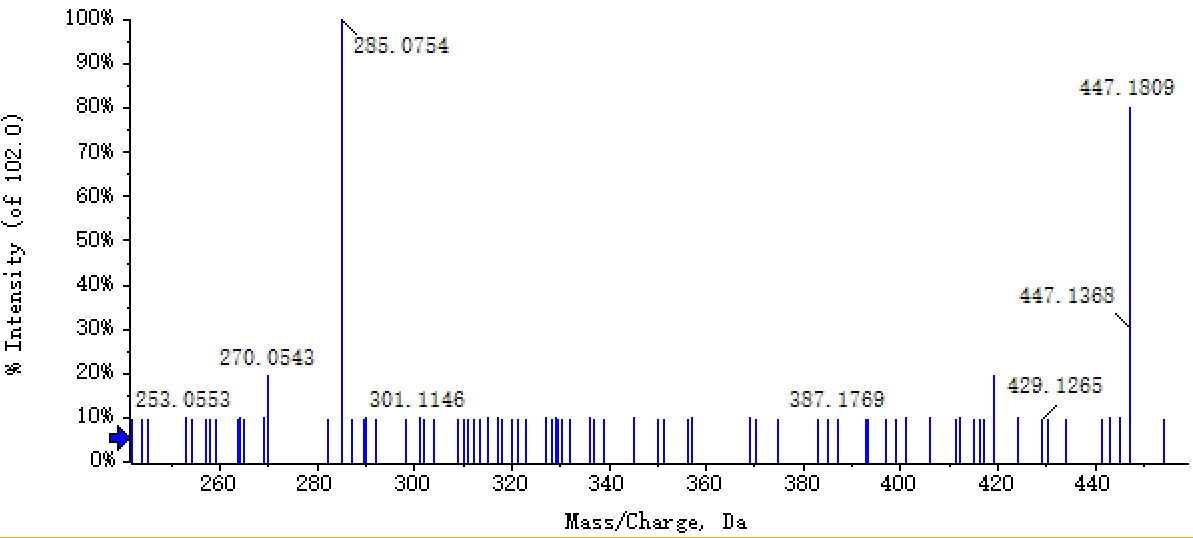


SL28: Tilianin


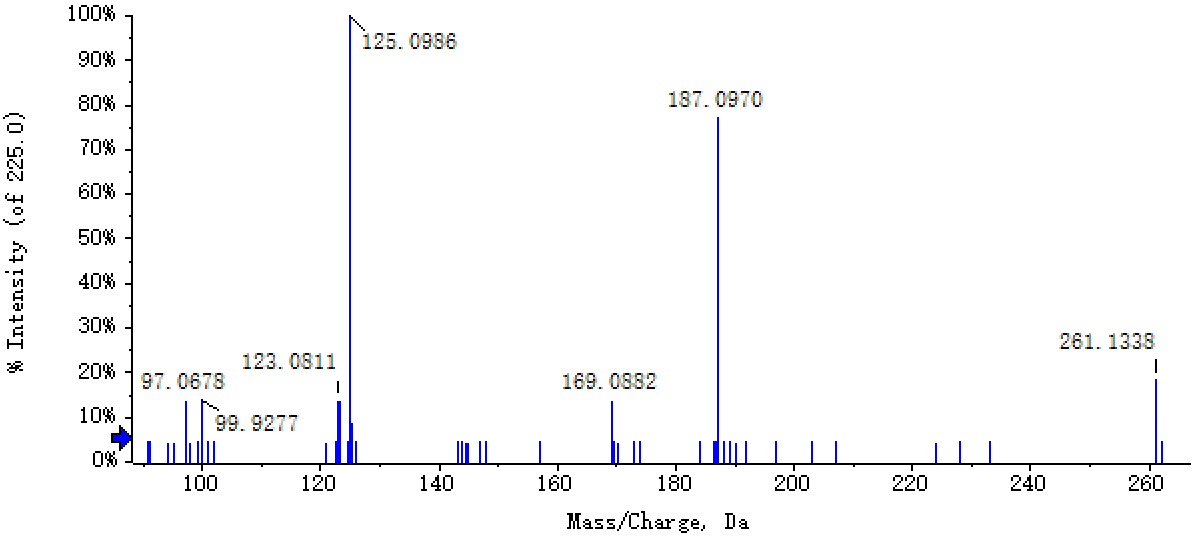


SL29: 9-(2′,3′-Dihydroxypropyloxy)-9-oxononanoic Acid


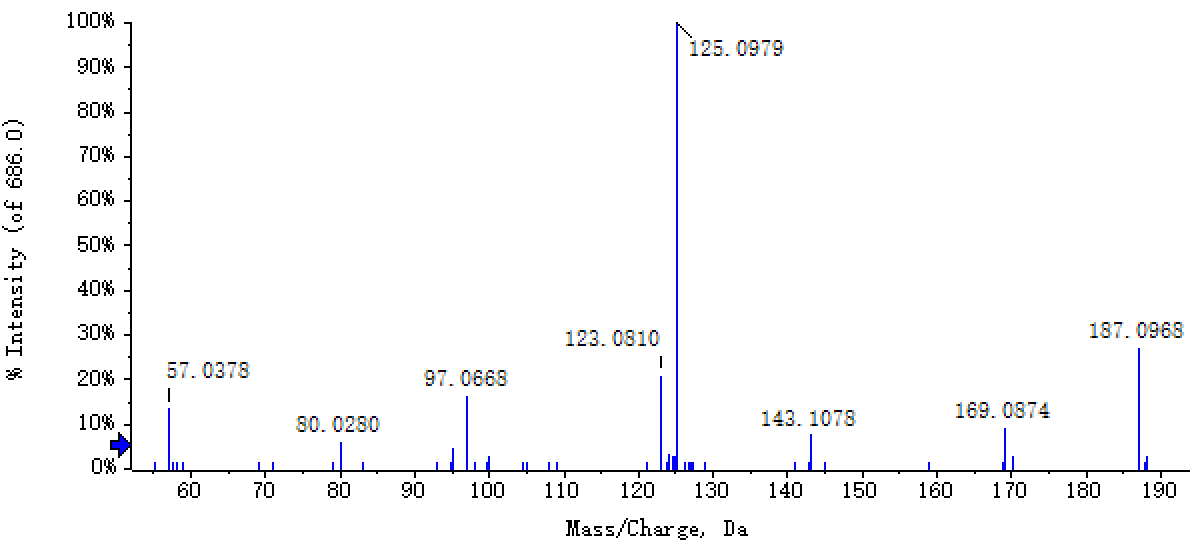


SL30: Azelaic acid


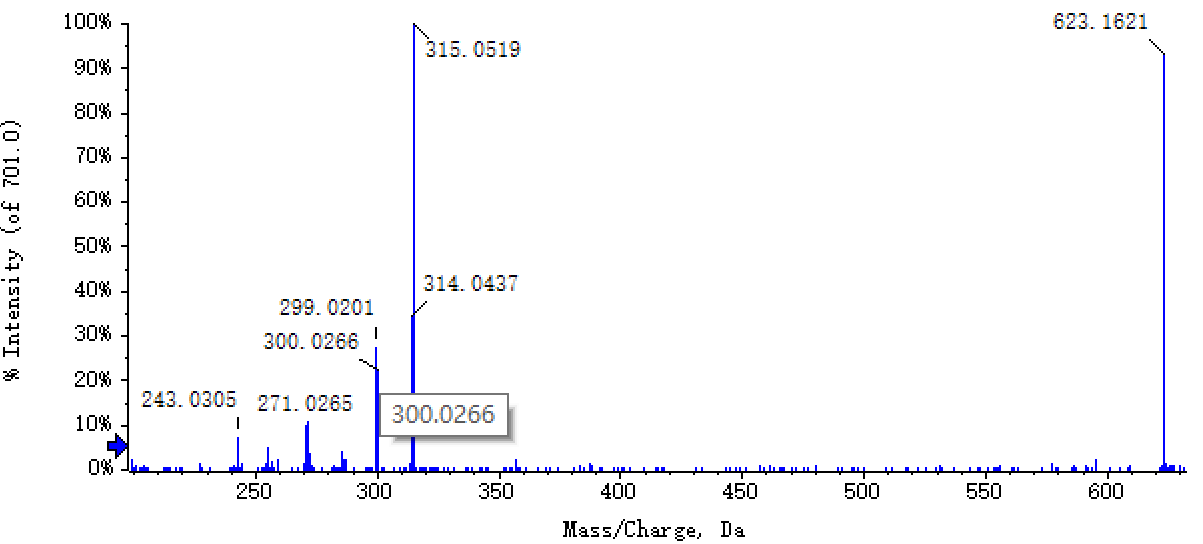


SL31: Narcissin


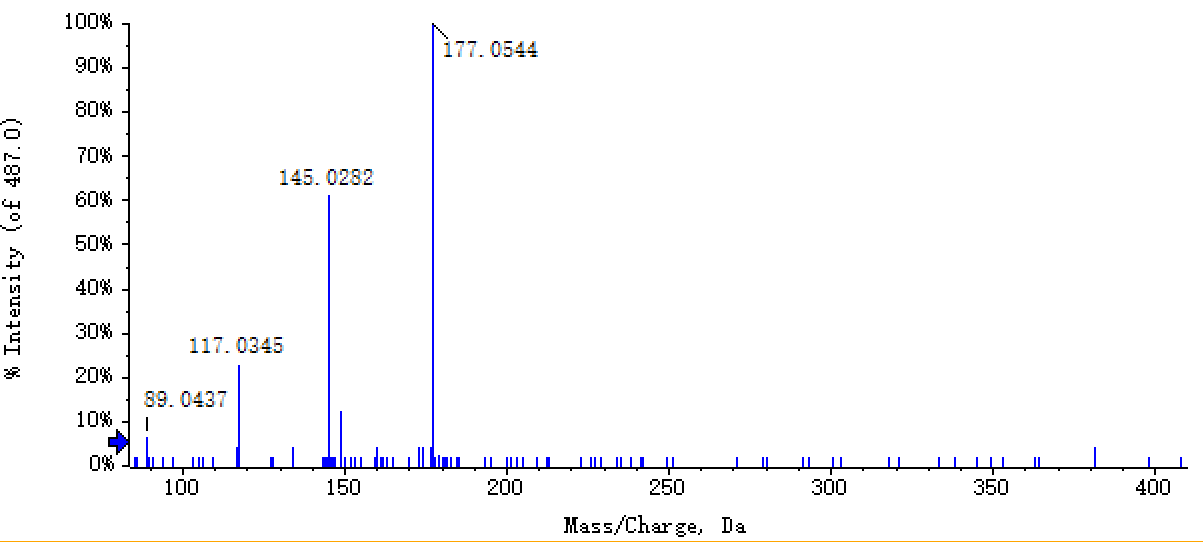


SL32: β-D-Glucopyranosiduronic acid, 4-methyl-2-oxo-2H-1-benzopyran-7-yl, ethyl ester


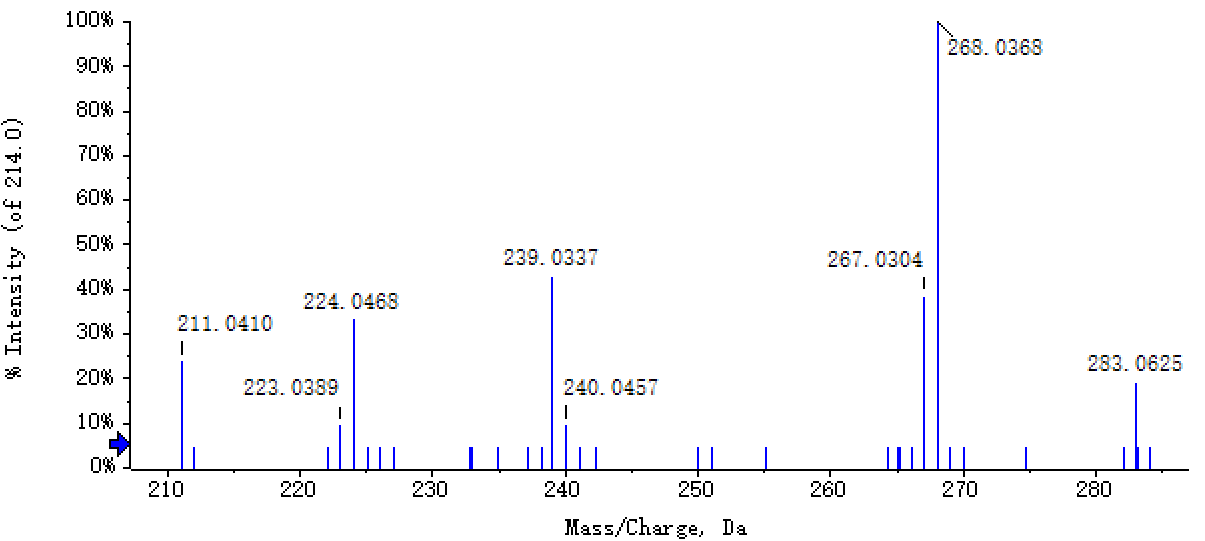


SL33: Acacetin


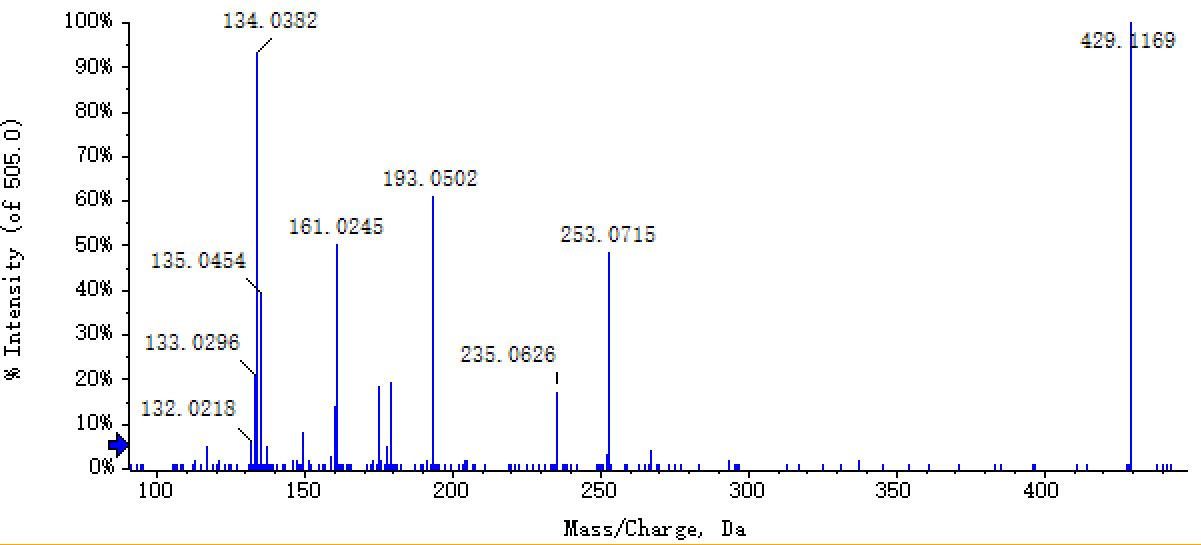


SL34: Feruloyl-caffeoylglycerol


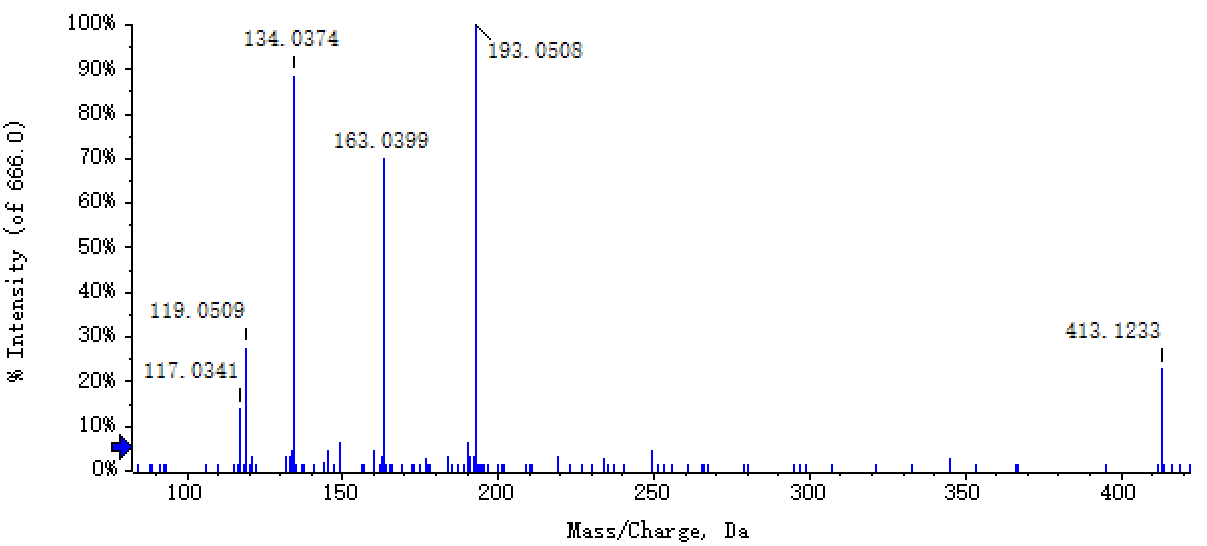


SL35: *p*-Coumaroyl-feruloylglycerol


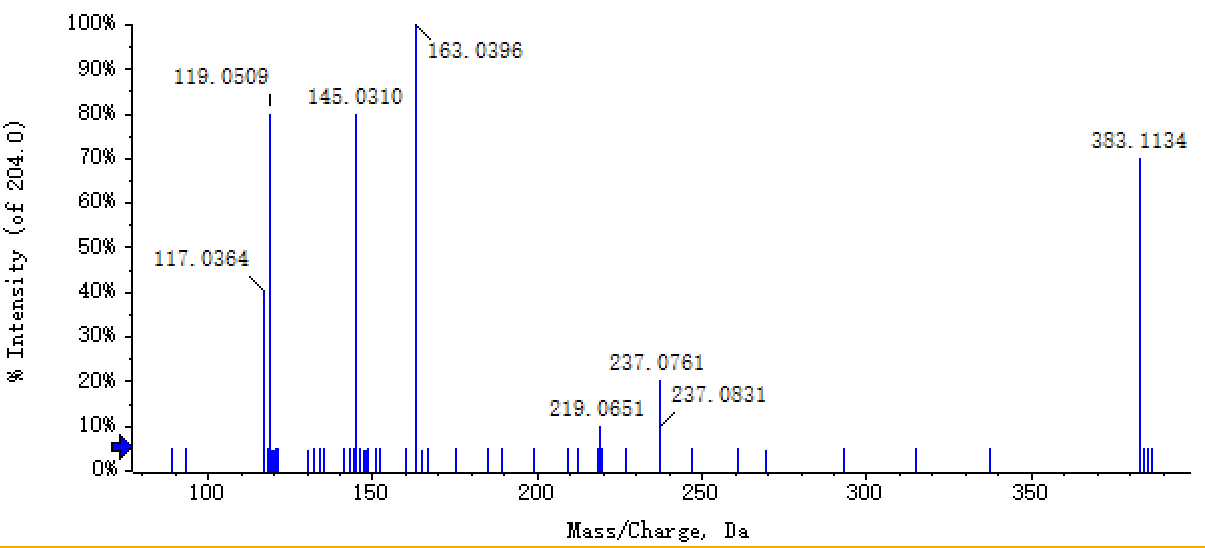


SL36: 1,3-O-Di-trans-p-coumaroylglycerol


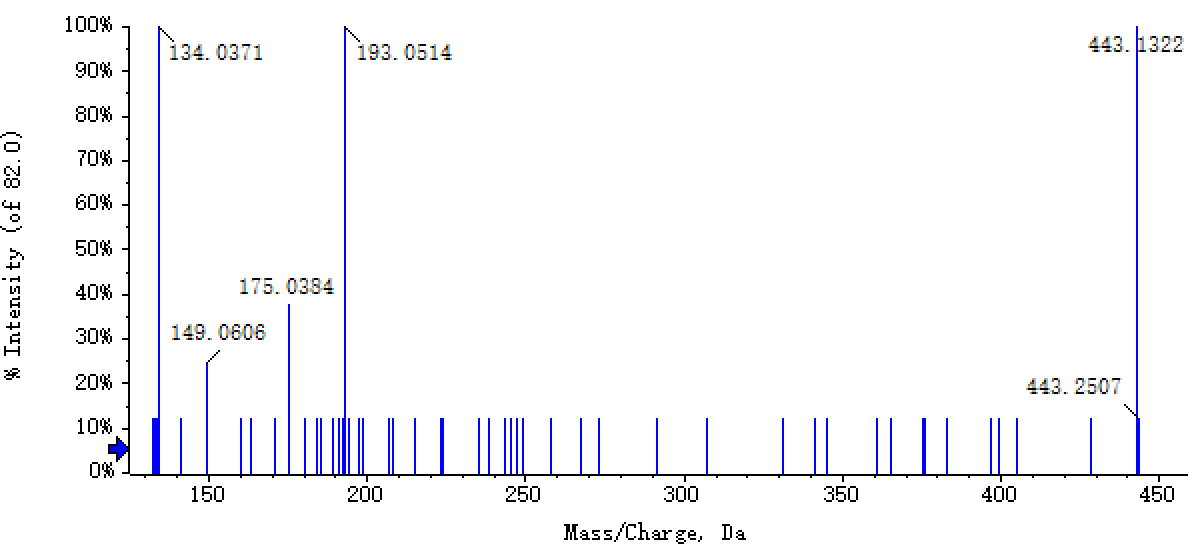


SL37: 1,3-O-Diferuloyl glycerol


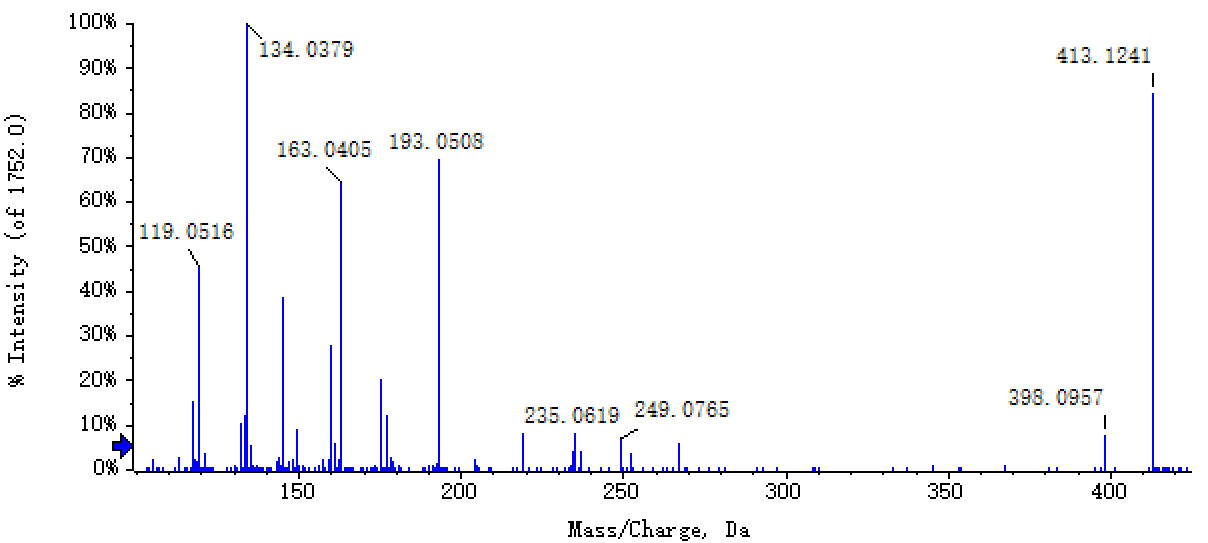


SL38: 1-O-Feruloyl-3-O-p-coumaroylglycerol


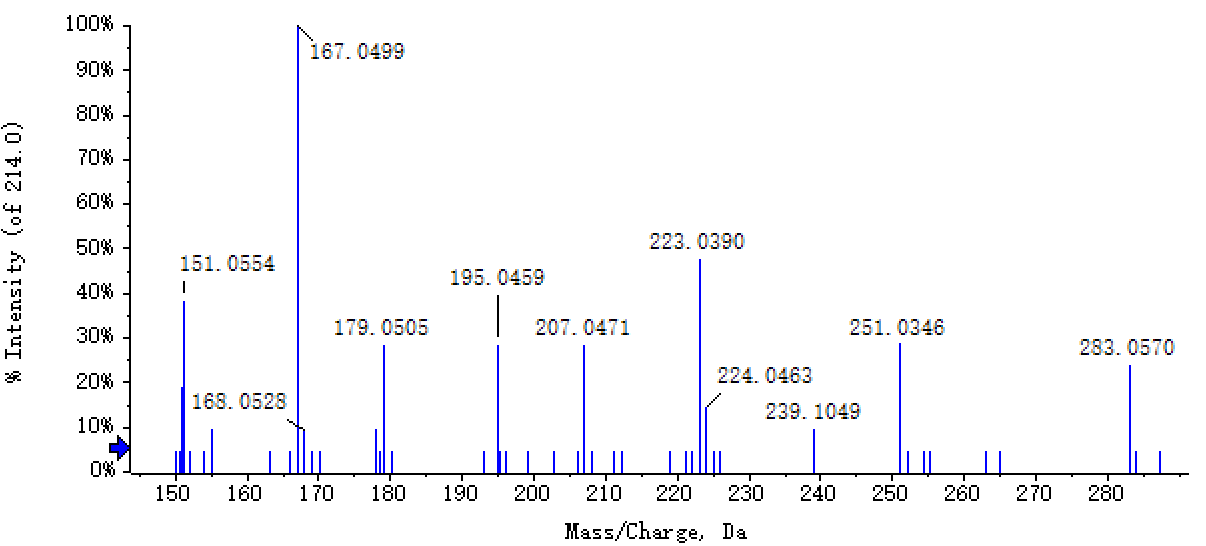


SL39: Methyl 3, 6-dihydroxy-2-[( 2-hydroxyphenyl) ethynyl]benzoate


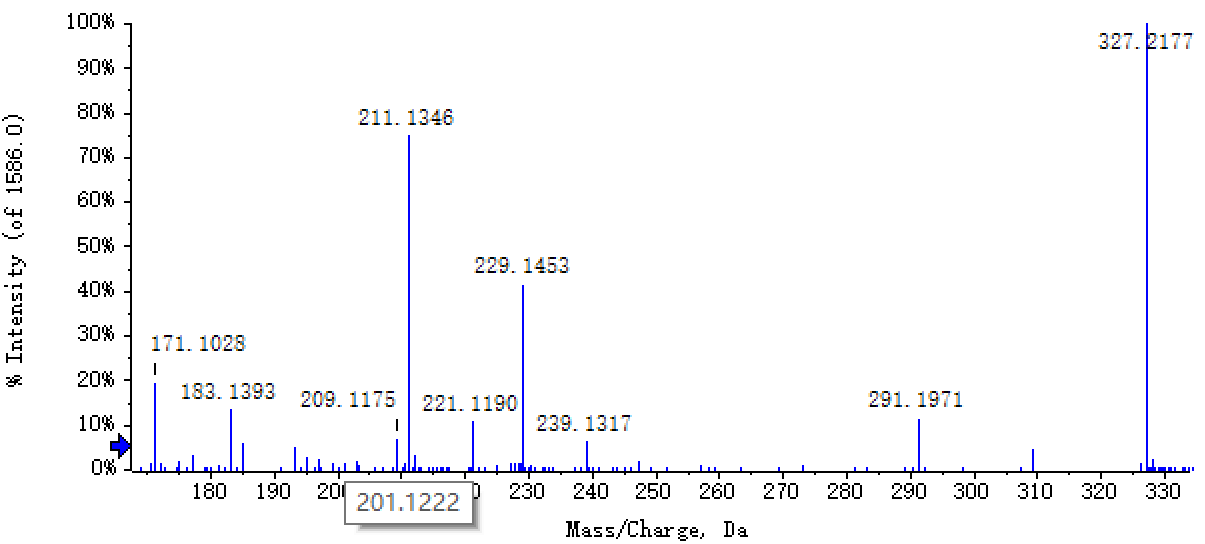


SL40: 9S,12R,13S-Trihydroxy-10E,15Zoctadecadienoic

Acid


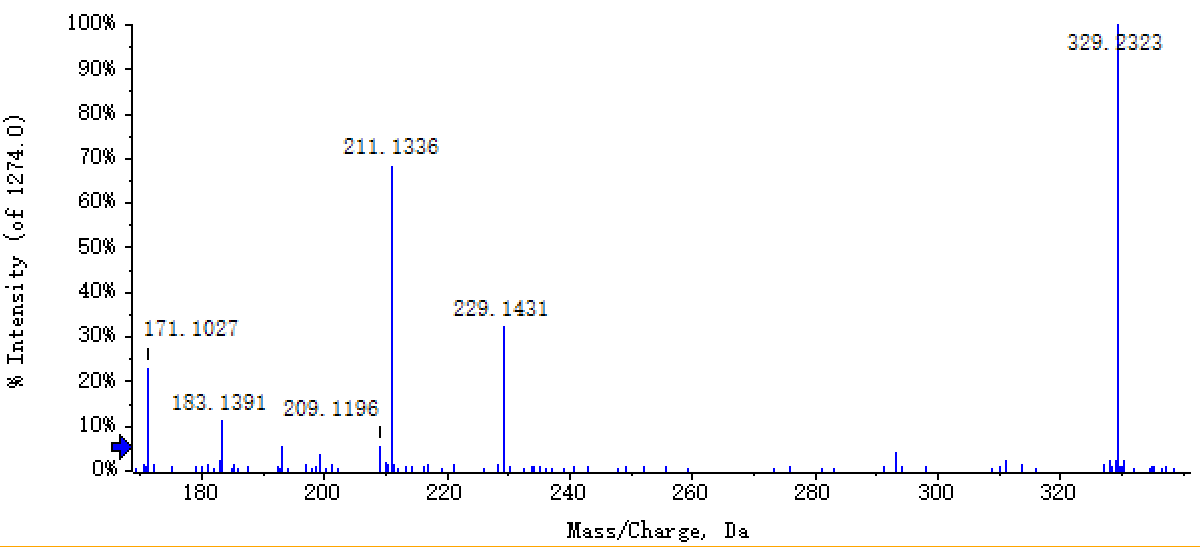


SL41: sanleng acid
