# Supplementary material for: Study of the active ingredients and mechanism of Sparganii rhizoma in gastric cancer based on HPLC-Q-TOF–MS/MS and network pharmacology
Source: Sci Rep. 2021 Jan 21;11:1905. doi: 10.1038/s41598-021-81485-0 (PMC7820434; doi:10.1038/s41598-021-81485-0)
Supplement: Supplementary file 2 — Supplementary Tables. [file 41598_2021_81485_MOESM2_ESM.docx]

Study of the Active Ingredients and Mechanism of *Sparganii rhizoma* in Gastric Cancer Based on HPLC-Q-TOF-MS/MS and Network Pharmacology

Xiaona Lu^1,2†^, Yawei Zheng^2†^, Fang Wen^2^, Wenjie Huang^2^, Xiaoxue Chen^2^, Shuai Ruan^2^, Suping Gu, Yue Hu^1,2^, Yuhao Teng^1,2^ and Peng Shu^1,2*^

^1^ Oncology Department, Affiliated Hospital of Nanjing University of Chinese Medicine, Nanjing, China.

^2^ First School of Clinical Medicine, Nanjing University of Chinese Medicine, Nanjing, China.

**†**these authors contributed equally to this work

*****corresponding.shupengsp@njucm.edu.cn

**Table S1.** The 471 identified potential targets of SL

| NO. | Gene names | Uniprot ID |
| --- | --- | --- |
| 1 | SLC5A7 | Q9GZV3 |
| 2 | NOS2 | P35228 |
| 3 | NOS1 | P29475 |
| 4 | ARG1 | P05089 |
| 5 | CA2 | P00918 |
| 6 | CA1 | P00915 |
| 7 | CA12 | O43570 |
| 8 | CA9 | Q16790 |
| 9 | HCAR2 | Q8TDS4 |
| 10 | DDO | Q99489 |
| 11 | MMP9 | P14780 |
| 12 | MMP2 | P08253 |
| 13 | GDA | Q9Y2T3 |
| 14 | ADORA2A | P29274 |
| 15 | CCNE1 | P24864 |
| 16 | CDK2 | P24941 |
| 17 | ADORA1 | P30542 |
| 18 | ADORA2B | P29275 |
| 19 | SIRT3 | Q9NTG7 |
| 20 | SIRT2 | Q8IXJ6 |
| 21 | SLC22A6 | Q4U2R8 |
| 22 | FYN | P06241 |
| 23 | LCK | P06239 |
| 24 | DPYD | Q12882 |
| 25 | TYMS | P04818 |
| 26 | PARP1 | P09874 |
| 27 | EGLN1 | Q9GZT9 |
| 28 | HDAC3 | O15379 |
| 29 | KDM4E | B2RXH2 |
| 30 | EGFR | P00533 |
| 31 | TH | P07101 |
| 32 | CACNA2D1 | P54289 |
| 33 | THRA | P10827 |
| 34 | THRB | P10828 |
| 35 | SLC7A5 | Q01650 |
| 36 | NR1H4 | Q96RI1 |
| 37 | APEX1 | P27695 |
| 38 | PTPRA | P18433 |
| 39 | TAAR1 | Q96RJ0 |
| 40 | KMO | O15229 |
| 41 | KDM4C | Q9H3R0 |
| 42 | GABBR2 | O75899 |
| 43 | GABBR1 | Q9UBS5 |
| 44 | PEPD | P12955 |
| 45 | MAPKAPK2 | P49137 |
| 46 | CPB1 | P15086 |
| 47 | GRB2 | P62993 |
| 48 | KIF11 | P52732 |
| 49 | ESR1 | P03372 |
| 50 | ESR2 | Q92731 |
| 51 | GABRA1 | P14867 |
| 52 | GABRB2 | P47870 |
| 53 | GABRG2 | P18507 |
| 54 | SLC6A11 | P48066 |
| 55 | SLC6A13 | Q9NSD5 |
| 56 | GABRR1 | P24046 |
| 57 | SLC6A1 | P30531 |
| 58 | OAT | P04181 |
| 59 | SLC1A1 | P43005 |
| 60 | GRM4 | Q14833 |
| 61 | GRM5 | P41594 |
| 62 | GRM8 | O00222 |
| 63 | GRM1 | Q13255 |
| 64 | GRM7 | Q14831 |
| 65 | DPP4 | P27487 |
| 66 | ADK | P55263 |
| 67 | ADORA3 | P0DMS8 |
| 68 | HSPA8 | P11142 |
| 69 | HSPA5 | P11021 |
| 70 | AHCY | P23526 |
| 71 | ADA | P00813 |
| 72 | GAPDH | P04406 |
| 73 | EHMT1 | Q9H9B1 |
| 74 | EHMT2 | Q96KQ7 |
| 75 | MCL1 | Q07820 |
| 76 | SETD7 | Q8WTS6 |
| 77 | SRM | P19623 |
| 78 | AMD1 | P17707 |
| 79 | PDCD4 | Q53EL6 |
| 80 | SRC | P12931 |
| 81 | MAPK1 | P28482 |
| 82 | SLC29A1 | Q99808 |
| 83 | PNP | P00491 |
| 84 | SETD2 | Q9BYW2 |
| 85 | CARM1 | Q86X55 |
| 86 | PRMT1 | Q99873 |
| 87 | DOT1L | Q8TEK3 |
| 88 | P2RY1 | P47900 |
| 89 | P2RY11 | Q96G91 |
| 90 | GRK1 | Q15835 |
| 91 | SMS | P52788 |
| 92 | MTAP | Q13126 |
| 93 | FBP1 | P09467 |
| 94 | FHIT | P49789 |
| 95 | QARS | P47897 |
| 96 | KMT2A | Q03164 |
| 97 | SUV39H1 | O43463 |
| 98 | DNMT1 | P26358 |
| 99 | INMT | O95050 |
| 100 | SMYD2 | Q9NRG4 |
| 101 | EZH2 | Q15910 |
| 102 | EZH1 | Q92800 |
| 103 | SETDB1 | Q15047 |
| 104 | PNMT | P11086 |
| 105 | DNMT3B | Q9UBC3 |
| 106 | CDA | P32320 |
| 107 | HSPA1A | P0DMV8 |
| 108 | CCND1 | P24385 |
| 109 | CDK4 | P11802 |
| 110 | RARS | P54136 |
| 111 | PIM1 | P11309 |
| 112 | P2RX1 | P51575 |
| 113 | HSD17B1 | P14061 |
| 114 | GBA | P04062 |
| 115 | OGA | O60502 |
| 116 | MARS | P56192 |
| 117 | CA7 | P43166 |
| 118 | CA13 | Q8N1Q1 |
| 119 | PRMT7 | Q9NVM4 |
| 120 | GAA | P10253 |
| 121 | PARG | Q86W56 |
| 122 | GSK3B | P49841 |
| 123 | FUCA1 | P04066 |
| 124 | IARS | P41252 |
| 125 | CA14 | Q9ULX7 |
| 126 | SLC5A2 | P31639 |
| 127 | SLC28A2 | O43868 |
| 128 | DAO | P14920 |
| 129 | CA3 | P07451 |
| 130 | CA6 | P23280 |
| 131 | CA5A | P35218 |
| 132 | CA4 | P22748 |
| 133 | TPMT | P51580 |
| 134 | TTR | P02766 |
| 135 | CA5B | Q9Y2D0 |
| 136 | FUT7 | Q11130 |
| 137 | KDM4A | O75164 |
| 138 | KDM3A | Q9Y4C1 |
| 139 | KDM6B | O15054 |
| 140 | FTO | Q9C0B1 |
| 141 | AKR1C3 | P42330 |
| 142 | KDM2A | Q9Y2K7 |
| 143 | MMP1 | P03956 |
| 144 | MMP8 | P22894 |
| 145 | SQLE | Q14534 |
| 146 | POLA1 | P09884 |
| 147 | POLB | P06746 |
| 148 | SERPINE1 | P05121 |
| 149 | TUBB1 | Q9H4B7 |
| 150 | TACR1 | P25103 |
| 151 | ADRA2A | P08913 |
| 152 | ADRA2B | P18089 |
| 153 | ADRA1A | P35348 |
| 154 | CPB2 | Q96IY4 |
| 155 | KDM1A | O60341 |
| 156 | CPA3 | P15088 |
| 157 | SLC15A1 | P46059 |
| 158 | ANPEP | P15144 |
| 159 | ENPEP | Q07075 |
| 160 | SLC6A2 | P23975 |
| 161 | SLC6A4 | P31645 |
| 162 | RNPEP | Q9H4A4 |
| 163 | SRD5A2 | P31213 |
| 164 | AKR1C2 | P52895 |
| 165 | AKR1C1 | Q04828 |
| 166 | ERN1 | O75460 |
| 167 | HDAC6 | Q9UBN7 |
| 168 | HDAC8 | Q9BY41 |
| 169 | LDHA | P00338 |
| 170 | LDHB | P07195 |
| 171 | AKR1B1 | P15121 |
| 172 | NOX4 | Q9NPH5 |
| 173 | CAMKK2 | Q96RR4 |
| 174 | GPR35 | Q9HC97 |
| 175 | ECE1 | P42892 |
| 176 | ALOX5 | P09917 |
| 177 | PTPN1 | P18031 |
| 178 | AKR1B10 | O60218 |
| 179 | MIF | P14174 |
| 180 | NQO2 | P16083 |
| 181 | TLR4 | O00206 |
| 182 | AKR1C4 | P17516 |
| 183 | NGFR | P08138 |
| 184 | SYK | P43405 |
| 185 | APP | P05067 |
| 186 | CTBP2 | P56545 |
| 187 | MAOB | P27338 |
| 188 | PTGS1 | P23219 |
| 189 | PIK3CB | P42338 |
| 190 | CYP1A2 | P05177 |
| 191 | CYP2C9 | P11712 |
| 192 | CYP3A4 | P08684 |
| 193 | CYP2C19 | P33261 |
| 194 | PIK3CA | P42336 |
| 195 | ELANE | P08246 |
| 196 | F3 | P13726 |
| 197 | HSD11B1 | P28845 |
| 198 | NFE2L2 | Q16236 |
| 199 | STAT3 | P40763 |
| 200 | FOLH1 | Q04609 |
| 201 | TRPA1 | O75762 |
| 202 | PGR | P06401 |
| 203 | MET | P08581 |
| 204 | CYP1A1 | P04798 |
| 205 | CYP1B1 | Q16678 |
| 206 | CPA1 | P15085 |
| 207 | PTGS2 | P35354 |
| 208 | RELA | Q04206 |
| 209 | SLC16A1 | P53985 |
| 210 | TLR9 | Q9NR96 |
| 211 | ALOX15 | P16050 |
| 212 | PRKCE | Q02156 |
| 213 | TUBB3 | Q13509 |
| 214 | ABCB1 | P08183 |
| 215 | ACE | P12821 |
| 216 | REN | P00797 |
| 217 | TOP2A | P11388 |
| 218 | GLO1 | Q04760 |
| 219 | MAOA | P21397 |
| 220 | BACE1 | P56817 |
| 221 | AHR | P35869 |
| 222 | F2 | P00734 |
| 223 | CTNNB1 | P35222 |
| 224 | SLC13A5 | Q86YT5 |
| 225 | MMP13 | P45452 |
| 226 | MMP12 | P39900 |
| 227 | SLC5A1 | P13866 |
| 228 | SLC28A3 | Q9HAS3 |
| 229 | TYR | P14679 |
| 230 | PRKCA | P17252 |
| 231 | HRAS | P01112 |
| 232 | ERBB2 | P04626 |
| 233 | MAPK14 | Q16539 |
| 234 | MAP2K1 | Q02750 |
| 235 | ADAM17 | P78536 |
| 236 | SLC5A4 | Q9NY91 |
| 237 | MMP7 | P09237 |
| 238 | IMPDH1 | P20839 |
| 239 | IMPDH2 | P12268 |
| 240 | IGFBP3 | P17936 |
| 241 | NMUR2 | Q9GZQ4 |
| 242 | ADRA2C | P18825 |
| 243 | ACHE | P22303 |
| 244 | RPS6KA3 | P51812 |
| 245 | XDH | P47989 |
| 246 | CD38 | P28907 |
| 247 | PDE5A | O76074 |
| 248 | TNF | P01375 |
| 249 | IL2 | P60568 |
| 250 | TERT | O14746 |
| 251 | ALDH2 | P05091 |
| 252 | MGMT | P16455 |
| 253 | PDGFRA | P16234 |
| 254 | PDGFRB | P09619 |
| 255 | NRAS | P01111 |
| 256 | F10 | P00742 |
| 257 | FABP4 | P15090 |
| 258 | FABP3 | P05413 |
| 259 | FABP5 | Q01469 |
| 260 | PPARD | Q03181 |
| 261 | FFAR1 | O14842 |
| 262 | FABP2 | P12104 |
| 263 | PPARA | Q07869 |
| 264 | AR | P10275 |
| 265 | VDR | P11473 |
| 266 | CDC25A | P30304 |
| 267 | GPBAR1 | Q8TDU6 |
| 268 | UGT2B7 | P16662 |
| 269 | CYP19A1 | P11511 |
| 270 | SERPINA6 | P08185 |
| 271 | SHBG | P04278 |
| 272 | HSD17B3 | P37058 |
| 273 | G6PD | P11413 |
| 274 | KDM5C | P41229 |
| 275 | PTGFR | P43088 |
| 276 | NPC1L1 | Q9UHC9 |
| 277 | GABRA2 GABRB2 GABRG2 | P47869 |
| 278 | HSD11B2 | P80365 |
| 279 | PHF8 | Q9UPP1 |
| 280 | FNTA | P49354 |
| 281 | FNTB | P49356 |
| 282 | PLG | P00747 |
| 283 | PTGER2 | P43116 |
| 284 | CDC45 | O75419 |
| 285 | LTA4H | P09960 |
| 286 | HAO1 | Q9UJM8 |
| 287 | CHRNA7 | P36544 |
| 288 | GSTK1 | Q9Y2Q3 |
| 289 | FDPS | P14324 |
| 290 | EGLN3 | Q9H6Z9 |
| 291 | FDFT1 | P37268 |
| 292 | PTGDR2 | Q9Y5Y4 |
| 293 | PTPRC | P08575 |
| 294 | CBR1 | P16152 |
| 295 | ABCC1 | P33527 |
| 296 | TNKS2 | Q9H2K2 |
| 297 | TNKS | O95271 |
| 298 | HSD17B2 | P37059 |
| 299 | CDK5R1 | Q15078 |
| 300 | CDK5 | Q00535 |
| 301 | CCNB3 | Q8WWL7 |
| 302 | CDK1 | P06493 |
| 303 | CCNB1 | P14635 |
| 304 | CCNB2 | O95067 |
| 305 | CDK6 | Q00534 |
| 306 | ABCG2 | Q9UNQ0 |
| 307 | FLT3 | P36888 |
| 308 | KIT | P10721 |
| 309 | OPRD1 | P41143 |
| 310 | ALOX12 | P18054 |
| 311 | PTPRS | Q13332 |
| 312 | CSNK2A1 | P68400 |
| 313 | CFTR | P13569 |
| 314 | GRK6 | P43250 |
| 315 | TOP1 | P11387 |
| 316 | PLA2G2A | P14555 |
| 317 | NAE1 | Q13564 |
| 318 | AMY1A | P04745 |
| 319 | ESRRA | P11474 |
| 320 | SLC22A12 | Q96S37 |
| 321 | PFKFB3 | Q16875 |
| 322 | IGF1R | P08069 |
| 323 | ST6GAL1 | P15907 |
| 324 | SIGMAR1 | Q99720 |
| 325 | CALM1 | P62158 |
| 326 | DAPK1 | P53355 |
| 327 | MPG | P29372 |
| 328 | OPRM1 | P35372 |
| 329 | BCHE | P06276 |
| 330 | AVPR2 | P30518 |
| 331 | AURKB | Q96GD4 |
| 332 | DRD4 | P21917 |
| 333 | MPO | P05164 |
| 334 | PIK3R1 | P27986 |
| 335 | PYGL | P06737 |
| 336 | PTK2 | Q05397 |
| 337 | KDR | P35968 |
| 338 | MMP3 | P08254 |
| 339 | PLK1 | P53350 |
| 340 | EP300 | Q09472 |
| 341 | IKBKG | Q9Y6K9 |
| 342 | IKBKB | O14920 |
| 343 | CHUK | O15111 |
| 344 | TDP1 | Q9NUW8 |
| 345 | PTGES | O14684 |
| 346 | YARS | P54577 |
| 347 | GRIK1 | P39086 |
| 348 | HTR1A | P08908 |
| 349 | PGF | P49763 |
| 350 | VEGFA | P15692 |
| 351 | NADK | O95544 |
| 352 | AKT1 | P31749 |
| 353 | SRD5A1 | P18405 |
| 354 | HSP90AA1 | P07900 |
| 355 | PLAU | P00749 |
| 356 | MCHR1 | Q99705 |
| 357 | LIPG | Q9Y5X9 |
| 358 | CCNA2 CDK2 | P20248 |
| 359 | RET | P07949 |
| 360 | DYRK1A | Q13627 |
| 361 | BTK | Q06187 |
| 362 | CAPN1 | P07384 |
| 363 | CAPNS1 | P04632 |
| 364 | F11 | P03951 |
| 365 | MTOR | P42345 |
| 366 | PIK3CD | O00329 |
| 367 | PIK3CG | P48736 |
| 368 | PDE10A | Q9Y233 |
| 369 | EIF4A1 | P60842 |
| 370 | F3 F7 | P08709 |
| 371 | FKBP1A | P62942 |
| 372 | MAP3K14 | Q99558 |
| 373 | DNM1 | Q05193 |
| 374 | DHFR | P00374 |
| 375 | CTSD | P07339 |
| 376 | MGLL | Q99685 |
| 377 | PRSS1 | P07477 |
| 378 | PRSS3 | P35030 |
| 379 | CHEK1 | O14757 |
| 380 | PLA2G7 | Q13093 |
| 381 | TRPM8 | Q7Z2W7 |
| 382 | FASN | P49327 |
| 383 | GUSB | P08236 |
| 384 | NQO1 | P15559 |
| 385 | MME | P08473 |
| 386 | EIF2AK2 | P19525 |
| 387 | CDK9 | P50750 |
| 388 | CCNT1 | O60563 |
| 389 | PTK2B | Q14289 |
| 390 | ROCK1 | Q13464 |
| 391 | BRAF | P15056 |
| 392 | ALPG | P10696 |
| 393 | PLAA | Q9Y263 |
| 394 | CXCR2 | P25025 |
| 395 | GSK3A | P49840 |
| 396 | DYRK1B | Q9Y463 |
| 397 | PPIA | P62937 |
| 398 | MMP16 | P51512 |
| 399 | DUSP3 | P51452 |
| 400 | MMP14 | P50281 |
| 401 | DBF4 | Q9UBU7 |
| 402 | CDC7 | O00311 |
| 403 | KCNMA1 | Q12791 |
| 404 | PIM2 | Q9P1W9 |
| 405 | ALPL | P05186 |
| 406 | JAK3 | P52333 |
| 407 | ADAMTS5 | Q9UNA0 |
| 408 | PTGER1 | P34995 |
| 409 | PTGER4 | P35408 |
| 410 | PTGER3 | P43115 |
| 411 | PTGIR | P43119 |
| 412 | PTGDR | Q13258 |
| 413 | PPARG | P37231 |
| 414 | LTB4R | Q15722 |
| 415 | FABP1 | P07148 |
| 416 | AMPD1 | P23109 |
| 417 | CES2 | O00748 |
| 418 | PLA2G1B | P04054 |
| 419 | GRM2 | Q14416 |
| 420 | PDE4D | Q08499 |
| 421 | FAAH | O00519 |
| 422 | RORA | P35398 |
| 423 | GCK | P35557 |
| 424 | GART | P22102 |
| 425 | PTPN2 | P17706 |
| 426 | CD81 | P60033 |
| 427 | ADRB2 | P07550 |
| 428 | ADRB1 | P08588 |
| 429 | ADRB3 | P13945 |
| 430 | TYRO3 | Q06418 |
| 431 | ITGA2B | P08514 |
| 432 | ITGB3 | P05106 |
| 433 | SSTR5 | P35346 |
| 434 | CASP3 | P42574 |
| 435 | PTPN6 | P29350 |
| 436 | NR3C1 | P04150 |
| 437 | EDNRA | P25101 |
| 438 | MAPK10 | P53779 |
| 439 | HMGCR | P04035 |
| 440 | TBXA2R | P21731 |
| 441 | AURKA | O14965 |
| 442 | CTSA | P10619 |
| 443 | AMPD2 | Q01433 |
| 444 | DGAT1 | O75907 |
| 445 | FLT1 | P17948 |
| 446 | DPP7 | Q9UHL4 |
| 447 | PDE6D | O43924 |
| 448 | ITGB1 | P05556 |
| 449 | ITGA4 | P13612 |
| 450 | EDNRB | P24530 |
| 451 | CASR | P41180 |
| 452 | HSP90AB1 | P08238 |
| 453 | SCD | O00767 |
| 454 | SOAT1 | P35610 |
| 455 | PTPN22 | Q9Y2R2 |
| 456 | CYP17A1 | P05093 |
| 457 | RHOA | P61586 |
| 458 | MDM2 | Q00987 |
| 459 | SCN10A | Q9Y5Y9 |
| 460 | SCN9A | Q15858 |
| 461 | TKT | P29401 |
| 462 | PDE4B | Q07343 |
| 463 | NR1H3 | Q13133 |
| 464 | GLP1R | P43220 |
| 465 | GCGR | P47871 |
| 466 | PTPN7 | P35236 |
| 467 | GIPR | P48546 |
| 468 | PLA2G4B | P0C869 |
| 469 | PREP | P48147 |
| 470 | KCNH2 | Q12809 |
| 471 | ITK | Q08881 |

**Table S2.** The 3225 identified genes relevant to GC

| NO. | Gene names |
| --- | --- |
| 1 | CDH1 |
| 2 | TP53 |
| 3 | APC |
| 4 | MLH1 |
| 5 | BRCA1 |
| 6 | BRCA2 |
| 7 | MSH2 |
| 8 | ATM |
| 9 | ERBB2 |
| 10 | KRAS |
| 11 | EGFR |
| 12 | PTEN |
| 13 | CDKN2A |
| 14 | CHEK2 |
| 15 | MSH6 |
| 16 | CTNNB1 |
| 17 | MET |
| 18 | PIK3CA |
| 19 | VEGFA |
| 20 | MUTYH |
| 21 | STK11 |
| 22 | AKT1 |
| 23 | MYC |
| 24 | CCND1 |
| 25 | SMAD4 |
| 26 | BRAF |
| 27 | IL1B |
| 28 | ESR1 |
| 29 | RB1 |
| 30 | TGFBR2 |
| 31 | TERT |
| 32 | TNF |
| 33 | CDKN1B |
| 34 | EGF |
| 35 | HRAS |
| 36 | CDKN1A |
| 37 | IL6 |
| 38 | STAT3 |
| 39 | CDK4 |
| 40 | BAX |
| 41 | PALB2 |
| 42 | TGFB1 |
| 43 | PMS2 |
| 44 | MTOR |
| 45 | KIT |
| 46 | FGFR2 |
| 47 | AXIN2 |
| 48 | FAS |
| 49 | CTNNA1 |
| 50 | MIR21 |
| 51 | IL1RN |
| 52 | BMPR1A |
| 53 | CASP8 |
| 54 | NRAS |
| 55 | NOTCH1 |
| 56 | TYMS |
| 57 | MDM2 |
| 58 | SMARCA4 |
| 59 | SDHD |
| 60 | MGMT |
| 61 | NF1 |
| 62 | IGF2 |
| 63 | FASLG |
| 64 | IL10 |
| 65 | PTGS2 |
| 66 | SDHB |
| 67 | CDKN2B |
| 68 | MTHFR |
| 69 | TYMP |
| 70 | AURKA |
| 71 | AR |
| 72 | DNMT1 |
| 73 | PLAU |
| 74 | EZH2 |
| 75 | SMAD3 |
| 76 | BCL2 |
| 77 | FHIT |
| 78 | FGFR1 |
| 79 | MIR34A |
| 80 | RAF1 |
| 81 | ERCC1 |
| 82 | TLR4 |
| 83 | MAP2K1 |
| 84 | MIR145 |
| 85 | H19 |
| 86 | TOP2A |
| 87 | ESR2 |
| 88 | IRF1 |
| 89 | DICER1 |
| 90 | MIR146A |
| 91 | TWIST1 |
| 92 | PIK3R1 |
| 93 | PTCH1 |
| 94 | AKT2 |
| 95 | BARD1 |
| 96 | CXCR4 |
| 97 | EPCAM |
| 98 | SLC2A1 |
| 99 | MIR27A |
| 100 | MMP9 |
| 101 | SOX2 |
| 102 | MIR17 |
| 103 | SRC |
| 104 | GLI1 |
| 105 | KLF6 |
| 106 | RET |
| 107 | MIR31 |
| 108 | DPYD |
| 109 | MIR143 |
| 110 | CXCL8 |
| 111 | RAD51C |
| 112 | TP73 |
| 113 | NFKB1 |
| 114 | CASP3 |
| 115 | TGFBR1 |
| 116 | MUC1 |
| 117 | MIR141 |
| 118 | MIR125A |
| 119 | INS |
| 120 | TCF7L2 |
| 121 | MIR126 |
| 122 | MIR221 |
| 123 | PPARG |
| 124 | MAPK1 |
| 125 | SMAD7 |
| 126 | GAST |
| 127 | NFE2L2 |
| 128 | MEN1 |
| 129 | CASP10 |
| 130 | FGFR3 |
| 131 | BRIP1 |
| 132 | MIR200C |
| 133 | GSTP1 |
| 134 | HIF1A |
| 135 | RAD51D |
| 136 | MMP2 |
| 137 | IFNG |
| 138 | GSTM1 |
| 139 | ODC1 |
| 140 | RAD51 |
| 141 | OGG1 |
| 142 | WNT5A |
| 143 | MRE11 |
| 144 | CXCL12 |
| 145 | MIR222 |
| 146 | YAP1 |
| 147 | MIR214 |
| 148 | TGFB2 |
| 149 | MIR223 |
| 150 | ABCB1 |
| 151 | TERC |
| 152 | BIRC5 |
| 153 | MIR203A |
| 154 | PDGFRB |
| 155 | PTPN11 |
| 156 | MIR10B |
| 157 | JUN |
| 158 | EP300 |
| 159 | TFF1 |
| 160 | MIR20A |
| 161 | SPP1 |
| 162 | FLT1 |
| 163 | WNT1 |
| 164 | IGF1 |
| 165 | PDGFRA |
| 166 | CD44 |
| 167 | ERCC2 |
| 168 | XIAP |
| 169 | MIR22 |
| 170 | MIR29C |
| 171 | MIR25 |
| 172 | ARID1A |
| 173 | SMARCB1 |
| 174 | IGF1R |
| 175 | POLE |
| 176 | PROM1 |
| 177 | MIR106B |
| 178 | NBN |
| 179 | ZEB1 |
| 180 | TP63 |
| 181 | MIRLET7A1 |
| 182 | ZEB2 |
| 183 | MSH3 |
| 184 | HGF |
| 185 | MIR106A |
| 186 | FGF2 |
| 187 | MUC5AC |
| 188 | CEACAM5 |
| 189 | TGFA |
| 190 | MIR155 |
| 191 | RAD50 |
| 192 | MIR150 |
| 193 | JAK2 |
| 194 | PSCA |
| 195 | AXIN1 |
| 196 | FOXP3 |
| 197 | PGR |
| 198 | BCL2L1 |
| 199 | MIR144 |
| 200 | TNFRSF10B |
| 201 | MIR93 |
| 202 | XRCC1 |
| 203 | TSC2 |
| 204 | HSPB1 |
| 205 | MIR16-1 |
| 206 | LEP |
| 207 | MIR142 |
| 208 | MAPK3 |
| 209 | MIR200A |
| 210 | ZFHX3 |
| 211 | CAT |
| 212 | NOTCH3 |
| 213 | GNAS |
| 214 | RELA |
| 215 | HMOX1 |
| 216 | MIR107 |
| 217 | STAT1 |
| 218 | CREB1 |
| 219 | PDCD1 |
| 220 | MMP7 |
| 221 | NOS2 |
| 222 | GHRL |
| 223 | SNAI2 |
| 224 | CD274 |
| 225 | MIR191 |
| 226 | VEGFC |
| 227 | CHEK1 |
| 228 | KCNQ1OT1 |
| 229 | ATP4A |
| 230 | SDHC |
| 231 | RASSF1 |
| 232 | CDX2 |
| 233 | JAG1 |
| 234 | ERBB4 |
| 235 | MIR205 |
| 236 | MIR182 |
| 237 | MIR192 |
| 238 | SP1 |
| 239 | MUC6 |
| 240 | MIR15B |
| 241 | CASP9 |
| 242 | ABCG2 |
| 243 | MIR451A |
| 244 | PLAUR |
| 245 | KDR |
| 246 | CDK2 |
| 247 | CD40LG |
| 248 | NOTCH2 |
| 249 | MIR122 |
| 250 | MIR200B |
| 251 | FH |
| 252 | SST |
| 253 | E2F1 |
| 254 | ERBB3 |
| 255 | VDR |
| 256 | BIRC3 |
| 257 | NTRK1 |
| 258 | NQO1 |
| 259 | ERCC6 |
| 260 | HNF4A |
| 261 | MIR148A |
| 262 | VHL |
| 263 | CDKN3 |
| 264 | TACC1 |
| 265 | KRT20 |
| 266 | RARB |
| 267 | MIAT |
| 268 | MAP2K2 |
| 269 | MIR29B1 |
| 270 | MIR195 |
| 271 | MIR29A |
| 272 | HOTAIR |
| 273 | RUNX3 |
| 274 | CCNE1 |
| 275 | CYCS |
| 276 | MAPK8 |
| 277 | TSC1 |
| 278 | WNT2B |
| 279 | WT1 |
| 280 | CD40 |
| 281 | ABCC1 |
| 282 | MIR193A |
| 283 | FOS |
| 284 | ICAM1 |
| 285 | IL2 |
| 286 | MIR100 |
| 287 | MIR181A1 |
| 288 | CDK6 |
| 289 | SMAD2 |
| 290 | MPO |
| 291 | RPS6KB1 |
| 292 | PDGFB |
| 293 | ALB |
| 294 | TIMP1 |
| 295 | TLR2 |
| 296 | KRT19 |
| 297 | MUC2 |
| 298 | MMP14 |
| 299 | BAP1 |
| 300 | SNAI1 |
| 301 | ABCC2 |
| 302 | LRP5 |
| 303 | CCKBR |
| 304 | IDH1 |
| 305 | AFP |
| 306 | RHOA |
| 307 | MIR335 |
| 308 | MIR34C |
| 309 | SOD2 |
| 310 | S100A8 |
| 311 | BMP2 |
| 312 | MIR204 |
| 313 | CCNB1 |
| 314 | MIR24-2 |
| 315 | DNMT3A |
| 316 | BAK1 |
| 317 | MALAT1 |
| 318 | ACTB |
| 319 | MIR373 |
| 320 | NME1 |
| 321 | CYP1A1 |
| 322 | MIR30E |
| 323 | ENG |
| 324 | GSK3B |
| 325 | WWOX |
| 326 | FLT4 |
| 327 | PRKCA |
| 328 | PLK1 |
| 329 | PTK2 |
| 330 | MMP1 |
| 331 | MIR483 |
| 332 | TIMP2 |
| 333 | CTSD |
| 334 | ADIPOQ |
| 335 | MIR183 |
| 336 | CYP19A1 |
| 337 | CYP17A1 |
| 338 | NFKBIA |
| 339 | IGFBP3 |
| 340 | THBS1 |
| 341 | DLEC1 |
| 342 | PCNA |
| 343 | MIR15A |
| 344 | NF2 |
| 345 | MIR18A |
| 346 | MIR140 |
| 347 | CTLA4 |
| 348 | CHGA |
| 349 | PHB |
| 350 | ABL1 |
| 351 | TIMP3 |
| 352 | HRH2 |
| 353 | MIR127 |
| 354 | MIR215 |
| 355 | AREG |
| 356 | MIR424 |
| 357 | ROS1 |
| 358 | DNMT3B |
| 359 | MKI67 |
| 360 | MIR99A |
| 361 | UCA1 |
| 362 | E2F3 |
| 363 | DCC |
| 364 | SHH |
| 365 | NCOA3 |
| 366 | H3-3A |
| 367 | MMP3 |
| 368 | CYP2D6 |
| 369 | BAD |
| 370 | COMT |
| 371 | SERPINB5 |
| 372 | GRP |
| 373 | MIR23A |
| 374 | PRKAR1A |
| 375 | MIRLET7B |
| 376 | MIR196B |
| 377 | ANXA5 |
| 378 | KRT7 |
| 379 | CDK1 |
| 380 | GSTT1 |
| 381 | CSF3 |
| 382 | CRP |
| 383 | GRB2 |
| 384 | MCL1 |
| 385 | BCL10 |
| 386 | DAPK1 |
| 387 | HBEGF |
| 388 | MIR199A1 |
| 389 | PVT1 |
| 390 | PTGS1 |
| 391 | CCND2 |
| 392 | MIR9-1 |
| 393 | S100A4 |
| 394 | HLA-A |
| 395 | ACE |
| 396 | KRT18 |
| 397 | MIR375 |
| 398 | MIR146B |
| 399 | FGF1 |
| 400 | VEGFD |
| 401 | RMRP |
| 402 | PIK3CB |
| 403 | MIR23B |
| 404 | MIR224 |
| 405 | MAPK14 |
| 406 | F2 |
| 407 | MIR486-1 |
| 408 | MCM4 |
| 409 | FGF7 |
| 410 | RNF43 |
| 411 | LBR |
| 412 | CCK |
| 413 | SKP2 |
| 414 | BSG |
| 415 | MAP3K6 |
| 416 | MEG3 |
| 417 | CAV1 |
| 418 | ALK |
| 419 | PMS1 |
| 420 | RPL15 |
| 421 | JUP |
| 422 | MYCN |
| 423 | FLNC |
| 424 | STAT5B |
| 425 | SHC1 |
| 426 | PAK1 |
| 427 | CCNA2 |
| 428 | GAS5 |
| 429 | SDHA |
| 430 | IL17A |
| 431 | GATA3 |
| 432 | MTAP |
| 433 | KLK3 |
| 434 | RUNX2 |
| 435 | MUC16 |
| 436 | BMI1 |
| 437 | FZD5 |
| 438 | KITLG |
| 439 | WRN |
| 440 | MIRLET7D |
| 441 | KLF4 |
| 442 | CDH2 |
| 443 | SERPINA3 |
| 444 | PRKCD |
| 445 | VIM |
| 446 | CDKN2B-AS1 |
| 447 | CD82 |
| 448 | MIR26A1 |
| 449 | SOX4 |
| 450 | PIK3R3 |
| 451 | CCAT1 |
| 452 | U2AF1 |
| 453 | MAGEA1 |
| 454 | XPC |
| 455 | HLA-DRB1 |
| 456 | IL7 |
| 457 | EDNRA |
| 458 | PAX5 |
| 459 | CSF2 |
| 460 | JAK3 |
| 461 | EZR |
| 462 | MIR331 |
| 463 | GADD45A |
| 464 | FEZF1 |
| 465 | HNF1B |
| 466 | SPINK1 |
| 467 | CBLIF |
| 468 | NEAT1 |
| 469 | LEF1 |
| 470 | IL4 |
| 471 | MTDH |
| 472 | MIR185 |
| 473 | TTN |
| 474 | CTNND1 |
| 475 | CREBBP |
| 476 | HSP90AA1 |
| 477 | TFF2 |
| 478 | FOXM1 |
| 479 | PLCG1 |
| 480 | KEAP1 |
| 481 | MIR149 |
| 482 | CALCA |
| 483 | E2F2 |
| 484 | MIR196A1 |
| 485 | GKN1 |
| 486 | TNFSF11 |
| 487 | TUG1 |
| 488 | SERPINE1 |
| 489 | NTRK3 |
| 490 | XRCC3 |
| 491 | SERPINA1 |
| 492 | IFI27 |
| 493 | MAGEA3 |
| 494 | SUFU |
| 495 | CCAT2 |
| 496 | ENO2 |
| 497 | CCR6 |
| 498 | ITGB3 |
| 499 | NAT2 |
| 500 | TOP1 |
| 501 | BMP6 |
| 502 | LGALS3 |
| 503 | EPHA2 |
| 504 | XIST |
| 505 | EIF4EBP1 |
| 506 | MIR181B1 |
| 507 | EPOR |
| 508 | FLCN |
| 509 | CLDN4 |
| 510 | GCG |
| 511 | FGF4 |
| 512 | CA9 |
| 513 | WNT6 |
| 514 | PIK3CG |
| 515 | RAC1 |
| 516 | MIR30A |
| 517 | FBXW7 |
| 518 | CCNE2 |
| 519 | POMC |
| 520 | CTAG2 |
| 521 | TFF3 |
| 522 | SOD1 |
| 523 | MIRLET7I |
| 524 | FASN |
| 525 | BUB1B |
| 526 | MIR152 |
| 527 | WNT3 |
| 528 | MUC4 |
| 529 | HPSE |
| 530 | CFLAR |
| 531 | CASC2 |
| 532 | ECT2 |
| 533 | GKN2 |
| 534 | KRT17 |
| 535 | FSCN1 |
| 536 | ALOX5 |
| 537 | IKBKG |
| 538 | CTAG1B |
| 539 | MIR193B |
| 540 | MYD88 |
| 541 | PGA3 |
| 542 | LINC-ROR |
| 543 | MIR137 |
| 544 | PARP1 |
| 545 | IDH2 |
| 546 | MIR342 |
| 547 | TNFSF10 |
| 548 | SIRT1 |
| 549 | SPRY4-IT1 |
| 550 | CYP3A4 |
| 551 | MIR133B |
| 552 | HSPA5 |
| 553 | PCAT1 |
| 554 | HOTTIP |
| 555 | HDC |
| 556 | CDKN1C |
| 557 | SYP |
| 558 | ZFAS1 |
| 559 | PGA5 |
| 560 | CYTOR |
| 561 | WNT3A |
| 562 | S100A6 |
| 563 | KRT8 |
| 564 | BECN1 |
| 565 | PGA4 |
| 566 | GRB7 |
| 567 | MIR30B |
| 568 | FZD7 |
| 569 | CASR |
| 570 | CCL2 |
| 571 | CXCL1 |
| 572 | HULC |
| 573 | BUB1 |
| 574 | MAX |
| 575 | CCL5 |
| 576 | CD36 |
| 577 | REG4 |
| 578 | CBL |
| 579 | AKT3 |
| 580 | ADA |
| 581 | CD24 |
| 582 | MCC |
| 583 | GHET1 |
| 584 | IFNA1 |
| 585 | CD19 |
| 586 | HDAC9 |
| 587 | URGCP |
| 588 | GREM1 |
| 589 | PDCD4 |
| 590 | ADH1C |
| 591 | DES |
| 592 | FGFR4 |
| 593 | ATRX |
| 594 | MIR338 |
| 595 | FANCC |
| 596 | BCL2L11 |
| 597 | CDH17 |
| 598 | BMP4 |
| 599 | TNFRSF1B |
| 600 | CTSB |
| 601 | STMN1 |
| 602 | MIR324 |
| 603 | CEACAM6 |
| 604 | TPX2 |
| 605 | MIR423 |
| 606 | MME |
| 607 | MIR27B |
| 608 | LIMK1 |
| 609 | WNT2 |
| 610 | RECQL4 |
| 611 | H2AC18 |
| 612 | IFNA2 |
| 613 | CYP1B1 |
| 614 | CEACAM1 |
| 615 | RUNX1 |
| 616 | ALDH1A1 |
| 617 | MALT1 |
| 618 | HOXA11-AS |
| 619 | CDC42 |
| 620 | MIR24-1 |
| 621 | CLDN3 |
| 622 | PTPN3 |
| 623 | DIABLO |
| 624 | NFKB2 |
| 625 | FGF19 |
| 626 | SNHG1 |
| 627 | NOTCH4 |
| 628 | SNHG16 |
| 629 | TLR9 |
| 630 | EGR1 |
| 631 | ANGPT2 |
| 632 | ACTC1 |
| 633 | COL1A1 |
| 634 | MTA1 |
| 635 | MIR532 |
| 636 | WNT10B |
| 637 | CSNK2A1 |
| 638 | CD34 |
| 639 | PWRN1 |
| 640 | MIR129-1 |
| 641 | MIR181C |
| 642 | IQGAP1 |
| 643 | HLA-B |
| 644 | NKX2-1 |
| 645 | ATAD2 |
| 646 | ADH1B |
| 647 | DANCR |
| 648 | CAPN9 |
| 649 | SCT |
| 650 | UMPS |
| 651 | NOD2 |
| 652 | RARA |
| 653 | RECK |
| 654 | CDX1 |
| 655 | DKK3 |
| 656 | CLDN7 |
| 657 | GACAT2 |
| 658 | AFAP1-AS1 |
| 659 | PANDAR |
| 660 | PTENP1 |
| 661 | CRNDE |
| 662 | BIRC2 |
| 663 | SOX2-OT |
| 664 | CD4 |
| 665 | CR2 |
| 666 | FLT3 |
| 667 | ALDH2 |
| 668 | BID |
| 669 | PRDM2 |
| 670 | ELANE |
| 671 | LMNA |
| 672 | UBE2C |
| 673 | PKM |
| 674 | GJA1 |
| 675 | CXCR2 |
| 676 | FENDRR |
| 677 | MIF |
| 678 | MIR139 |
| 679 | POSTN |
| 680 | RUFY3 |
| 681 | LINC00673 |
| 682 | MIR124-1 |
| 683 | COX5A |
| 684 | BANCR |
| 685 | LZTS1 |
| 686 | TET2 |
| 687 | NANOG |
| 688 | ANXA1 |
| 689 | GATA6 |
| 690 | GACAT3 |
| 691 | SMARCA2 |
| 692 | CCNA1 |
| 693 | PRL |
| 694 | FOXP1 |
| 695 | FOXO1 |
| 696 | CCR7 |
| 697 | PIK3R2 |
| 698 | MIR101-1 |
| 699 | MMP11 |
| 700 | GCRG224 |
| 701 | CYP2C19 |
| 702 | ETV1 |
| 703 | ELAVL1 |
| 704 | SETD2 |
| 705 | TLR5 |
| 706 | XRCC2 |
| 707 | MIR378A |
| 708 | TGFB3 |
| 709 | FGF9 |
| 710 | VIP |
| 711 | DHFR |
| 712 | ITGA6 |
| 713 | IRS1 |
| 714 | ETS1 |
| 715 | BLACAT1 |
| 716 | SSTR2 |
| 717 | NOS1 |
| 718 | PTPRC |
| 719 | KDM4C |
| 720 | IGF2R |
| 721 | DKK1 |
| 722 | BCAR4 |
| 723 | MT-CYB |
| 724 | IL2RA |
| 725 | ELN |
| 726 | CLDN18 |
| 727 | PLA2G2A |
| 728 | ATR |
| 729 | ATP7A |
| 730 | TH |
| 731 | INHBA |
| 732 | PTH |
| 733 | MECOM |
| 734 | IL11 |
| 735 | CNDP2 |
| 736 | CFTR |
| 737 | TLR3 |
| 738 | BLM |
| 739 | XRCC6 |
| 740 | BCL6 |
| 741 | F3 |
| 742 | EPHX1 |
| 743 | ITGB1 |
| 744 | CHUK |
| 745 | CTTN |
| 746 | XRCC5 |
| 747 | WNT9A |
| 748 | CDK12 |
| 749 | DLC1 |
| 750 | KCMF1 |
| 751 | HAGLR |
| 752 | NR3C2 |
| 753 | GNRH1 |
| 754 | MAP2K4 |
| 755 | PDPN |
| 756 | DLL4 |
| 757 | KLF5 |
| 758 | RPS6KB2 |
| 759 | LINC00261 |
| 760 | KMT2A |
| 761 | WNT8B |
| 762 | CCN2 |
| 763 | GDNF |
| 764 | TNFRSF6B |
| 765 | IL3 |
| 766 | L1CAM |
| 767 | DVL1 |
| 768 | DDB2 |
| 769 | HDAC1 |
| 770 | MIR125B1 |
| 771 | NTS |
| 772 | PECAM1 |
| 773 | FBN1 |
| 774 | RIOX2 |
| 775 | CD80 |
| 776 | SLCO1B3 |
| 777 | ZKSCAN1 |
| 778 | EPHB2 |
| 779 | GIP |
| 780 | BTK |
| 781 | SOS2 |
| 782 | AHR |
| 783 | TP53COR1 |
| 784 | PPM1D |
| 785 | ATP12A |
| 786 | FZD2 |
| 787 | TINCR |
| 788 | CACYBP |
| 789 | PSG2 |
| 790 | KLK10 |
| 791 | HNF1A-AS1 |
| 792 | CYP2E1 |
| 793 | CDH13 |
| 794 | SNHG7 |
| 795 | PEBP1 |
| 796 | GIPR |
| 797 | EDN1 |
| 798 | SNHG12 |
| 799 | HMGB1 |
| 800 | ESM1 |
| 801 | POU5F1 |
| 802 | TCF7 |
| 803 | MLH3 |
| 804 | PRNCR1 |
| 805 | MIR210 |
| 806 | HOXA-AS2 |
| 807 | ITGA2B |
| 808 | NCAM1 |
| 809 | LMNB2 |
| 810 | RHBDF2 |
| 811 | MST1R |
| 812 | CASC9 |
| 813 | SNHG15 |
| 814 | HSPD1 |
| 815 | CBR3-AS1 |
| 816 | AGTR1 |
| 817 | TEK |
| 818 | GBA |
| 819 | ARID1B |
| 820 | ACTA2 |
| 821 | KRT5 |
| 822 | HMGA2 |
| 823 | ITGAM |
| 824 | IL24 |
| 825 | FN1 |
| 826 | MLN |
| 827 | APAF1 |
| 828 | LHCGR |
| 829 | EPAS1 |
| 830 | ERCC5 |
| 831 | TUSC7 |
| 832 | ERG |
| 833 | GAPLINC |
| 834 | TNFAIP3 |
| 835 | FUT2 |
| 836 | MIR19A |
| 837 | LNCRNA-ATB |
| 838 | XPA |
| 839 | G6PD |
| 840 | KDM1A |
| 841 | ZEB1-AS1 |
| 842 | MAGEA4 |
| 843 | STAT5A |
| 844 | SNHG5 |
| 845 | SGK1 |
| 846 | MYOD1 |
| 847 | HIF1A-AS2 |
| 848 | PIWIL1 |
| 849 | SOS1 |
| 850 | FOXO3 |
| 851 | MAD1L1 |
| 852 | ERCC4 |
| 853 | MIR92A2 |
| 854 | GACAT1 |
| 855 | FGF23 |
| 856 | EGOT |
| 857 | IGH |
| 858 | ANPEP |
| 859 | POT1 |
| 860 | RNF6 |
| 861 | SERPINB3 |
| 862 | PTTG1 |
| 863 | DLL1 |
| 864 | SNHG20 |
| 865 | CCDC136 |
| 866 | KCNH2 |
| 867 | PGC |
| 868 | DUSP1 |
| 869 | CD79A |
| 870 | NRG1 |
| 871 | CCL11 |
| 872 | DMBT1 |
| 873 | ICOSLG |
| 874 | TNFRSF8 |
| 875 | HSPA4 |
| 876 | SPINK5 |
| 877 | MIR31HG |
| 878 | CDC25B |
| 879 | PIP |
| 880 | CEACAM7 |
| 881 | GUCY2C |
| 882 | CXCR3 |
| 883 | JAG2 |
| 884 | CLDN23 |
| 885 | PTHLH |
| 886 | PYY |
| 887 | SMARCE1 |
| 888 | APEX1 |
| 889 | UGT1A1 |
| 890 | CD28 |
| 891 | PRKN |
| 892 | KISS1 |
| 893 | TNFRSF1A |
| 894 | TNFRSF10A |
| 895 | NR3C1 |
| 896 | WT1-AS |
| 897 | MIR34B |
| 898 | TNFRSF13C |
| 899 | ZNRD1 |
| 900 | AMH |
| 901 | CDKN2C |
| 902 | KRT10 |
| 903 | NOS3 |
| 904 | GSDMA |
| 905 | ANGPT1 |
| 906 | FRGCA |
| 907 | NAA15 |
| 908 | CRNN |
| 909 | FEZF1-AS1 |
| 910 | CTSL |
| 911 | CASC15 |
| 912 | ING1 |
| 913 | RAP1A |
| 914 | MBL2 |
| 915 | CXCR5 |
| 916 | NCOR2 |
| 917 | MIR96 |
| 918 | DMTF1 |
| 919 | GADD45G |
| 920 | HLA-DQB1 |
| 921 | HOXA13 |
| 922 | SNHG6 |
| 923 | NES |
| 924 | FER1L4 |
| 925 | WRAP53 |
| 926 | MIR16-2 |
| 927 | PLCE1 |
| 928 | IFNB1 |
| 929 | NPTN-IT1 |
| 930 | TG |
| 931 | APOE |
| 932 | SERPINB2 |
| 933 | UCHL1 |
| 934 | SOX9 |
| 935 | DRD4 |
| 936 | BDNF |
| 937 | MIR4435-2HG |
| 938 | FGF8 |
| 939 | GH1 |
| 940 | SPARC |
| 941 | FLG |
| 942 | PRLR |
| 943 | TMEM127 |
| 944 | DRD3 |
| 945 | CALR |
| 946 | MIR574 |
| 947 | H2AX |
| 948 | S100B |
| 949 | IL1A |
| 950 | ETV4 |
| 951 | HPGD |
| 952 | BGLAP |
| 953 | ANXA2 |
| 954 | PDGFA |
| 955 | ASXL1 |
| 956 | TRAF6 |
| 957 | CUBN |
| 958 | WIF1 |
| 959 | ETV6 |
| 960 | TRAF2 |
| 961 | LIPF |
| 962 | SELE |
| 963 | FOLH1 |
| 964 | LINC01234 |
| 965 | MIR30C1 |
| 966 | KLK6 |
| 967 | TFRC |
| 968 | TCF4 |
| 969 | STAT6 |
| 970 | MIR186 |
| 971 | MSR1 |
| 972 | KRT4 |
| 973 | INSR |
| 974 | SDC1 |
| 975 | GPER1 |
| 976 | B2M |
| 977 | ABHD11-AS1 |
| 978 | LOX |
| 979 | GALNT12 |
| 980 | YBX1 |
| 981 | PRKAA2 |
| 982 | GPT |
| 983 | GDF15 |
| 984 | AGAP2-AS1 |
| 985 | PLA2G4A |
| 986 | DUXAP9 |
| 987 | GPC3 |
| 988 | PIN1 |
| 989 | HLA-G |
| 990 | MIR92A1 |
| 991 | BCYRN1 |
| 992 | TNK2 |
| 993 | MDM4 |
| 994 | NTHL1 |
| 995 | KRT14 |
| 996 | PRKACA |
| 997 | MMP10 |
| 998 | MYH11 |
| 999 | MDK |
| 1000 | GLI2 |
| 1001 | S100A9 |
| 1002 | TRAF3 |
| 1003 | LINC00628 |
| 1004 | OR3A4P |
| 1005 | GHRH |
| 1006 | CCR5 |
| 1007 | LINC00052 |
| 1008 | RNASEL |
| 1009 | LINC01772 |
| 1010 | IL5 |
| 1011 | COL1A2 |
| 1012 | CALB2 |
| 1013 | ALPP |
| 1014 | ABCC3 |
| 1015 | ANO1 |
| 1016 | LGALS1 |
| 1017 | TMEM238L |
| 1018 | NPM1 |
| 1019 | IL13 |
| 1020 | MIR206 |
| 1021 | AXL |
| 1022 | RXRA |
| 1023 | TJP1 |
| 1024 | XBP1 |
| 1025 | GRN |
| 1026 | MTR |
| 1027 | TFAP2A |
| 1028 | CASP7 |
| 1029 | MIR103A1 |
| 1030 | MIR130B |
| 1031 | HSPA8 |
| 1032 | PML |
| 1033 | THRB |
| 1034 | DLEU1 |
| 1035 | MIR29B2 |
| 1036 | APC2 |
| 1037 | RELB |
| 1038 | MIR135A1 |
| 1039 | MS4A1 |
| 1040 | IGF2BP3 |
| 1041 | CEACAM3 |
| 1042 | IL12RB1 |
| 1043 | ITGAV |
| 1044 | MVP |
| 1045 | IL18 |
| 1046 | GZMB |
| 1047 | FOXA1 |
| 1048 | IRF4 |
| 1049 | HMGA1 |
| 1050 | IGFBP2 |
| 1051 | MSMB |
| 1052 | SETBP1 |
| 1053 | TDRG1 |
| 1054 | VWF |
| 1055 | MIR455 |
| 1056 | ELAC2 |
| 1057 | TMPRSS2 |
| 1058 | NRP1 |
| 1059 | MIR151A |
| 1060 | SDHAF2 |
| 1061 | PHF10 |
| 1062 | DROSHA |
| 1063 | DDR2 |
| 1064 | MACC1 |
| 1065 | VTRNA2-1 |
| 1066 | MIR361 |
| 1067 | SLCO1B1 |
| 1068 | PPP1R1B |
| 1069 | TBK1 |
| 1070 | EPRS1 |
| 1071 | PRF1 |
| 1072 | MIR202 |
| 1073 | CXCL5 |
| 1074 | XDH |
| 1075 | EBAG9 |
| 1076 | PLG |
| 1077 | PRDM16-DT |
| 1078 | LINC00901 |
| 1079 | SHBG |
| 1080 | DRD5 |
| 1081 | CCND3 |
| 1082 | IL15 |
| 1083 | COL18A1 |
| 1084 | MIR10A |
| 1085 | TLR1 |
| 1086 | PTPRT |
| 1087 | CEBPB |
| 1088 | SNHG8 |
| 1089 | PRSS21 |
| 1090 | PPP2R1B |
| 1091 | MAP3K1 |
| 1092 | IL4R |
| 1093 | EGFLAM-AS1 |
| 1094 | EIF4E |
| 1095 | CSF1 |
| 1096 | MIRLET7E |
| 1097 | PTGES |
| 1098 | RACK1 |
| 1099 | MIR512-1 |
| 1100 | HLA-DQA1 |
| 1101 | PRSS1 |
| 1102 | TBX1 |
| 1103 | SUMO1P3 |
| 1104 | MMP13 |
| 1105 | GATA4 |
| 1106 | MIR128-2 |
| 1107 | JAK1 |
| 1108 | KLLN |
| 1109 | RYR1 |
| 1110 | MIR218-1 |
| 1111 | KL |
| 1112 | LOC111589215 |
| 1113 | NORAD |
| 1114 | MIR320A |
| 1115 | HFE |
| 1116 | MIRLET7G |
| 1117 | MSLN |
| 1118 | NTRK2 |
| 1119 | PXN |
| 1120 | DCLK1 |
| 1121 | ERVH48-1 |
| 1122 | NSD1 |
| 1123 | CD46 |
| 1124 | MIR212 |
| 1125 | AICDA |
| 1126 | POLK |
| 1127 | FGF3 |
| 1128 | INHA |
| 1129 | CCKAR |
| 1130 | MIR196A2 |
| 1131 | MIR135B |
| 1132 | HDAC2 |
| 1133 | DAXX |
| 1134 | TXN |
| 1135 | NDRG1 |
| 1136 | NAT1 |
| 1137 | DRD2 |
| 1138 | MIR494 |
| 1139 | NGF |
| 1140 | GATA6-AS1 |
| 1141 | CYP27B1 |
| 1142 | HTR3A |
| 1143 | GAPDH |
| 1144 | MSTO2P |
| 1145 | MYH9 |
| 1146 | COL17A1 |
| 1147 | IKBKB |
| 1148 | HDAC4 |
| 1149 | ROCK1 |
| 1150 | MIR499A |
| 1151 | MIR330 |
| 1152 | MIRLET7C |
| 1153 | CD81 |
| 1154 | HAGLROS |
| 1155 | MIR542 |
| 1156 | SAG |
| 1157 | IL6R |
| 1158 | DDX41 |
| 1159 | KMT2C |
| 1160 | EPHA3 |
| 1161 | CYP2A6 |
| 1162 | LINC00941 |
| 1163 | CLU |
| 1164 | ILK |
| 1165 | CYP1A2 |
| 1166 | CD14 |
| 1167 | TCN1 |
| 1168 | MDC1-AS1 |
| 1169 | CIP2A |
| 1170 | SOCS1 |
| 1171 | FOXF1 |
| 1172 | LTA |
| 1173 | CD86 |
| 1174 | PTPN1 |
| 1175 | ENPP1 |
| 1176 | IGF2BP2 |
| 1177 | TRIP13 |
| 1178 | IFITM1 |
| 1179 | CDC73 |
| 1180 | PRKAA1 |
| 1181 | SOCS3 |
| 1182 | SQSTM1 |
| 1183 | IL2RB |
| 1184 | BCAR1 |
| 1185 | RAD51B |
| 1186 | DUXAP8 |
| 1187 | FANCD2 |
| 1188 | CXCL13 |
| 1189 | ENSG00000278769 |
| 1190 | ENO1 |
| 1191 | RPL34-AS1 |
| 1192 | CIB1 |
| 1193 | BNC2-AS1 |
| 1194 | MCM7 |
| 1195 | SFTA1P |
| 1196 | FADD |
| 1197 | LCN2 |
| 1198 | PTK2B |
| 1199 | DDIT3 |
| 1200 | CEBPA-DT |
| 1201 | LINC01006 |
| 1202 | VPS9D1-AS1 |
| 1203 | S100A1 |
| 1204 | FCGR2A |
| 1205 | AFDN-DT |
| 1206 | AKR7L |
| 1207 | NCRUPAR |
| 1208 | RPS6KA3 |
| 1209 | TRERNA1 |
| 1210 | HMMR |
| 1211 | LEPR |
| 1212 | OPCML |
| 1213 | LRP6 |
| 1214 | CDC25C |
| 1215 | CDC25A |
| 1216 | IDO1 |
| 1217 | MXI1 |
| 1218 | TKT |
| 1219 | SNCG |
| 1220 | MITF |
| 1221 | IGFBP1 |
| 1222 | CRH |
| 1223 | LUCAT1 |
| 1224 | CCN1 |
| 1225 | NR1H2 |
| 1226 | SLC22A18 |
| 1227 | KCNQ1 |
| 1228 | PRKCB |
| 1229 | AVP |
| 1230 | ARAF |
| 1231 | TNFSF13B |
| 1232 | CHRNA5 |
| 1233 | COL3A1 |
| 1234 | SH2D1A |
| 1235 | TRIM28 |
| 1236 | MIR181A2 |
| 1237 | EPHB4 |
| 1238 | MIR429 |
| 1239 | CDH3 |
| 1240 | BMP1 |
| 1241 | MAP3K20-AS1 |
| 1242 | TK1 |
| 1243 | LAMA2 |
| 1244 | MACC1-AS1 |
| 1245 | LINC00668 |
| 1246 | PMAIP1 |
| 1247 | EWSR1 |
| 1248 | DRAIC |
| 1249 | SATB2 |
| 1250 | ZMAT1 |
| 1251 | MIRLET7A3 |
| 1252 | INHBA-AS1 |
| 1253 | MIR675 |
| 1254 | MAPK10 |
| 1255 | TREX1 |
| 1256 | ARID4B |
| 1257 | MIR296 |
| 1258 | ITGA5 |
| 1259 | TNFRSF10A-AS1 |
| 1260 | MC1R |
| 1261 | SLC5A4-AS1 |
| 1262 | ST14 |
| 1263 | HSD11B2 |
| 1264 | PROS1 |
| 1265 | ACVRL1 |
| 1266 | TAC1 |
| 1267 | MIR32 |
| 1268 | IFNGR1 |
| 1269 | EREG |
| 1270 | STIM1 |
| 1271 | MT-CO1 |
| 1272 | TSG101 |
| 1273 | PRDM16 |
| 1274 | ADH7 |
| 1275 | POLB |
| 1276 | GSTM3 |
| 1277 | GAB1 |
| 1278 | HSPA1A |
| 1279 | MMP8 |
| 1280 | SLC7A11-AS1 |
| 1281 | AMACR |
| 1282 | CD9 |
| 1283 | WFS1 |
| 1284 | CSF1R |
| 1285 | SLC5A8 |
| 1286 | TOX3 |
| 1287 | MIR132 |
| 1288 | KRT7-AS |
| 1289 | AURKB |
| 1290 | PSMB8 |
| 1291 | PSEN2 |
| 1292 | TNNI3 |
| 1293 | CD5 |
| 1294 | ACP3 |
| 1295 | MT1DP |
| 1296 | MIR28 |
| 1297 | REST |
| 1298 | MYLK |
| 1299 | SFTPD |
| 1300 | ENAH |
| 1301 | LINC01612 |
| 1302 | LGR5 |
| 1303 | SSTR1 |
| 1304 | GSN |
| 1305 | EPO |
| 1306 | TCONS_00068220 |
| 1307 | DPP4 |
| 1308 | IRF5 |
| 1309 | BRD4 |
| 1310 | ATF6 |
| 1311 | IRS2 |
| 1312 | MIR491 |
| 1313 | CCNG1 |
| 1314 | FAP |
| 1315 | NKILA |
| 1316 | FAH |
| 1317 | TTR |
| 1318 | CBFB |
| 1319 | KCNJ5 |
| 1320 | DEFB4A |
| 1321 | FIP1L1 |
| 1322 | IL6ST |
| 1323 | LDHA |
| 1324 | TCF7L1 |
| 1325 | PDCD1LG2 |
| 1326 | TNFRSF11B |
| 1327 | ABCB11 |
| 1328 | GHR |
| 1329 | ITGB4 |
| 1330 | GNAQ |
| 1331 | MIR590 |
| 1332 | MTRR |
| 1333 | PAX8 |
| 1334 | ALPL |
| 1335 | F2R |
| 1336 | ZNRF3 |
| 1337 | RAC2 |
| 1338 | TUBB |
| 1339 | PRODH |
| 1340 | FOXE1 |
| 1341 | PTGER4 |
| 1342 | MIR148B |
| 1343 | BIRC7 |
| 1344 | FMR1 |
| 1345 | KIF1B |
| 1346 | HAVCR2 |
| 1347 | GATA2 |
| 1348 | PCAT29 |
| 1349 | PRKD1 |
| 1350 | SSX2 |
| 1351 | FZD4 |
| 1352 | ITGA3 |
| 1353 | CCL21 |
| 1354 | ID1 |
| 1355 | CHAT |
| 1356 | SNHG14 |
| 1357 | TGIF1 |
| 1358 | EFEMP1 |
| 1359 | PANTR1 |
| 1360 | LAMC2 |
| 1361 | TUSC3 |
| 1362 | SEMA4A |
| 1363 | GHSR |
| 1364 | CYP11B2 |
| 1365 | PNP |
| 1366 | PCNA-AS1 |
| 1367 | ENSG00000266990 |
| 1368 | FOXD2-AS1 |
| 1369 | INTS7 |
| 1370 | KLRK1 |
| 1371 | CASP1 |
| 1372 | IBSP |
| 1373 | ENSG00000225032 |
| 1374 | CAGE1 |
| 1375 | GRPR |
| 1376 | TRPV1 |
| 1377 | CYBB |
| 1378 | NMB |
| 1379 | FAT4 |
| 1380 | YWHAE |
| 1381 | SLC22A4 |
| 1382 | GLI3 |
| 1383 | MIR128-1 |
| 1384 | SERPINC1 |
| 1385 | LINC00460 |
| 1386 | SOX11 |
| 1387 | BUB3 |
| 1388 | CCL7 |
| 1389 | ACTA1 |
| 1390 | ANXA2P2 |
| 1391 | RN7SK |
| 1392 | THBD |
| 1393 | CYP3A5 |
| 1394 | ENSG00000249201 |
| 1395 | CYP24A1 |
| 1396 | CUL1 |
| 1397 | GPR65 |
| 1398 | TNFRSF13B |
| 1399 | LCK |
| 1400 | TIE1 |
| 1401 | S100P |
| 1402 | C1S |
| 1403 | SMO |
| 1404 | GYPA |
| 1405 | ITGA2 |
| 1406 | REN |
| 1407 | CASP2 |
| 1408 | ARID2 |
| 1409 | RAD54L |
| 1410 | SMIM31 |
| 1411 | RAG1 |
| 1412 | CP |
| 1413 | ACTG2 |
| 1414 | SFRP1 |
| 1415 | IGFBP5 |
| 1416 | KCNJ11 |
| 1417 | ENTPD1-AS1 |
| 1418 | PRKDC |
| 1419 | LTF |
| 1420 | FOSL1 |
| 1421 | LINC00572 |
| 1422 | BAG1 |
| 1423 | AQP3 |
| 1424 | TMEFF2 |
| 1425 | CISD2 |
| 1426 | LASP1 |
| 1427 | SLC11A1 |
| 1428 | IL1R1 |
| 1429 | POU2AF1 |
| 1430 | WFDC2 |
| 1431 | KRT18P55 |
| 1432 | LOC101929759 |
| 1433 | EDNRB |
| 1434 | PON1 |
| 1435 | MBD4 |
| 1436 | VIPR1 |
| 1437 | HP |
| 1438 | F5 |
| 1439 | ECM1 |
| 1440 | SULT1A1 |
| 1441 | PDPK1 |
| 1442 | SF3B1 |
| 1443 | ENSG00000232406 |
| 1444 | F7 |
| 1445 | TF |
| 1446 | HSD17B1 |
| 1447 | MDH2 |
| 1448 | GFAP |
| 1449 | HSP90B1 |
| 1450 | HK2 |
| 1451 | PTGER2 |
| 1452 | RHOB |
| 1453 | ARMC5 |
| 1454 | PLAT |
| 1455 | TSNAX-DISC1 |
| 1456 | ATF1 |
| 1457 | RUNX1T1 |
| 1458 | ECRG4 |
| 1459 | YWHAZ |
| 1460 | CCL3 |
| 1461 | ENSG00000285159 |
| 1462 | MIR328 |
| 1463 | HIC1 |
| 1464 | OXT |
| 1465 | RUNX1-IT1 |
| 1466 | MYLK-AS1 |
| 1467 | ZFHX4-AS1 |
| 1468 | SH3RF3-AS1 |
| 1469 | SUCLG2-AS1 |
| 1470 | A2M-AS1 |
| 1471 | LINC01097 |
| 1472 | ENSG00000224220 |
| 1473 | ENSG00000229717 |
| 1474 | LINC01856 |
| 1475 | LINC02461 |
| 1476 | ENSG00000253389 |
| 1477 | ENSG00000250406 |
| 1478 | ENSG00000277200 |
| 1479 | LOC105372446 |
| 1480 | NCOR1 |
| 1481 | DAB2IP |
| 1482 | HSD11B1 |
| 1483 | NOG |
| 1484 | MCM2 |
| 1485 | XAF1 |
| 1486 | PRSS8 |
| 1487 | CNC2 |
| 1488 | S100A2 |
| 1489 | CADM1 |
| 1490 | VCAM1 |
| 1491 | SFN |
| 1492 | CHKA |
| 1493 | UHRF1 |
| 1494 | PPARD |
| 1495 | CYP11B1 |
| 1496 | ADNP |
| 1497 | SFTPC |
| 1498 | PIK3C2A |
| 1499 | HABP2 |
| 1500 | CTSE |
| 1501 | PLCB1 |
| 1502 | DUOX2 |
| 1503 | MIR497 |
| 1504 | CALCR |
| 1505 | PIM1 |
| 1506 | AFAP1 |
| 1507 | LAMB3 |
| 1508 | APOB |
| 1509 | TDGF1 |
| 1510 | GPX1 |
| 1511 | MIR130A |
| 1512 | SPHK1 |
| 1513 | RARG |
| 1514 | RHOC |
| 1515 | HPRT1 |
| 1516 | MTUS1 |
| 1517 | ZAP70 |
| 1518 | ATP4B |
| 1519 | HSP90AB1 |
| 1520 | PIK3CD |
| 1521 | LGALS3BP |
| 1522 | ALCAM |
| 1523 | TCHP |
| 1524 | NEDD4 |
| 1525 | BCAR3 |
| 1526 | CCN4 |
| 1527 | SRSF2 |
| 1528 | RETN |
| 1529 | CYBA |
| 1530 | NGFR |
| 1531 | RRM2 |
| 1532 | RARS1 |
| 1533 | F2RL1 |
| 1534 | TCIM |
| 1535 | CALD1 |
| 1536 | MIR198 |
| 1537 | WWTR1 |
| 1538 | SBDS |
| 1539 | MLNR |
| 1540 | HDAC6 |
| 1541 | DNAH8 |
| 1542 | CCDC26 |
| 1543 | PDX1 |
| 1544 | TSHR |
| 1545 | CD7 |
| 1546 | TP53BP1 |
| 1547 | NAMPT |
| 1548 | MAD2L1 |
| 1549 | ABCC6 |
| 1550 | DEFB1 |
| 1551 | VTN |
| 1552 | PTP4A3 |
| 1553 | OSM |
| 1554 | NQO2 |
| 1555 | GTF2IRD1 |
| 1556 | BBC3 |
| 1557 | TGM2 |
| 1558 | TMPRSS11A |
| 1559 | ELK1 |
| 1560 | SRD5A1 |
| 1561 | NPPA |
| 1562 | EPHA4 |
| 1563 | SMARCC2 |
| 1564 | DKC1 |
| 1565 | MIR449A |
| 1566 | NUMA1 |
| 1567 | ADA2 |
| 1568 | SULF2 |
| 1569 | PODXL |
| 1570 | ITGA4 |
| 1571 | C5 |
| 1572 | PER1 |
| 1573 | NAPSA |
| 1574 | SMAD6 |
| 1575 | CHI3L1 |
| 1576 | NAB2 |
| 1577 | SPDEF |
| 1578 | FANCG |
| 1579 | HNF1A |
| 1580 | CXCR1 |
| 1581 | DDR1 |
| 1582 | CDK7 |
| 1583 | GAL |
| 1584 | CYP11A1 |
| 1585 | PSMB9 |
| 1586 | CD59 |
| 1587 | LPL |
| 1588 | SLC29A1 |
| 1589 | BNIP3 |
| 1590 | SPINT1 |
| 1591 | FANCM |
| 1592 | GNB5 |
| 1593 | GGT1 |
| 1594 | ROBO1 |
| 1595 | PES1 |
| 1596 | CARD11 |
| 1597 | NEDD9 |
| 1598 | CACNA1G |
| 1599 | MIR9-3 |
| 1600 | USH2A |
| 1601 | ALDOA |
| 1602 | MAP3K8 |
| 1603 | NUP214 |
| 1604 | CXCL10 |
| 1605 | RASA2 |
| 1606 | MYH7 |
| 1607 | TUBB3 |
| 1608 | MIR197 |
| 1609 | SLC6A4 |
| 1610 | PER2 |
| 1611 | HPN |
| 1612 | CD55 |
| 1613 | CD38 |
| 1614 | IL33 |
| 1615 | VTCN1 |
| 1616 | CCR3 |
| 1617 | MIR184 |
| 1618 | TPM2 |
| 1619 | C4A |
| 1620 | HES1 |
| 1621 | MAPK9 |
| 1622 | ALOX12 |
| 1623 | SLPI |
| 1624 | ERGIC1 |
| 1625 | SLC52A3 |
| 1626 | ATP7B |
| 1627 | TMPO |
| 1628 | SPN |
| 1629 | LYVE1 |
| 1630 | MIR377 |
| 1631 | JRK |
| 1632 | AVPR2 |
| 1633 | MIR124-3 |
| 1634 | ASCC1 |
| 1635 | DPH1 |
| 1636 | TCF3 |
| 1637 | NRXN1 |
| 1638 | ATP2B3 |
| 1639 | AKR1C3 |
| 1640 | CEP57 |
| 1641 | MMP12 |
| 1642 | IGHV4-38-2 |
| 1643 | KRT13 |
| 1644 | RPS27 |
| 1645 | SCG5 |
| 1646 | MIR26B |
| 1647 | CD22 |
| 1648 | PTPA |
| 1649 | ACTN4 |
| 1650 | PRKCE |
| 1651 | ABCC4 |
| 1652 | CDC6 |
| 1653 | AIFM1 |
| 1654 | AGR2 |
| 1655 | MECP2 |
| 1656 | SLC19A1 |
| 1657 | AGER |
| 1658 | MYH6 |
| 1659 | MIR33A |
| 1660 | MIR495 |
| 1661 | CLDN1 |
| 1662 | CHIC2 |
| 1663 | TPBG |
| 1664 | PTK6 |
| 1665 | FGF6 |
| 1666 | FKBP5 |
| 1667 | DELEC1 |
| 1668 | MIR133A1 |
| 1669 | PRKCI |
| 1670 | FBLN1 |
| 1671 | SULF1 |
| 1672 | PGF |
| 1673 | STK4 |
| 1674 | NPY |
| 1675 | H4-16 |
| 1676 | HNRNPK |
| 1677 | MIR30D |
| 1678 | MYCL |
| 1679 | PAX3 |
| 1680 | OCRL |
| 1681 | ADAM17 |
| 1682 | IL7R |
| 1683 | ADAR |
| 1684 | FLNA |
| 1685 | ATOH1 |
| 1686 | UBE3A |
| 1687 | NOL8 |
| 1688 | MICA |
| 1689 | NOD1 |
| 1690 | PRKCQ |
| 1691 | FANCI |
| 1692 | VAV3 |
| 1693 | ADAM12 |
| 1694 | APOA1 |
| 1695 | DVL3 |
| 1696 | AGT |
| 1697 | TPM3 |
| 1698 | SSTR5 |
| 1699 | MBP |
| 1700 | RPSA |
| 1701 | C5orf66-AS1 |
| 1702 | UFC1 |
| 1703 | TNFSF13 |
| 1704 | SSX1 |
| 1705 | RRM1 |
| 1706 | IL23A |
| 1707 | DDX53 |
| 1708 | WEE1 |
| 1709 | WNT5B |
| 1710 | IL12A |
| 1711 | BCR |
| 1712 | SELENBP1 |
| 1713 | DCK |
| 1714 | PIAS1 |
| 1715 | TNFRSF10C |
| 1716 | MT-ND4L |
| 1717 | RBX1 |
| 1718 | GSTA1 |
| 1719 | IST1 |
| 1720 | FZD6 |
| 1721 | FZD8 |
| 1722 | ADIPOR2 |
| 1723 | CYP27A1 |
| 1724 | GADD45B |
| 1725 | E2F4 |
| 1726 | RPS20 |
| 1727 | BRMS1 |
| 1728 | LINC-PINT |
| 1729 | CCL8 |
| 1730 | NUDT1 |
| 1731 | ADAM10 |
| 1732 | ICOS |
| 1733 | WNT11 |
| 1734 | MIR605 |
| 1735 | SYK |
| 1736 | CTH |
| 1737 | SLC12A3 |
| 1738 | MAPRE1 |
| 1739 | IFI16 |
| 1740 | CD2 |
| 1741 | LATS2 |
| 1742 | CSK |
| 1743 | MIR376A1 |
| 1744 | NPC1 |
| 1745 | TPM4 |
| 1746 | PAX6 |
| 1747 | CXADR |
| 1748 | LUC7L2 |
| 1749 | CEMIP |
| 1750 | STUB1 |
| 1751 | MTM1 |
| 1752 | MIR370 |
| 1753 | AKR1A1 |
| 1754 | HSPB8 |
| 1755 | IL12B |
| 1756 | XRCC4 |
| 1757 | IL1RAPL2 |
| 1758 | SAT1 |
| 1759 | HSF1 |
| 1760 | DEFA5 |
| 1761 | MADCAM1 |
| 1762 | BMPR1B |
| 1763 | ATF3 |
| 1764 | SEMA3B |
| 1765 | ZMYND10 |
| 1766 | TNFSF12 |
| 1767 | TCN2 |
| 1768 | CLPTM1L |
| 1769 | FUT3 |
| 1770 | SLC9A1 |
| 1771 | CT83 |
| 1772 | SLC7A5 |
| 1773 | DUSP5 |
| 1774 | CASC3 |
| 1775 | ATF4 |
| 1776 | CEBPA |
| 1777 | LIN28B |
| 1778 | FGF10 |
| 1779 | COL11A1 |
| 1780 | HDGF |
| 1781 | THPO |
| 1782 | TIAM1 |
| 1783 | HLA-C |
| 1784 | FCGR2B |
| 1785 | AMIGO2 |
| 1786 | FAM120A |
| 1787 | TNFRSF10D |
| 1788 | BMP7 |
| 1789 | HIP1R |
| 1790 | TAP1 |
| 1791 | IRF7 |
| 1792 | UBE2T |
| 1793 | IHH |
| 1794 | GSR |
| 1795 | ITGAX |
| 1796 | TNC |
| 1797 | PHEX |
| 1798 | CDC20 |
| 1799 | ACCS |
| 1800 | CD8A |
| 1801 | ANGPTL4 |
| 1802 | EIF2AK3 |
| 1803 | TRIM24 |
| 1804 | GJB1 |
| 1805 | SLC4A2 |
| 1806 | WNT10A |
| 1807 | MAPT |
| 1808 | CDH5 |
| 1809 | IGHMBP2 |
| 1810 | CASK |
| 1811 | MIR129-2 |
| 1812 | NCL |
| 1813 | RNASE3 |
| 1814 | ITPR1 |
| 1815 | CLDN6 |
| 1816 | ACTG1 |
| 1817 | ABCA1 |
| 1818 | RIPK1 |
| 1819 | PRNP |
| 1820 | CCAR2 |
| 1821 | RXRG |
| 1822 | TFE3 |
| 1823 | MIR371A |
| 1824 | PDGFD |
| 1825 | EHMT1 |
| 1826 | KAT5 |
| 1827 | PRDX1 |
| 1828 | PRRT2 |
| 1829 | HNRNPA2B1 |
| 1830 | LIF |
| 1831 | HLTF |
| 1832 | TGM3 |
| 1833 | SBF2-AS1 |
| 1834 | ADRB2 |
| 1835 | ELF3 |
| 1836 | DIRAS3 |
| 1837 | CD69 |
| 1838 | RBBP4 |
| 1839 | MIR490 |
| 1840 | CD27 |
| 1841 | NCOA1 |
| 1842 | FZD9 |
| 1843 | FZD3 |
| 1844 | PPY |
| 1845 | MIB2 |
| 1846 | RAB25 |
| 1847 | HMGCR |
| 1848 | ATP8B1 |
| 1849 | PBRM1 |
| 1850 | KDM6A |
| 1851 | IGFBP4 |
| 1852 | CARD10 |
| 1853 | EFNA1 |
| 1854 | AMN |
| 1855 | BLK |
| 1856 | PRMT7 |
| 1857 | MUC3A |
| 1858 | GUSB |
| 1859 | APP |
| 1860 | CTCF |
| 1861 | KMT2D |
| 1862 | LOXL2 |
| 1863 | EXO1 |
| 1864 | OTC |
| 1865 | MIR134 |
| 1866 | STAT4 |
| 1867 | KRT1 |
| 1868 | IL21 |
| 1869 | MELK |
| 1870 | C1R |
| 1871 | ADM |
| 1872 | ANXA3 |
| 1873 | BMPR2 |
| 1874 | RAPSN |
| 1875 | RBBP6 |
| 1876 | AKR1B10 |
| 1877 | TREM1 |
| 1878 | PAK4 |
| 1879 | SLC9A3R1 |
| 1880 | SCN1A |
| 1881 | SREBF1 |
| 1882 | MACF1 |
| 1883 | ENPP2 |
| 1884 | OLFM4 |
| 1885 | IL32 |
| 1886 | DRD1 |
| 1887 | CASP6 |
| 1888 | LACTB |
| 1889 | SMN1 |
| 1890 | FZD1 |
| 1891 | TNFRSF4 |
| 1892 | CYLD |
| 1893 | ASS1 |
| 1894 | PRC1 |
| 1895 | LTBP4 |
| 1896 | TERF2IP |
| 1897 | GJB2 |
| 1898 | PHLPP1 |
| 1899 | GLE1 |
| 1900 | PABPN1 |
| 1901 | EEF2 |
| 1902 | SELL |
| 1903 | SYNE1 |
| 1904 | HDAC3 |
| 1905 | HTR2A |
| 1906 | IGFBP7 |
| 1907 | NEK1 |
| 1908 | TXNRD1 |
| 1909 | DDX3X |
| 1910 | MT-CO2 |
| 1911 | MUC5B |
| 1912 | CLCNKB |
| 1913 | GPR68 |
| 1914 | DCN |
| 1915 | SUZ12 |
| 1916 | LPAR1 |
| 1917 | PTH1R |
| 1918 | SPOP |
| 1919 | FOLR1 |
| 1920 | S100A7 |
| 1921 | PTN |
| 1922 | ROCK2 |
| 1923 | ZNF217 |
| 1924 | RPRM |
| 1925 | PRMT1 |
| 1926 | GATA1 |
| 1927 | SFRP2 |
| 1928 | MIR372 |
| 1929 | NCF2 |
| 1930 | PTPRH |
| 1931 | PPARA |
| 1932 | WNT7A |
| 1933 | TNFRSF11A |
| 1934 | TFPI2 |
| 1935 | FOXA2 |
| 1936 | ATF2 |
| 1937 | KLK15 |
| 1938 | RRAS |
| 1939 | EEF1A1 |
| 1940 | ETS2 |
| 1941 | RASGRP1 |
| 1942 | CACNA1H |
| 1943 | MYBPC3 |
| 1944 | SIX1 |
| 1945 | CLCN5 |
| 1946 | GC |
| 1947 | SNCA |
| 1948 | NET1 |
| 1949 | GNB3 |
| 1950 | DVL2 |
| 1951 | LPAR2 |
| 1952 | SCN2A |
| 1953 | CA2 |
| 1954 | TRH |
| 1955 | SLC5A5 |
| 1956 | CTBP1 |
| 1957 | NR4A1 |
| 1958 | CCL20 |
| 1959 | CGA |
| 1960 | SELP |
| 1961 | PTGER3 |
| 1962 | FUT4 |
| 1963 | GPNMB |
| 1964 | CDR2 |
| 1965 | STC2 |
| 1966 | SLC6A3 |
| 1967 | MAGEL2 |
| 1968 | GPX3 |
| 1969 | NRP2 |
| 1970 | PEG10 |
| 1971 | ACKR3 |
| 1972 | IL17F |
| 1973 | EGFR-AS1 |
| 1974 | LIN28A |
| 1975 | CCL22 |
| 1976 | MIR502 |
| 1977 | IAPP |
| 1978 | ABCC12 |
| 1979 | SRGAP1 |
| 1980 | CSNK1D |
| 1981 | MAGED2 |
| 1982 | THBS2 |
| 1983 | SPINT2 |
| 1984 | FGF5 |
| 1985 | MIR125B2 |
| 1986 | EPHB6 |
| 1987 | RPS27A |
| 1988 | ROR1 |
| 1989 | PTCH2 |
| 1990 | COL4A2 |
| 1991 | IKBKE |
| 1992 | LAMC1 |
| 1993 | ROR2 |
| 1994 | HELLS |
| 1995 | PBOV1 |
| 1996 | CYP2C9 |
| 1997 | NDUFA13 |
| 1998 | ARSH |
| 1999 | SCN1B |
| 2000 | BCL3 |
| 2001 | CKS1B |
| 2002 | MIR337 |
| 2003 | PTPN13 |
| 2004 | RHOD |
| 2005 | TRPM8 |
| 2006 | MLXIPL |
| 2007 | TINF2 |
| 2008 | KLK11 |
| 2009 | B3GAT1 |
| 2010 | PRKD2 |
| 2011 | CD79B |
| 2012 | FLI1 |
| 2013 | GSTO2 |
| 2014 | TNS4 |
| 2015 | NOX1 |
| 2016 | UPK2 |
| 2017 | LOC110806263 |
| 2018 | HIPK2 |
| 2019 | PTPRJ |
| 2020 | PTP4A1 |
| 2021 | RTEL1 |
| 2022 | CLIC1 |
| 2023 | SLC34A1 |
| 2024 | HDAC5 |
| 2025 | NR5A1 |
| 2026 | VEGFB |
| 2027 | IGKC |
| 2028 | TRIM25 |
| 2029 | THBS4 |
| 2030 | ESS2 |
| 2031 | LPAR3 |
| 2032 | XAGE1A |
| 2033 | HERC2 |
| 2034 | ASPM |
| 2035 | DMD |
| 2036 | FZD10 |
| 2037 | SATB1 |
| 2038 | RAG2 |
| 2039 | PARK7 |
| 2040 | CDC27 |
| 2041 | TMPRSS4 |
| 2042 | TERF2 |
| 2043 | USP7 |
| 2044 | BAZ1B |
| 2045 | WNT4 |
| 2046 | CRKL |
| 2047 | LYST |
| 2048 | POLH |
| 2049 | STC1 |
| 2050 | NTN1 |
| 2051 | PIEZO2 |
| 2052 | VCP |
| 2053 | PRKCZ |
| 2054 | PRDX2 |
| 2055 | MORC2 |
| 2056 | CCAR1 |
| 2057 | CDR1-AS |
| 2058 | CPOX |
| 2059 | SLC5A7 |
| 2060 | CLOCK |
| 2061 | EPHA7 |
| 2062 | ZMPSTE24 |
| 2063 | TP73-AS1 |
| 2064 | GTF2I |
| 2065 | PRDM1 |
| 2066 | REG1A |
| 2067 | RCVRN |
| 2068 | DNAH1 |
| 2069 | CYP21A2 |
| 2070 | ABCA3 |
| 2071 | TUBB2A |
| 2072 | TXNRD2 |
| 2073 | NLRP7 |
| 2074 | SLC2A10 |
| 2075 | ANXA7 |
| 2076 | WASF2 |
| 2077 | SEC23B |
| 2078 | SOX10 |
| 2079 | AMFR |
| 2080 | LDLR |
| 2081 | NKX2-8 |
| 2082 | HSD17B3 |
| 2083 | SREBF2 |
| 2084 | MIR340 |
| 2085 | CNTNAP1 |
| 2086 | SALL4 |
| 2087 | PDE5A |
| 2088 | POLG |
| 2089 | CNR1 |
| 2090 | POLG2 |
| 2091 | SLC7A11 |
| 2092 | MFN2 |
| 2093 | KAT2B |
| 2094 | TET1 |
| 2095 | HECTD4 |
| 2096 | WNT7B |
| 2097 | SIRT3 |
| 2098 | SKI |
| 2099 | MIR138-1 |
| 2100 | LINC01554 |
| 2101 | TPO |
| 2102 | TPM1 |
| 2103 | BTNL2 |
| 2104 | EGLN3 |
| 2105 | MC2R |
| 2106 | INPPL1 |
| 2107 | LIFR |
| 2108 | MLF1 |
| 2109 | CD164 |
| 2110 | AMHR2 |
| 2111 | PLCG2 |
| 2112 | RAD52 |
| 2113 | BLCAP |
| 2114 | HOXA10 |
| 2115 | CAMK2G |
| 2116 | CX3CR1 |
| 2117 | GNA11 |
| 2118 | ADIPOR1 |
| 2119 | TP53BP2 |
| 2120 | ADAM9 |
| 2121 | IRF2BP2 |
| 2122 | UCN |
| 2123 | FUS |
| 2124 | KDM5B |
| 2125 | XAGE1B |
| 2126 | TRIM31 |
| 2127 | MIR218-2 |
| 2128 | PPIA |
| 2129 | TTN-AS1 |
| 2130 | CDCP1 |
| 2131 | MCAM |
| 2132 | AGO2 |
| 2133 | CASC8 |
| 2134 | F2RL3 |
| 2135 | MUC7 |
| 2136 | CSNK1A1 |
| 2137 | CCR2 |
| 2138 | CD276 |
| 2139 | PTPRG |
| 2140 | MAK |
| 2141 | HOXA9 |
| 2142 | HSPG2 |
| 2143 | GABRA1 |
| 2144 | PLA2G6 |
| 2145 | SMURF1 |
| 2146 | VSIG1 |
| 2147 | SASH1 |
| 2148 | MIR376C |
| 2149 | CAPN2 |
| 2150 | DDX5 |
| 2151 | SKP1 |
| 2152 | PLD2 |
| 2153 | ETV5 |
| 2154 | ITGAL |
| 2155 | TEAD1 |
| 2156 | ALDH7A1 |
| 2157 | MIR17HG |
| 2158 | TIMELESS |
| 2159 | MPL |
| 2160 | BIN1 |
| 2161 | STAG3 |
| 2162 | ABO |
| 2163 | SPINK7 |
| 2164 | MYB |
| 2165 | OPA3 |
| 2166 | PPARGC1A |
| 2167 | DSP |
| 2168 | IL22 |
| 2169 | APOBEC3B |
| 2170 | HK1 |
| 2171 | CKS2 |
| 2172 | FST |
| 2173 | THY1 |
| 2174 | SRF |
| 2175 | APOLD1 |
| 2176 | GATD3A |
| 2177 | FGF20 |
| 2178 | EHMT2 |
| 2179 | POU5F1B |
| 2180 | SPHK2 |
| 2181 | MSI1 |
| 2182 | DPF2 |
| 2183 | PRDX5 |
| 2184 | SMAD1 |
| 2185 | WASF3 |
| 2186 | IL13RA2 |
| 2187 | NDRG2 |
| 2188 | CHFR |
| 2189 | CD247 |
| 2190 | IGFBP6 |
| 2191 | HSPB2 |
| 2192 | NEU1 |
| 2193 | HNRNPA1 |
| 2194 | ITPR3 |
| 2195 | PRMT5 |
| 2196 | CCNB2 |
| 2197 | RBP4 |
| 2198 | GPRC5A |
| 2199 | NTSR1 |
| 2200 | MST1 |
| 2201 | WNT16 |
| 2202 | FAM3C |
| 2203 | NEB |
| 2204 | KRT16 |
| 2205 | SLC39A1 |
| 2206 | OPRM1 |
| 2207 | PRKCG |
| 2208 | STX11 |
| 2209 | ABI1 |
| 2210 | NUP107 |
| 2211 | BAMBI |
| 2212 | FEN1 |
| 2213 | FABP4 |
| 2214 | POLR2E |
| 2215 | ATG5 |
| 2216 | CAPN3 |
| 2217 | HTR4 |
| 2218 | FRAT1 |
| 2219 | TSPO |
| 2220 | IL16 |
| 2221 | MBD5 |
| 2222 | A2ML1 |
| 2223 | CDA |
| 2224 | UGT1A7 |
| 2225 | MIR625 |
| 2226 | IL23R |
| 2227 | CMIP |
| 2228 | TGFBR3 |
| 2229 | LYN |
| 2230 | CBLB |
| 2231 | SRA1 |
| 2232 | SLC17A5 |
| 2233 | TBX3 |
| 2234 | SPAG9 |
| 2235 | HOXB9 |
| 2236 | PORCN |
| 2237 | RALBP1 |
| 2238 | HOXA5 |
| 2239 | MAP1LC3A |
| 2240 | P2RY12 |
| 2241 | CCR4 |
| 2242 | BACH1 |
| 2243 | TFAP2C |
| 2244 | ECE1 |
| 2245 | SH3TC2 |
| 2246 | STARD13 |
| 2247 | LTO1 |
| 2248 | GLP1R |
| 2249 | FGF18 |
| 2250 | NR0B2 |
| 2251 | PROX1 |
| 2252 | KLK13 |
| 2253 | MAP2K7 |
| 2254 | ATP1A1 |
| 2255 | RAD17 |
| 2256 | TCOF1 |
| 2257 | FUCA1 |
| 2258 | KDM5A |
| 2259 | PTPN6 |
| 2260 | CAPN1 |
| 2261 | CBS |
| 2262 | MUCL1 |
| 2263 | BCL2L2 |
| 2264 | RBP1 |
| 2265 | UPF1 |
| 2266 | LOC110806262 |
| 2267 | TFG |
| 2268 | TACR1 |
| 2269 | PAGE4 |
| 2270 | CKB |
| 2271 | F10 |
| 2272 | CXCL9 |
| 2273 | DLST |
| 2274 | TLR7 |
| 2275 | F13A1 |
| 2276 | SCTR |
| 2277 | NCF4 |
| 2278 | CREB3L1 |
| 2279 | FDPS |
| 2280 | NEK2 |
| 2281 | MBD2 |
| 2282 | KLK8 |
| 2283 | EPB41L3 |
| 2284 | DEK |
| 2285 | RBM5 |
| 2286 | RFC4 |
| 2287 | FGF21 |
| 2288 | LAPTM4B |
| 2289 | TERF1 |
| 2290 | PNLIP |
| 2291 | MIR345 |
| 2292 | UBC |
| 2293 | YWHAQ |
| 2294 | AGTR2 |
| 2295 | SAPCD2 |
| 2296 | PSMD10 |
| 2297 | FLNB |
| 2298 | CD83 |
| 2299 | SKIV2L |
| 2300 | FPGS |
| 2301 | HSD3B2 |
| 2302 | PIAS3 |
| 2303 | CRK |
| 2304 | MIR326 |
| 2305 | PHLPP2 |
| 2306 | PIGR |
| 2307 | TLR10 |
| 2308 | PKD1 |
| 2309 | FOXC2 |
| 2310 | SLC16A1 |
| 2311 | IRF3 |
| 2312 | ALDH9A1 |
| 2313 | ST6GAL1 |
| 2314 | CDK5 |
| 2315 | AKAP13 |
| 2316 | SSTR3 |
| 2317 | SPRR3 |
| 2318 | STX1A |
| 2319 | FOXO4 |
| 2320 | LSP1 |
| 2321 | RICTOR |
| 2322 | G6PC |
| 2323 | RHO |
| 2324 | CD74 |
| 2325 | CD70 |
| 2326 | MIR216A |
| 2327 | RARRES1 |
| 2328 | RAN |
| 2329 | RASSF5 |
| 2330 | MSN |
| 2331 | NSD2 |
| 2332 | COPS5 |
| 2333 | RRP1B |
| 2334 | EIF3A |
| 2335 | EML4 |
| 2336 | FGF17 |
| 2337 | SLC35B2 |
| 2338 | CCR1 |
| 2339 | KIF15 |
| 2340 | RXRB |
| 2341 | SLC35C2 |
| 2342 | NR1I2 |
| 2343 | RHOBTB2 |
| 2344 | ARG1 |
| 2345 | COLCA1 |
| 2346 | MNT |
| 2347 | PEA15 |
| 2348 | CLCA1 |
| 2349 | ERCC8 |
| 2350 | RPL11 |
| 2351 | COLCA2 |
| 2352 | CDH11 |
| 2353 | RFC3 |
| 2354 | KISS1R |
| 2355 | GSTM2 |
| 2356 | MGAT5 |
| 2357 | PPP1R13L |
| 2358 | MIR503 |
| 2359 | AIF1 |
| 2360 | SLC25A11 |
| 2361 | ELOC |
| 2362 | FGA |
| 2363 | LRP1B |
| 2364 | MIR339 |
| 2365 | ASPSCR1 |
| 2366 | HEY1 |
| 2367 | EPHA1 |
| 2368 | EIF2AK2 |
| 2369 | CFLAR-AS1 |
| 2370 | CFP |
| 2371 | LMTK3 |
| 2372 | CSF3R |
| 2373 | CISH |
| 2374 | MIR381 |
| 2375 | LINC00365 |
| 2376 | TJP2 |
| 2377 | C20orf204 |
| 2378 | SRSF1 |
| 2379 | QKI |
| 2380 | CTNNA3 |
| 2381 | ANG |
| 2382 | MT2A |
| 2383 | LINC01194 |
| 2384 | PDK1 |
| 2385 | SLC45A3 |
| 2386 | CCL4 |
| 2387 | MAP3K5 |
| 2388 | RAD23B |
| 2389 | KIF20B |
| 2390 | PLK4 |
| 2391 | EEF1A2 |
| 2392 | SH2B3 |
| 2393 | BMP10 |
| 2394 | NNMT |
| 2395 | OCIAD1 |
| 2396 | NT5E |
| 2397 | BCAS2 |
| 2398 | AZGP1 |
| 2399 | CDK2AP1 |
| 2400 | TEF |
| 2401 | GLO1 |
| 2402 | ENPP7 |
| 2403 | LAMA3 |
| 2404 | BAG3 |
| 2405 | PITX2 |
| 2406 | CD68 |
| 2407 | MYBL2 |
| 2408 | CLIP2 |
| 2409 | SMARCA1 |
| 2410 | BTRC |
| 2411 | GNAI2 |
| 2412 | TACC3 |
| 2413 | POU3F3 |
| 2414 | SLC2A4 |
| 2415 | MIR663A |
| 2416 | ITGA9 |
| 2417 | MIR661 |
| 2418 | CDR1 |
| 2419 | CDK9 |
| 2420 | DACT1 |
| 2421 | PTPN12 |
| 2422 | MIEN1 |
| 2423 | HOPX |
| 2424 | ESRRG |
| 2425 | MIR199A2 |
| 2426 | SGK3 |
| 2427 | TSLP |
| 2428 | ZMIZ1 |
| 2429 | SUSD2 |
| 2430 | AKR1C1 |
| 2431 | TACC2 |
| 2432 | STARD3 |
| 2433 | CUL3 |
| 2434 | CFH |
| 2435 | FTO |
| 2436 | ADCY10 |
| 2437 | PBX1 |
| 2438 | ACVR1 |
| 2439 | NUPR1 |
| 2440 | LGI4 |
| 2441 | TMSB4X |
| 2442 | ACVR2A |
| 2443 | DIRC3 |
| 2444 | PRKAB1 |
| 2445 | PLCB4 |
| 2446 | HIP1 |
| 2447 | MRC1 |
| 2448 | KLF17 |
| 2449 | ACACA |
| 2450 | SMYD3 |
| 2451 | MIR506 |
| 2452 | IGLL5 |
| 2453 | COL4A3 |
| 2454 | PTGER1 |
| 2455 | SMAD9 |
| 2456 | NOVA1 |
| 2457 | CD63 |
| 2458 | FOXL2 |
| 2459 | TNFRSF17 |
| 2460 | ALYREF |
| 2461 | LRBA |
| 2462 | NR4A2 |
| 2463 | JUNB |
| 2464 | TACSTD2 |
| 2465 | SP3 |
| 2466 | ERN1 |
| 2467 | TBL1XR1 |
| 2468 | AQP4 |
| 2469 | CUX1 |
| 2470 | PELP1 |
| 2471 | CXCL14 |
| 2472 | ARSA |
| 2473 | GHRHR |
| 2474 | CALM1 |
| 2475 | CHRM3 |
| 2476 | MTHFD1 |
| 2477 | NNT-AS1 |
| 2478 | LIG3 |
| 2479 | MIR498 |
| 2480 | ADCYAP1 |
| 2481 | REV3L |
| 2482 | TNFRSF9 |
| 2483 | PRG2 |
| 2484 | TIA1 |
| 2485 | HHEX |
| 2486 | EIF3H |
| 2487 | UBR5 |
| 2488 | S100A14 |
| 2489 | GAS6 |
| 2490 | HSPA9 |
| 2491 | GATA5 |
| 2492 | LRP1 |
| 2493 | MIR425 |
| 2494 | ARNT |
| 2495 | FBP1 |
| 2496 | ACSL4 |
| 2497 | SDR9C7 |
| 2498 | RPS19 |
| 2499 | NR1H4 |
| 2500 | UBD |
| 2501 | FAM215A |
| 2502 | CIITA |
| 2503 | TGIF2 |
| 2504 | PARN |
| 2505 | ACD |
| 2506 | GLUL |
| 2507 | CRYAB |
| 2508 | SLC2A3 |
| 2509 | ACP1 |
| 2510 | VCAN |
| 2511 | PKHD1 |
| 2512 | ING5 |
| 2513 | CTBP2 |
| 2514 | MT-ND5 |
| 2515 | MAP2K3 |
| 2516 | INSL3 |
| 2517 | FANCE |
| 2518 | BTC |
| 2519 | DSG2 |
| 2520 | AGK |
| 2521 | GSDMB |
| 2522 | PDIA3 |
| 2523 | NR0B1 |
| 2524 | AKAP12 |
| 2525 | CAST |
| 2526 | RBBP8 |
| 2527 | GLS |
| 2528 | POU2F1 |
| 2529 | ID4 |
| 2530 | SCGB2A1 |
| 2531 | CCL18 |
| 2532 | MIR154 |
| 2533 | MICB |
| 2534 | MIR124-2 |
| 2535 | APPL1 |
| 2536 | PSEN1 |
| 2537 | RPA1 |
| 2538 | CEL |
| 2539 | SMPD1 |
| 2540 | NOB1 |
| 2541 | SLC2A2 |
| 2542 | CARTPT |
| 2543 | RBL2 |
| 2544 | CCL19 |
| 2545 | MARK1 |
| 2546 | RFC2 |
| 2547 | DDX21 |
| 2548 | VCL |
| 2549 | MIR1-1 |
| 2550 | SFTPB |
| 2551 | LYPD3 |
| 2552 | SAA1 |
| 2553 | ITGB2 |
| 2554 | ESRRA |
| 2555 | CUEDC2 |
| 2556 | PRDM5 |
| 2557 | MMP17 |
| 2558 | PHLDB1 |
| 2559 | CLEC3B |
| 2560 | MYH2 |
| 2561 | NR4A3 |
| 2562 | IL21R |
| 2563 | LINC02155 |
| 2564 | LINC02157 |
| 2565 | SLC4A1 |
| 2566 | TSPAN31 |
| 2567 | FNDC5 |
| 2568 | TRRAP |
| 2569 | IL3RA |
| 2570 | TRAF1 |
| 2571 | HTRA1 |
| 2572 | USP15 |
| 2573 | GRM1 |
| 2574 | CLDN9 |
| 2575 | UGT2B15 |
| 2576 | CHSY1 |
| 2577 | HTT |
| 2578 | RPS24 |
| 2579 | AQP1 |
| 2580 | NTPCR |
| 2581 | SOX17 |
| 2582 | PCGEM1 |
| 2583 | PTPN9 |
| 2584 | PMEPA1 |
| 2585 | TBL2 |
| 2586 | LIMA1 |
| 2587 | MIR7-1 |
| 2588 | TGM1 |
| 2589 | TNFSF15 |
| 2590 | CERNA2 |
| 2591 | ACHE |
| 2592 | HCRT |
| 2593 | CCL17 |
| 2594 | MIR374A |
| 2595 | CTCFL |
| 2596 | FAT1 |
| 2597 | IGF2BP1 |
| 2598 | CD151 |
| 2599 | MIR608 |
| 2600 | SULT2A1 |
| 2601 | EIF5A2 |
| 2602 | TMEM70 |
| 2603 | TXNIP |
| 2604 | NBAT1 |
| 2605 | GOLPH3 |
| 2606 | BARX1 |
| 2607 | MAP1LC3B |
| 2608 | ADSS1 |
| 2609 | MIR7-3 |
| 2610 | NCF1 |
| 2611 | ALOX15 |
| 2612 | HOXA11 |
| 2613 | PTPRU |
| 2614 | ARRB1 |
| 2615 | VAV1 |
| 2616 | MACROH2A1 |
| 2617 | PLAG1 |
| 2618 | BTG2 |
| 2619 | LDOC1 |
| 2620 | NAGLU |
| 2621 | P4HB |
| 2622 | CGB5 |
| 2623 | PCDH10 |
| 2624 | CKAP2 |
| 2625 | MUC12 |
| 2626 | GNB1 |
| 2627 | TSPAN8 |
| 2628 | PSMA7 |
| 2629 | CSTB |
| 2630 | KCNH1 |
| 2631 | AMPH |
| 2632 | TAGLN |
| 2633 | CGB3 |
| 2634 | DAB2 |
| 2635 | LRIG1 |
| 2636 | ZEB2-AS1 |
| 2637 | FXYD5 |
| 2638 | RHNO1 |
| 2639 | MIR363 |
| 2640 | GPX2 |
| 2641 | HYAL2 |
| 2642 | SCGB3A1 |
| 2643 | BCOR |
| 2644 | ZMYND8 |
| 2645 | BMX |
| 2646 | CD163 |
| 2647 | LZTR1 |
| 2648 | TRPV4 |
| 2649 | LOC110386951 |
| 2650 | MAP2K5 |
| 2651 | DSPP |
| 2652 | RPS14 |
| 2653 | CTSK |
| 2654 | NUCB2 |
| 2655 | SLIT2 |
| 2656 | YES1 |
| 2657 | ZFP36L1 |
| 2658 | TNNI2 |
| 2659 | RBPJ |
| 2660 | MIR708 |
| 2661 | SPRY2 |
| 2662 | CHAC1 |
| 2663 | SLC16A7 |
| 2664 | MIR136 |
| 2665 | TPMT |
| 2666 | FAM72D |
| 2667 | SGO1 |
| 2668 | RSF1 |
| 2669 | BCHE |
| 2670 | PLD1 |
| 2671 | VOPP1 |
| 2672 | TSG11 |
| 2673 | SLC22A1L |
| 2674 | SDCCAG8 |
| 2675 | SCLC1 |
| 2676 | SCHLAP1 |
| 2677 | RB1CC1 |
| 2678 | RAD54B |
| 2679 | RABL3 |
| 2680 | POLD1 |
| 2681 | PDGFRL |
| 2682 | PCAT4 |
| 2683 | PCAT2 |
| 2684 | PCAT19 |
| 2685 | PCAT18 |
| 2686 | PCAP |
| 2687 | PCA3 |
| 2688 | PALLD |
| 2689 | OVCAS1 |
| 2690 | OVCA2 |
| 2691 | ORAOV1 |
| 2692 | LNCR5 |
| 2693 | LNCR4 |
| 2694 | LNCR3 |
| 2695 | LNCR1 |
| 2696 | LCO |
| 2697 | KMHN1 |
| 2698 | KKLC1 |
| 2699 | HPCX2 |
| 2700 | HPCX1 |
| 2701 | HPCQTL19 |
| 2702 | HPC9 |
| 2703 | HPC7 |
| 2704 | HPC6 |
| 2705 | HPC5 |
| 2706 | HPC4 |
| 2707 | HPC3 |
| 2708 | HPC15 |
| 2709 | HPC14 |
| 2710 | HPC11 |
| 2711 | HPC10 |
| 2712 | HOXB13 |
| 2713 | HMPS1 |
| 2714 | HIC2 |
| 2715 | HEPN1 |
| 2716 | GAEC1 |
| 2717 | FALEC |
| 2718 | EIF4G1 |
| 2719 | EHBP1 |
| 2720 | DIRC1 |
| 2721 | CTAG3 |
| 2722 | CTAG1A |
| 2723 | CT47B1 |
| 2724 | CT47A9 |
| 2725 | CT47A8 |
| 2726 | CT47A7 |
| 2727 | CT47A6 |
| 2728 | CT47A5 |
| 2729 | CT47A4 |
| 2730 | CT47A3 |
| 2731 | CT47A2 |
| 2732 | CT47A11 |
| 2733 | CT47A10 |
| 2734 | CT47A1 |
| 2735 | CT45A6 |
| 2736 | CT45A5 |
| 2737 | CT45A4 |
| 2738 | CT45A3 |
| 2739 | CT45A2 |
| 2740 | CT45A1 |
| 2741 | CRCS9 |
| 2742 | CRCS8 |
| 2743 | CRCS7 |
| 2744 | CRCS6 |
| 2745 | CRCS5 |
| 2746 | CRCS2 |
| 2747 | CRCS11 |
| 2748 | CHRNA3 |
| 2749 | CASC21 |
| 2750 | CASC19 |
| 2751 | CASC11 |
| 2752 | CASC1 |
| 2753 | C2orf40 |
| 2754 | BCPR |
| 2755 | BASE |
| 2756 | ACVR1B |
| 2757 | WIP1 |
| 2758 | UVO |
| 2759 | UROC28 |
| 2760 | TXBP181 |
| 2761 | TNFSF6 |
| 2762 | TITF1 |
| 2763 | TIL4 |
| 2764 | TAOS1 |
| 2765 | STK15 |
| 2766 | SH2D3B |
| 2767 | SDH2 |
| 2768 | SCAL1 |
| 2769 | SAR1 |
| 2770 | RSTS2 |
| 2771 | RNS4 |
| 2772 | RNF124 |
| 2773 | RECA |
| 2774 | RAD53 |
| 2775 | RAD51L3 |
| 2776 | PTPG1 |
| 2777 | PSCP |
| 2778 | PRAD1 |
| 2779 | PNCA5 |
| 2780 | PMSL2 |
| 2781 | PLA2B |
| 2782 | PJS |
| 2783 | PHBP |
| 2784 | PGQTL2 |
| 2785 | PGL3 |
| 2786 | PGL1 |
| 2787 | PDGRL |
| 2788 | PCAT8 |
| 2789 | PCAT114 |
| 2790 | PCA2 |
| 2791 | PARK2 |
| 2792 | P53 |
| 2793 | ODCRCS |
| 2794 | NS7 |
| 2795 | NMOR2 |
| 2796 | NISBD2 |
| 2797 | NGL |
| 2798 | NEDSDV |
| 2799 | MYH |
| 2800 | MVCD4 |
| 2801 | MUC5 |
| 2802 | MTS1 |
| 2803 | MTCL1AS1 |
| 2804 | MRMV1 |
| 2805 | MMAC1 |
| 2806 | MCH5 |
| 2807 | MCH4 |
| 2808 | MAR |
| 2809 | MADH7 |
| 2810 | MADH4 |
| 2811 | LOC255313 |
| 2812 | LNCR2 |
| 2813 | LINC01458 |
| 2814 | LINC01245 |
| 2815 | LINC01244 |
| 2816 | LINC01190 |
| 2817 | LINC01092 |
| 2818 | LINC00990 |
| 2819 | LINC00912 |
| 2820 | LINC00860 |
| 2821 | LINC00340 |
| 2822 | LINC00178 |
| 2823 | LAS1 |
| 2824 | LAGE2A |
| 2825 | LAGE1 |
| 2826 | KRAS2 |
| 2827 | KIAA1304 |
| 2828 | KIAA0992 |
| 2829 | KIAA0903 |
| 2830 | KIAA0790 |
| 2831 | IRHOM2 |
| 2832 | HRG22 |
| 2833 | HR54 |
| 2834 | HPC2 |
| 2835 | HPC13 |
| 2836 | HNPCC7 |
| 2837 | HNPCC6 |
| 2838 | HLRCC |
| 2839 | GTBP |
| 2840 | GS |
| 2841 | GIF |
| 2842 | GDEP |
| 2843 | FRP1 |
| 2844 | FKHL15 |
| 2845 | FANCO |
| 2846 | FANCN |
| 2847 | FANCD1 |
| 2848 | FAL1 |
| 2849 | ESR |
| 2850 | EPHT3 |
| 2851 | EMS1 |
| 2852 | EIF4G |
| 2853 | ECOP |
| 2854 | DOC2 |
| 2855 | DOC1 |
| 2856 | DIA4 |
| 2857 | DHTR |
| 2858 | DEP1 |
| 2859 | DD3 |
| 2860 | CYP2A3 |
| 2861 | CXorf61 |
| 2862 | CWS6 |
| 2863 | CTAG1 |
| 2864 | CT47A13 |
| 2865 | CT45.6 |
| 2866 | CT45.5 |
| 2867 | CT45.4 |
| 2868 | CT45.3 |
| 2869 | CT45.2 |
| 2870 | CT45 |
| 2871 | CRCS12 |
| 2872 | CRCS10 |
| 2873 | CRCS1 |
| 2874 | CRAC1 |
| 2875 | COT |
| 2876 | COPEB |
| 2877 | COCA2 |
| 2878 | COCA1 |
| 2879 | CMM6 |
| 2880 | CLOVE |
| 2881 | CKN2 |
| 2882 | CCCAP |
| 2883 | CC1 |
| 2884 | CARLO5 |
| 2885 | BWSCR1A |
| 2886 | BUBR1 |
| 2887 | BHD |
| 2888 | BEK |
| 2889 | BCSG1 |
| 2890 | BCEI |
| 2891 | BC10 |
| 2892 | ATBF1 |
| 2893 | ATA |
| 2894 | ASV |
| 2895 | ALPS4 |
| 2896 | AIB1 |
| 2897 | ADH2 |
| 2898 | ACVRLK4 |
| 2899 | ACSTD1 |
| 2900 | ACH |
| 2901 | UC28 |
| 2902 | TROP1 |
| 2903 | TOC |
| 2904 | TNRC14 |
| 2905 | TITF2 |
| 2906 | TFM |
| 2907 | SSPCS |
| 2908 | SRC1 |
| 2909 | SLSN7 |
| 2910 | SDHIP |
| 2911 | SCKL1 |
| 2912 | RASK2 |
| 2913 | PRLTS |
| 2914 | PRCA1 |
| 2915 | PPP1R54 |
| 2916 | PNCA3 |
| 2917 | PNCA1 |
| 2918 | PLA2L |
| 2919 | PDJ |
| 2920 | PARK18 |
| 2921 | PAOD2 |
| 2922 | P16 |
| 2923 | NSP2 |
| 2924 | NS6 |
| 2925 | NMTC2 |
| 2926 | NMOR1 |
| 2927 | NKX2A |
| 2928 | NEU |
| 2929 | MVA1 |
| 2930 | MRMV2 |
| 2931 | MKHK2 |
| 2932 | MDPL |
| 2933 | MCUL1 |
| 2934 | MCAP |
| 2935 | LKB1 |
| 2936 | LINC00913 |
| 2937 | LFS1 |
| 2938 | LCAM |
| 2939 | KIAA1020 |
| 2940 | KIAA0203 |
| 2941 | JDVS |
| 2942 | IMPT1 |
| 2943 | IF |
| 2944 | HRAD54 |
| 2945 | HPC12 |
| 2946 | HNPCC5 |
| 2947 | HNPCC4 |
| 2948 | HNPCC2 |
| 2949 | HGPPS2 |
| 2950 | HGFAL |
| 2951 | GLM2 |
| 2952 | GASP |
| 2953 | FPC |
| 2954 | FILS |
| 2955 | FCC1 |
| 2956 | FANCJ |
| 2957 | EVR7 |
| 2958 | ESTRR |
| 2959 | EST |
| 2960 | DRT |
| 2961 | DPC4 |
| 2962 | DIRA |
| 2963 | CYP2A |
| 2964 | CUDR |
| 2965 | CRCS4 |
| 2966 | CRCS3 |
| 2967 | COXPD17 |
| 2968 | COFS1 |
| 2969 | CHK2 |
| 2970 | CFD1 |
| 2971 | CARLO7 |
| 2972 | CARLO6 |
| 2973 | CARLO4 |
| 2974 | CARLO2 |
| 2975 | CARLO1 |
| 2976 | CAPOK |
| 2977 | CAMEL |
| 2978 | BROVCA4 |
| 2979 | BROVCA3 |
| 2980 | BROVCA2 |
| 2981 | BROVCA1 |
| 2982 | BCL1 |
| 2983 | BCD1 |
| 2984 | AURORA2 |
| 2985 | AT1 |
| 2986 | APT1LG1 |
| 2987 | ALPS2B |
| 2988 | ALPS2 |
| 2989 | ALK4 |
| 2990 | AAT3 |
| 2991 | ZF9 |
| 2992 | TTF2 |
| 2993 | TTF1 |
| 2994 | TPL2 |
| 2995 | THC6 |
| 2996 | SBMA |
| 2997 | PNCA4 |
| 2998 | PGL4 |
| 2999 | P450C2A |
| 3000 | NS |
| 3001 | MOM1 |
| 3002 | MLM |
| 3003 | MFS2 |
| 3004 | MCM |
| 3005 | M4S1 |
| 3006 | JWS |
| 3007 | JIP |
| 3008 | IMAGEI |
| 3009 | HPC1 |
| 3010 | HNPCC1 |
| 3011 | HER2 |
| 3012 | GLM3 |
| 3013 | FSAP |
| 3014 | FMRD |
| 3015 | FCTCS |
| 3016 | FASL |
| 3017 | FANCR |
| 3018 | ERK |
| 3019 | ECAD |
| 3020 | DUP15q |
| 3021 | DUH1 |
| 3022 | CWS1 |
| 3023 | CSB |
| 3024 | CMNS |
| 3025 | CDS1 |
| 3026 | BTPS2 |
| 3027 | BTAK |
| 3028 | BCC7 |
| 3029 | BBS16 |
| 3030 | BAIPRCK |
| 3031 | TK14 |
| 3032 | PNCA2 |
| 3033 | PCBC |
| 3034 | NMTC5 |
| 3035 | NMTC4 |
| 3036 | NMTC1 |
| 3037 | NCMS |
| 3038 | MYHRS |
| 3039 | MIC18 |
| 3040 | MCMTC |
| 3041 | LFS2 |
| 3042 | LDS2 |
| 3043 | KD |
| 3044 | FANCS |
| 3045 | DESMD |
| 3046 | CMM2 |
| 3047 | CFC2 |
| 3048 | C15DUPq |
| 3049 | BMFS5 |
| 3050 | BCDS1 |
| 3051 | ARMD5 |
| 3052 | ARK1 |
| 3053 | ALPS1B |
| 3054 | UVSS1 |
| 3055 | STK6 |
| 3056 | SMAX1 |
| 3057 | RALD |
| 3058 | DIAR5 |
| 3059 | CWS5 |
| 3060 | CAPB |
| 3061 | BBDS |
| 3062 | POF11 |
| 3063 | OES |
| 3064 | HYSP1 |
| 3065 | HNPCC8 |
| 3066 | CLAPO |
| 3067 | BDPLT22 |
| 3068 | AIK |
| 3069 | ZBTB20 |
| 3070 | ARL6IP5 |
| 3071 | FSD1 |
| 3072 | MTSS1 |
| 3073 | PYCARD |
| 3074 | CLCN3 |
| 3075 | PDHA1 |
| 3076 | RORA |
| 3077 | MIA |
| 3078 | SCRN1 |
| 3079 | EBI3 |
| 3080 | LRRC3B |
| 3081 | DCBLD2 |
| 3082 | CST1 |
| 3083 | ECHS1 |
| 3084 | FGG |
| 3085 | ICAM2 |
| 3086 | FADS1 |
| 3087 | MSX1 |
| 3088 | COPS7A |
| 3089 | HIKESHI |
| 3090 | NBAS |
| 3091 | PLAGL1 |
| 3092 | RGS2 |
| 3093 | RPS6 |
| 3094 | SLC1A2 |
| 3095 | SPZ1 |
| 3096 | MIR22HG |
| 3097 | SLC16A3 |
| 3098 | TRAP1 |
| 3099 | CDKN2D |
| 3100 | CNPY2 |
| 3101 | NOP56 |
| 3102 | CCT7 |
| 3103 | CTSC |
| 3104 | CLN3 |
| 3105 | PLIN2 |
| 3106 | MBD3L2 |
| 3107 | CTNNA2 |
| 3108 | ADRB1 |
| 3109 | DDB1 |
| 3110 | DPAGT1 |
| 3111 | ALDH1A3 |
| 3112 | FKBP2 |
| 3113 | ALDOB |
| 3114 | FAM168A |
| 3115 | BOP1 |
| 3116 | ATP6V0D2 |
| 3117 | FYN |
| 3118 | SERBP1 |
| 3119 | ACAD8 |
| 3120 | SNX5 |
| 3121 | FILIP1 |
| 3122 | PRPF19 |
| 3123 | MRPS18B |
| 3124 | MRPL13 |
| 3125 | ABT1 |
| 3126 | CPSF1 |
| 3127 | HNRNPL |
| 3128 | HOXA2 |
| 3129 | HTR1A |
| 3130 | IDH3B |
| 3131 | M6PR |
| 3132 | MX1 |
| 3133 | NDUFA2 |
| 3134 | NDUFS1 |
| 3135 | NDUFV1 |
| 3136 | PA2G4 |
| 3137 | UBXN1 |
| 3138 | ZNF593 |
| 3139 | GMPR2 |
| 3140 | POLR3K |
| 3141 | PGAM1 |
| 3142 | PTOV1 |
| 3143 | PPIC |
| 3144 | PPP2R1A |
| 3145 | PREP |
| 3146 | NAXD |
| 3147 | EXOSC5 |
| 3148 | RANBP10 |
| 3149 | PTPRF |
| 3150 | RAD23A |
| 3151 | HRH4 |
| 3152 | RPL13 |
| 3153 | RPL18 |
| 3154 | RPS15 |
| 3155 | RPS21 |
| 3156 | RPS26 |
| 3157 | BLVRB |
| 3158 | MRPS11 |
| 3159 | SNRPB |
| 3160 | SPRR2A |
| 3161 | CA1 |
| 3162 | ZNF177 |
| 3163 | EPHX3 |
| 3164 | PUS1 |
| 3165 | ULBP2 |
| 3166 | TAF15 |
| 3167 | URM1 |
| 3168 | SYMPK |
| 3169 | ARFGAP2 |
| 3170 | ZNF559 |
| 3171 | TUBA1C |
| 3172 | ITGA8 |
| 3173 | CST7 |
| 3174 | PLPP1 |
| 3175 | TNFSF9 |
| 3176 | SUCLG1 |
| 3177 | FCGBP |
| 3178 | ZNF160 |
| 3179 | RRP9 |
| 3180 | WDR46 |
| 3181 | TMEM63A |
| 3182 | SMR3B |
| 3183 | SLCO6A1 |
| 3184 | TIMM8A |
| 3185 | ELAVL2 |
| 3186 | TBC1D9 |
| 3187 | HPGDS |
| 3188 | REM1 |
| 3189 | GSTK1 |
| 3190 | COX2 |
| 3191 | SGCB |
| 3192 | LILRB1 |
| 3193 | TOB1 |
| 3194 | TMED7-TICAM2 |
| 3195 | LRPPRC |
| 3196 | ZNRD2 |
| 3197 | DCTN6 |
| 3198 | SLC12A9 |
| 3199 | PSMD9 |
| 3200 | ANGPTL2 |
| 3201 | ARHGDIB |
| 3202 | TNFAIP8L2 |
| 3203 | HSPB3 |
| 3204 | KLRC4-KLRK1 |
| 3205 | KLF2 |
| 3206 | CXCR6 |
| 3207 | KLF8 |
| 3208 | FOXK1 |
| 3209 | FRZB |
| 3210 | GABPA |
| 3211 | PRPF31 |
| 3212 | RNF180 |
| 3213 | C17orf97 |
| 3214 | LMX1A |
| 3215 | NOX4 |
| 3216 | GDE1 |
| 3217 | METTL3 |
| 3218 | CHPT1 |
| 3219 | TEAD4 |
| 3220 | XPO1 |
| 3221 | DHDDS |
| 3222 | ARHGAP24 |
| 3223 | RASAL1 |
| 3224 | FOXQ1 |
| 3225 | PTK |

**Table S3.** Gene function annotation

| Category | Term | Count | % | PValue | Genes |
| --- | --- | --- | --- | --- | --- |
| BP | positive regulation of transcription from RNA polymerase II promoter | 45 | 17.17557 | 1.57E-10 | PPARA, HRAS, TNF, KMT2A, THRB, PPARG, TLR4, NR3C1, RORA, TLR9, CTNNB1, AKT1, PGR, KDM1A, VDR, APP, SERPINE1, TOP2A, CHUK, PIK3R1, NR1H4, EGFR, AR, ESRRA, NOS1, CTBP2, RELA, MET, ESR1, CDK9, STAT3, AHR, HDAC3, RPS6KA3, ADRB2, EP300, MAPK14, GSK3B, CD81, VEGFA, IKBKG, NFE2L2, PARP1, IKBKB, IL2 |
| BP | signal transduction | 42 | 16.03053 | 5.73E-07 | HRAS, PGF, ERBB2, PPARG, CXCR2, NR3C1, KIT, SRC, EDNRA, AKT1, PGR, VDR, IGF1R, CSNK2A1, PTGES, PTK2B, PIK3R1, NR1H4, EGFR, AR, RET, HSP90AA1, ROCK1, MAP2K1, PIK3CB, MET, PIK3CD, ESR1, ESR2, MAPK10, PRKCE, CDK4, STAT3, DAPK1, MAPK1, CD38, RPS6KA3, MAPK14, PDE5A, PDGFRB, MTOR, PLAU |
| BP | response to drug | 40 | 15.26718 | 9.91E-25 | LDHA, PTGS2, PGF, MGMT, SLC6A4, PPARG, PNP, SRC, ADA, CTNNB1, TYMS, TOP1, ARG1, CASP3, BCHE, PTK2B, SRD5A1, APEX1, DNMT3B, CHUK, CDK1, RET, HSP90AA1, CYP1A1, RELA, CDK9, ABCB1, CDK4, STAT3, ABCG2, CCNB1, CD38, CCND1, CA9, FYN, LCK, HSD11B2, ADAM17, MDM2, ABCC1 |
| BP | negative regulation of apoptotic process | 40 | 15.26718 | 2.07E-18 | PPARD, MCL1, MMP9, MGMT, AURKA, PDCD4, SRC, MIF, AKT1, EDNRB, IGF1R, PTK2, CASP3, PTK2B, GLO1, HSPA5, NQO1, PIK3R1, NR1H4, EGFR, CDK1, BRAF, RELA, PIM1, STAT3, KDR, CD38, HDAC3, RPS6KA3, PLK1, GSK3B, VEGFA, MPO, PDGFRB, MDM2, NGFR, IKBKB, EIF2AK2, IL2, ALOX12 |
| BP | oxidation-reduction process | 40 | 15.26718 | 1.67E-14 | XDH, CYP3A4, LDHA, CYP1B1, CYP2C19, PTGS2, HMGCR, HSD17B1, PTGS1, TH, EGLN3, AKR1C3, KDM1A, GSTK1, FASN, SRD5A1, HSD17B3, NOS2, NQO1, APEX1, AKR1C1, NQO2, CYP19A1, NOX4, CTBP2, NOS1, CYP1A1, CYP2C9, CYP1A2, ALOX15, CYP17A1, G6PD, DHFR, AKR1B10, HSD11B1, KDM4C, MPO, HSD11B2, ALOX5, DPYD |
| BP | protein phosphorylation | 39 | 14.8855 | 1.51E-17 | ERBB2, AURKA, AURKB, BTK, AKT1, CCNE1, APP, CSNK2A1, PTK2B, PIK3CA, CHUK, PIK3R1, SYK, PRKCA, PIK3CG, RET, ROCK1, BRAF, PIK3CD, PIM1, CDK9, CDK6, MAPK10, PRKCE, CDK4, DAPK1, MAPK1, CCND1, RPS6KA3, FYN, PLK1, GSK3B, LCK, ERN1, JAK3, MTOR, EIF2AK2, IKBKB, IGFBP3 |
| BP | positive regulation of cell proliferation | 36 | 13.74046 | 8.80E-15 | AVPR2, HRAS, PGF, CXCR2, KIT, ITGB1, AKR1C3, EDNRB, IGF1R, PTK2, CSNK2A1, HTR1A, PTK2B, DPP4, EGFR, PTPN6, AR, FLT1, FLT3, RELA, CDK4, STAT3, CDK2, KDR, CAPN1, MAPK1, F2, CD81, VEGFA, PDGFRA, KDM4C, ADAM17, PDGFRB, MDM2, IL2, ALOX12 |
| BP | negative regulation of transcription from RNA polymerase II promoter | 30 | 11.45038 | 2.52E-06 | PPARA, PPARD, TNF, THRB, EZH2, PPARG, AURKB, CTNNB1, EDNRB, VDR, KDM1A, DNMT3B, NR1H4, ESRRA, CTBP2, EHMT1, RELA, ELANE, ESR1, ESR2, EHMT2, STAT3, CCND1, HDAC3, EP300, PLK1, VEGFA, MDM2, DNMT1, PARP1 |
| BP | positive regulation of gene expression | 28 | 10.68702 | 6.33E-15 | AVPR2, HRAS, PPARD, TNF, ERBB2, SLC6A4, TLR4, HSPA1A, KIT, TLR9, VDR, DNMT3B, GBA, CDK1, AR, MAP2K1, BRAF, PIK3CB, PIK3CD, CDK6, STAT3, MAPK14, VEGFA, MDM2, DNMT1, MTOR, NFE2L2, ALOX12 |
| BP | cell proliferation | 26 | 9.923664 | 5.58E-10 | HRAS, PPARD, ACHE, ERBB2, AURKB, SRC, MIF, EDNRA, AKT1, KDM1A, TYMS, HTR1A, SYK, EGFR, PTPN6, CDK1, AR, CYP1A1, MET, PIM1, CDK9, CDK5, CDC25A, STAT3, PLK1, CD81 |
| BP | inflammatory response | 26 | 9.923664 | 1.15E-09 | TNF, PTGS2, TACR1, PTGS1, CXCR2, TLR4, KIT, MIF, TLR9, AKT1, NR1H4, CHUK, SYK, PIK3CG, NOX4, PTGER1, PTGER2, PTGER3, RELA, PIK3CD, POLB, ALOX15, IKBKG, NGFR, NFE2L2, IKBKB |
| BP | response to hypoxia | 25 | 9.541985 | 2.01E-16 | NOX4, PPARA, LDHA, NOS1, CYP1A1, PGF, SLC6A4, TH, EGLN3, MMP14, MMP2, ADA, EDNRA, CD38, CASP3, EP300, CA9, PTK2B, VEGFA, HSD11B2, ADAM17, NOS2, DNMT3B, DPP4, PLAU |
| BP | proteolysis | 24 | 9.160305 | 3.43E-06 | PREP, MMP9, ELANE, MMP8, PRSS1, MMP7, MME, ANPEP, MMP3, MMP14, MMP2, MMP13, PLG, MMP1, MMP12, CAPN1, CASP3, FOLH1, REN, F2, ADAM17, CTSD, DPP4, PLAU |
| BP | protein autophosphorylation | 23 | 8.778626 | 2.60E-14 | EGFR, FLT1, FLT3, ERBB2, PIM1, AURKA, KIT, AURKB, CDK5, SRC, KDR, DAPK1, AKT1, IGF1R, PTK2, PTK2B, GSK3B, ERN1, PDGFRA, PDGFRB, MTOR, EIF2AK2, SYK |
| BP | positive regulation of cell migration | 23 | 8.778626 | 1.07E-13 | PRKCA, EGFR, HRAS, RET, F10, FLT1, PIK3CD, KIT, MMP14, KDR, IGF1R, MAPK1, PTK2, PTK2B, F3, VEGFA, PDGFRA, PDGFRB, ADAM17, HSPA5, PLAU, PIK3R1, ALOX12 |
| BP | positive regulation of transcription, DNA-templated | 22 | 8.396947 | 5.44E-05 | PPARA, AR, RET, PPARD, TNF, NOS1, MAP2K1, KMT2A, RELA, PPARG, ESR1, RORA, ESR2, SRC, AHR, CDK2, STAT3, CTNNB1, CCNE1, MAPK1, CD38, IKBKB |
| BP | leukocyte migration | 21 | 8.015267 | 3.12E-15 | PTPN6, HRAS, ROCK1, GRB2, PIK3CB, MMP9, ITGA4, ITGB3, ITGB1, SLC7A5, MMP1, SRC, MIF, NRAS, SLC16A1, PPIA, FYN, LCK, F2, PIK3CA, PIK3R1 |
| BP | negative regulation of cell proliferation | 21 | 8.015267 | 3.83E-06 | NOX4, OPRM1, PTPN6, AR, HRAS, CYP1B1, CTBP2, MAP2K1, PTGS2, KMT2A, CDK6, PLG, STAT3, CTNNB1, VDR, SSTR5, PTGES, BCHE, PTK2B, EIF2AK2, IGFBP3 |
| BP | peptidyl-tyrosine phosphorylation | 20 | 7.633588 | 2.73E-12 | EGFR, PTPN6, RET, FLT1, HSP90AA1, MAP2K1, FLT3, ERBB2, MET, KIT, SRC, KDR, BTK, PTK2, FYN, PTK2B, PDGFRA, PDGFRB, JAK3, EIF2AK2 |
| BP | positive regulation of ERK1 and ERK2 cascade | 20 | 7.633588 | 3.01E-11 | PRKCA, NOX4, OPRM1, EGFR, HRAS, TNF, BRAF, MAP2K1, HMGCR, TLR4, SRC, KDR, MIF, ALOX15, PTK2B, VEGFA, PLA2G2A, PDGFRA, PDGFRB, NQO2 |
| BP | innate immune response | 20 | 7.633588 | 4.33E-05 | PIK3CG, PPARG, PIK3CD, TLR4, SRC, BTK, TLR9, MIF, PTK2, APP, FYN, PTK2B, LCK, IKBKG, JAK3, EIF2AK2, IKBKB, CHUK, NR1H4, SYK |
| BP | negative regulation of transcription, DNA-templated | 20 | 7.633588 | 2.97E-04 | PPARD, TNF, EHMT1, CTBP2, RELA, EZH2, PPARG, CDK5, PDCD4, SRC, AHR, CTNNB1, KDM1A, VDR, CD38, HDAC3, FABP4, MDM2, HSPA8, HDAC6 |
| BP | apoptotic process | 20 | 7.633588 | 0.001376 | PTPN6, CDK1, PPARD, KMT2A, PIM1, EGLN3, NR3C1, PRKCE, PDCD4, AHR, DAPK1, MAPK1, CASP3, RPS6KA3, CSNK2A1, EP300, PTK2B, MAPK14, IKBKG, IGFBP3 |
| BP | response to lipopolysaccharide | 19 | 7.251908 | 8.46E-11 | ALPL, OPRM1, PTGER1, PTGER2, PTGS2, CYP1A1, PTGER4, ELANE, TH, TLR4, CYP1A2, CASP3, RPS6KA3, PTGES, REN, MPO, ADAM17, NGFR, CHUK |
| BP | aging | 19 | 7.251908 | 9.35E-11 | CYP1A1, GRB2, HMGCR, RELA, MMP7, POLB, AURKB, ADA, STAT3, SIRT3, AKT1, TYMS, EDNRB, ARG1, MPO, NFE2L2, NQO1, APEX1, ALOX12 |
| BP | regulation of cell proliferation | 19 | 7.251908 | 6.20E-10 | ESRRA, PTGER2, TNF, BRAF, PTGS1, EZH2, EGLN3, CHEK1, KIT, SRC, BTK, PTK2, FYN, PTK2B, LCK, DNMT1, NOS2, NGFR, PLAU |
| BP | angiogenesis | 19 | 7.251908 | 1.23E-08 | PRKCA, PIK3CG, CYP1B1, FLT1, PTGS2, PGF, ANPEP, RORA, MMP14, MMP2, KDR, PTK2, PTK2B, MAPK14, SERPINE1, VEGFA, PIK3CA, SETD2, SYK |
| BP | MAPK cascade | 19 | 7.251908 | 1.46E-07 | EGFR, HRAS, RET, TNF, BRAF, MAP2K1, GRB2, ERBB2, KIT, NRAS, MAPK1, PTK2, FYN, PTK2B, PDGFRA, PDGFRB, JAK3, CALM1, IL2 |
| BP | cell surface receptor signaling pathway | 18 | 6.870229 | 1.34E-06 | EGFR, PTPRC, HRAS, ERBB2, TACR1, MET, CXCR2, MIF, EDNRB, PRMT1, ADRB2, PTK2B, MAPK14, F2, GIPR, CD81, CDA, GLP1R |
| BP | phosphatidylinositol-mediated signaling | 17 | 6.48855 | 7.32E-12 | EGFR, PIK3CG, GRB2, PIK3CB, ERBB2, PIK3CD, KIT, AKT1, IGF1R, FYN, LCK, RHOA, PDGFRA, PIK3CA, PDGFRB, MTOR, PIK3R1 |
| BP | positive regulation of MAP kinase activity | 16 | 6.10687 | 1.02E-14 | NOX4, PIK3CG, EGFR, HRAS, TNF, FLT1, FLT3, ERBB2, EZH2, ELANE, KIT, SRC, MIF, VEGFA, PDE5A, PDGFRB |
| BP | vascular endothelial growth factor receptor signaling pathway | 16 | 6.10687 | 2.48E-13 | PTK2, FLT1, HSP90AA1, ROCK1, PTK2B, FYN, PIK3CB, PGF, MAPK14, VEGFA, RHOA, PIK3CA, ITGB3, PIK3R1, SRC, KDR |
| BP | platelet activation | 16 | 6.10687 | 2.84E-10 | PRKCA, PIK3CG, PTPN6, PIK3CB, ITGB3, PRKCE, SRC, AKT1, MAPK1, FYN, F2, LCK, RHOA, PIK3CA, PIK3R1, SYK |
| BP | positive regulation of protein phosphorylation | 16 | 6.10687 | 1.17E-09 | EGFR, HRAS, TNF, ERBB2, MMP9, ITGB3, KDR, AKT1, EDNRB, PTK2, HDAC3, CCND1, F2, VEGFA, ADAM17, MTOR |
| BP | positive regulation of cytosolic calcium ion concentration | 16 | 6.10687 | 2.49E-09 | EDNRA, OPRM1, PIK3CG, EDNRB, PTGER1, CD38, PTGER2, PTGER3, PTGER4, PTK2B, GIPR, PDGFRA, ESR1, CXCR2, GLP1R, IL2 |
| BP | viral process | 16 | 6.10687 | 6.89E-05 | GRB2, FDPS, STAT3, MMP1, KDR, TOP1, MAPK1, EP300, FYN, LCK, RHOA, PDGFRA, MDM2, HSPA8, PIK3R1, SYK |
| BP | response to toxic substance | 15 | 5.725191 | 4.58E-11 | CDK1, CYP1B1, MGMT, SLC6A4, CDK4, AHR, MAPK1, TYMS, MDM2, PDGFRB, EIF2AK2, NQO1, DNMT3B, CHUK, HDAC6 |
| BP | response to estradiol | 15 | 5.725191 | 1.19E-10 | ESRRA, PTGS2, EZH2, TH, SLC6A4, ESR1, CYP1A2, STAT3, CTNNB1, CD38, CASP3, CCND1, PDGFRB, NQO1, DNMT3B |
| BP | Fc-epsilon receptor signaling pathway | 15 | 5.725191 | 7.29E-07 | HRAS, GRB2, PIK3CB, RELA, MAPK10, BTK, NRAS, MAPK1, IKBKG, PIK3CA, IKBKB, PIK3R1, CHUK, SYK, CALM1 |
| BP | regulation of phosphatidylinositol 3-kinase signaling | 14 | 5.343511 | 1.96E-10 | EGFR, GRB2, PIK3CB, ERBB2, PIK3CD, KIT, AKT1, MAPK1, FYN, LCK, PDGFRA, PIK3CA, PDGFRB, PIK3R1 |
| BP | transmembrane receptor protein tyrosine kinase signaling pathway | 14 | 5.343511 | 2.81E-09 | EGFR, RET, FLT1, PIK3CB, FLT3, ERBB2, MET, KIT, KDR, BTK, IGF1R, FYN, LCK, SYK |
| BP | response to ethanol | 14 | 5.343511 | 8.58E-09 | CDK1, HMGCR, TH, MGMT, POLB, EHMT2, STAT3, TYMS, CCND1, G6PD, FYN, PTK2B, NQO1, IL2 |
| BP | negative regulation of gene expression | 14 | 5.343511 | 2.12E-07 | XDH, HRAS, TNF, MAP2K1, ESR1, MIF, PGR, AKT1, CCNB1, ACE, FYN, NOS2, TERT, GBA |
| BP | transcription initiation from RNA polymerase II promoter | 14 | 5.343511 | 7.06E-07 | PPARA, PPARD, ESRRA, AR, THRB, PPARG, ESR1, CDK9, NR3C1, RORA, ESR2, PGR, VDR, NR1H4 |
| BP | positive regulation of apoptotic process | 14 | 5.343511 | 8.39E-04 | NOX4, LDHA, TNF, CYP1B1, PTGS2, CDK4, ITGB1, SRC, CTNNB1, AKT1, PDGFRB, NGFR, IGFBP3, TOP2A |
| BP | extracellular matrix disassembly | 13 | 4.961832 | 1.90E-09 | MMP9, MMP8, ELANE, MMP7, PRSS1, MMP3, MMP14, MMP2, MMP13, MMP12, MMP1, PLG, CAPN1 |
| BP | phosphatidylinositol phosphorylation | 13 | 4.961832 | 2.28E-08 | PIK3CG, EGFR, GRB2, PIK3CB, ERBB2, PIK3CD, KIT, FYN, LCK, PDGFRA, PIK3CA, PDGFRB, PIK3R1 |
| BP | cellular response to hypoxia | 13 | 4.961832 | 2.91E-08 | PPARD, PTGS2, RORA, PRKCE, SRC, SLC29A1, AKT1, CCNB1, VEGFA, MDM2, NFE2L2, MTOR, TERT |
| BP | activation of MAPK activity | 13 | 4.961832 | 9.92E-08 | MAPK1, CDK1, TNF, MAP2K1, PIK3CB, MAPK14, DRD4, CD81, IKBKG, TLR4, MAPK10, KIT, GRM1 |
| BP | positive regulation of angiogenesis | 13 | 4.961832 | 2.21E-07 | PRKCA, FLT1, CYP1B1, PTK2B, PGF, F3, VEGFA, SERPINE1, CXCR2, NFE2L2, TERT, KDR, ALOX12 |
| BP | peptidyl-serine phosphorylation | 13 | 4.961832 | 5.51E-07 | PRKCA, CDK1, PRKCE, CDK5, CDK2, SRC, AKT1, MAPK1, PLK1, MAPK14, GSK3B, MTOR, SYK |
| BP | T cell receptor signaling pathway | 13 | 4.961832 | 3.33E-06 | PTPRC, MAPK1, HRAS, FYN, PIK3CB, RELA, LCK, IKBKG, PIK3CD, PIK3CA, IKBKB, CHUK, PIK3R1 |
| BP | cell division | 13 | 4.961832 | 0.008599 | CCNB1, CCNE1, CDK1, CCND1, CCNB2, AURKA, CDK6, NR3C1, PRKCE, CDK4, CDK5, CDC25A, CDK2 |
| BP | response to estrogen | 12 | 4.580153 | 4.25E-09 | MAPK1, LDHA, CCND1, HSP90AA1, EP300, PPARG, ESR1, PDGFRB, SRD5A1, CA2, MMP14, GBA |
| BP | positive regulation of cell growth | 12 | 4.580153 | 6.80E-08 | AKT1, EGFR, CD38, RPS6KA3, CSNK2A1, PTK2B, ERBB2, F2, ADAM17, MMP14, IL2, ALOX12 |
| BP | regulation of signal transduction by p53 class mediator | 12 | 4.580153 | 3.59E-06 | AKT1, EHMT1, EP300, CSNK2A1, MAPK14, MDM2, CHEK1, AURKA, AURKB, EHMT2, CDK5, CDK2 |
| BP | Fc-gamma receptor signaling pathway involved in phagocytosis | 12 | 4.580153 | 4.53E-06 | HSP90AB1, MAPK1, PTK2, HSP90AA1, FYN, PIK3CB, GRB2, PIK3CA, PRKCE, PIK3R1, SRC, SYK |
| BP | positive regulation of nitric oxide biosynthetic process | 11 | 4.198473 | 8.15E-10 | OPRM1, HSP90AB1, AKT1, EGFR, TNF, HSP90AA1, PTGS2, PTK2B, ESR1, TLR4, MTOR |
| BP | cellular response to organic cyclic compound | 11 | 4.198473 | 2.15E-08 | CCNB1, HSP90AB1, ALPL, AKT1, SLC16A1, CASP3, TNF, CYP1B1, CYP1A1, MGMT, STAT3 |
| BP | positive regulation of phosphatidylinositol 3-kinase signaling | 11 | 4.198473 | 5.64E-08 | PTPN6, PTK2, PPARD, FLT1, FYN, FLT3, F2, PDGFRA, PDGFRB, KIT, KDR |
| BP | wound healing | 11 | 4.198473 | 4.23E-07 | OPRM1, EGFR, PPARA, ALOX15, CASP3, PPARD, ERBB2, PDGFRA, PDGFRB, MTOR, ITGB3 |
| BP | positive regulation of MAPK cascade | 11 | 4.198473 | 4.77E-07 | AR, HRAS, ADRB2, FLT1, FLT3, NGFR, KIT, PRKCE, IGFBP3, KDR, CTNNB1 |
| BP | cellular response to lipopolysaccharide | 11 | 4.198473 | 1.02E-05 | EDNRB, ARG1, PPARD, TNF, MAPK14, RELA, SERPINE1, TLR4, NOS2, SRC, NR1H4 |
| BP | regulation of cell cycle | 11 | 4.198473 | 2.31E-05 | CCNB1, CCNE1, PTPRC, CCNB2, EP300, PLK1, ITGB1, SRC, CDC25A, HSPA8, STAT3 |
| BP | positive regulation of NF-kappaB transcription factor activity | 11 | 4.198473 | 4.23E-05 | AR, TNF, RELA, IKBKG, TLR4, HSPA1A, EIF2AK2, IKBKB, CHUK, BTK, TLR9 |
| BP | G2/M transition of mitotic cell cycle | 11 | 4.198473 | 5.45E-05 | CCNB1, CDK1, HSP90AA1, CCNB2, PLK1, CHEK1, AURKA, ABCB1, CDC25A, CDK2, CALM1 |
| BP | ERBB2 signaling pathway | 10 | 3.816794 | 4.91E-09 | AKT1, EGFR, NRAS, HRAS, HSP90AA1, GRB2, ERBB2, PIK3CA, PIK3R1, SRC |
| BP | peptidyl-tyrosine autophosphorylation | 10 | 3.816794 | 8.01E-09 | IGF1R, PTK2, FYN, PTK2B, LCK, JAK3, SRC, KDR, SYK, BTK |
| BP | epidermal growth factor receptor signaling pathway | 10 | 3.816794 | 1.79E-07 | EGFR, NRAS, PTK2, HRAS, GRB2, PTK2B, ADAM17, PIK3CA, PIK3R1, SRC |
| BP | steroid hormone mediated signaling pathway | 10 | 3.816794 | 2.09E-07 | PGR, VDR, PPARA, ESRRA, PPARD, THRB, PPARG, ESR1, RORA, NR1H4 |
| BP | collagen catabolic process | 10 | 3.816794 | 5.83E-07 | MMP9, MMP8, MMP7, CTSD, MMP14, MMP3, MMP13, MMP2, MMP12, MMP1 |
| BP | cellular response to drug | 10 | 3.816794 | 1.12E-06 | HSP90AB1, EGFR, BRAF, REN, TH, FBP1, NFE2L2, NOS2, KCNH2, EHMT2 |
| BP | circadian rhythm | 10 | 3.816794 | 2.29E-06 | EGFR, TOP1, TYMS, HDAC3, EP300, GSK3B, SLC6A4, SERPINE1, NOS2, CDK4 |
| BP | xenobiotic metabolic process | 10 | 3.816794 | 3.20E-06 | CYP3A4, CYP1B1, CYP2C19, CYP2C9, PTGS1, RORA, CYP1A2, NQO1, AHR, AKR1C1 |
| BP | T cell costimulation | 10 | 3.816794 | 3.20E-06 | AKT1, PTPN6, FYN, GRB2, LCK, PIK3CA, MTOR, PIK3R1, SRC, DPP4 |
| BP | positive regulation of protein kinase B signaling | 10 | 3.816794 | 5.96E-06 | PIK3CG, NOX4, AKR1C3, EGFR, PTK2, TNF, F10, F3, MTOR, SRC |
| BP | G1/S transition of mitotic cell cycle | 10 | 3.816794 | 2.91E-05 | CCNE1, CDK1, TYMS, CCND1, DHFR, CDK6, CDK4, ITGB1, CDC25A, CDK2 |
| BP | stimulatory C-type lectin receptor signaling pathway | 10 | 3.816794 | 3.67E-05 | NRAS, HRAS, EP300, FYN, RELA, IKBKG, IKBKB, SRC, CHUK, SYK |
| BP | positive regulation of I-kappaB kinase/NF-kappaB signaling | 10 | 3.816794 | 9.21E-04 | TNF, FYN, RELA, IKBKG, RHOA, PRKCE, IKBKB, CHUK, CTNNB1, TLR9 |
| BP | cell migration | 10 | 3.816794 | 0.001462 | CDK1, FLT1, FYN, PIK3CB, GSK3B, RHOA, PDGFRB, JAK3, ITGB3, ITGB1 |
| BP | extracellular matrix organization | 10 | 3.816794 | 0.003536 | TTR, PTK2, APP, TNF, SERPINE1, ITGA4, ITGB3, ITGB1, KDR, ITGA2B |
| BP | cell cycle | 10 | 3.816794 | 0.006815 | MAPK1, RPS6KA3, CCND1, PIM1, AURKA, AURKB, PRKCE, CDK5, SRC, AHR |
| BP | positive regulation of reactive oxygen species metabolic process | 9 | 3.435115 | 1.29E-08 | NOX4, AKR1C3, XDH, GRB2, MAPK14, F2, PDGFRB, NFE2L2, NQO2 |
| BP | intracellular receptor signaling pathway | 9 | 3.435115 | 9.71E-08 | PPARA, AR, ESRRA, PPARD, THRB, RORA, AHR, STAT3, NR1H4 |
| BP | cellular response to hydrogen peroxide | 9 | 3.435115 | 2.54E-06 | CDK1, ARG1, CYP1B1, RELA, EZH2, MDM2, NFE2L2, APEX1, HDAC6 |
| BP | positive regulation of smooth muscle cell proliferation | 9 | 3.435115 | 3.78E-06 | AKT1, EGFR, TNF, PTGS2, HMGCR, ELANE, PDGFRB, MTOR, ALOX12 |
| BP | positive regulation of endothelial cell proliferation | 9 | 3.435115 | 1.10E-05 | PRKCA, AKT1, ARG1, PGF, F3, VEGFA, MTOR, ITGB3, KDR |
| BP | negative regulation of inflammatory response | 9 | 3.435115 | 3.00E-05 | PPARA, PPARD, PTGER4, ELANE, RORA, NR1H4, ADA, GBA, IL2 |
| BP | activation of cysteine-type endopeptidase activity involved in apoptotic process | 9 | 3.435115 | 4.29E-05 | XDH, CASP3, RET, TNF, F3, LCK, PPARG, EGLN3, NGFR |
| BP | phosphorylation | 9 | 3.435115 | 1.62E-04 | PIK3CG, AKT1, TOP1, PIK3CB, PIK3CD, PIK3CA, MTOR, CDK5, STAT3 |
| BP | positive regulation of sequence-specific DNA binding transcription factor activity | 9 | 3.435115 | 2.27E-04 | AKT1, KDM1A, TNF, EP300, PPARG, ESR1, ESR2, KIT, CTNNB1 |
| BP | heart development | 9 | 3.435115 | 0.007768 | EDNRA, PPARA, PPARD, ECE1, EP300, MAP2K1, ERBB2, PPARG, TH |
| BP | in utero embryonic development | 9 | 3.435115 | 0.008794 | EDNRA, CCNB1, KDM1A, AR, PRMT1, CCNB2, VEGFA, ITGB1, CTNNB1 |
| BP | positive regulation of phosphatidylinositol 3-kinase activity | 8 | 3.053435 | 3.77E-07 | PTK2, FLT1, FLT3, PTK2B, PDGFRA, PDGFRB, KIT, SRC |
| BP | activation of adenylate cyclase activity | 8 | 3.053435 | 2.37E-06 | PRKCA, EDNRA, AVPR2, ADRB2, ADRB1, GIPR, GLP1R, CALM1 |
| BP | steroid metabolic process | 8 | 3.053435 | 3.94E-06 | AKR1C3, CYP3A4, CYP17A1, CYP1B1, CYP1A1, CYP2C19, CYP2C9, AKR1B10 |
| BP | visual learning | 8 | 3.053435 | 5.40E-06 | APP, BRAF, KMT2A, HMGCR, KIT, MTOR, CDK5, ITGB1 |
| BP | B cell receptor signaling pathway | 8 | 3.053435 | 1.87E-05 | MAPK1, PTPRC, CD38, PTPN6, PIK3CD, LCK, SYK, BTK |
| BP | positive regulation of fibroblast proliferation | 8 | 3.053435 | 1.87E-05 | CCNB1, EGFR, PDGFRA, ESR1, PDGFRB, CDK6, CDK4, MIF |
| BP | I-kappaB kinase/NF-kappaB signaling | 8 | 3.053435 | 3.77E-05 | AVPR2, TNF, ROCK1, IKBKG, TLR4, IKBKB, CHUK, BTK |
| BP | response to glucocorticoid | 8 | 3.053435 | 6.37E-05 | ALPL, TYMS, CASP3, TNF, PTGS2, BCHE, HSD11B2, GBA |
| BP | apoptotic signaling pathway | 8 | 3.053435 | 1.12E-04 | PRKCA, CD38, CASP3, PPARD, TLR4, NGFR, BTK, DAPK1 |
| BP | positive regulation of inflammatory response | 8 | 3.053435 | 1.34E-04 | EGFR, PTGER4, SERPINE1, PLA2G2A, FABP4, TLR4, TLR9, IL2 |
| BP | response to nutrient | 8 | 3.053435 | 1.46E-04 | AKR1C3, LDHA, HMGCR, SLC6A4, GIPR, PPARG, MTOR, NQO1 |
| BP | regulation of cellular response to heat | 8 | 3.053435 | 1.59E-04 | HSP90AB1, MAPK1, HSP90AA1, EP300, GSK3B, HSPA1A, MTOR, HSPA8 |
| BP | positive regulation of peptidyl-tyrosine phosphorylation | 8 | 3.053435 | 2.78E-04 | PTK2B, VEGFA, CD81, MTOR, ITGB3, SRC, SYK, MIF |
| BP | ephrin receptor signaling pathway | 8 | 3.053435 | 3.73E-04 | PTK2, HRAS, ROCK1, FYN, MMP9, RHOA, MMP2, SRC |
| BP | organ morphogenesis | 8 | 3.053435 | 5.63E-04 | HRAS, EP300, BRAF, THRB, RELA, GSK3B, TH, SYK |
| BP | integrin-mediated signaling pathway | 8 | 3.053435 | 8.73E-04 | PTK2, PTK2B, ITGA4, ITGB3, ITGB1, SRC, SYK, ITGA2B |
| BP | response to virus | 8 | 3.053435 | 0.001616 | TNF, CYP1A1, IKBKG, CDK6, EIF2AK2, IKBKB, SRC, CHUK |
| BP | cellular protein metabolic process | 8 | 3.053435 | 0.002413 | TTR, APP, F2, IGFBP3, MMP13, MMP2, PLG, MMP1 |
| BP | negative regulation of neuron apoptotic process | 8 | 3.053435 | 0.004499 | HSP90AB1, HRAS, ROCK1, BRAF, FYN, PTK2B, PIK3CA, TERT |
| BP | adaptive immune response | 8 | 3.053435 | 0.008295 | PIK3CG, FYN, PTK2B, PIK3CD, JAK3, SYK, BTK, IL2 |
| BP | response to food | 7 | 2.671756 | 5.81E-07 | OPRM1, AKT1, SLC16A1, G6PD, CYP1A1, HSD11B2, MPO |
| BP | positive regulation of protein localization to nucleus | 7 | 2.671756 | 5.81E-07 | AKT1, CDK1, FYN, PLK1, F2, NGFR, SRC |
| BP | DNA methylation | 7 | 2.671756 | 1.80E-06 | EHMT1, KMT2A, MGMT, EZH2, DNMT1, EHMT2, DNMT3B |
| BP | response to cocaine | 7 | 2.671756 | 8.41E-06 | OPRM1, HSP90AB1, HSP90AA1, PTK2B, MDM2, MTOR, DNMT3B |
| BP | peptidyl-threonine phosphorylation | 7 | 2.671756 | 2.37E-05 | AKT1, MAPK1, CDK1, GSK3B, CHEK1, MTOR, CDK5 |
| BP | positive regulation of B cell proliferation | 7 | 2.671756 | 2.76E-05 | PTPRC, CD38, CD81, TLR4, ADA, IL2, MIF |
| BP | negative regulation of neuron death | 7 | 2.671756 | 3.21E-05 | AKT1, PPARA, IKBKG, CDK5, STAT3, CTNNB1, GBA |
| BP | phosphatidylinositol-3-phosphate biosynthetic process | 7 | 2.671756 | 1.04E-04 | PIK3CG, PIK3CB, GRB2, PIK3CD, PIK3CA, PIK3R1, TLR9 |
| BP | cellular response to cAMP | 7 | 2.671756 | 1.46E-04 | PIK3CG, NOX4, KDM1A, APP, SRD5A1, CFTR, APEX1 |
| BP | sensory perception of pain | 7 | 2.671756 | 1.46E-04 | OPRM1, EDNRB, MAPK1, PTGS2, MME, GRM1, CDK5 |
| BP | rhythmic process | 7 | 2.671756 | 1.80E-04 | CSNK2A1, DRD4, EZH2, PPARG, MAPK10, TOP2A, CDK5 |
| BP | negative regulation of protein binding | 7 | 2.671756 | 2.44E-04 | KDM1A, PPARA, ACE, ROCK1, GSK3B, AURKA, AURKB |
| BP | response to mechanical stimulus | 7 | 2.671756 | 2.95E-04 | CCNB1, PTGER4, PTK2B, PPARG, MPO, MMP14, SRC |
| BP | response to stress | 7 | 2.671756 | 3.54E-04 | HSP90AB1, EGFR, MAPK1, HSP90AA1, PTK2B, MTOR, GLP1R |
| BP | DNA damage response, signal transduction by p53 class mediator resulting in cell cycle arrest | 7 | 2.671756 | 3.87E-04 | CCNB1, CDK1, PRMT1, EP300, MDM2, AURKA, CDK2 |
| BP | regulation of blood pressure | 7 | 2.671756 | 5.00E-04 | EDNRA, EDNRB, ACE, PTGS2, REN, PTGS1, PPARG |
| BP | B cell differentiation | 7 | 2.671756 | 5.43E-04 | EP300, FLT3, ADAM17, ITGA4, JAK3, ITGB1, PIK3R1 |
| BP | glucose metabolic process | 7 | 2.671756 | 5.89E-04 | AKT1, PPARD, TNF, G6PD, MAPK14, PIK3CA, GAPDH |
| BP | regulation of insulin secretion | 7 | 2.671756 | 5.89E-04 | PRKCA, SSTR5, SLC16A1, TNF, GIPR, NOS2, GLP1R |
| BP | negative regulation of cysteine-type endopeptidase activity involved in apoptotic process | 7 | 2.671756 | 6.90E-04 | AKT1, RPS6KA3, CSNK2A1, VEGFA, MDM2, NGFR, SRC |
| BP | regulation of protein stability | 7 | 2.671756 | 7.45E-04 | MAPK1, HDAC3, CD81, AURKA, HSPA8, TERT, HDAC6 |
| BP | positive regulation of peptidyl-serine phosphorylation | 7 | 2.671756 | 7.45E-04 | AKT1, TNF, BRAF, GSK3B, VEGFA, PIK3CA, MIF |
| BP | cellular response to mechanical stimulus | 7 | 2.671756 | 8.03E-04 | AKT1, EGFR, PTGER4, PTGS2, MMP7, CHEK1, TLR4 |
| BP | lung development | 7 | 2.671756 | 0.001149 | EGFR, ARG1, EP300, VEGFA, HSD11B1, CYP1A2, MMP14 |
| BP | regulation of gene expression | 7 | 2.671756 | 0.004612 | F2, PIK3CA, CDK6, CYP1A2, CDK4, DNMT3B, AHR |
| BP | glucose homeostasis | 7 | 2.671756 | 0.004841 | AKT1, SSTR5, SLC16A1, PPARG, NGFR, STAT3, NR1H4 |
| BP | platelet degranulation | 7 | 2.671756 | 0.005324 | APP, VEGFA, SERPINE1, ITGB3, PLG, CALM1, ITGA2B |
| BP | cellular response to tumor necrosis factor | 7 | 2.671756 | 0.007297 | RELA, FABP4, RORA, NFE2L2, IKBKB, CHUK, GBA |
| BP | response to oxidative stress | 7 | 2.671756 | 0.007297 | AKT1, EGFR, APP, PTGS2, PTGS1, MPO, MMP14 |
| BP | cellular response to fluid shear stress | 6 | 2.290076 | 6.21E-07 | HDAC3, PTGS2, PTK2B, CA2, NFE2L2, SRC |
| BP | nitric oxide biosynthetic process | 6 | 2.290076 | 9.96E-07 | AKT1, CYP1B1, NOS1, RORA, NOS2, NQO1 |
| BP | positive regulation of vascular smooth muscle cell proliferation | 6 | 2.290076 | 2.27E-06 | MMP9, ERN1, MDM2, MMP2, TERT, NQO2 |
| BP | response to vitamin A | 6 | 2.290076 | 6.22E-06 | ARG1, TYMS, PPARD, CYP1A1, PPARG, DNMT3B |
| BP | response to immobilization stress | 6 | 2.290076 | 1.42E-05 | CYP1A1, PTK2B, REN, PPARG, TH, CYP1A2 |
| BP | positive regulation of nitric-oxide synthase activity | 6 | 2.290076 | 1.82E-05 | AKT1, DHFR, PTK2B, ESR1, TERT, CALM1 |
| BP | cellular response to vascular endothelial growth factor stimulus | 6 | 2.290076 | 2.29E-05 | AKT1, FLT1, MAPK14, VEGFA, ERN1, KDR |
| BP | phosphatidylinositol 3-kinase signaling | 6 | 2.290076 | 5.23E-05 | PIK3CG, IGF1R, PIK3CB, ERBB2, PIK3CD, PIK3R1 |
| BP | regulation of cell motility | 6 | 2.290076 | 6.29E-05 | EGFR, ROCK1, ERBB2, CD81, RHOA, CDK6 |
| BP | lipopolysaccharide-mediated signaling pathway | 6 | 2.290076 | 1.22E-04 | AKT1, MAPK1, TNF, MAPK14, TLR4, PRKCE |
| BP | positive regulation of protein serine/threonine kinase activity | 6 | 2.290076 | 1.90E-04 | HSP90AB1, MAP2K1, EZH2, RHOA, SRC, CALM1 |
| BP | negative regulation of extrinsic apoptotic signaling pathway in absence of ligand | 6 | 2.290076 | 2.49E-04 | AKT1, TNF, MCL1, FYN, HSPA1A, TERT |
| BP | platelet aggregation | 6 | 2.290076 | 4.07E-04 | PIK3CG, PTPN6, PIK3CB, PDGFRA, ITGB3, ITGA2B |
| BP | receptor internalization | 6 | 2.290076 | 5.10E-04 | ACHE, GRB2, CD81, CXCR2, ITGB1, SYK |
| BP | regulation of macroautophagy | 6 | 2.290076 | 5.68E-04 | CASP3, ERN1, CDK5, GAPDH, GBA, CAPN1 |
| BP | cellular response to UV | 6 | 2.290076 | 5.68E-04 | KDM1A, EP300, PTGS2, AURKB, PIK3R1, CDC25A |
| BP | cellular response to growth factor stimulus | 6 | 2.290076 | 6.31E-04 | NOS1, ERBB2, TH, MDM2, SRD5A1, CTNNB1 |
| BP | positive regulation of endothelial cell migration | 6 | 2.290076 | 6.99E-04 | PRKCA, PTK2B, VEGFA, ITGB3, KDR, ALOX12 |
| BP | regulation of circadian rhythm | 6 | 2.290076 | 9.36E-04 | PPARA, DRD4, EZH2, PPARG, MAPK10, TOP2A |
| BP | response to organic cyclic compound | 6 | 2.290076 | 9.36E-04 | EDNRB, CDK1, G6PD, CYP1A1, PTGES, MMP14 |
| BP | response to hydrogen peroxide | 6 | 2.290076 | 0.001125 | LDHA, CASP3, PTK2B, PDGFRB, SRC, ADA |
| BP | circadian regulation of gene expression | 6 | 2.290076 | 0.00186 | PPARA, TOP1, KMT2A, RORA, NGFR, AHR |
| BP | response to calcium ion | 6 | 2.290076 | 0.00201 | EGFR, CCND1, PTGES, PTK2B, GIPR, CALM1 |
| BP | positive regulation of protein binding | 6 | 2.290076 | 0.002514 | HSP90AB1, ACE, EP300, GSK3B, CDK5, TERT |
| BP | positive regulation of proteasomal ubiquitin-dependent protein catabolic process | 6 | 2.290076 | 0.003103 | AKT1, PLK1, GSK3B, MDM2, AURKA, HSPA1A |
| BP | phospholipase C-activating G-protein coupled receptor signaling pathway | 6 | 2.290076 | 0.003548 | OPRM1, EDNRB, PTGER3, TACR1, ESR1, CXCR2 |
| BP | Ras protein signal transduction | 6 | 2.290076 | 0.004571 | NRAS, HRAS, GRB2, MAPK14, DNMT1, CDK2 |
| BP | cellular response to insulin stimulus | 6 | 2.290076 | 0.00684 | AKT1, PPARG, SRD5A1, PARP1, PIK3R1, SRC |
| BP | viral entry into host cell | 6 | 2.290076 | 0.008018 | CD81, ANPEP, HSPA1A, ITGB3, ITGB1, DPP4 |
| BP | omega-hydroxylase P450 pathway | 5 | 1.908397 | 6.66E-06 | CYP1B1, CYP1A1, CYP2C19, CYP2C9, CYP1A2 |
| BP | positive regulation of phospholipase C activity | 5 | 1.908397 | 6.66E-06 | FLT1, PDGFRA, ESR1, PDGFRB, KIT |
| BP | positive regulation of cell size | 5 | 1.908397 | 1.10E-05 | HSP90AB1, KDM1A, RET, HSP90AA1, CDK4 |
| BP | exogenous drug catabolic process | 5 | 1.908397 | 2.52E-05 | CYP3A4, NOS1, CYP2C19, CYP2C9, CYP1A2 |
| BP | positive regulation of chemokine production | 5 | 1.908397 | 1.14E-04 | TNF, ADAM17, TLR4, EIF2AK2, TLR9 |
| BP | cellular response to prostaglandin E stimulus | 5 | 1.908397 | 1.14E-04 | AKT1, PTGER2, PTGER4, PPARG, PRKCE |
| BP | negative regulation of anoikis | 5 | 1.908397 | 1.14E-04 | PTK2, MCL1, PIK3CA, ITGB1, SRC |
| BP | epoxygenase P450 pathway | 5 | 1.908397 | 1.45E-04 | CYP1B1, CYP1A1, CYP2C19, CYP2C9, CYP1A2 |
| BP | positive regulation of blood vessel endothelial cell migration | 5 | 1.908397 | 1.81E-04 | PRKCA, AKT1, MAPK14, VEGFA, NFE2L2 |
| BP | cellular response to peptide hormone stimulus | 5 | 1.908397 | 2.24E-04 | FYN, RELA, MDM2, APEX1, SRC |
| BP | negative regulation of cell cycle arrest | 5 | 1.908397 | 2.24E-04 | CCND1, CDK9, MDM2, CDK4, MIF |
| BP | positive regulation of NF-kappaB import into nucleus | 5 | 1.908397 | 2.73E-04 | TNF, PTGS2, RHOA, TLR4, TLR9 |
| BP | bone resorption | 5 | 1.908397 | 3.30E-04 | NOX4, ADRB2, PTK2B, SRC, CTNNB1 |
| BP | response to morphine | 5 | 1.908397 | 3.94E-04 | RELA, MDM2, MTOR, PRKCE, ADA |
| BP | positive regulation of cytokine secretion | 5 | 1.908397 | 5.50E-04 | TNF, PTGER4, SRC, SYK, MIF |
| BP | cell aging | 5 | 1.908397 | 5.50E-04 | NOX4, CDK1, MTOR, PDCD4, MIF |
| BP | positive regulation of interleukin-8 production | 5 | 1.908397 | 6.42E-04 | TNF, SERPINE1, TLR4, HSPA1A, TLR9 |
| BP | drug metabolic process | 5 | 1.908397 | 7.45E-04 | CYP3A4, CYP1A1, CYP2C19, CYP2C9, CYP1A2 |
| BP | endodermal cell differentiation | 5 | 1.908397 | 7.45E-04 | MMP9, MMP8, ITGA4, MMP14, MMP2 |
| BP | negative regulation of endothelial cell apoptotic process | 5 | 1.908397 | 8.58E-04 | BRAF, SERPINE1, NFE2L2, TERT, KDR |
| BP | negative regulation of interleukin-6 production | 5 | 1.908397 | 8.58E-04 | TNF, TLR4, NR1H4, GBA, TLR9 |
| BP | positive regulation of mitotic cell cycle | 5 | 1.908397 | 8.58E-04 | PRKCA, CCNB1, CDK1, APP, MDM2 |
| BP | positive regulation of vasodilation | 5 | 1.908397 | 9.83E-04 | EGFR, PPARD, NOS1, NOS2, ALOX12 |
| BP | liver regeneration | 5 | 1.908397 | 9.83E-04 | EGFR, TYMS, CCND1, EZH2, AURKA |
| BP | endothelial cell migration | 5 | 1.908397 | 9.83E-04 | PTK2, CYP1B1, RHOA, PIK3CA, DPP4 |
| BP | positive regulation of glucose import | 5 | 1.908397 | 0.001121 | AKT1, MAPK14, NFE2L2, PIK3R1, TERT |
| BP | regulation of tumor necrosis factor-mediated signaling pathway | 5 | 1.908397 | 0.001121 | TNF, IKBKG, IKBKB, CHUK, SYK |
| BP | T cell differentiation | 5 | 1.908397 | 0.001121 | PTPRC, PIK3CD, LCK, KIT, IL2 |
| BP | response to amino acid | 5 | 1.908397 | 0.001272 | ARG1, CASP3, RELA, MTOR, CHUK |
| BP | response to antibiotic | 5 | 1.908397 | 0.001436 | ALPL, CASP3, HSP90AA1, CYP1A1, MDM2 |
| BP | positive regulation of vasoconstriction | 5 | 1.908397 | 0.001436 | AKT1, EGFR, AVPR2, CD38, PTGS2 |
| BP | placenta development | 5 | 1.908397 | 0.002016 | HSP90AB1, PTK2, MAPK14, PPARG, ADA |
| BP | cell maturation | 5 | 1.908397 | 0.002241 | REN, VEGFA, PPARG, CTNNB1, BTK |
| BP | response to cold | 5 | 1.908397 | 0.002241 | ADRB2, HSP90AA1, ADRB1, TRPM8, PPARG |
| BP | negative regulation of protein ubiquitination | 5 | 1.908397 | 0.003627 | FYN, HSPA1A, MTOR, PRKCE, CDK5 |
| BP | positive regulation of insulin secretion | 5 | 1.908397 | 0.003627 | CD38, PPARD, GIPR, PRKCE, SIRT3 |
| BP | protein catabolic process | 5 | 1.908397 | 0.003627 | AKT1, REN, ELANE, CTSD, MTOR |
| BP | lactation | 5 | 1.908397 | 0.003961 | SLC29A1, XDH, VDR, CCND1, VEGFA |
| BP | positive regulation of DNA replication | 5 | 1.908397 | 0.003961 | EGFR, CDK1, IGF1R, HRAS, PDGFRA |
| BP | positive regulation of cell adhesion | 5 | 1.908397 | 0.004315 | PRKCA, TNF, ERBB2, VEGFA, ALOX12 |
| BP | positive regulation of neuron apoptotic process | 5 | 1.908397 | 0.004315 | CASP3, NQO1, CDK5, CTNNB1, NQO2 |
| BP | thymus development | 5 | 1.908397 | 0.004315 | MAPK1, CCNB2, BRAF, MAP2K1, CTNNB1 |
| BP | mitotic nuclear envelope disassembly | 5 | 1.908397 | 0.004689 | PRKCA, CCNB1, CDK1, CCNB2, PLK1 |
| BP | positive regulation of actin filament polymerization | 5 | 1.908397 | 0.005085 | ALOX15, GRB2, PTK2B, MTOR, PRKCE |
| BP | endoplasmic reticulum unfolded protein response | 5 | 1.908397 | 0.005085 | CCND1, PTPN1, HSPA5, NFE2L2, EIF2AK2 |
| BP | response to cAMP | 5 | 1.908397 | 0.005503 | LDHA, BRAF, PTK2B, REN, RELA |
| BP | organ regeneration | 5 | 1.908397 | 0.005943 | CDK1, PGF, FLT3, PPARG, CDK4 |
| BP | positive regulation of protein kinase activity | 5 | 1.908397 | 0.005943 | PTPRC, PTK2, TNF, PTK2B, CDK5 |
| BP | T cell activation | 5 | 1.908397 | 0.005943 | PIK3CG, FYN, PIK3CD, DPP4, ADA |
| BP | cellular response to starvation | 5 | 1.908397 | 0.005943 | AKR1C3, SRD5A1, EHMT2, HSPA8, GBA |
| BP | social behavior | 5 | 1.908397 | 0.006406 | HRAS, SLC6A4, DRD4, TH, MTOR |
| BP | nucleobase-containing compound metabolic process | 5 | 1.908397 | 0.006892 | SLC29A1, TYMS, MTAP, TPMT, PNP |
| BP | substantia nigra development | 5 | 1.908397 | 0.006892 | LDHA, G6PD, RHOA, HSPA5, CALM1 |
| BP | adenylate cyclase-activating G-protein coupled receptor signaling pathway | 5 | 1.908397 | 0.007402 | PTGER1, PTGER2, ADRB2, PTGER3, PTGER4 |
| BP | cellular response to glucose stimulus | 5 | 1.908397 | 0.008495 | SLC29A1, NOX4, TH, ERN1, PIK3CA |
| BP | positive regulation of translation | 5 | 1.908397 | 0.009079 | MAPK1, PTK2B, ERBB2, MTOR, CDK4 |
| BP | monoterpenoid metabolic process | 4 | 1.526718 | 7.09E-05 | CYP3A4, CYP2C19, CYP2C9, CYP1A2 |
| BP | response to fungicide | 4 | 1.526718 | 1.23E-04 | KDM1A, EHMT1, SRD5A1, EHMT2 |
| BP | positive regulation of cyclin-dependent protein serine/threonine kinase activity involved in G1/S transition of mitotic cell cycle | 4 | 1.526718 | 1.94E-04 | AKT1, EGFR, PIM1, ADAM17 |
| BP | response to salt stress | 4 | 1.526718 | 2.88E-04 | HSP90AB1, TNF, HSP90AA1, TH |
| BP | progesterone metabolic process | 4 | 1.526718 | 2.88E-04 | AKR1C3, CYP17A1, SRD5A1, AKR1C1 |
| BP | cyclooxygenase pathway | 4 | 1.526718 | 4.06E-04 | AKR1C3, PTGS2, PTGES, PTGS1 |
| BP | I-kappaB phosphorylation | 4 | 1.526718 | 7.28E-04 | TLR4, IKBKB, CHUK, TLR9 |
| BP | oxidative demethylation | 4 | 1.526718 | 7.28E-04 | CYP3A4, CYP2C9, FTO, CYP1A2 |
| BP | positive regulation of DNA biosynthetic process | 4 | 1.526718 | 9.36E-04 | NOX4, PTK2B, PDGFRB, SRC |
| BP | leukocyte tethering or rolling | 4 | 1.526718 | 9.36E-04 | TNF, ROCK1, ITGA4, ITGB1 |
| BP | lipoxygenase pathway | 4 | 1.526718 | 9.36E-04 | ALOX15, PTGS2, ALOX5, ALOX12 |
| BP | negative regulation of smooth muscle cell migration | 4 | 1.526718 | 0.001177 | PPARD, SERPINE1, APEX1, IGFBP3 |
| BP | prostaglandin biosynthetic process | 4 | 1.526718 | 0.001177 | PTGS2, PTGES, PTGS1, MIF |
| BP | JAK-STAT cascade involved in growth hormone signaling pathway | 4 | 1.526718 | 0.001455 | MAPK1, PTPN1, JAK3, STAT3 |
| BP | stress fiber assembly | 4 | 1.526718 | 0.001455 | PTK2B, RHOA, ITGB1, SRC |
| BP | positive regulation of cardiac muscle hypertrophy | 4 | 1.526718 | 0.00177 | PRKCA, PDE5A, CDK9, PARP1 |
| BP | mammary gland alveolus development | 4 | 1.526718 | 0.002125 | AR, CCND1, VEGFA, ESR1 |
| BP | vasoconstriction | 4 | 1.526718 | 0.002125 | EDNRA, EDNRB, HTR1A, SLC6A4 |
| BP | histone methylation | 4 | 1.526718 | 0.002125 | PRMT1, EHMT1, PRMT7, EHMT2 |
| BP | response to hyperoxia | 4 | 1.526718 | 0.002125 | CYP1A1, PDGFRB, POLB, CDK4 |
| BP | positive regulation of histone H3-K4 methylation | 4 | 1.526718 | 0.002125 | KMT2A, DNMT1, DNMT3B, CTNNB1 |
| BP | regulation of protein binding | 4 | 1.526718 | 0.002959 | APP, PRMT7, PLK1, SRC |
| BP | positive regulation of pri-miRNA transcription from RNA polymerase II promoter | 4 | 1.526718 | 0.003442 | RELA, NGFR, TERT, STAT3 |
| BP | cellular response to reactive oxygen species | 4 | 1.526718 | 0.003442 | AKR1C3, PDGFRA, SRC, BTK |
| BP | negative regulation of proteolysis | 4 | 1.526718 | 0.00397 | AKT1, F2, CDK5, HDAC6 |
| BP | positive regulation of axonogenesis | 4 | 1.526718 | 0.00397 | BRAF, MAP2K1, RHOA, NGFR |
| BP | fibrinolysis | 4 | 1.526718 | 0.00397 | F2, SERPINE1, PLG, PLAU |
| BP | cellular response to cytokine stimulus | 4 | 1.526718 | 0.004545 | FLT3, MME, CDK9, ITGA4 |
| BP | osteoclast differentiation | 4 | 1.526718 | 0.005168 | TNF, MAPK14, GLO1, CTNNB1 |
| BP | negative regulation of adenylate cyclase activity | 4 | 1.526718 | 0.005168 | OPRM1, PRKCA, EDNRB, DRD4 |
| BP | regulation of ubiquitin-protein ligase activity involved in mitotic cell cycle | 4 | 1.526718 | 0.005168 | CCNB1, CDK1, PLK1, CDK2 |
| BP | regulation of transcription involved in G1/S transition of mitotic cell cycle | 4 | 1.526718 | 0.005168 | CCNE1, CDK1, TYMS, DHFR |
| BP | cortical actin cytoskeleton organization | 4 | 1.526718 | 0.005839 | TNF, ROCK1, IKBKB, CDK5 |
| BP | cellular response to interleukin-4 | 4 | 1.526718 | 0.005839 | HSP90AB1, ARG1, FASN, HSPA5 |
| BP | cell motility | 4 | 1.526718 | 0.005839 | PTK2, MAP2K1, ADAM17, MMP14 |
| BP | leukocyte cell-cell adhesion | 4 | 1.526718 | 0.00656 | ROCK1, ITGA4, ITGB1, SYK |
| BP | regulation of sequence-specific DNA binding transcription factor activity | 4 | 1.526718 | 0.00656 | MAPK1, MAPK14, MAPK10, SYK |
| BP | regulation of multicellular organism growth | 4 | 1.526718 | 0.007332 | APP, FTO, PIK3CA, STAT3 |
| BP | regulation of nitric-oxide synthase activity | 4 | 1.526718 | 0.007332 | AKT1, EGFR, HSP90AA1, CALM1 |
| BP | positive regulation of peptidyl-threonine phosphorylation | 4 | 1.526718 | 0.008156 | MAPK1, PLK1, GSK3B, CALM1 |
| BP | regulation of transcription from RNA polymerase II promoter in response to hypoxia | 4 | 1.526718 | 0.009032 | EP300, CA9, VEGFA, EGLN3 |
| BP | TRIF-dependent toll-like receptor signaling pathway | 4 | 1.526718 | 0.009032 | IKBKG, TLR4, IKBKB, CHUK |
| BP | cellular response to dexamethasone stimulus | 4 | 1.526718 | 0.00996 | EGFR, ARG1, SRD5A1, DNMT3B |
| BP | positive regulation of cell proliferation by VEGF-activated platelet derived growth factor receptor signaling pathway | 3 | 1.145038 | 7.09E-04 | VEGFA, PDGFRA, PDGFRB |
| BP | respiratory burst involved in defense response | 3 | 1.145038 | 7.09E-04 | PIK3CG, PIK3CD, MPO |
| BP | response to UV-A | 3 | 1.145038 | 7.09E-04 | AKT1, EGFR, CCND1 |
| BP | negative regulation of bicellular tight junction assembly | 3 | 1.145038 | 0.001404 | TNF, ROCK1, IKBKB |
| BP | histone H3-K27 methylation | 3 | 1.145038 | 0.001404 | EHMT1, EZH2, EHMT2 |
| BP | testosterone biosynthetic process | 3 | 1.145038 | 0.001404 | AKR1C3, HSD17B1, HSD17B3 |
| BP | positive regulation of fever generation | 3 | 1.145038 | 0.002316 | TNF, PTGER3, PTGS2 |
| BP | negative regulation of myosin-light-chain-phosphatase activity | 3 | 1.145038 | 0.002316 | TNF, ROCK1, IKBKB |
| BP | heterocycle metabolic process | 3 | 1.145038 | 0.003438 | CYP3A4, CYP2C19, CYP1A2 |
| BP | vascular endothelial growth factor signaling pathway | 3 | 1.145038 | 0.003438 | FLT1, FLT3, VEGFA |
| BP | trachea formation | 3 | 1.145038 | 0.003438 | MAPK1, MAP2K1, CTNNB1 |
| BP | protein localization to chromatin | 3 | 1.145038 | 0.003438 | PLK1, EZH2, ESR1 |
| BP | drug catabolic process | 3 | 1.145038 | 0.003438 | CYP3A4, CYP2C9, CYP1A2 |
| BP | negative regulation of histone H3-K9 methylation | 3 | 1.145038 | 0.003438 | KDM1A, DNMT1, DNMT3B |
| BP | regulation of establishment of endothelial barrier | 3 | 1.145038 | 0.004764 | TNF, ROCK1, IKBKB |
| BP | response to herbicide | 3 | 1.145038 | 0.004764 | ARG1, CYP1A1, TH |
| BP | myeloid progenitor cell differentiation | 3 | 1.145038 | 0.004764 | BRAF, FLT3, KIT |
| BP | response to folic acid | 3 | 1.145038 | 0.004764 | TYMS, BCHE, MGMT |
| BP | acute inflammatory response to antigenic stimulus | 3 | 1.145038 | 0.006288 | OPRM1, ELANE, CXCR2 |
| BP | doxorubicin metabolic process | 3 | 1.145038 | 0.006288 | AKR1C3, AKR1B10, AKR1C1 |
| BP | signal complex assembly | 3 | 1.145038 | 0.006288 | PTK2, PTK2B, SRC |
| BP | cellular response to nicotine | 3 | 1.145038 | 0.006288 | TNF, RELA, TH |
| BP | growth hormone receptor signaling pathway | 3 | 1.145038 | 0.006288 | PTK2, PIK3R1, STAT3 |
| BP | embryonic cleavage | 3 | 1.145038 | 0.006288 | TOP1, PIK3CB, TOP2A |
| BP | arginine catabolic process | 3 | 1.145038 | 0.006288 | ARG1, NOS1, NOS2 |
| BP | daunorubicin metabolic process | 3 | 1.145038 | 0.006288 | AKR1C3, AKR1B10, AKR1C1 |
| BP | positive regulation of interleukin-8 biosynthetic process | 3 | 1.145038 | 0.006288 | TNF, ELANE, TLR4 |
| BP | activation of signaling protein activity involved in unfolded protein response | 3 | 1.145038 | 0.006288 | ERN1, PTPN1, HSPA5 |
| BP | regulation of early endosome to late endosome transport | 3 | 1.145038 | 0.006288 | MAPK1, MAP2K1, SRC |
| BP | positive regulation of myotube differentiation | 3 | 1.145038 | 0.008002 | MAPK14, MTOR, MMP14 |
| BP | glucocorticoid biosynthetic process | 3 | 1.145038 | 0.008002 | CYP17A1, HSD11B1, HSD11B2 |
| BP | positive regulation of alpha-beta T cell differentiation | 3 | 1.145038 | 0.008002 | PNP, ADA, SYK |
| BP | cellular response to low-density lipoprotein particle stimulus | 3 | 1.145038 | 0.008002 | CD81, ITGB1, SYK |
| BP | enzyme linked receptor protein signaling pathway | 3 | 1.145038 | 0.008002 | ERBB2, JAK3, SYK |
| BP | regulation of receptor activity | 3 | 1.145038 | 0.008002 | SERPINE1, PLAU, HDAC6 |
| BP | striated muscle cell differentiation | 3 | 1.145038 | 0.009901 | AKT1, MAPK14, CHUK |
| BP | negative regulation of fibrinolysis | 3 | 1.145038 | 0.009901 | F2, SERPINE1, PLG |
| BP | positive regulation of mitochondrial depolarization | 3 | 1.145038 | 0.009901 | PARP1, KDR, ALOX12 |
| BP | positive regulation of protein import into nucleus, translocation | 3 | 1.145038 | 0.009901 | HSP90AB1, CDK1, HSP90AA1 |
| BP | protein deacetylation | 3 | 1.145038 | 0.009901 | HDAC3, SIRT3, HDAC6 |
| CC | nucleus | 125 | 47.70992 | 8.51E-10 | FHIT, HRAS, LDHA, THRB, PTGS2, PTGS1, AURKA, AURKB, RORA, PNP, MMP2, CTNNB1, BTK, PGR, KDM1A, TOP1, CSNK2A1, SYK, EGFR, BRAF, PIK3CB, RELA, PIM1, POLB, AHR, SSTR5, CD38, MAPK1, EP300, PDGFRA, PDGFRB, NFE2L2, EIF2AK2, ACHE, MCL1, GRB2, ERBB2, CHEK1, ITGB3, SRC, ARG1, VDR, PTK2, DNMT3B, NR1H4, PTPN6, ESRRA, EHMT1, MAP2K1, FTO, TKT, EHMT2, ABCG2, KDR, HDAC3, PPIA, PLK1, IKBKG, FABP4, DNMT1, MTOR, IKBKB, PARP1, HDAC6, HSP90AB1, PPARA, PPARD, EZH2, PPARG, PDCD4, AKR1C3, AKT1, CCNE1, PRMT1, CASP3, PRMT7, NOS2, TOP2A, TERT, TUBB3, CDK1, AR, HSP90AA1, CTBP2, ESR1, CDK6, ESR2, PRKCE, CDK4, GRM1, CDK5, CDK2, CCND1, ADRB2, G6PD, MDM2, MTAP, NGFR, PREP, KMT2A, TH, MGMT, EGLN3, NR3C1, TYMS, PTK2B, HSPA5, APEX1, GAPDH, PIK3R1, HSPA8, FLT3, STAT3, CDC25A, CCNB1, RPS6KA3, CCNB2, FYN, GSK3B, MAPK14, KDM4C, MPO, SETD2, IGFBP3, CALM1 |
| CC | plasma membrane | 120 | 45.80153 | 8.68E-17 | HRAS, CASR, SLC6A4, TLR4, MMP2, SLC7A5, CTNNB1, BTK, TLR9, APP, SLC16A1, CSNK2A1, HTR1A, SERPINE1, PIK3CA, SYK, EGFR, PIK3CG, RET, F10, BRAF, ROCK1, PIK3CB, PIK3CD, PIM1, SSTR5, CD38, F3, F2, PDGFRA, PLA2G2A, PDGFRB, NFE2L2, GLP1R, ITGA2B, ACHE, GRB2, ERBB2, DRD4, MME, ITGB3, ITGB1, ADA, SRC, SLC29A1, PTK2, ACE, ECE1, GABRA1, MAP2K1, MET, ABCB1, ITGA4, ABCG2, KDR, HDAC3, PTPN1, PLAU, OPRM1, TACR1, CXCR2, EDNRA, AKT1, EDNRB, CASP3, RHOA, TERT, DPP4, PRKCA, PTGER1, PTGER2, AR, PTGER3, HSP90AA1, TRPM8, PTGER4, ESR1, CFTR, PRKCE, MMP14, GRM1, CDK5, DAPK1, ALOX15, ADRB2, FOLH1, ADRB1, CA9, CD81, GIPR, LCK, ADAM17, MDM2, CA2, NGFR, KCNH2, ALPL, AVPR2, TNF, KIT, IGF1R, REN, FASN, HSPA5, GAPDH, HSPA8, PIK3R1, PTPRC, FLT1, FLT3, MAPK10, STAT3, PLG, CAPN1, NRAS, FYN, GSK3B, ABCC1, SLC5A7, CALM1 |
| CC | cytoplasm | 115 | 43.89313 | 1.37E-07 | CYP3A4, FHIT, HRAS, LDHA, PTGS2, PTGS1, TLR4, PNP, SLC7A5, CTNNB1, BTK, TLR9, TTR, APP, NQO1, NQO2, SYK, PIK3CG, EGFR, RET, BRAF, RELA, FBP1, PIM1, POLB, AHR, MAPK1, EP300, VEGFA, PDGFRA, PDGFRB, NFE2L2, EIF2AK2, MCL1, GRB2, ERBB2, MME, HSPA1A, ITGB1, ADA, SRC, ARG1, PTK2, GLO1, DNMT3B, PTPN6, MAP2K1, FDPS, HDAC3, PLK1, IKBKG, FABP4, MTOR, IKBKB, HDAC6, HSP90AB1, EZH2, PDCD4, AKR1C3, AKT1, PRMT1, CASP3, NOS2, TOP2A, TUBB3, CHUK, PRKCA, CDK1, AR, HSP90AA1, ELANE, ESR1, CDK9, CFTR, CDK6, PRKCE, MMP14, CDK5, CDK2, DAPK1, CCND1, FOLH1, G6PD, ERN1, ADAM17, MDM2, MTAP, CA2, CA1, ALOX12, PREP, KMT2A, HSD17B1, TH, EGLN3, NR3C1, MIF, TYMS, PTK2B, FASN, APEX1, GAPDH, PIK3R1, NOS1, MAPK10, FUCA1, STAT3, CDC25A, CAPN1, CCNB1, RPS6KA3, GSK3B, MAPK14, DPYD, CALM1 |
| CC | cytosol | 112 | 42.74809 | 2.73E-20 | FHIT, HRAS, LDHA, SLC6A4, AURKA, AURKB, PNP, SLC7A5, CTNNB1, BTK, APP, CSNK2A1, PIK3CA, NQO1, SYK, PIK3CG, BRAF, ROCK1, PIK3CB, RELA, PIK3CD, FBP1, MAPK1, AKR1B10, PDE5A, NFE2L2, EIF2AK2, XDH, MCL1, GRB2, HSPA1A, CHEK1, ADA, SRC, ARG1, PTK2, CDA, GLO1, PTPN6, MAP2K1, FDPS, TKT, HDAC3, DHFR, PPIA, PLK1, IKBKG, FABP4, MTOR, PTPN1, IKBKB, HDAC6, HSP90AB1, PPARG, ANPEP, PDCD4, AKR1C3, AKT1, CCNE1, PRMT1, CASP3, PRMT7, RHOA, NOS2, CHUK, AKR1C1, PRKCA, CDK1, AR, HSP90AA1, CFTR, CDK6, PRKCE, CDK4, CDK5, CDK2, ALOX15, CCND1, G6PD, LCK, MDM2, MTAP, NGFR, CA2, CA1, ALOX12, HSD17B1, TH, EGLN3, TYMS, PTK2B, FASN, GAPDH, HSPA8, PIK3R1, NOS1, FLT3, MAPK10, TPMT, STAT3, CDC25A, CAPN1, CCNB1, RPS6KA3, CCNB2, FYN, MAPK14, GSK3B, ALOX5, JAK3, DPYD, CALM1 |
| CC | extracellular exosome | 86 | 32.82443 | 2.90E-12 | HSP90AB1, FHIT, LDHA, THRB, MMP9, PTGS1, MMP7, ANPEP, PNP, SLC7A5, CTNNB1, AKR1C3, TTR, APP, SLC16A1, SERPINE1, RHOA, NQO1, DPP4, TUBB3, AKR1C1, NQO2, PRKCA, SHBG, CDK1, HSP90AA1, ELANE, FBP1, CFTR, CD38, MAPK1, FOLH1, G6PD, F3, AKR1B10, F2, CD81, LCK, PLA2G2A, PDGFRB, MTAP, CTSD, CA2, CA1, ALOX12, ITGA2B, ALPL, GRB2, PRSS1, MME, ITGB3, ITGB1, SRC, MIF, ARG1, ACE, ECE1, GSTK1, FASN, GLO1, HSPA5, GAPDH, HSPA8, GBA, PTPRC, PTPN6, ST6GAL1, MAP2K1, GUSB, ABCB1, TKT, ITGA4, TPMT, FUCA1, PLG, CAPN1, NRAS, PPIA, MAPK14, ALDH2, FABP4, MPO, ABCC1, IGFBP3, PLAU, CALM1 |
| CC | nucleoplasm | 81 | 30.91603 | 2.22E-10 | HSP90AB1, PPARA, PPARD, THRB, EZH2, PPARG, AURKA, RORA, AURKB, PDCD4, CTNNB1, PGR, AKT1, TOP1, KDM1A, CCNE1, CASP3, PRMT1, CSNK2A1, PRMT7, TOP2A, TERT, CHUK, NQO2, PRKCA, CDK1, AR, HSP90AA1, RELA, ESR1, CDK9, POLB, CDK6, ESR2, CDK4, CDK5, AHR, CDK2, MAPK1, CCND1, EP300, MDM2, NGFR, MCL1, KMT2A, GRB2, HSD17B1, MGMT, EGLN3, CHEK1, HSPA1A, NR3C1, MIF, TYMS, VDR, PTK2B, APEX1, DNMT3B, NR1H4, HSPA8, ESRRA, EHMT1, FDPS, TKT, MAPK10, EHMT2, STAT3, CDC25A, CCNB1, HDAC3, RPS6KA3, CCNB2, DHFR, PLK1, MAPK14, DNMT1, KDM4C, MTOR, SETD2, PARP1, CALM1 |
| CC | membrane | 70 | 26.71756 | 1.49E-10 | HSP90AB1, HRAS, LDHA, PGF, CXCR2, SLC7A5, CTNNB1, SLC16A1, PTGES, DPP4, PIK3CG, EGFR, CDK1, RET, HSP90AA1, ESR1, CDK9, CFTR, CDK5, CD38, ALOX15, CCND1, FOLH1, G6PD, CD81, VEGFA, HSD11B1, PDGFRA, PDGFRB, ADAM17, KCNH2, EIF2AK2, ALOX12, ALPL, PREP, ACHE, TNF, MCL1, ERBB2, DRD4, MGMT, KIT, ITGB1, ADA, SLC29A1, IGF1R, ACE, ECE1, BCHE, GSTK1, FASN, HSPA5, GAPDH, HSPA8, PIK3R1, CYP19A1, PTPRC, PTPN6, GUSB, ABCB1, ITGA4, CAPN1, CCNB1, NRAS, CCNB2, PPIA, ABCC1, JAK3, MTOR, PARP1 |
| CC | integral component of plasma membrane | 44 | 16.79389 | 2.03E-06 | OPRM1, AVPR2, CASR, TNF, TACR1, SLC6A4, DRD4, MME, CXCR2, TLR4, ANPEP, ITGB3, SLC7A5, EDNRA, SLC29A1, EDNRB, IGF1R, SLC16A1, APP, HTR1A, PTGER1, PTPRC, PTGER2, RET, GABRA1, PTGER3, FLT1, PTGER4, FLT3, MET, MMP14, GRM1, KDR, SSTR5, FOLH1, ADRB2, ADRB1, CD81, PDGFRA, ADAM17, ABCC1, NGFR, SLC5A7, ITGA2B |
| CC | extracellular space | 41 | 15.64885 | 8.45E-06 | ALPL, XDH, ACHE, TNF, PGF, MMP9, MMP8, MMP7, ANPEP, CHEK1, KIT, MMP3, MMP2, ADA, MIF, TTR, ARG1, ACE, APP, REN, SERPINE1, HSPA8, GBA, EGFR, FLT1, GUSB, ELANE, MMP13, PLG, PPIA, F3, F2, VEGFA, PLA2G2A, MPO, CTSD, ALOX5, CA2, IGFBP3, PLAU, IL2 |
| CC | extracellular region | 40 | 15.26718 | 8.07E-04 | ACHE, TNF, PGF, MMP9, MMP8, MMP7, PRSS1, MMP3, PNP, MMP2, MMP1, MIF, TLR9, TTR, ACE, APP, BCHE, REN, SERPINE1, CDA, SHBG, F10, HSP90AA1, ELANE, MET, ESR2, MMP13, PLG, MMP12, KDR, PPIA, F2, VEGFA, PLA2G2A, CTSD, NGFR, IGFBP3, PLAU, CALM1, IL2 |
| CC | mitochondrion | 36 | 13.74046 | 3.56E-04 | HSP90AB1, FHIT, CYP1B1, MCL1, TH, HSPA1A, MMP2, SRC, AKT1, TYMS, SLC16A1, GSTK1, FASN, HSPA5, APEX1, PRKCA, NOX4, CDK1, NOS1, MAP2K1, BRAF, CYP1A1, FDPS, MAPK10, ESR2, PRKCE, SIRT3, CAPN1, MAPK1, CYP17A1, FYN, GSK3B, MAPK14, ERN1, MPO, PARP1 |
| CC | cell surface | 32 | 12.21374 | 5.47E-11 | HSP90AB1, ACHE, TNF, MMP7, CXCR2, TLR4, ITGB3, ITGB1, ADA, MIF, APP, HSPA5, DPP4, EGFR, PTPRC, HSP90AA1, ELANE, MET, ABCB1, CFTR, ITGA4, PLG, CD38, FOLH1, F3, VEGFA, ADAM17, PDGFRB, NGFR, KCNH2, PLAU, ITGA2B |
| CC | perinuclear region of cytoplasm | 31 | 11.83206 | 6.16E-09 | HRAS, ACHE, ERBB2, PPARG, TLR4, HSPA1A, AURKA, ITGB1, SRC, BTK, CTNNB1, APP, ECE1, PTK2B, PTGES, SRD5A1, NOS2, GAPDH, APEX1, PRKCA, NOX4, EGFR, HSP90AA1, NOS1, PRKCE, CDK4, GSK3B, PLA2G2A, KCNH2, EIF2AK2, HDAC6 |
| CC | focal adhesion | 27 | 10.30534 | 8.51E-11 | OPRM1, NOX4, EGFR, PTPRC, FLT1, MAP2K1, MME, HSPA1A, ITGA4, ITGB3, MMP14, ITGB1, CAPN1, CTNNB1, MAPK1, PTK2, PPIA, PTK2B, CD81, RHOA, PDGFRB, ADAM17, HSPA5, PLAU, DPP4, HSPA8, ITGA2B |
| CC | endoplasmic reticulum membrane | 25 | 9.541985 | 0.001481 | CYP3A4, CYP1B1, PTGS2, CYP2C19, HMGCR, PTGS1, TLR9, PTGES, RHOA, HSD17B3, SRD5A1, HSPA5, CYP19A1, NOX4, EGFR, CYP1A1, TRPM8, CYP2C9, CYP1A2, CYP17A1, HSD11B1, PLA2G2A, ERN1, HSD11B2, MTOR |
| CC | intracellular membrane-bounded organelle | 24 | 9.160305 | 5.88E-06 | CYP3A4, HRAS, RET, CYP1B1, CYP2C19, BRAF, CYP1A1, CYP2C9, GUSB, PPARG, PTGS1, CDK9, CHEK1, CYP1A2, SLC7A5, BTK, IGF1R, APP, G6PD, GSTK1, HSD17B3, DNMT3B, GAPDH, CHUK |
| CC | membrane raft | 21 | 8.015267 | 1.78E-11 | OPRM1, EGFR, PTPRC, RET, TNF, NOS1, TRPM8, SLC6A4, ITGB1, KDR, BTK, EDNRB, CASP3, APP, FYN, PTK2B, LCK, ADAM17, CTSD, IKBKB, DPP4 |
| CC | protein complex | 19 | 7.251908 | 2.94E-05 | AR, NOS1, HSP90AA1, PTGS2, FLT3, POLB, CFTR, NR3C1, CTNNB1, AKT1, KDM1A, MAPK1, TTR, PDGFRA, MDM2, PARP1, TOP2A, SYK, HDAC6 |
| CC | apical plasma membrane | 15 | 5.725191 | 8.29E-05 | NOX4, HSP90AB1, EGFR, HSP90AA1, ERBB2, CFTR, ABCB1, SLC7A5, TLR9, SLC29A1, PTK2, ADRB2, PDGFRB, ADAM17, DPP4 |
| CC | centrosome | 15 | 5.725191 | 0.003488 | CDK1, AURKA, CHEK1, CDK6, CDK2, CTNNB1, CCNB1, SLC16A1, G6PD, CCNB2, PLK1, GSK3B, NFE2L2, APEX1, CALM1 |
| CC | endosome | 14 | 5.343511 | 2.29E-05 | EGFR, AVPR2, FLT1, GRB2, CDK2, KDR, TLR9, APP, ACE, ADRB2, ECE1, FYN, RHOA, NGFR |
| CC | extracellular matrix | 13 | 4.961832 | 0.00121 | HSP90AA1, F3, SERPINE1, MMP8, MMP7, CTSD, HSPA5, MMP14, MMP13, MMP2, GAPDH, HSPA8, MMP1 |
| CC | neuronal cell body | 12 | 4.580153 | 0.005916 | ARG1, CYP17A1, RET, HSP90AA1, PTK2B, GSK3B, SRD5A1, MTOR, NQO1, CDK5, TUBB3, ADA |
| CC | cell-cell adherens junction | 12 | 4.580153 | 0.007082 | HSP90AB1, EGFR, LDHA, CCNB2, FASN, HSPA1A, PTPN1, HSPA5, ITGB1, SRC, HSPA8, CTNNB1 |
| CC | basolateral plasma membrane | 11 | 4.198473 | 2.77E-04 | SLC29A1, HSP90AB1, EGFR, HSP90AA1, CA9, ERBB2, ABCC1, CFTR, CA2, CTNNB1, TLR9 |
| CC | external side of plasma membrane | 11 | 4.198473 | 0.00103 | PTPRC, ACE, TNF, ECE1, TRPM8, TLR4, ANPEP, KIT, ITGB1, ADA, ITGA2B |
| CC | axon | 11 | 4.198473 | 0.001406 | MAPK1, CYP17A1, APP, RET, PTK2B, MME, CA2, SLC5A7, CDK5, TUBB3, HDAC6 |
| CC | lysosome | 11 | 4.198473 | 0.001605 | ACE, ADRB2, REN, AKR1B10, MPO, CTSD, MTOR, SRC, ADA, TLR9, CAPN1 |
| CC | extrinsic component of cytoplasmic side of plasma membrane | 10 | 3.816794 | 5.21E-07 | PTK2, ALOX15, FYN, PTK2B, LCK, RHOA, JAK3, SRC, SYK, BTK |
| CC | receptor complex | 10 | 3.816794 | 9.00E-05 | EGFR, VDR, IGF1R, APP, ADRB2, RET, FLT1, ERBB2, ITGB3, ITGB1 |
| CC | nuclear chromatin | 10 | 3.816794 | 0.001904 | KDM1A, AR, PPARD, THRB, RELA, EZH2, ESR1, KDM4C, EHMT2, STAT3 |
| CC | neuron projection | 10 | 3.816794 | 0.007325 | OPRM1, ARG1, SSTR5, HSP90AA1, BRAF, PTGS2, SLC6A4, TH, NGFR, SRC |
| CC | organelle membrane | 9 | 3.435115 | 3.50E-05 | CYP3A4, CYP1B1, CYP1A1, CYP2C19, PTGS2, CYP2C9, PTGS1, SRD5A1, CYP1A2 |
| CC | melanosome | 9 | 3.435115 | 1.02E-04 | HSP90AB1, HSP90AA1, FASN, CTSD, HSPA5, ITGB3, MMP14, ITGB1, HSPA8 |
| CC | lamellipodium | 9 | 3.435115 | 0.002186 | PTK2, ROCK1, PTK2B, RHOA, PIK3CA, CDK5, ITGB1, DPP4, CTNNB1 |
| CC | transcription factor complex | 9 | 3.435115 | 0.006769 | KDM1A, EP300, RELA, PARP1, CDK4, APEX1, AHR, CDK2, CTNNB1 |
| CC | vesicle | 8 | 3.053435 | 0.002479 | AKT1, ECE1, CD81, RHOA, TKT, GAPDH, CALM1, MIF |
| CC | myelin sheath | 8 | 3.053435 | 0.00636 | HSP90AA1, ERBB2, SRD5A1, TKT, HSPA5, CA2, HSPA8, MIF |
| CC | spindle microtubule | 7 | 2.671756 | 3.64E-05 | CDK1, HDAC3, PLK1, AURKA, POLB, AURKB, CALM1 |
| CC | lysosomal lumen | 7 | 2.671756 | 0.001385 | HSP90AA1, GUSB, PDGFRB, CTSD, FUCA1, HSPA8, GBA |
| CC | perikaryon | 7 | 2.671756 | 0.004216 | OPRM1, MAPK1, TOP1, TH, SLC5A7, CDK5, HDAC6 |
| CC | phosphatidylinositol 3-kinase complex | 6 | 2.290076 | 1.04E-06 | PIK3CG, PIK3CB, PIK3CD, PIK3CA, MTOR, PIK3R1 |
| CC | cyclin-dependent protein kinase holoenzyme complex | 5 | 1.908397 | 4.96E-05 | CCNE1, CCND1, CDK6, CDK4, CDK2 |
| CC | mast cell granule | 5 | 1.908397 | 2.03E-04 | PIK3CG, PIK3CD, CXCR2, KIT, BTK |
| CC | postsynapse | 5 | 1.908397 | 2.45E-04 | SLC29A1, OPRM1, AKT1, GSK3B, DRD4 |
| CC | cytoplasmic side of plasma membrane | 5 | 1.908397 | 0.003001 | G6PD, TH, KIT, IKBKB, CHUK |
| CC | nuclear envelope lumen | 4 | 1.526718 | 1.54E-04 | APP, BCHE, PTGES, ALOX5 |
| CC | spindle midzone | 4 | 1.526718 | 0.002374 | APP, PLK1, AURKA, AURKB |
| CC | integrin complex | 4 | 1.526718 | 0.006588 | ITGA4, ITGB3, ITGB1, ITGA2B |
| CC | IkappaB kinase complex | 3 | 1.145038 | 0.008524 | IKBKG, IKBKB, CHUK |
| MF | protein binding | 200 | 76.33588 | 4.04E-16 | FHIT, HRAS, LDHA, PTGS2, PGF, AURKA, RORA, AURKB, BTK, CTNNB1, PGR, APP, HTR1A, SERPINE1, PIK3CA, SYK, PIK3CG, F10, ROCK1, BRAF, CYP1A1, PIK3CB, PIK3CD, PIM1, POLB, MAPK1, F3, F2, VEGFA, PDGFRA, PDGFRB, EIF2AK2, ITGA2B, ERBB2, MME, CHEK1, HSPA1A, ADA, CDA, DNMT3B, NR1H4, ST6GAL1, ESRRA, MAP2K1, TKT, ABCG2, KDR, PARP1, PLAU, OPRM1, PPARA, PPARD, EZH2, PPARG, EDNRA, EDNRB, CCNE1, NOS2, TOP2A, TUBB3, DPP4, AR, CTBP2, ELANE, CFTR, MMP14, GRM1, CCND1, ALOX15, G6PD, MTAP, ADAM17, KCNH2, ALOX12, ALPL, PREP, AVPR2, TH, KIT, MIF, PTK2B, FASN, NOS1, MAPK10, PLG, STAT3, CAPN1, CCNB1, RPS6KA3, CCNB2, FYN, MAPK14, GSK3B, DPYD, CALM1, CASR, THRB, HMGCR, MMP9, SLC6A4, TLR4, MMP3, MMP2, KDM1A, TOP1, TTR, CSNK2A1, NQO1, NQO2, EGFR, RET, RELA, FBP1, AHR, EP300, AKR1B10, NFE2L2, GLP1R, ACHE, MCL1, GRB2, DRD4, ITGB3, ITGB1, SRC, VDR, PTK2, ECE1, GSTK1, PTPN6, EHMT1, MET, ABCB1, ITGA4, EHMT2, HDAC3, PPIA, PLK1, IKBKG, DNMT1, MTOR, PTPN1, IKBKB, HDAC6, HSP90AB1, TACR1, CXCR2, PDCD4, AKT1, PRMT1, CASP3, RHOA, TERT, AKR1C1, CHUK, PRKCA, CDK1, HSP90AA1, TRPM8, PTGER4, ESR1, CDK9, CDK6, ESR2, PRKCE, CDK4, CDK5, CDK2, DAPK1, ADRB2, ADRB1, CD81, LCK, ERN1, MDM2, CTSD, CA2, NGFR, CA1, TNF, KMT2A, EGLN3, NR3C1, IGF1R, HSPA5, GAPDH, APEX1, HSPA8, PIK3R1, GBA, NOX4, PTPRC, FLT1, FLT3, CDC25A, SIRT3, JAK3, ALOX5, SETD2, IGFBP3 |
| MF | ATP binding | 62 | 23.66412 | 1.14E-12 | HSP90AB1, AURKA, AURKB, BTK, AKT1, CSNK2A1, PIK3CA, TOP2A, CHUK, SYK, EGFR, PRKCA, PIK3CG, CDK1, RET, HSP90AA1, ROCK1, BRAF, PIK3CB, PIK3CD, PIM1, CDK9, CFTR, CDK6, PRKCE, CDK4, CDK5, CDK2, DAPK1, MAPK1, LCK, PDGFRA, ERN1, PDGFRB, EIF2AK2, ERBB2, CHEK1, HSPA1A, KIT, SRC, IGF1R, PTK2, PTK2B, HSPA5, HSPA8, FLT1, MAP2K1, FLT3, MET, ABCB1, MAPK10, ABCG2, KDR, RPS6KA3, FYN, PLK1, GSK3B, MAPK14, ABCC1, JAK3, MTOR, IKBKB |
| MF | zinc ion binding | 43 | 16.41221 | 2.49E-07 | PPARA, PPARD, THRB, KMT2A, MMP9, MMP8, PPARG, MMP7, MME, ANPEP, NR3C1, RORA, MMP3, MMP2, MMP1, ADA, PGR, VDR, ACE, CDA, GLO1, NR1H4, PRKCA, AR, ESRRA, EHMT1, ESR1, ESR2, MMP14, EHMT2, MMP13, MMP12, SIRT3, EP300, CA9, KDM4C, MDM2, DNMT1, CA2, PTPN1, PARP1, CA1, HDAC6 |
| MF | enzyme binding | 41 | 15.64885 | 2.83E-24 | CYP3A4, CYP2C19, THRB, PTGS2, TH, PPARG, HSPA1A, ITGB3, SRC, CTNNB1, AKT1, PGR, KDM1A, APP, PRMT1, BCHE, HSPA5, TOP2A, HSPA8, PRKCA, EGFR, AR, CYP1A1, ESR1, CFTR, POLB, ESR2, CYP1A2, PRKCE, SIRT3, CCND1, HDAC3, FYN, MAPK14, ERN1, KDM4C, PDGFRB, MDM2, PTPN1, PARP1, HDAC6 |
| MF | identical protein binding | 38 | 14.50382 | 3.81E-10 | FHIT, LDHA, TNF, KMT2A, GRB2, MMP9, ERBB2, DRD4, PPARG, ITGB3, BTK, AKT1, TTR, IGF1R, APP, PRMT1, BCHE, NQO1, GAPDH, DPP4, EGFR, HSP90AA1, BRAF, RELA, FBP1, ESR1, STAT3, DAPK1, MAPK1, G6PD, FYN, LCK, VEGFA, ERN1, MDM2, PARP1, KCNH2, ITGA2B |
| MF | protein kinase activity | 36 | 13.74046 | 2.25E-18 | ERBB2, CHEK1, AURKA, AURKB, SRC, BTK, AKT1, PTK2, CSNK2A1, CHUK, SYK, PRKCA, PIK3CG, EGFR, CDK1, RET, ROCK1, BRAF, MAP2K1, MET, CDK9, MAPK10, PRKCE, CDK4, CDK5, CDK2, DAPK1, CCND1, RPS6KA3, PLK1, MAPK14, GSK3B, PDGFRA, MTOR, IKBKB, EIF2AK2 |
| MF | protein homodimerization activity | 35 | 13.35878 | 9.37E-09 | XDH, ACHE, MCL1, PTGS2, KMT2A, HMGCR, PGF, SLC6A4, KIT, TYMS, SLC16A1, ECE1, FASN, CDA, NOS2, TOP2A, DPP4, CHUK, TERT, HSP90AA1, CTBP2, TRPM8, FLT3, RELA, TKT, ABCG2, ADRB2, G6PD, IKBKG, VEGFA, ERN1, PDGFRA, DPYD, IKBKB, KCNH2 |
| MF | protein serine/threonine kinase activity | 29 | 11.0687 | 6.09E-12 | CHEK1, AURKA, AURKB, AKT1, CSNK2A1, PIK3CA, SYK, PRKCA, PIK3CG, CDK1, BRAF, MAP2K1, ROCK1, PIM1, CDK9, PRKCE, CDK4, CDK5, CDK2, DAPK1, MAPK1, RPS6KA3, PLK1, MAPK14, GSK3B, ERN1, MTOR, IKBKB, EIF2AK2 |
| MF | kinase activity | 28 | 10.68702 | 7.81E-16 | ERBB2, CHEK1, SRC, BTK, AKT1, CCNE1, CSNK2A1, PIK3CA, PIK3R1, PRKCA, PIK3CG, RET, BRAF, PIK3CB, PIK3CD, CDK9, CDK5, CDK2, DAPK1, MAPK1, RPS6KA3, PLK1, GSK3B, LCK, IKBKG, MTOR, PLAU, CALM1 |
| MF | protein kinase binding | 27 | 10.30534 | 1.85E-10 | HSP90AB1, GRB2, AURKA, CTNNB1, CCNE1, PTK2, SYK, EGFR, PTPN6, PTPRC, MAP2K1, RELA, CDK5, CDC25A, STAT3, CCNB1, RPS6KA3, CCND1, PLK1, GSK3B, LCK, PDGFRB, PTPN1, MTOR, PARP1, IKBKB, CALM1 |
| MF | receptor binding | 20 | 7.633588 | 2.62E-06 | AR, GUSB, HSPA1A, PLG, SRC, BTK, MIF, PGR, PTK2, APP, PTK2B, GSTK1, REN, F2, SERPINE1, PDGFRB, NOS2, JAK3, DPP4, GBA |
| MF | serine-type endopeptidase activity | 19 | 7.251908 | 9.48E-08 | PREP, F10, MMP9, MMP8, ELANE, PRSS1, MMP7, MMP3, MMP14, MMP13, MMP2, PLG, MMP12, MMP1, F3, F2, CTSD, PLAU, DPP4 |
| MF | protein tyrosine kinase activity | 19 | 7.251908 | 2.30E-12 | EGFR, RET, HSP90AA1, FLT1, MAP2K1, ERBB2, MET, KIT, SRC, KDR, BTK, IGF1R, PTK2, FYN, PTK2B, LCK, PDGFRB, JAK3, SYK |
| MF | protein heterodimerization activity | 18 | 6.870229 | 9.47E-04 | EGFR, PPARD, BRAF, MCL1, PGF, ERBB2, RELA, ITGB1, AHR, CTNNB1, TTR, ADRB1, IKBKG, VEGFA, IKBKB, TOP2A, CHUK, PIK3R1 |
| MF | chromatin binding | 18 | 6.870229 | 1.31E-04 | EGFR, CDK1, AR, CTBP2, KMT2A, RELA, EZH2, PPARG, ESR1, CDK9, KDM1A, TOP1, HDAC3, EP300, DNMT1, MPO, DNMT3B, TOP2A |
| MF | transcription factor binding | 18 | 6.870229 | 2.17E-06 | PPARA, AR, PPARD, RELA, PPARG, PIM1, ESR1, RORA, STAT3, AHR, CTNNB1, KDM1A, MAPK1, CCND1, HDAC3, EP300, PARP1, PIK3R1 |
| MF | oxidoreductase activity | 15 | 5.725191 | 2.88E-06 | NOX4, AKR1C3, CYP3A4, KDM1A, CYP2C19, CYP1A1, CYP2C9, ALDH2, HSD11B1, EGLN3, FASN, CYP1A2, APEX1, AKR1C1, NQO2 |
| MF | iron ion binding | 15 | 5.725191 | 1.11E-07 | XDH, CYP3A4, NOS1, CYP1B1, CYP1A1, CYP2C19, CYP2C9, TH, EGLN3, CYP1A2, CYP17A1, ALOX15, ALOX5, CYP19A1, ALOX12 |
| MF | heme binding | 15 | 5.725191 | 2.74E-08 | NOX4, CYP3A4, CYP17A1, CYP1B1, NOS1, CYP1A1, CYP2C19, PTGS2, CYP2C9, PTGS1, MPO, CYP1A2, NOS2, SRC, CYP19A1 |
| MF | ubiquitin protein ligase binding | 13 | 4.961832 | 0.001754 | EGFR, FHIT, RELA, GSK3B, IKBKG, MDM2, AURKA, HSPA1A, NGFR, HSPA5, KCNH2, HSPA8, HDAC6 |
| MF | Ras guanyl-nucleotide exchange factor activity | 13 | 4.961832 | 2.18E-07 | EGFR, PTK2, RET, ADRB1, FYN, GRB2, ERBB2, PDGFRA, PDGFRB, KIT, JAK3, CALM1, IL2 |
| MF | drug binding | 13 | 4.961832 | 1.87E-09 | HSP90AB1, TYMS, PPARA, ACE, PPARD, GABRA1, DHFR, CYP2C9, DRD4, PPARG, FASN, TOP2A, PNP |
| MF | phosphatidylinositol-4,5-bisphosphate 3-kinase activity | 13 | 4.961832 | 1.58E-10 | PIK3CG, EGFR, GRB2, PIK3CB, ERBB2, PIK3CD, KIT, FYN, LCK, PDGFRA, PIK3CA, PDGFRB, PIK3R1 |
| MF | steroid hormone receptor activity | 13 | 4.961832 | 4.43E-11 | PPARA, PPARD, ESRRA, AR, THRB, PPARG, ESR1, NR3C1, ESR2, RORA, PGR, VDR, NR1H4 |
| MF | cadherin binding involved in cell-cell adhesion | 12 | 4.580153 | 0.005674 | HSP90AB1, EGFR, LDHA, CCNB2, FASN, HSPA1A, PTPN1, HSPA5, ITGB1, SRC, HSPA8, CTNNB1 |
| MF | metalloendopeptidase activity | 12 | 4.580153 | 1.41E-06 | ECE1, MMP9, MMP8, MMP7, ADAM17, MME, MMP14, MMP3, MMP13, MMP2, MMP12, MMP1 |
| MF | protein phosphatase binding | 12 | 4.580153 | 2.96E-09 | EGFR, HSP90AA1, GRB2, MAPK14, ERBB2, LCK, PPARG, MET, JAK3, PIK3R1, STAT3, CTNNB1 |
| MF | protein domain specific binding | 11 | 4.198473 | 0.00152 | OPRM1, PPARA, ESRRA, GUSB, IKBKG, TH, HSPA5, MTOR, NFE2L2, PLG, CALM1 |
| MF | protein complex binding | 11 | 4.198473 | 0.001411 | NRAS, PPARA, CASP3, CCND1, FLT3, PTK2B, RELA, NR3C1, CDK4, APEX1, ITGB1 |
| MF | RNA polymerase II transcription factor activity, ligand-activated sequence-specific DNA binding | 11 | 4.198473 | 1.17E-10 | PPARA, AR, ESRRA, PPARD, PPARG, ESR1, RORA, ESR2, AHR, STAT3, NR1H4 |
| MF | transcription regulatory region DNA binding | 10 | 3.816794 | 0.006005 | KDM1A, AR, TNF, KMT2A, RELA, PPARG, NFE2L2, AHR, STAT3, CTNNB1 |
| MF | histone deacetylase binding | 10 | 3.816794 | 2.88E-05 | HSP90AB1, HDAC3, CCND1, HSP90AA1, RELA, HSPA1A, PARP1, DNMT3B, TOP2A, HDAC6 |
| MF | transmembrane receptor protein tyrosine kinase activity | 10 | 3.816794 | 4.85E-09 | EGFR, IGF1R, RET, FLT1, FLT3, ERBB2, MET, PDGFRA, KIT, KDR |
| MF | protein C-terminus binding | 9 | 3.435115 | 0.00746 | OPRM1, HRAS, EP300, MAP2K1, ERBB2, LCK, TOP2A, SRC, CTNNB1 |
| MF | protease binding | 9 | 3.435115 | 1.72E-04 | CASP3, TNF, F3, ELANE, SERPINE1, KIT, ITGB3, ITGB1, DPP4 |
| MF | oxygen binding | 9 | 3.435115 | 5.48E-07 | CYP3A4, CYP17A1, CYP1B1, CYP1A1, CYP2C19, CYP2C9, TH, CYP1A2, CYP19A1 |
| MF | non-membrane spanning protein tyrosine kinase activity | 9 | 3.435115 | 4.60E-07 | PTK2, FYN, PTK2B, LCK, JAK3, EIF2AK2, SRC, SYK, BTK |
| MF | steroid binding | 9 | 3.435115 | 5.05E-09 | SHBG, PGR, CYP3A4, AR, ESRRA, ESR1, HSD11B2, NR3C1, ESR2 |
| MF | endopeptidase activity | 8 | 3.053435 | 1.86E-05 | ACE, ECE1, MMP9, ELANE, MME, MMP3, MMP12, MMP1 |
| MF | cyclin-dependent protein serine/threonine kinase activity | 8 | 3.053435 | 7.34E-07 | CCNB1, CDK1, CCNB2, CDK9, CDK6, CDK4, CDK5, CDK2 |
| MF | ion channel binding | 7 | 2.671756 | 0.008231 | HSP90AB1, HSP90AA1, NOS1, FYN, SRC, CTNNB1, CALM1 |
| MF | integrin binding | 7 | 2.671756 | 0.005803 | EGFR, ADAM17, MMP14, ITGB1, SRC, KDR, SYK |
| MF | electron carrier activity | 7 | 2.671756 | 0.002717 | NOX4, XDH, ALDH2, SRD5A1, CYP1A2, CYP19A1, NQO2 |
| MF | metallopeptidase activity | 7 | 2.671756 | 0.001589 | ACE, MMP9, ADAM17, MME, ANPEP, MMP3, MMP2 |
| MF | p53 binding | 7 | 2.671756 | 5.85E-04 | KDM1A, EHMT1, EP300, GSK3B, MDM2, EHMT2, CDK5 |
| MF | monooxygenase activity | 7 | 2.671756 | 2.66E-04 | CYP3A4, CYP1B1, CYP1A1, CYP2C19, CYP2C9, CYP1A2, CYP19A1 |
| MF | 1-phosphatidylinositol-3-kinase activity | 7 | 2.671756 | 4.87E-05 | PIK3CG, PIK3CB, GRB2, PIK3CD, PIK3CA, PIK3R1, TLR9 |
| MF | NADP binding | 7 | 2.671756 | 1.44E-05 | G6PD, DHFR, NOS1, HMGCR, NOS2, DPYD, GAPDH |
| MF | beta-catenin binding | 6 | 2.290076 | 0.008827 | AR, EP300, GSK3B, ESR1, RORA, HDAC6 |
| MF | glycoprotein binding | 6 | 2.290076 | 0.0033 | HSP90AB1, EGFR, HSP90AA1, FYN, LCK, HSPA5 |
| MF | flavin adenine dinucleotide binding | 6 | 2.290076 | 0.003084 | NOX4, XDH, KDM1A, NOS1, NOS2, DPYD |
| MF | chromatin DNA binding | 6 | 2.290076 | 0.001998 | EP300, THRB, RELA, EZH2, APEX1, STAT3 |
| MF | oxidoreductase activity, acting on paired donors, with incorporation or reduction of molecular oxygen | 6 | 2.290076 | 0.001848 | CYP3A4, CYP1B1, CYP2C19, CYP2C9, EGLN3, CYP19A1 |
| MF | scaffold protein binding | 6 | 2.290076 | 8.46E-04 | NOS1, MDM2, KCNH2, IKBKB, SRC, CHUK |
| MF | core promoter sequence-specific DNA binding | 6 | 2.290076 | 5.06E-04 | TOP1, KMT2A, PPARG, ESR1, RORA, ESR2 |
| MF | ephrin receptor binding | 6 | 2.290076 | 4.29E-05 | PIK3CG, FYN, GRB2, PTPN1, CDK5, SRC |
| MF | nitric-oxide synthase regulator activity | 6 | 2.290076 | 4.59E-08 | HSP90AB1, AKT1, EGFR, HSP90AA1, ESR1, CALM1 |
| MF | androgen receptor binding | 5 | 1.908397 | 0.003296 | KDM1A, CCNE1, EP300, KDM4C, CTNNB1 |
| MF | histone-lysine N-methyltransferase activity | 5 | 1.908397 | 0.003002 | EHMT1, KMT2A, EZH2, SETD2, EHMT2 |
| MF | NAD binding | 5 | 1.908397 | 0.002727 | LDHA, CTBP2, HSD11B2, PARP1, GAPDH |
| MF | NF-kappaB binding | 5 | 1.908397 | 0.001115 | HDAC3, PPARD, RELA, GSK3B, APEX1 |
| MF | Hsp90 protein binding | 5 | 1.908397 | 7.41E-04 | CSNK2A1, ERN1, AHR, KDR, HDAC6 |
| MF | aromatase activity | 5 | 1.908397 | 7.41E-04 | CYP3A4, CYP1B1, CYP1A1, CYP1A2, CYP19A1 |
| MF | fibronectin binding | 5 | 1.908397 | 6.39E-04 | VEGFA, ITGA4, ITGB3, IGFBP3, ITGB1 |
| MF | steroid hydroxylase activity | 5 | 1.908397 | 6.39E-04 | CYP3A4, CYP1A1, CYP2C19, CYP2C9, CYP19A1 |
| MF | cyclin binding | 5 | 1.908397 | 2.23E-04 | CDK1, HDAC3, CDK6, CDK4, CDK2 |
| MF | phosphatidylinositol 3-kinase binding | 5 | 1.908397 | 1.80E-04 | IGF1R, FYN, LCK, PDGFRB, PIK3R1 |
| MF | platelet-derived growth factor receptor binding | 5 | 1.908397 | 6.67E-05 | VEGFA, PDGFRA, ERN1, PDGFRB, ITGB3 |
| MF | oxidoreductase activity, acting on paired donors, with incorporation or reduction of molecular oxygen, reduced flavin or flavoprotein as one donor, and incorporation of one atom of oxygen | 5 | 1.908397 | 6.67E-05 | CYP3A4, CYP1B1, CYP1A1, CYP1A2, CYP19A1 |
| MF | insulin receptor substrate binding | 5 | 1.908397 | 1.69E-05 | IGF1R, PIK3CB, GRB2, PIK3CA, PIK3R1 |
| MF | receptor signaling protein tyrosine kinase activity | 5 | 1.908397 | 1.09E-05 | EGFR, ERBB2, KIT, KDR, SYK |
| MF | protein serine/threonine/tyrosine kinase activity | 4 | 1.526718 | 0.009922 | AKT1, MAP2K1, AURKA, AURKB |
| MF | SH2 domain binding | 4 | 1.526718 | 0.009922 | PTPN6, PTK2, LCK, SRC |
| MF | MHC class II protein complex binding | 4 | 1.526718 | 0.001763 | HSP90AB1, HSP90AA1, CD81, HSPA8 |
| MF | NADPH binding | 4 | 1.526718 | 0.001449 | DHFR, HMGCR, FASN, SRD5A1 |
| MF | transcription coactivator binding | 4 | 1.526718 | 4.05E-04 | PPARA, CDK9, RORA, TERT |
| MF | vascular endothelial growth factor-activated receptor activity | 4 | 1.526718 | 1.22E-04 | FLT1, FLT3, PDGFRA, KDR |
| MF | 1-phosphatidylinositol-4-phosphate 3-kinase activity | 4 | 1.526718 | 1.22E-04 | PIK3CG, PIK3CB, PIK3CD, PIK3CA |
| MF | prostaglandin E receptor activity | 4 | 1.526718 | 3.57E-05 | PTGER1, PTGER2, PTGER3, PTGER4 |
| MF | phosphatidylinositol 3-kinase activity | 4 | 1.526718 | 3.57E-05 | PIK3CG, PIK3CB, PIK3CD, PIK3CA |
| MF | testosterone dehydrogenase (NAD+) activity | 3 | 1.145038 | 0.004751 | AKR1C3, HSD17B1, HSD17B3 |
| MF | C3HC4-type RING finger domain binding | 3 | 1.145038 | 0.003429 | HSPA1A, KCNH2, HSPA8 |
| MF | vascular endothelial growth factor binding | 3 | 1.145038 | 0.002309 | PDGFRA, PDGFRB, KDR |
| MF | C-X3-C chemokine binding | 3 | 1.145038 | 0.002309 | ITGA4, ITGB3, ITGB1 |
| MF | demethylase activity | 3 | 1.145038 | 0.002309 | KDM1A, CYP1A1, CYP1A2 |
| MF | DNA-methyltransferase activity | 3 | 1.145038 | 0.002309 | MGMT, DNMT1, DNMT3B |
| MF | growth factor receptor binding | 3 | 1.145038 | 0.0014 | APP, FYN, SRC |
| MF | histone kinase activity | 3 | 1.145038 | 0.0014 | CCNB1, CDK1, CDK2 |
| MF | ErbB-3 class receptor binding | 3 | 1.145038 | 0.0014 | ERBB2, CDK5, PIK3R1 |
| MF | tetrahydrobiopterin binding | 3 | 1.145038 | 0.0014 | NOS1, TH, NOS2 |
| MF | caffeine oxidase activity | 3 | 1.145038 | 0.0014 | CYP3A4, CYP2C9, CYP1A2 |
| MF | indanol dehydrogenase activity | 3 | 1.145038 | 7.07E-04 | AKR1C3, AKR1B10, AKR1C1 |
| MF | histone methyltransferase activity (H3-K27 specific) | 3 | 1.145038 | 7.07E-04 | EHMT1, EZH2, EHMT2 |
| KEGG | Pathways in cancer | 65 | 24.80916 | 1.76E-27 | HSP90AB1, HRAS, PPARD, PTGS2, PGF, MMP9, PPARG, MMP2, MMP1, CTNNB1, EDNRA, AKT1, EDNRB, CCNE1, CASP3, RHOA, PIK3CA, NOS2, CHUK, EGFR, PIK3CG, PRKCA, PTGER1, AR, RET, PTGER2, CTBP2, HSP90AA1, PTGER3, ROCK1, BRAF, PTGER4, PIK3CB, RELA, PIK3CD, CDK6, CDK4, CDK2, DAPK1, MAPK1, CCND1, EP300, VEGFA, PDGFRA, PDGFRB, MDM2, ITGA2B, GRB2, ERBB2, EGLN3, KIT, ITGB1, IGF1R, PTK2, PIK3R1, MAP2K1, FLT3, MET, MAPK10, STAT3, NRAS, GSK3B, IKBKG, MTOR, IKBKB |
| KEGG | PI3K-Akt signaling pathway | 47 | 17.93893 | 5.24E-16 | HSP90AB1, HRAS, MCL1, GRB2, PGF, TLR4, KIT, ITGB3, ITGB1, AKT1, CCNE1, IGF1R, PTK2, PIK3CA, CHUK, PIK3R1, SYK, PRKCA, PIK3CG, EGFR, HSP90AA1, FLT1, MAP2K1, PIK3CB, RELA, MET, PIK3CD, CDK6, ITGA4, CDK4, CDK2, KDR, MAPK1, NRAS, CCND1, GSK3B, VEGFA, IKBKG, PDGFRA, MDM2, PDGFRB, JAK3, MTOR, NGFR, IKBKB, ITGA2B, IL2 |
| KEGG | Proteoglycans in cancer | 40 | 15.26718 | 1.54E-19 | HRAS, TNF, GRB2, MMP9, ERBB2, TLR4, ITGB3, MMP2, PDCD4, ITGB1, SRC, CTNNB1, AKT1, IGF1R, PTK2, CASP3, RHOA, PIK3CA, PIK3R1, PRKCA, PIK3CG, EGFR, PTPN6, BRAF, MAP2K1, ROCK1, PIK3CB, MET, PIK3CD, ESR1, STAT3, KDR, MAPK1, NRAS, CCND1, MAPK14, VEGFA, MDM2, MTOR, PLAU |
| KEGG | MicroRNAs in cancer | 37 | 14.12214 | 6.72E-12 | HRAS, CYP1B1, MCL1, PTGS2, GRB2, MMP9, ERBB2, EZH2, ITGB3, PDCD4, CCNE1, CASP3, RHOA, DNMT3B, PRKCA, EGFR, MAP2K1, ROCK1, MET, PIM1, CDK6, ABCB1, PRKCE, CDC25A, STAT3, NRAS, CCND1, EP300, VEGFA, PDGFRA, PDGFRB, MDM2, DNMT1, ABCC1, MTOR, IKBKB, PLAU |
| KEGG | Focal adhesion | 35 | 13.35878 | 9.24E-15 | HRAS, GRB2, PGF, ERBB2, ITGB3, ITGB1, SRC, CTNNB1, AKT1, IGF1R, PTK2, RHOA, PIK3CA, PIK3R1, PRKCA, PIK3CG, EGFR, FLT1, BRAF, MAP2K1, ROCK1, PIK3CB, MET, PIK3CD, ITGA4, MAPK10, KDR, MAPK1, CCND1, FYN, GSK3B, VEGFA, PDGFRA, PDGFRB, ITGA2B |
| KEGG | Prostate cancer | 32 | 12.21374 | 6.00E-24 | HSP90AB1, HRAS, GRB2, ERBB2, CTNNB1, AKT1, CCNE1, IGF1R, PIK3CA, CHUK, PIK3R1, PIK3CG, EGFR, AR, HSP90AA1, BRAF, MAP2K1, PIK3CB, RELA, PIK3CD, CDK2, MAPK1, NRAS, CCND1, EP300, GSK3B, IKBKG, PDGFRA, PDGFRB, MDM2, MTOR, IKBKB |
| KEGG | Rap1 signaling pathway | 31 | 11.83206 | 1.85E-11 | HRAS, PGF, ITGB3, KIT, ITGB1, SRC, CTNNB1, AKT1, IGF1R, RHOA, PIK3CA, PIK3R1, PRKCA, PIK3CG, EGFR, FLT1, BRAF, MAP2K1, PIK3CB, MET, PIK3CD, KDR, MAPK1, NRAS, MAPK14, VEGFA, PDGFRA, PDGFRB, NGFR, ITGA2B, CALM1 |
| KEGG | Ras signaling pathway | 31 | 11.83206 | 1.21E-10 | HRAS, GRB2, PGF, KIT, AKT1, IGF1R, RHOA, PIK3CA, CHUK, PIK3R1, PRKCA, PIK3CG, EGFR, FLT1, MAP2K1, PIK3CB, RELA, MET, PIK3CD, MAPK10, KDR, MAPK1, NRAS, VEGFA, IKBKG, PLA2G2A, PDGFRA, PDGFRB, NGFR, IKBKB, CALM1 |
| KEGG | Hepatitis B | 30 | 11.45038 | 5.58E-15 | HRAS, TNF, GRB2, MMP9, TLR4, SRC, AKT1, CCNE1, CASP3, PTK2B, PIK3CA, CHUK, PIK3R1, PRKCA, PIK3CG, MAP2K1, PIK3CB, RELA, PIK3CD, CDK6, MAPK10, CDK4, CDK2, STAT3, NRAS, MAPK1, CCND1, EP300, IKBKG, IKBKB |
| KEGG | Viral carcinogenesis | 30 | 11.45038 | 5.28E-11 | HRAS, GRB2, CHEK1, SRC, CCNE1, CASP3, RHOA, PIK3CA, PIK3R1, SYK, PIK3CG, CDK1, PIK3CB, RELA, PIK3CD, CDK6, POLB, CDK4, CDK2, STAT3, MAPK1, NRAS, CCND1, HDAC3, EP300, IKBKG, MDM2, JAK3, EIF2AK2, HDAC6 |
| KEGG | FoxO signaling pathway | 28 | 10.68702 | 4.28E-14 | HRAS, GRB2, AKT1, IGF1R, PRMT1, PIK3CA, CHUK, PIK3R1, PIK3CG, EGFR, BRAF, MAP2K1, PIK3CB, PIK3CD, MAPK10, GRM1, CDK2, STAT3, CCNB1, MAPK1, NRAS, CCND1, EP300, CCNB2, PLK1, MAPK14, MDM2, IKBKB |
| KEGG | HTLV-I infection | 27 | 10.30534 | 5.06E-07 | HRAS, TNF, CHEK1, CTNNB1, AKT1, PIK3CA, CHUK, PIK3R1, TERT, PIK3CG, PIK3CB, RELA, PIK3CD, FDPS, POLB, CDK4, NRAS, CCND1, EP300, GSK3B, LCK, IKBKG, PDGFRA, PDGFRB, JAK3, IKBKB, IL2 |
| KEGG | cAMP signaling pathway | 26 | 9.923664 | 1.36E-08 | PIK3CG, PPARA, PTGER2, PTGER3, ROCK1, BRAF, MAP2K1, PIK3CB, RELA, PIK3CD, CFTR, MAPK10, AKT1, EDNRA, SSTR5, MAPK1, ADRB2, HTR1A, ADRB1, EP300, GIPR, RHOA, PIK3CA, GLP1R, PIK3R1, CALM1 |
| KEGG | T cell receptor signaling pathway | 25 | 9.541985 | 1.93E-14 | PIK3CG, PTPRC, PTPN6, HRAS, TNF, MAP2K1, GRB2, PIK3CB, RELA, PIK3CD, CDK4, AKT1, MAPK1, NRAS, FYN, GSK3B, MAPK14, LCK, IKBKG, RHOA, PIK3CA, IKBKB, CHUK, PIK3R1, IL2 |
| KEGG | Measles | 25 | 9.541985 | 1.47E-11 | PIK3CG, PIK3CB, RELA, TACR1, PIK3CD, TLR4, HSPA1A, CDK6, CDK4, STAT3, CDK2, TLR9, AKT1, CCNE1, CCND1, CSNK2A1, FYN, GSK3B, PIK3CA, JAK3, EIF2AK2, CHUK, HSPA8, PIK3R1, IL2 |
| KEGG | Chemokine signaling pathway | 25 | 9.541985 | 1.76E-08 | HRAS, GRB2, CXCR2, SRC, AKT1, PTK2, PTK2B, RHOA, PIK3CA, PIK3R1, CHUK, PIK3CG, ROCK1, BRAF, MAP2K1, PIK3CB, RELA, PIK3CD, STAT3, MAPK1, NRAS, GSK3B, IKBKG, JAK3, IKBKB |
| KEGG | Estrogen signaling pathway | 24 | 9.160305 | 1.48E-13 | EGFR, HSP90AB1, PIK3CG, OPRM1, HRAS, HSP90AA1, MAP2K1, GRB2, PIK3CB, MMP9, PIK3CD, ESR1, HSPA1A, ESR2, GRM1, MMP2, SRC, AKT1, MAPK1, NRAS, PIK3CA, PIK3R1, HSPA8, CALM1 |
| KEGG | Hepatitis C | 24 | 9.160305 | 9.88E-11 | EGFR, PIK3CG, PPARA, HRAS, TNF, BRAF, GRB2, PIK3CB, RELA, PIK3CD, MAPK10, STAT3, AKT1, NRAS, MAPK1, GSK3B, MAPK14, CD81, IKBKG, PIK3CA, EIF2AK2, IKBKB, CHUK, PIK3R1 |
| KEGG | Acute myeloid leukemia | 23 | 8.778626 | 1.80E-18 | PIK3CG, HRAS, PPARD, MAP2K1, BRAF, FLT3, GRB2, PIK3CB, RELA, PIK3CD, PIM1, KIT, STAT3, AKT1, NRAS, MAPK1, CCND1, IKBKG, PIK3CA, MTOR, IKBKB, CHUK, PIK3R1 |
| KEGG | Glioma | 23 | 8.778626 | 7.66E-17 | PRKCA, EGFR, PIK3CG, HRAS, MAP2K1, BRAF, GRB2, PIK3CB, PIK3CD, CDK6, CDK4, AKT1, IGF1R, MAPK1, NRAS, CCND1, PDGFRA, MDM2, PIK3CA, PDGFRB, MTOR, PIK3R1, CALM1 |
| KEGG | HIF-1 signaling pathway | 23 | 8.778626 | 6.94E-13 | PRKCA, EGFR, PIK3CG, FLT1, MAP2K1, PIK3CB, ERBB2, RELA, PIK3CD, EGLN3, TLR4, STAT3, AKT1, IGF1R, MAPK1, EP300, SERPINE1, VEGFA, PIK3CA, NOS2, MTOR, GAPDH, PIK3R1 |
| KEGG | Influenza A | 23 | 8.778626 | 1.04E-07 | PRKCA, PIK3CG, TNF, MAP2K1, PIK3CB, RELA, PIK3CD, PRSS1, FDPS, TLR4, HSPA1A, MAPK10, PLG, AKT1, MAPK1, EP300, GSK3B, MAPK14, PIK3CA, EIF2AK2, IKBKB, HSPA8, PIK3R1 |
| KEGG | Neuroactive ligand-receptor interaction | 23 | 8.778626 | 2.03E-04 | OPRM1, PTGER1, AVPR2, PTGER2, GABRA1, PTGER3, THRB, PTGER4, TACR1, DRD4, PRSS1, NR3C1, GRM1, PLG, EDNRA, SSTR5, EDNRB, ADRB2, HTR1A, ADRB1, F2, GIPR, GLP1R |
| KEGG | Central carbon metabolism in cancer | 22 | 8.396947 | 9.29E-16 | EGFR, PIK3CG, HRAS, RET, MAP2K1, FLT3, PIK3CB, ERBB2, PIK3CD, MET, KIT, SLC7A5, SIRT3, AKT1, MAPK1, NRAS, G6PD, PDGFRA, PIK3CA, PDGFRB, MTOR, PIK3R1 |
| KEGG | Prolactin signaling pathway | 22 | 8.396947 | 9.36E-15 | PIK3CG, HRAS, MAP2K1, GRB2, PIK3CB, RELA, PIK3CD, TH, ESR1, MAPK10, ESR2, SRC, STAT3, AKT1, MAPK1, NRAS, CYP17A1, CCND1, GSK3B, MAPK14, PIK3CA, PIK3R1 |
| KEGG | Progesterone-mediated oocyte maturation | 22 | 8.396947 | 7.96E-13 | HSP90AB1, PIK3CG, CDK1, HSP90AA1, MAP2K1, BRAF, PIK3CB, PIK3CD, MAPK10, CDC25A, CDK2, PGR, AKT1, CCNB1, IGF1R, MAPK1, RPS6KA3, CCNB2, PLK1, MAPK14, PIK3CA, PIK3R1 |
| KEGG | Thyroid hormone signaling pathway | 22 | 8.396947 | 2.34E-10 | PRKCA, PIK3CG, HRAS, THRB, MAP2K1, PIK3CB, PIK3CD, ESR1, ITGB3, SRC, CTNNB1, AKT1, MAPK1, NRAS, CCND1, HDAC3, EP300, GSK3B, MDM2, PIK3CA, MTOR, PIK3R1 |
| KEGG | Osteoclast differentiation | 22 | 8.396947 | 2.84E-09 | PIK3CG, TNF, MAP2K1, GRB2, PIK3CB, RELA, PPARG, PIK3CD, MAPK10, ITGB3, BTK, AKT1, MAPK1, FYN, MAPK14, LCK, IKBKG, PIK3CA, IKBKB, CHUK, PIK3R1, SYK |
| KEGG | Regulation of actin cytoskeleton | 22 | 8.396947 | 9.71E-06 | EGFR, PIK3CG, HRAS, ROCK1, MAP2K1, BRAF, PIK3CB, PIK3CD, ITGB3, ITGA4, ITGB1, SRC, NRAS, MAPK1, PTK2, F2, RHOA, PDGFRA, PIK3CA, PDGFRB, PIK3R1, ITGA2B |
| KEGG | MAPK signaling pathway | 22 | 8.396947 | 1.53E-04 | PRKCA, EGFR, HRAS, TNF, MAP2K1, BRAF, GRB2, RELA, HSPA1A, MAPK10, AKT1, NRAS, MAPK1, CASP3, RPS6KA3, MAPK14, IKBKG, PDGFRA, PDGFRB, IKBKB, CHUK, HSPA8 |
| KEGG | Pancreatic cancer | 21 | 8.015267 | 1.78E-14 | EGFR, PIK3CG, MAP2K1, BRAF, PIK3CB, ERBB2, RELA, PIK3CD, CDK6, MAPK10, CDK4, STAT3, AKT1, MAPK1, CCND1, IKBKG, VEGFA, PIK3CA, IKBKB, CHUK, PIK3R1 |
| KEGG | Chronic myeloid leukemia | 21 | 8.015267 | 1.59E-13 | PIK3CG, HRAS, CTBP2, MAP2K1, BRAF, GRB2, PIK3CB, RELA, PIK3CD, CDK6, CDK4, AKT1, MAPK1, NRAS, CCND1, IKBKG, MDM2, PIK3CA, IKBKB, CHUK, PIK3R1 |
| KEGG | Small cell lung cancer | 21 | 8.015267 | 4.79E-12 | PIK3CG, FHIT, PTGS2, PIK3CB, RELA, PIK3CD, CDK6, CDK4, ITGB1, CDK2, AKT1, CCNE1, PTK2, CCND1, IKBKG, PIK3CA, NOS2, IKBKB, CHUK, PIK3R1, ITGA2B |
| KEGG | Neurotrophin signaling pathway | 21 | 8.015267 | 3.43E-09 | PIK3CG, HRAS, MAP2K1, BRAF, GRB2, PIK3CB, RELA, PIK3CD, MAPK10, AKT1, NRAS, MAPK1, RPS6KA3, GSK3B, MAPK14, RHOA, PIK3CA, NGFR, IKBKB, PIK3R1, CALM1 |
| KEGG | Sphingolipid signaling pathway | 21 | 8.015267 | 3.43E-09 | PRKCA, PIK3CG, HRAS, TNF, ROCK1, MAP2K1, PIK3CB, RELA, PIK3CD, MAPK10, PRKCE, AKT1, NRAS, MAPK1, FYN, MAPK14, RHOA, PIK3CA, CTSD, ABCC1, PIK3R1 |
| KEGG | B cell receptor signaling pathway | 20 | 7.633588 | 7.85E-13 | PIK3CG, PTPN6, HRAS, MAP2K1, GRB2, PIK3CB, RELA, PIK3CD, BTK, AKT1, NRAS, MAPK1, GSK3B, CD81, IKBKG, PIK3CA, IKBKB, CHUK, PIK3R1, SYK |
| KEGG | Melanoma | 20 | 7.633588 | 1.38E-12 | EGFR, PIK3CG, HRAS, MAP2K1, BRAF, PIK3CB, PIK3CD, MET, CDK6, CDK4, AKT1, IGF1R, NRAS, MAPK1, CCND1, PDGFRA, MDM2, PIK3CA, PDGFRB, PIK3R1 |
| KEGG | ErbB signaling pathway | 20 | 7.633588 | 6.82E-11 | PRKCA, EGFR, PIK3CG, HRAS, MAP2K1, BRAF, GRB2, PIK3CB, ERBB2, PIK3CD, MAPK10, SRC, AKT1, NRAS, MAPK1, PTK2, GSK3B, PIK3CA, MTOR, PIK3R1 |
| KEGG | Chagas disease (American trypanosomiasis) | 20 | 7.633588 | 1.74E-09 | PIK3CG, TNF, PIK3CB, RELA, PIK3CD, TLR4, MAPK10, TLR9, AKT1, MAPK1, ACE, MAPK14, IKBKG, SERPINE1, PIK3CA, NOS2, IKBKB, CHUK, PIK3R1, IL2 |
| KEGG | TNF signaling pathway | 20 | 7.633588 | 2.87E-09 | PIK3CG, TNF, PTGS2, MAP2K1, PIK3CB, MMP9, RELA, PIK3CD, MAPK10, MMP3, MMP14, AKT1, MAPK1, CASP3, MAPK14, IKBKG, PIK3CA, IKBKB, CHUK, PIK3R1 |
| KEGG | Insulin signaling pathway | 20 | 7.633588 | 2.07E-07 | PIK3CG, HRAS, MAP2K1, BRAF, GRB2, PIK3CB, PIK3CD, FBP1, MAPK10, AKT1, NRAS, MAPK1, GSK3B, FASN, PIK3CA, MTOR, PTPN1, IKBKB, PIK3R1, CALM1 |
| KEGG | Non-small cell lung cancer | 19 | 7.251908 | 1.63E-13 | PRKCA, EGFR, PIK3CG, FHIT, HRAS, MAP2K1, BRAF, GRB2, PIK3CB, ERBB2, PIK3CD, CDK6, CDK4, AKT1, NRAS, MAPK1, CCND1, PIK3CA, PIK3R1 |
| KEGG | Natural killer cell mediated cytotoxicity | 19 | 7.251908 | 1.51E-07 | PRKCA, PIK3CG, PTPN6, HRAS, TNF, BRAF, MAP2K1, GRB2, PIK3CB, PIK3CD, NRAS, MAPK1, CASP3, FYN, PTK2B, LCK, PIK3CA, PIK3R1, SYK |
| KEGG | Fc epsilon RI signaling pathway | 18 | 6.870229 | 7.20E-11 | PRKCA, PIK3CG, HRAS, TNF, MAP2K1, GRB2, PIK3CB, PIK3CD, MAPK10, BTK, AKT1, NRAS, MAPK1, FYN, MAPK14, PIK3CA, PIK3R1, SYK |
| KEGG | Choline metabolism in cancer | 18 | 6.870229 | 4.63E-08 | PRKCA, EGFR, PIK3CG, HRAS, MAP2K1, GRB2, PIK3CB, PIK3CD, MAPK10, AKT1, NRAS, MAPK1, PDGFRA, PIK3CA, PDGFRB, SLC5A7, MTOR, PIK3R1 |
| KEGG | Epstein-Barr virus infection | 18 | 6.870229 | 7.79E-07 | PIK3CG, PIK3CB, RELA, PIK3CD, MAPK10, CDK2, STAT3, AKT1, MAPK14, IKBKG, MDM2, PIK3CA, JAK3, EIF2AK2, IKBKB, CHUK, PIK3R1, SYK |
| KEGG | Platelet activation | 18 | 6.870229 | 1.93E-06 | PIK3CG, ROCK1, PIK3CB, PTGS1, PIK3CD, ITGB3, ITGB1, SRC, BTK, AKT1, MAPK1, FYN, MAPK14, RHOA, PIK3CA, PIK3R1, SYK, ITGA2B |
| KEGG | Calcium signaling pathway | 18 | 6.870229 | 1.34E-04 | PRKCA, EGFR, PTGER1, NOS1, PTGER3, ERBB2, TACR1, GRM1, EDNRA, CD38, EDNRB, ADRB2, ADRB1, PTK2B, PDGFRA, PDGFRB, NOS2, CALM1 |
| KEGG | Endometrial cancer | 17 | 6.48855 | 8.17E-12 | EGFR, PIK3CG, HRAS, MAP2K1, BRAF, GRB2, PIK3CB, ERBB2, PIK3CD, CTNNB1, AKT1, NRAS, MAPK1, CCND1, GSK3B, PIK3CA, PIK3R1 |
| KEGG | VEGF signaling pathway | 17 | 6.48855 | 1.21E-10 | PRKCA, PIK3CG, HRAS, MAP2K1, PTGS2, PIK3CB, PIK3CD, SRC, KDR, AKT1, NRAS, MAPK1, PTK2, MAPK14, VEGFA, PIK3CA, PIK3R1 |
| KEGG | Toll-like receptor signaling pathway | 17 | 6.48855 | 5.51E-07 | PIK3CG, TNF, MAP2K1, PIK3CB, RELA, PIK3CD, TLR4, MAPK10, TLR9, AKT1, MAPK1, MAPK14, IKBKG, PIK3CA, IKBKB, CHUK, PIK3R1 |
| KEGG | Insulin resistance | 17 | 6.48855 | 7.15E-07 | PIK3CG, PPARA, TNF, PIK3CB, RELA, PIK3CD, MAPK10, PRKCE, STAT3, AKT1, RPS6KA3, GSK3B, PIK3CA, MTOR, PTPN1, IKBKB, PIK3R1 |
| KEGG | Toxoplasmosis | 17 | 6.48855 | 9.22E-07 | TNF, RELA, TLR4, HSPA1A, MAPK10, ITGB1, STAT3, AKT1, MAPK1, CASP3, MAPK14, IKBKG, ALOX5, NOS2, IKBKB, HSPA8, CHUK |
| KEGG | Serotonergic synapse | 17 | 6.48855 | 1.05E-06 | PRKCA, HRAS, PTGS2, BRAF, MAP2K1, CYP2C19, CYP2C9, PTGS1, SLC6A4, NRAS, MAPK1, ALOX15, APP, CASP3, HTR1A, ALOX5, ALOX12 |
| KEGG | Signaling pathways regulating pluripotency of stem cells | 17 | 6.48855 | 2.24E-05 | PIK3CG, HRAS, MAP2K1, GRB2, PIK3CB, PIK3CD, STAT3, CTNNB1, AKT1, IGF1R, NRAS, MAPK1, GSK3B, MAPK14, PIK3CA, JAK3, PIK3R1 |
| KEGG | Transcriptional misregulation in cancer | 17 | 6.48855 | 1.90E-04 | IGF1R, PTK2, FLT1, KMT2A, FLT3, MMP9, RELA, ELANE, PPARG, MET, MPO, CDK9, MDM2, NGFR, MMP3, IGFBP3, PLAU |
| KEGG | Tuberculosis | 17 | 6.48855 | 3.71E-04 | TNF, RELA, TLR4, MAPK10, SRC, TLR9, AKT1, VDR, MAPK1, CASP3, EP300, MAPK14, RHOA, CTSD, NOS2, SYK, CALM1 |
| KEGG | Bladder cancer | 16 | 6.10687 | 2.10E-12 | EGFR, HRAS, BRAF, MAP2K1, MMP9, ERBB2, CDK4, MMP2, MMP1, SRC, DAPK1, NRAS, MAPK1, CCND1, VEGFA, MDM2 |
| KEGG | Renal cell carcinoma | 16 | 6.10687 | 4.12E-09 | PIK3CG, HRAS, MAP2K1, BRAF, GRB2, PIK3CB, PIK3CD, MET, EGLN3, AKT1, NRAS, MAPK1, EP300, VEGFA, PIK3CA, PIK3R1 |
| KEGG | Leukocyte transendothelial migration | 16 | 6.10687 | 8.15E-06 | PRKCA, PIK3CG, ROCK1, PIK3CB, MMP9, PIK3CD, ITGA4, ITGB1, MMP2, CTNNB1, PTK2, PTK2B, MAPK14, RHOA, PIK3CA, PIK3R1 |
| KEGG | Colorectal cancer | 15 | 5.725191 | 1.51E-08 | PIK3CG, BRAF, MAP2K1, PIK3CB, PIK3CD, MAPK10, CTNNB1, AKT1, MAPK1, CASP3, CCND1, GSK3B, RHOA, PIK3CA, PIK3R1 |
| KEGG | Inflammatory mediator regulation of TRP channels | 15 | 5.725191 | 5.60E-06 | PRKCA, PIK3CG, PTGER2, TRPM8, PTGER4, PIK3CB, PIK3CD, MAPK10, PRKCE, SRC, MAPK14, PIK3CA, PIK3R1, ALOX12, CALM1 |
| KEGG | Gap junction | 14 | 5.343511 | 8.36E-06 | PRKCA, EGFR, CDK1, HRAS, MAP2K1, GRB2, GRM1, SRC, NRAS, MAPK1, ADRB1, PDGFRA, PDGFRB, TUBB3 |
| KEGG | GnRH signaling pathway | 14 | 5.343511 | 1.22E-05 | PRKCA, EGFR, HRAS, MAP2K1, GRB2, MAPK10, MMP14, MMP2, SRC, NRAS, MAPK1, PTK2B, MAPK14, CALM1 |
| KEGG | Cholinergic synapse | 14 | 5.343511 | 1.03E-04 | PRKCA, PIK3CG, HRAS, ACHE, MAP2K1, PIK3CB, PIK3CD, AKT1, NRAS, MAPK1, FYN, PIK3CA, SLC5A7, PIK3R1 |
| KEGG | Oocyte meiosis | 14 | 5.343511 | 1.03E-04 | CDK1, AR, MAP2K1, AURKA, CDK2, CCNB1, PGR, CCNE1, MAPK1, IGF1R, RPS6KA3, CCNB2, PLK1, CALM1 |
| KEGG | AMPK signaling pathway | 14 | 5.343511 | 2.93E-04 | PIK3CG, PIK3CB, HMGCR, PIK3CD, PPARG, FBP1, CFTR, AKT1, IGF1R, CCND1, FASN, PIK3CA, MTOR, PIK3R1 |
| KEGG | Cell cycle | 14 | 5.343511 | 3.18E-04 | CDK1, CHEK1, CDK6, CDK4, CDK2, CDC25A, CCNB1, CCNE1, CCND1, EP300, CCNB2, PLK1, GSK3B, MDM2 |
| KEGG | Jak-STAT signaling pathway | 14 | 5.343511 | 0.001411 | PIK3CG, AKT1, PTPN6, CCND1, EP300, PIK3CB, GRB2, PIK3CD, PIM1, PIK3CA, JAK3, PIK3R1, STAT3, IL2 |
| KEGG | Non-alcoholic fatty liver disease (NAFLD) | 14 | 5.343511 | 0.002037 | PIK3CG, AKT1, PPARA, CASP3, TNF, PIK3CB, GSK3B, RELA, PIK3CD, ERN1, PIK3CA, MAPK10, IKBKB, PIK3R1 |
| KEGG | Steroid hormone biosynthesis | 13 | 4.961832 | 4.63E-07 | AKR1C3, CYP3A4, CYP17A1, CYP1B1, CYP1A1, HSD17B1, HSD11B1, HSD11B2, SRD5A1, HSD17B3, CYP1A2, AKR1C1, CYP19A1 |
| KEGG | mTOR signaling pathway | 13 | 4.961832 | 4.63E-07 | PRKCA, PIK3CG, AKT1, MAPK1, RPS6KA3, TNF, BRAF, PIK3CB, PIK3CD, PIK3CA, MTOR, IKBKB, PIK3R1 |
| KEGG | Apoptosis | 13 | 4.961832 | 9.92E-07 | PIK3CG, AKT1, CASP3, TNF, PIK3CB, RELA, IKBKG, PIK3CD, PIK3CA, IKBKB, CHUK, PIK3R1, CAPN1 |
| KEGG | p53 signaling pathway | 13 | 4.961832 | 2.36E-06 | CDK1, CDK6, CHEK1, CDK4, CDK2, CCNB1, CCNE1, CCND1, CASP3, CCNB2, SERPINE1, MDM2, IGFBP3 |
| KEGG | Adherens junction | 13 | 4.961832 | 4.47E-06 | EGFR, PTPN6, ERBB2, MET, SRC, CTNNB1, IGF1R, MAPK1, CSNK2A1, EP300, FYN, RHOA, PTPN1 |
| KEGG | Amoebiasis | 13 | 4.961832 | 2.64E-04 | PRKCA, PIK3CG, ARG1, PTK2, CASP3, TNF, PIK3CB, RELA, PIK3CD, PIK3CA, TLR4, NOS2, PIK3R1 |
| KEGG | Oxytocin signaling pathway | 13 | 4.961832 | 0.005446 | EGFR, PRKCA, HRAS, ROCK1, MAP2K1, PTGS2, SRC, CD38, NRAS, MAPK1, CCND1, RHOA, CALM1 |
| KEGG | Regulation of lipolysis in adipocytes | 12 | 4.580153 | 2.43E-06 | PIK3CG, AKT1, ADRB2, PTGER3, ADRB1, PTGS2, PIK3CB, PIK3CD, PTGS1, PIK3CA, FABP4, PIK3R1 |
| KEGG | Epithelial cell signaling in Helicobacter pylori infection | 12 | 4.580153 | 1.50E-05 | EGFR, CASP3, MAPK14, RELA, IKBKG, MET, ADAM17, CXCR2, MAPK10, IKBKB, SRC, CHUK |
| KEGG | Fc gamma R-mediated phagocytosis | 12 | 4.580153 | 1.30E-04 | PRKCA, PIK3CG, AKT1, MAPK1, PTPRC, MAP2K1, PIK3CB, PIK3CD, PIK3CA, PRKCE, PIK3R1, SYK |
| KEGG | NF-kappa B signaling pathway | 12 | 4.580153 | 1.78E-04 | TNF, CSNK2A1, PTGS2, RELA, LCK, IKBKG, TLR4, IKBKB, PLAU, CHUK, SYK, BTK |
| KEGG | Type II diabetes mellitus | 11 | 4.198473 | 4.03E-06 | PIK3CG, MAPK1, TNF, PIK3CB, PIK3CD, PIK3CA, MAPK10, MTOR, PRKCE, IKBKB, PIK3R1 |
| KEGG | Pertussis | 11 | 4.198473 | 2.26E-04 | MAPK1, CASP3, TNF, MAPK14, RELA, RHOA, TLR4, MAPK10, NOS2, ITGB1, CALM1 |
| KEGG | Bacterial invasion of epithelial cells | 11 | 4.198473 | 3.13E-04 | PIK3CG, PTK2, PIK3CB, PIK3CD, MET, RHOA, PIK3CA, ITGB1, PIK3R1, SRC, CTNNB1 |
| KEGG | Melanogenesis | 11 | 4.198473 | 0.002221 | PRKCA, EDNRB, MAPK1, NRAS, HRAS, EP300, MAP2K1, GSK3B, KIT, CTNNB1, CALM1 |
| KEGG | NOD-like receptor signaling pathway | 10 | 3.816794 | 1.08E-04 | HSP90AB1, MAPK1, TNF, HSP90AA1, MAPK14, RELA, IKBKG, MAPK10, IKBKB, CHUK |
| KEGG | Arachidonic acid metabolism | 10 | 3.816794 | 2.13E-04 | AKR1C3, ALOX15, CYP2C19, PTGS2, PTGES, CYP2C9, PTGS1, PLA2G2A, ALOX5, ALOX12 |
| KEGG | Shigellosis | 10 | 3.816794 | 3.09E-04 | MAPK1, ROCK1, MAPK14, RELA, IKBKG, MAPK10, IKBKB, ITGB1, SRC, CHUK |
| KEGG | Long-term potentiation | 10 | 3.816794 | 3.91E-04 | PRKCA, MAPK1, NRAS, RPS6KA3, HRAS, EP300, BRAF, MAP2K1, GRM1, CALM1 |
| KEGG | Adipocytokine signaling pathway | 10 | 3.816794 | 6.10E-04 | AKT1, PPARA, TNF, RELA, IKBKG, MAPK10, MTOR, IKBKB, CHUK, STAT3 |
| KEGG | Leishmaniasis | 10 | 3.816794 | 6.78E-04 | MAPK1, PTPN6, TNF, PTGS2, MAPK14, RELA, TLR4, ITGA4, NOS2, ITGB1 |
| KEGG | Thyroid cancer | 9 | 3.435115 | 4.04E-06 | MAPK1, NRAS, HRAS, CCND1, RET, BRAF, MAP2K1, PPARG, CTNNB1 |
| KEGG | Ovarian steroidogenesis | 9 | 3.435115 | 2.34E-04 | AKR1C3, IGF1R, CYP17A1, CYP1B1, CYP1A1, PTGS2, HSD17B1, ALOX5, CYP19A1 |
| KEGG | Long-term depression | 9 | 3.435115 | 9.59E-04 | PRKCA, MAPK1, NRAS, IGF1R, HRAS, NOS1, BRAF, MAP2K1, GRM1 |
| KEGG | Chemical carcinogenesis | 9 | 3.435115 | 0.006024 | CYP3A4, CYP1B1, CYP1A1, CYP2C19, PTGS2, CYP2C9, GSTK1, HSD11B1, CYP1A2 |
| KEGG | Hematopoietic cell lineage | 9 | 3.435115 | 0.009892 | CD38, TNF, FLT3, MME, ANPEP, ITGA4, KIT, ITGB3, ITGA2B |
| KEGG | Aldosterone-regulated sodium reabsorption | 8 | 3.053435 | 3.16E-04 | PRKCA, PIK3CG, MAPK1, PIK3CB, PIK3CD, HSD11B2, PIK3CA, PIK3R1 |
| KEGG | Pathogenic Escherichia coli infection | 8 | 3.053435 | 0.001673 | PRKCA, ROCK1, FYN, RHOA, TLR4, ITGB1, TUBB3, CTNNB1 |
| KEGG | Renin secretion | 8 | 3.053435 | 0.006151 | EDNRA, PTGER2, ACE, ADRB2, ADRB1, PTGER4, REN, CALM1 |
| KEGG | Linoleic acid metabolism | 6 | 2.290076 | 0.002807 | CYP3A4, ALOX15, CYP2C19, CYP2C9, PLA2G2A, CYP1A2 |
| KEGG | Renin-angiotensin system | 5 | 1.908397 | 0.007258 | PREP, ACE, REN, MME, ANPEP |
